# Design, Synthesis, and Evaluation of β‑Lactamase Inhibitors as Potential Therapeutics for Antimicrobial Resistance

**DOI:** 10.1021/acsomega.5c10798

**Published:** 2025-12-01

**Authors:** Sania Batool, Rabia Farid, Syed Sikander Azam, Abbas Hassan

**Affiliations:** † Department of Chemistry, 66757 Quaid-i-Azam University, Islamabad., 45320 Islamabad, Pakistan; ‡ Computational Biology Lab, National Center for Bioinformatics, Quaid-i-Azam University, Islamabad., 45320 Islamabad, Pakistan; § Department of Chemistry, College of Science, 11239 United Arab Emirates University, Al Ain 1551, Abu Dhabi, United Arab Emirates

## Abstract

Antimicrobial resistance is a global health threat affecting millions of people worldwide. The situation has been exacerbated by the emergence of β-lactamase enzymes that can hydrolyze the β-lactam rings within the antibiotics. This makes antibiotics incapable of preventing bacterial infections. The abuse of β-lactam antibiotics is instigating bacterial resistance, thus causing the antibiotics to fail. There is a need to develop newer drugs that can tackle these β-lactamases and overcome bacterial infections. Thienopyrimidines have exhibited diverse biological activities as promising potent antibacterial agents. We synthesized a wide array of substituted thienopyrimidines using a highly divergent approach to introduce different groups involving Suzuki and Sonogashira coupling reactions, aromatic nucleophilic substitution, and alkylation reactions. Novel thienopyrimidines were tested against *Pseudomonas aeruginosa* and *Staphylococcus aureus*, showed inhibitory activity, and were further tested for β-lactamase activity. In-depth investigation revealed that 4-(2-(*tert*-butyl)­thieno­[2,3-*d*]­pyrimidin-4-yl)­morpholine demonstrated exceptional results compared to cefixime (control). To evaluate the compound’s potency in combination therapy, synergistic effects were observed when imipenem was administered alongside our potent compound, resulting in a significantly enhanced minimum inhibitory effect against clinical isolates of *S. aureus*. The findings from this study hold substantial pharmacological significance in addressing the growing threat of bacterial resistance, particularly associated with β-lactamase enzymes.

## Graphical abstract

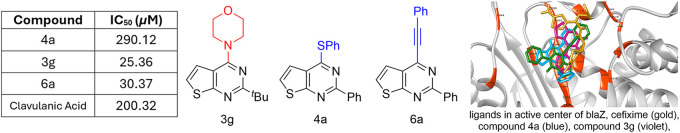

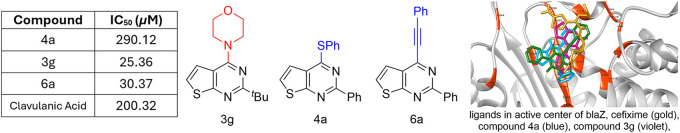

## Introduction

In the era of increasing antibiotic resistance, the Centers for Disease Control and Prevention (CDC) states that approximately 2.8 million infections are due to resistant bacteria, which cause more than 35,000 deaths annually.^1^ While these figures are for the US alone, health agencies all over the world, including the WHO, claim that antimicrobial resistance is set to increase healthcare costs and mortality, with an estimated 10 million deaths annually by the year 2050 if no new drugs are reported and the trends continue.^2^ β-Lactamase is a cataclysmic group of enzymes responsible for increasing bacterial resistance to many known antibiotics, including but not limited to carbapenems, cephalosporins, and penicillin. The mechanism of action of these enzymes is to hydrolyze the β-lactam ring within these antibiotics, thereby efficiently blocking their action.^3^ Due to the accumulating number of β-lactamase-producing bacteria, accompanied by stunted growth in the development of antibiotics, it is imperative for progression in the field of β-lactamase inhibition.^4^ *Staphylococcus aureus* and *Pseudomonas aeruginosa* are two of the most prominent β-lactamase-producing bacteria that are involved in antimicrobial resistance.^5^ The mortality rate for *S. aureus* alone is 10–20%, depending on the site of infection and other conditions contributing to the worsening of the disease.^6^ Similarly, for *P. aeruginosa*, the mortality rate within 30 days of infection lies between 30% and 40%.^7^

Thienopyrimidines are heterocyclic compounds with significant biological importance due to their diverse pharmacological activities. They serve as key structural motifs in various drugs and bioactive molecules.^8,9^ Thienopyrimidines are bioisosteric to purine and quinazoline^10^ and exhibit antiviral, anticancer, and antimicrobial properties, making them valuable in medicinal chemistry. Their versatile nature also extends to their use as enzyme inhibitors and modulators of biological pathways. Researchers continue to explore thienopyrimidines for their potential in the development of new therapeutic agents. For example, apitolisib (GDC-0980) is a significant anticancer agent that functions as a dual inhibitor of phosphoinositide 3-kinase (PI3K) and the mechanistic target of rapamycin (mTOR), both of which are key regulators of cell growth and survival. It has been investigated for the treatment of various cancers, including breast, lung, and renal cancers, by targeting and blocking signaling pathways that drive tumor progression. Apitolisib helps overcome resistance to traditional therapies and enhances the efficacy of other cancer treatments (Figure 1).^11^ Similarly, GNE-477 is a potent dual PI3K/mTOR inhibitor with promising anticancer activity and has shown efficacy in preclinical models, making it a potential candidate for cancer therapy.^12^ Compound **I** is an amino-thienopyrimidine analog, a potent EGFR inhibitor developed from bioisosteric replacement of the pyrrolopyrimidine lead compound,^13^ while thienyl-substituted thienopyrimidine **II** was found to be an excellent inhibitor for FLT_3_.^14^

**Figure 1:**
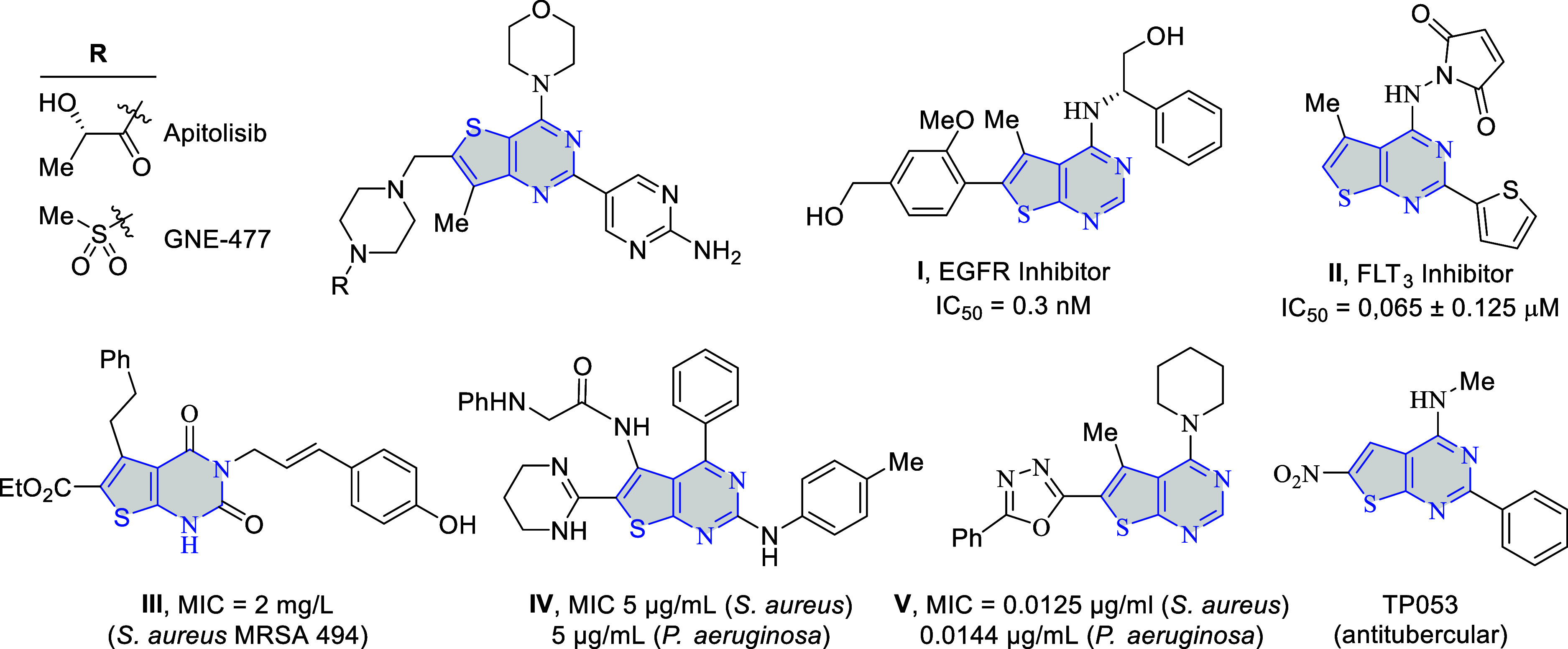
Biologically interesting thienopyrimidine derivatives.

Thienopyrimidines have gained attention as antibacterial agents due to their ability to inhibit key bacterial enzymes and disrupt essential cellular processes. Their unique heterocyclic structure allows them to target bacterial DNA gyrase, topoisomerases, and folate metabolism, leading to bacterial cell death.^15,16^ Several thienopyrimidine derivatives have demonstrated potent activity against Gram-positive and Gram-negative bacteria, including drug-resistant strains. Their structural versatility enables modifications to enhance antibacterial potency and reduce toxicity. For instance, compound **III** displayed potent antibacterial activity against a wide range of antibiotic-resistant bacteria and showed minimal cytotoxicity and no hemolytic activity (Figure 1).^17^ Compound **IV** was tested for antibacterial potential and found to be quite active against *S. aureus* and *P. aeruginosa* at low concentrations (MICs = 4.0–5.0 μg/mL).^18^ Likewise, a thienopyrimidine-based compound **V** was found to be a potent antibacterial against Gram-positive and -negative bacterial strains. It showed improved antibacterial activities compared to that of gentamicin with MIC values in the submicromolar range.^16^ TP053 is a promising thienopyrimidine-based antituberculosis drug, which is active against replicating and nonreplicating *M. tuberculosis*. It has shown potent activity against drug-resistant Gram-negative bacteria including carbapenem-resistant strains. TP053 represents a potential breakthrough in addressing antibiotic resistance and developing next-generation antibacterial therapies.^19^ In this context, this study evaluates the potency of compounds with relevant pharmacological properties with the help of both computational and experimental results.

## Results and Discussion

### Chemistry

To corroborate the biological activities of substituted thienopyrimidines, compounds **2a**–**2c** were synthesized as key intermediates with slight modifications of the reported procedures outlined in Scheme 1. Commercially available 1,4-dithiane-2,5-diol was used to synthesize 2-aminothiophene-3-carboxamide from cyanoacetamide in the presence of triethylamine in methanol. The resulting aminoacetamide was subjected to various acid chlorides to synthesize 2-substituted 2-amidothiophene-3-carboxamides. Sequential intramolecular cyclization of carboxamides was performed in an aqueous solution of sodium hydroxide to synthesize thienopyrimidinones (**1a**–**1c**).^20^ For the activation of C_4_ of thienopyrimidones, the carbonyl moiety was activated by using POCl_3_ in dioxane for the desired chloride (**2a**–**2c**) (Scheme 1).

**Scheme 1:**
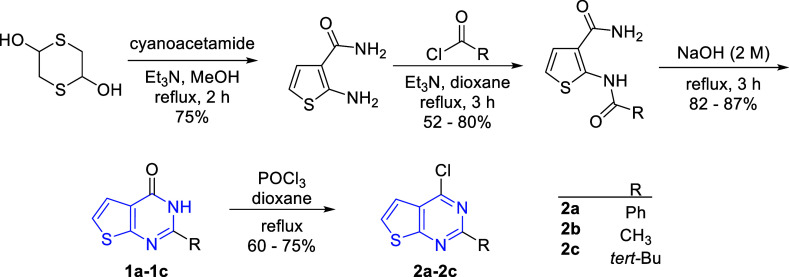
Synthesis of the Substituted Thieno­[2,3-*d*]­pyrimidine Core Structure

The 4-chloro-2-phenylthienopyrimidine **2a** was subjected to different amines and thiols for the synthesis of amine and thioether derivatives. The *S*_*N*_ *Ar* underwent smoothly using potassium carbonate in acetonitrile with primary and secondary amines.^9^ Hence, *N*-methylbenzylamine-derived thienopyrimidine **3a** was isolated in a 64% yield after column chromatography (Scheme 2). Secondary amines such as pyrrolidine and morpholine resulted in substitution products **3b** and **3c** in 66% and 69% isolated yields, respectively. Picolylamine resulted in product **3d** in a 67% yield.

**Scheme 2:**
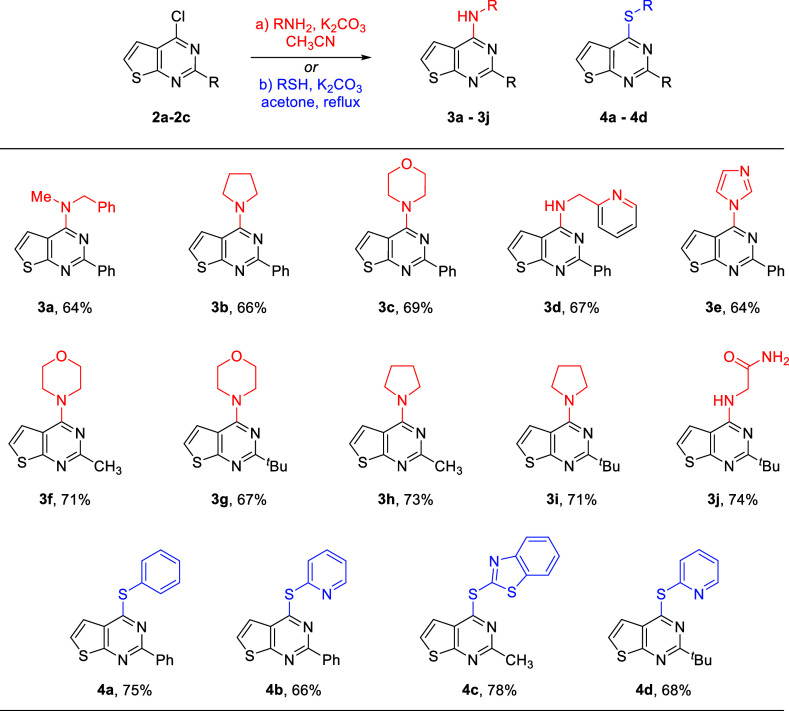
Aromatic Nucleophilic Substitution Reaction of 4-Chlorothienopyrimidine with Amines and Thiols

Interestingly, imidazole also underwent efficient *S*_*N*_ *Ar*, resulting in product **3e** having a 64% yield. Similar reactions were performed with methyl- and *tert*-butyl-substituted thienopyrimidine **2b** and **2c** derivatives, resulting in the corresponding products **3f**–**3i** in excellent yields. Finally, glycinamide resulted in carboxamide-dependent derivative **3j** in 74% yield. Aromatic and heteroaromatic thiols also reacted with intermediates **2a–2c,** and the substitution reaction was carried out in the presence of anhydrous potassium carbonate and dry acetone, which furnished thioether thienopyrimidines (**4a**–**4d**) in suitable yields (Scheme 2).

Palladium-catalyzed cross-coupling reactions provide a useful strategy to introduce aryl and heteroaryl substituents. For aryl and heteroaryl substitution, the Suzuki reaction was carried out with a variety of boronic acids, employing catalytic palladium acetate, triphenylphosphine, and potassium carbonate in THF.^21^ The thienopyrimidine chloride **2a** was treated with phenyl, 4-methoxyphenyl, 4-benzyloxyphenyl, and 3-pyridyl boronic acids, resulting in the aryl thienopyrimidines **5a–5d**. In all cases, the yields were higher except for pyridylboronic acid, resulting in only a 43% isolated yield. This can be attributed to the electron-deficient nature of the pyridine; however, the reactions were not individually optimized. Similar reactivity was found for chlorides **2b** and **2c** with products **5e**–**5g** (Scheme 3).

**Scheme 3:**
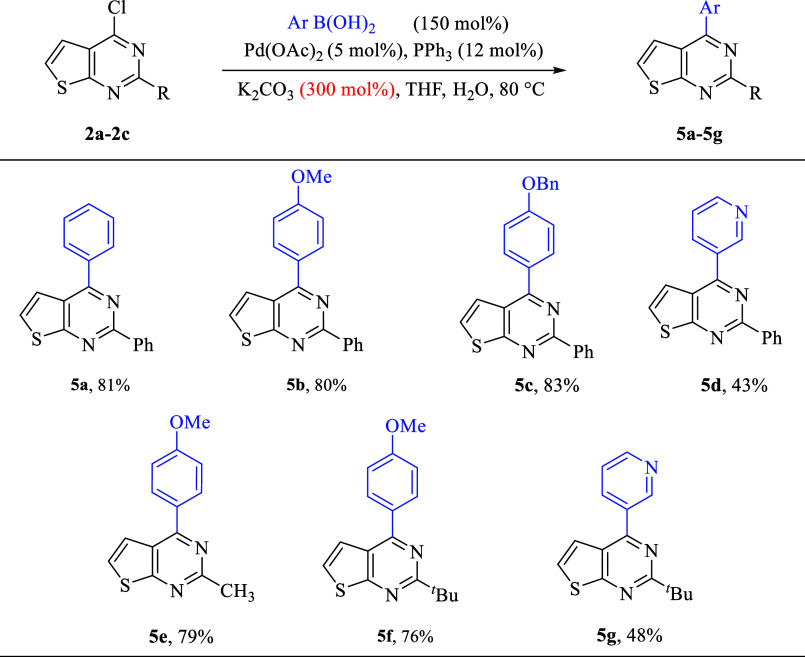
Suzuki Coupling Reaction of 4-Chloro-Substituted Thienopyrimidines

Further derivatization was carried out by using the Sonogashira cross-coupling reaction. For alkynylation, standard reaction conditions were used, consisting of Pd­(OAc)_2_ and CuI in DMF as solvent.^22^ The reaction worked well with phenylacetylene and 1-hexyne to afford products in moderate yields (**6a**–**6c**) (Scheme 4).

**Scheme 4:**
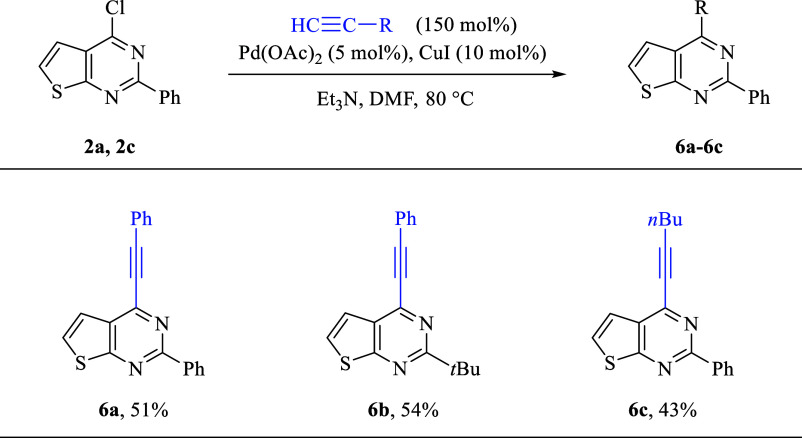
Sonogashira Cross-Coupling Reaction of 4-Chloro-Substituted Thienopyrimidine

To synthesize 4-alkylated thienopyrimidines, key intermediates (**1a**–**1c**) reacted with different alkyl halides in the presence of potassium carbonate, including methyl iodide, ethyl bromide, isobutyl bromide, allyl bromide, propargyl bromide, and benzyl bromide to obtain products (**7a**–**7f**) in good yields (Scheme 5). In all cases, *O*-alkylated thienopyrimidines were the major product. The reaction of thienopyrimidinones was also carried out with ester- and nitrile-containing alkyl bromides; consequently, thienopyrimidines with ethyl propionate, ethyl pentanoate, ethyl hexanoate, and pentane nitrile were isolated (**7j, 7i, 7h,** and **7g**, respectively). According to reported studies, the substituent present at the second position of thienopyrimidinone determines the position of the incoming alkyl group.^23^ The phenyl group favors the *O*-alkylation product, whereas a sterically less hindered group, such as a methyl group, enhances *N*-alkylation, and in the case of the pivaloyl group, no reaction was observed. Hence, with 2-methyl-substituted thienopyrimidinone **1b**, *N*-alkylated product **7k** was the major one with 68% yield (Scheme 5).

**Scheme 5:**
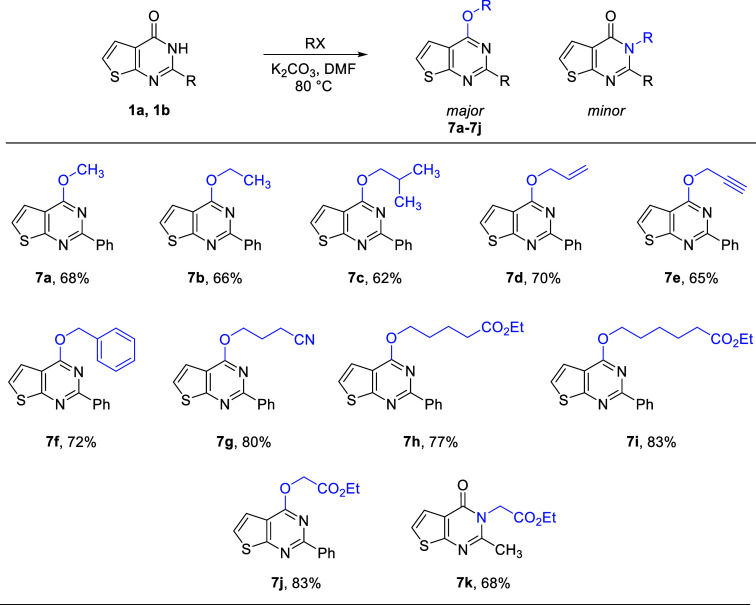
Alkylation Reaction of 2-Substituted Thienopyrimidinones

Hydrolysis of *O*-alkylated products containing propionate, pentanoate, and hexanoate ester groups (**7j, 7h,** and **7i**) was performed using lithium hydroxide as shown in Scheme 6. The resulting products (**8h, 8i,** and **8j**) were obtained with 79%, 72%, and 81% yields, respectively.

**Scheme 6:**
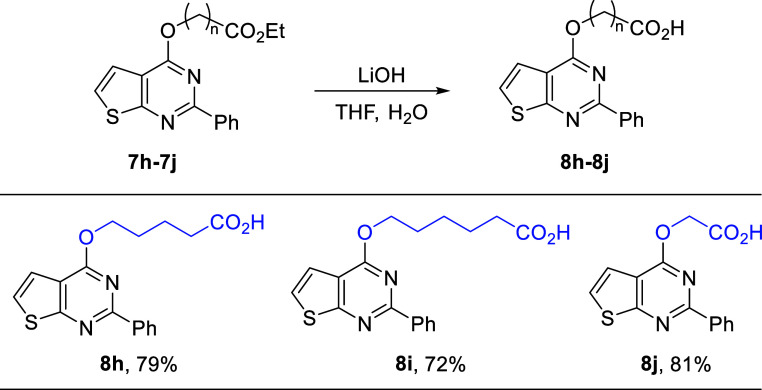
Hydrolysis of Esters Containing Thienopyrimidines

### Biological Study

#### Antibacterial Assays

In the present study, a total of 40 compounds were tested for their antimicrobial tendencies through experimental and computational analyses. The compounds were first tested for their bactericidal nature by performing antimicrobial assays by selecting one bacterial species from the Gram-negative class, i.e., *P. aeruginosa*, and one from the Gram-positive class, *S. aureus*. MDR clinical strains for both bacteria were used to determine the efficacy of compounds in clinical settings. A total of three sets of experiments were performed to level out any discrepancies, and the zones of inhibition (ZOI) were averaged. Cefixime, a third-generation cephalosporin that is effective against most clinical Gram-negative and -positive bacteria, was chosen as the control. After 24 h of incubation at 37 °C, the zones of inhibition ranged from 4 to 18 mm (Table 1) in *P. aeruginosa* and 10 to 20 mm (Table 1) for *S. aureus*. Among the synthesized compounds, 6 compounds (**3e, 5b, 3g, 6a, 5d,** and **4b**) showed antibacterial activity against *S. aureus*, while 9 compounds (**3c, 3e, 3d, 4a, 3g, 3f, 6a, 5d,** and **4b**) showed bactericidal activity against *P. aeruginosa*. The zones of inhibition (ZOI) for these compounds are shown in Table 1. A significant difference is seen in the ZOI between the control and a few other compounds. The ZOI for control were measured at 12 mm for *S. aureus* and 8 mm for *P. aeruginosa*. Among the synthesized compounds, for *S. aureus*, compounds **3e** and **5b** showed better ZOI, i.e., 18 and 20 mm, respectively, which is considerably larger compared to the control. Similarly, for *P. Aeruginosa*, compounds **3e**, **3f**, **6a**, and **4b** showed greater ZOI, i.e., 18, 12, 14, and 18 mm, respectively, compared to the 8 mm zone for the control. The MIC assays were also performed to study the quantitative effect of compounds on antibacterial activity against *P. aeruginosa*. Compound **5b** showed maximum antibacterial activity, having an MIC value of 32 μg/mL against the Gram-positive bacterial strain *S. aureus*, and this value was found to be much higher than that of the reference drug cefixime. The other compound **6a** also showed optimum activity against both bacterial strains, with an MIC value of 38 μg/mL (against *P. aeruginosa*). Similarly, **3f** and **4a** had the same MIC value of 42 μg/mL, which indicates their potency against *P. aeruginosa*.

**Table 1:** Compounds and Their Zones of Inhibition against *S. aureus* and *P. aeruginosa*

S. No.	compound name	zone of inhibition(mm) ± SD		MIC (μg/mL)^a^
		*S. aureus*	*P. aeruginosa*	
1	**3c**	-	5 ± 0.4	74
2	**3e**	18 ± 0.4	18 ± 0.4	52
3	**3d**	-	4 ± 0.4	50
4	**5b**	20 ± 0.4	-	32^b^
5	**4a**	-	8 ± 0.4	42
6	**3g**	12 ± 0.4	5 ± 0.4	62
7	**3f**	-	12 ± 0.4	42
8	**6a**	12 ± 0.4	14 ± 0.4	38
9	**5d**	10 ± 0.4	8 ± 0.4	46
10	**4b**	10 ± 0.4	18 ± 0.4	50
11	cefixime (control)	12 ± 0.4	8 ± 0.4	100 (CLSI susceptible≤ 4, resistant ≥8)

a MIC values of *P. aeruginosa*.

b MIC value of *S. aureus*.

MIC values were interpreted according to the Clinical and Laboratory Standards Institute (CLSI) guidelines. Cefixime was included as a control to demonstrate the multidrug-resistant nature of the clinical isolate. *S. aureus* is inherently less susceptible to cefixime, with CLSI breakpoints defining resistance at ≥ 8 μg/mL. Our measured MIC of 100 μg/mL for the isolate confirms its high level of resistance, serving as a relevant baseline for inactive control. Synthesized compounds exhibited MIC values between 32 and 74 μg/mL. While these concentrations are higher than the clinical breakpoints for highly effective antibiotics such as vancomycin (MIC ≤2 μg/mL), they are significantly more potent than the inactive control, cefixime (MIC 100 μg/mL), against this resistant strain. This suggests potential in vitro activity, demonstrating that further optimization can yield more potent inhibitors.

#### β-Lactamase Activity Assay

The compounds that showed antibacterial potential were further examined for possible β-lactamase inhibitory activity. For this purpose, an Abcam β-lactamase assay kit was utilized, and all observational readings were recorded using a spectrophotometer by measuring the amount of hydrolyzed nitrocefin at an OD of 490 nm. The results obtained are presented in Table 2. Values obtained from the results indicated compound **3g** as an excellent inhibitor with an IC_50_ of 25.36 μM in sharp contrast to the control with a concentration of 200.32 μM (Table 2). A graph of the percent activity is illustrated in Figure 2. Besides this, compound **6a** also showed a reduced IC_50_ of 30.37 μM. Under this scenario, it is reported that a combined regimen might result in synergistic efficacy, which would be extremely effective in the most serious infections.^24^ Since compound **3g** showed exceptional results as a β-lactamase inhibitor, a synergistic assay was also performed to validate this fact.

**Table 2:** β-Lactamase Activity of Selected Compounds at Different Concentrations along with IC_50_ in μM

concentration (μg/mL)	percent activity			
	6a	3g	4a	clavulanic acid
0.09	97.56	95.31	98.65	93.45
1	86.05	74.42	90.70	84.98
5	72.09	55.81	88.37	75.35
10	37.20	37.21	81.39	62.39
50	27.30	27.91	74.42	53.12
100	6.97	9.30	41.86	23.97
150	5.84	6.97	20.34	9.21
200	3.19	2.87	4.59	2.45
IC_50_ μM	30.37	25.36	290.12	200.32

**Figure 2:**
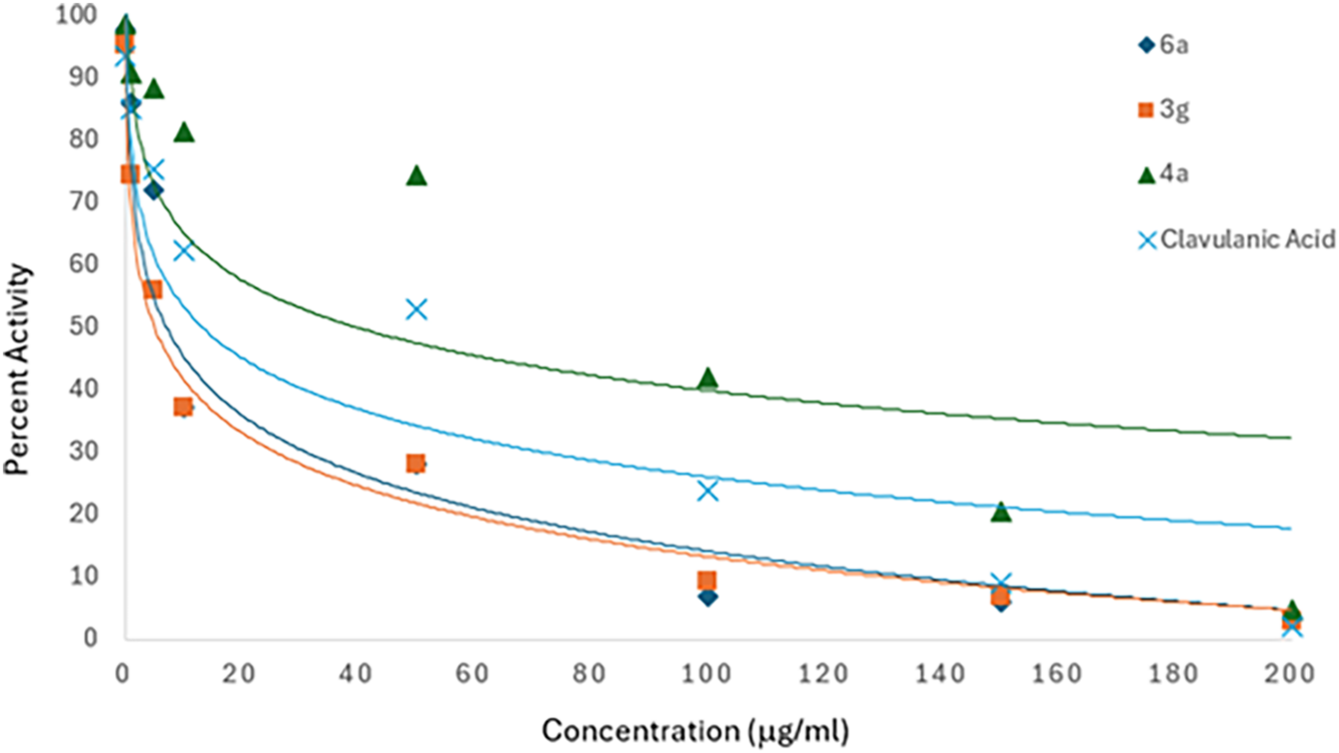
Graphical representation of the hydrolysis of 100 mM nitrocefin used to measure the activity of BlaZ, followed by 5 min preincubation with compounds **3g**, **6a**, and **4a**, and clavulanic acid inhibitor doses using the nonlinear regression curve.

#### Synergistic Assay

A synergistic assay was carried out to assess whether the most potent inhibitor, i.e., compound **3g,** performs better when administered in combination with known β-lactam antibiotics. The results are shown in Table 3. Imipenem and compound **3g** used as controls showed zones of 15 and 10 mm, respectively. Imipenem, alongside compound **3g**, showed a remarkable zone of inhibition of 54 mm at a concentration of 10 μg + 150 μg (Table 3). This shows that while compound **3g** shows some antibacterial activity when used in combination with a broad-spectrum antibiotic such as imipenem, it can produce remarkable results.

**Table 3:** Synergistic Effect of Compound **3g** on Clinical *S. aureus*

S. no.	compound	concentration	zone of inhibition
1	imipenem	10 μg	15 mm
2	3g	150 μg	10 mm
3	imipenem +3g	10 μg + 150 μg	54 mm

## Computational Analyses

### Molecular Docking

To gain computational insights into the interactions of these synthesized compounds in the active sites of known druggable targets from both bacteria, the compounds were divided into two groups and subjected to molecular docking. One group contained compounds that showed only bactericidal activity and no β-lactamase inhibitory activity, while the other group contained compounds that showed β-lactamase inhibitory activity. For antibacterial targets, DDl from *S. aureus* and RMLA from *P. aeruginosa* were selected, and docking was performed using Genetic Optimization for Ligand Docking (GOLD). DDl from *S. aureus* catalyzes *d* -alanine: *d* -alanine synthesis in a two-step process, which is important in the biosynthesis of the bacterial cell wall.^25^ For example, the control showed a gold score of 49.235, while the synthesized compounds showed scores between 43.183 and 51.653, as shown in Table 4. When correlated with antibacterial activity results, compound **5b** exhibited not only a better ZOI compared to the control (20 and 12 mm, respectively) but also a better docking score, i.e., 51.653 (Figure 3). This depicts complete coherence of experimental and computational calculations under the specific terms of values. Furthermore, interaction analyses provide an obvious degree of stability for the complex. This stability is attributed to glutamine at position 506, which forms hydrogen bonds. Moreover, multiple strong van der Waals interactions were observed, enhancing the stability of the complex. The interacting residues have been highlighted in the active site and are shown in Figure 3. Since compound **3e** did not show an inhibitory effect against β-lactamase enzymes, given its good ZOI of 18 vs 8 mm for control, it was assumed that the compound likely exerts its antibacterial effect through a different mechanism.

**Table 4:** GOLD Fitness Score of Selected Compounds Docked against DDl (*S. aureus*) and RMLA (*P. aeruginosa*)

S. no.	compound	GOLD score		S. no	compound	GOLD score	
		*S. aureus*	*P. aeruginosa*			*S. aureus*	*P. aeruginosa*
1	**3c**	-	28.4989	5	**3f**	-	45.5082
2	**3e**	43.1834	51.3588	6	**5d**	49.9516	45.1114
3	**3d**	-	32.6214	7	**4a**	-	50.6911
4	**5b**	51.6531	-	8	cefixime (control)	49.2356	15.8099

**Figure 3:**
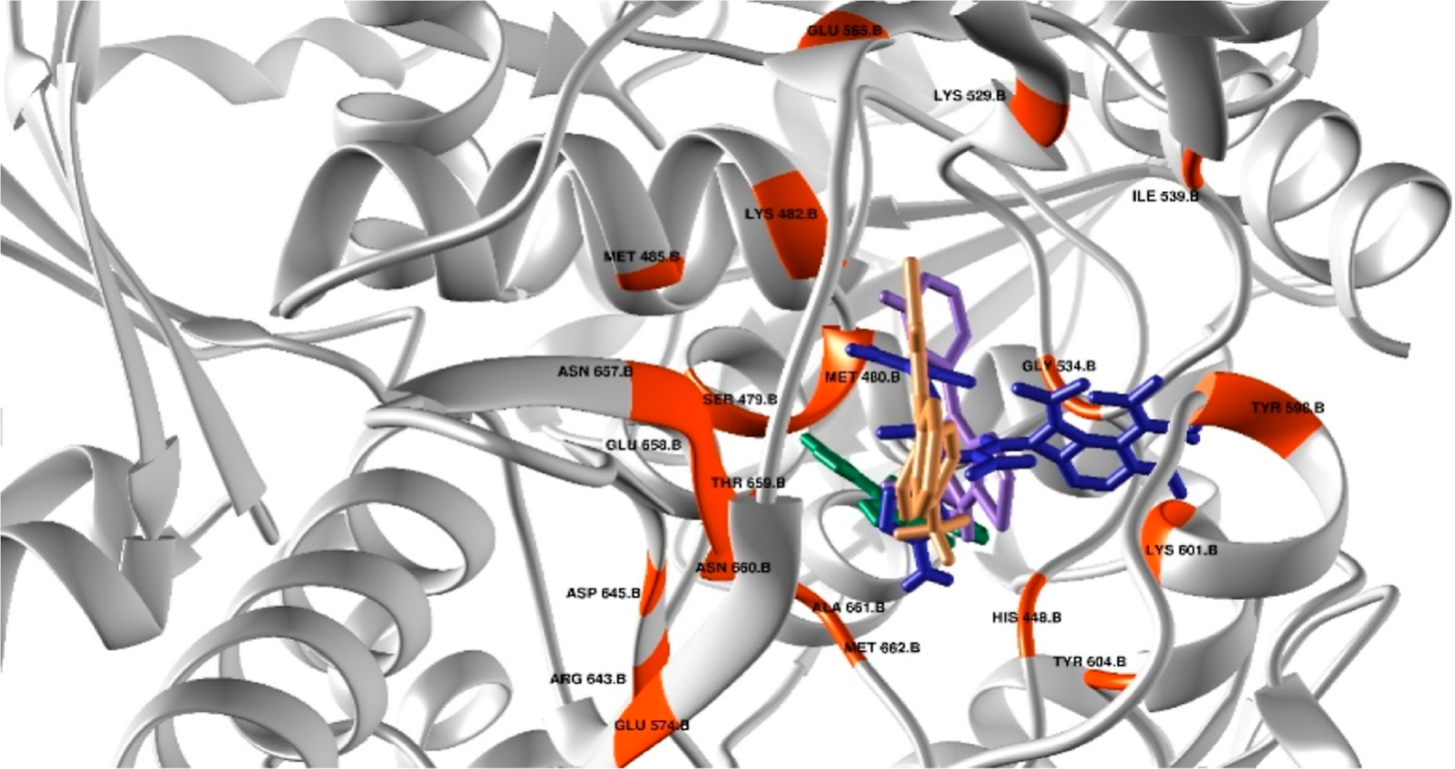
Orientation of ligands in the active center of DDl with interacting residues highlighted in orange. Control cefixime (dark blue), compound 3e (green), compound 5b (brown), and compound 5d (purple).

For *P. aeruginosa* RMLA, compounds **3e** and **4a** exhibited the highest gold scores, 51.358 and 50.691, respectively, compared to the control (15.809) (Table 4). This substantial difference aligns with the observed antibacterial activity, where inhibition zones were 18 and 8 mm, respectively (Figure 6). The interaction analysis reveals that compound **3e** forms a strong hydrogen bond with tyrosine at position 323, accompanied by multiple van der Waals interactions. Similarly, compound **4a** interacts with glycine 236 via a strong hydrogen bond with sulfur, supported by additional van der Waals interactions that stabilize the ligand within the binding pocket (Figure 4).

**Figure 4:**
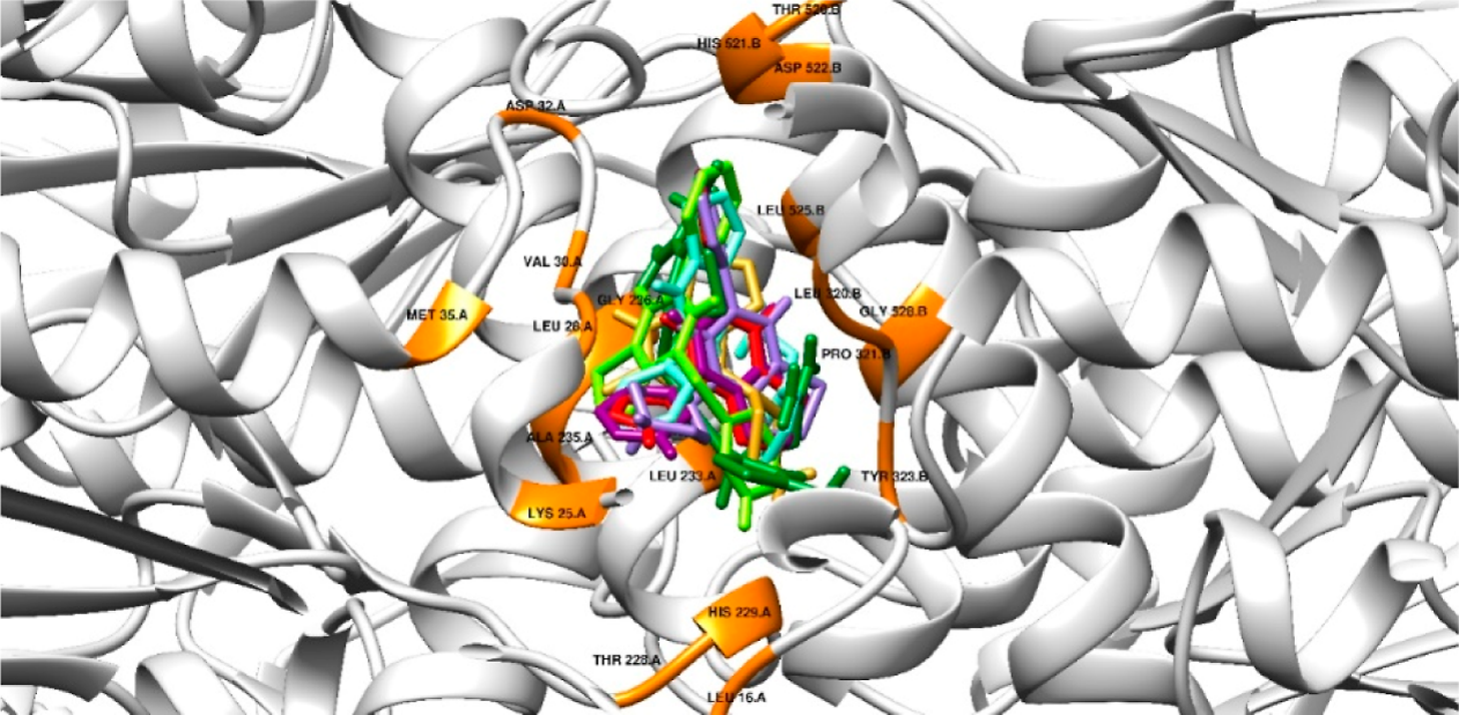
Orientation of ligands in the active center of RMLA with interacting residues highlighted in orange. Control cefixime (green), compound 3c (purple), compound 3e (red), compound 3d (aqua), compound 3f (gold), compound 5d (magenta), and compound 4a (light green).

**Figure 5:**
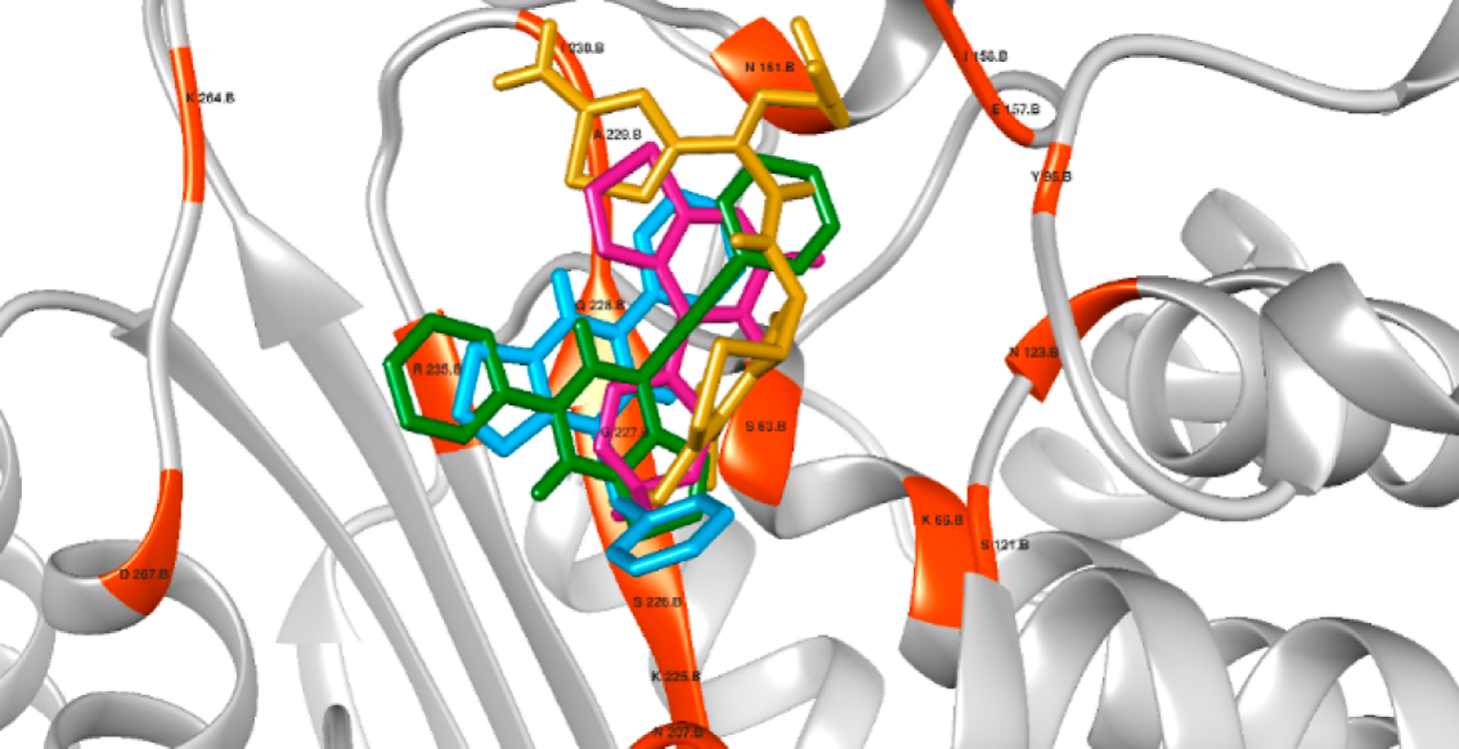
Orientation of ligands in the active center of blaZ with interacting residues highlighted in orange. Control cefixime (gold), compound **4a** (blue), compound **3g** (violet), and compound **6a** (green).

**Figure 6:**
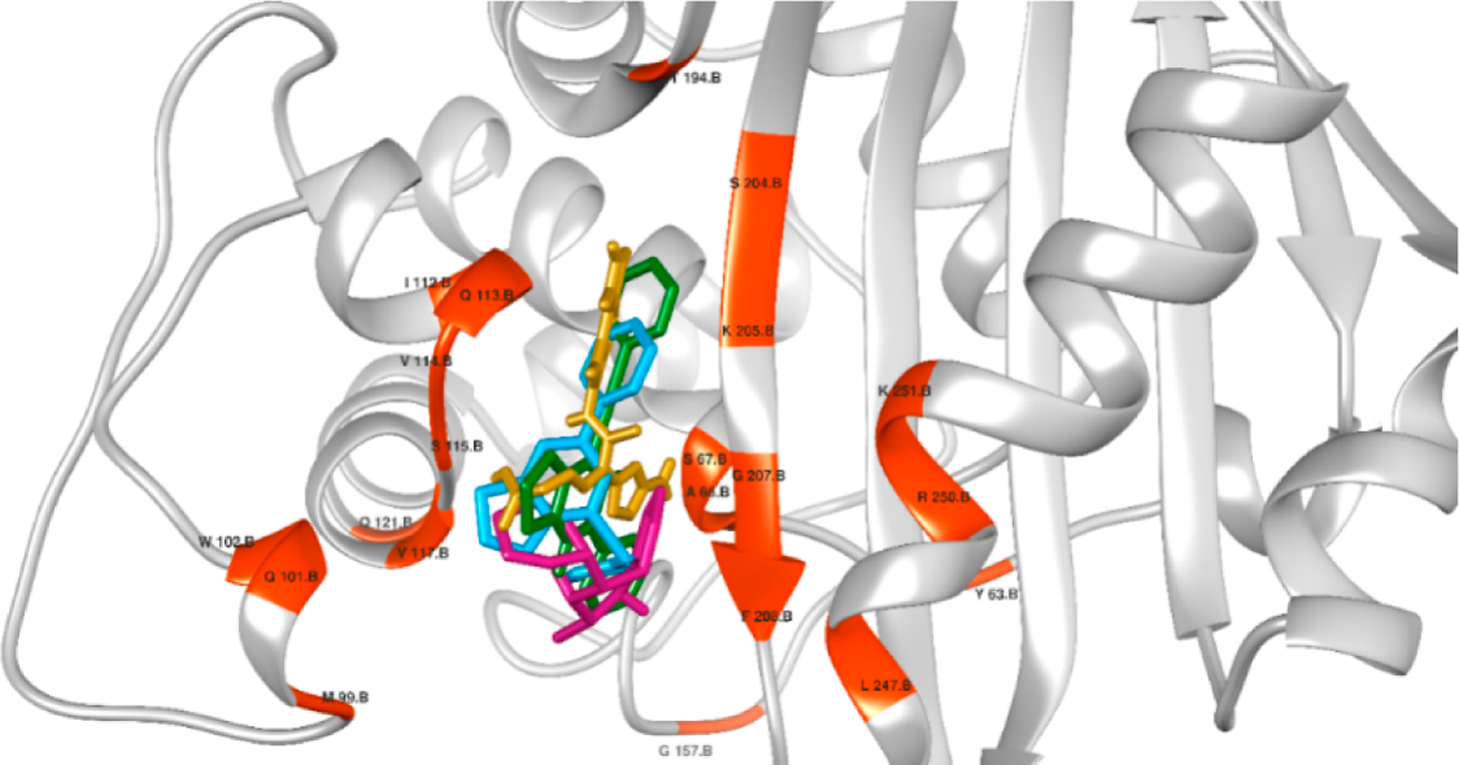
Orientation of ligands in the active center of OXA-10 with interacting residues highlighted in orange. Control cefixime (gold), compound **4a** (blue), compound **3g** (violet), and compound **6a** (green).

For β-lactamase targets, blaZ from *S. aureus* and OXA-10 from *P. aeruginosa* were selected. BlaZ is a class A β-lactamase responsible for penicillin resistance in *S. aureus*, while OXA-10, a class D β-lactamase, also confers resistance to penicillin and is increasingly prevalent. Both enzymes utilize a serine residue in their active sites for substrate hydrolysis. The control exhibited docking scores of 66.328 and 77.731 against *S. aureus* and *P. aeruginosa*, respectively. Compounds **4a**, **3g**, and **6a** showed docking scores of 54.111, 43.192, and 53.901 against blaZ and 62.171, 44.884, and 60.384 against OXA-10 (Table 5). Notably, all serine residues within 5 Å of the docked compounds (Figures 5 and 6) engaged in either hydrogen bonding or van der Waals interactions, reinforcing their potential to occupy the active site and hinder enzymatic function.

**Table 5:** GOLD Fitness Score of Selected Compounds Docked against blaZ (*S. aureus*) and OXA-10 (*P. aeruginosa*)

S. no.	compound	GOLD score	
		*S. aureus*	*P. aeruginosa*
1	4a	54.1114	62.1714
2	3g	43.1925	44.8845
3	6a	53.9015	60.3847
4	cefixime (control)	66.328	77.7312

### MD Simulation

The DDl-**3e** complex was subjected to MD simulations. A 100 ns molecular dynamics simulation was performed for the DDL-**3e** complex to evaluate its structural stability and binding dynamics. The RMSD plot (Figure 7a) revealed a steady increase from ∼1 to ∼3 Å throughout the 100 ns trajectory, indicating gradual conformational adaptation of the protein–ligand complex, stabilizing after around 70 ns. The RMSF plot (Figure 7b) indicated residue-wise flexibility. Most residues displayed moderate fluctuations of 1–2 Å in the glycine-rich areas, except a highly flexible region at residues 351 and 352, where RMSF peaked at 7.5 Å, pointing to the tail end of the dimer. The Rg plot (Figure 7c) maintained relative stability (∼26 Å), with minor fluctuations, suggesting that the overall protein compactness was conserved during the simulation, implying stable folding. *B*-factor values (Figure 7d) mirrored the RMSF data, confirming a localized region of high atomic mobility near residue 350, with peak values exceeding 1500 Å^2^. Other regions remained largely stable.

**Figure 7:**
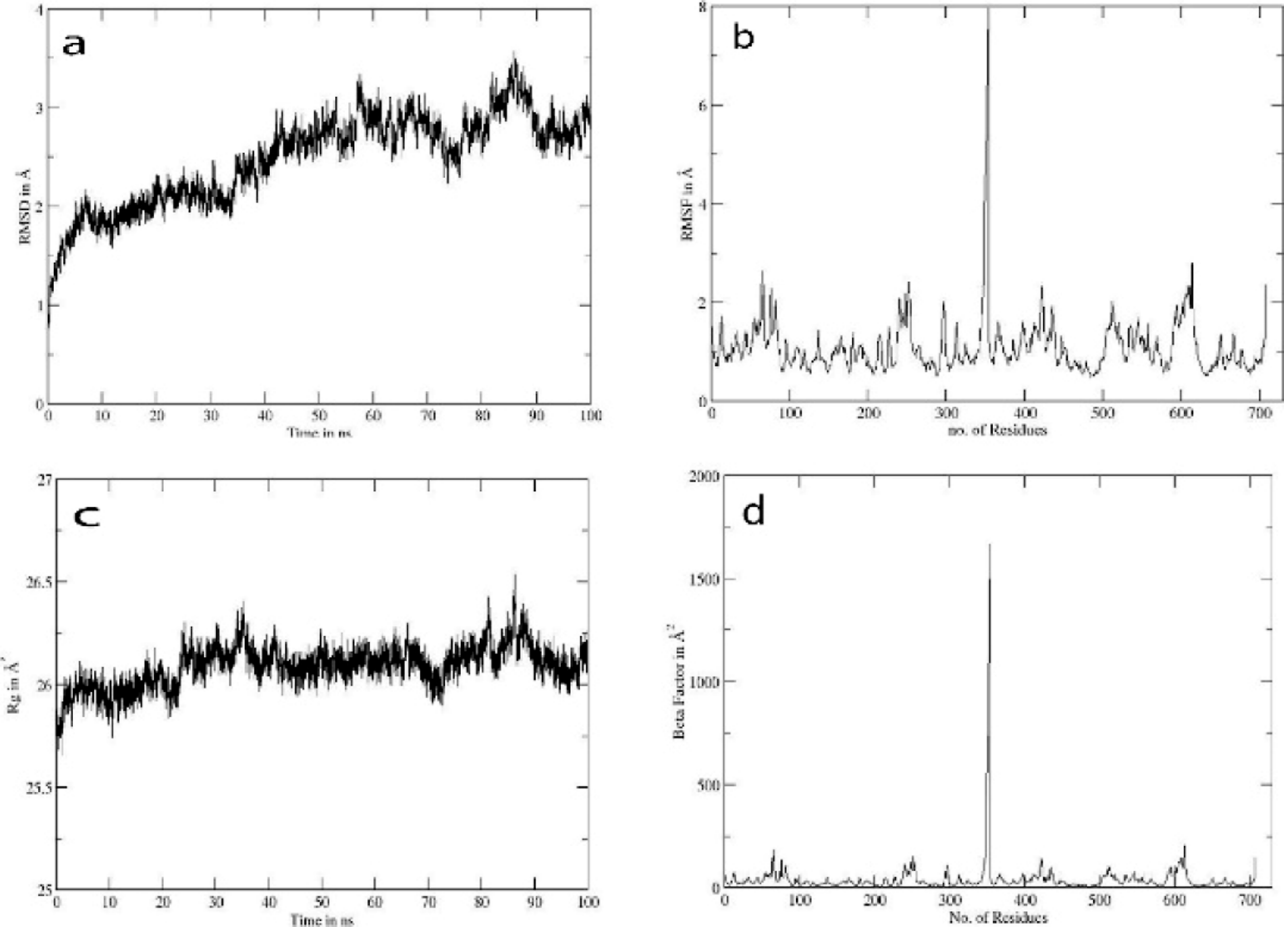
Trajectory analysis for DDL-compound **3e**: (a) shows RMSD in angstroms, (b) shows RMSF in angstroms, (c) shows the radius of gyration, and (d) shows the beta factor.

The increasing RMSD with eventual plateauing suggests a conformational adaptation period, followed by stabilization. The RMSF and *b*-factor analyses revealed a highly flexible region near chain ends not directly involved in ligand binding (Figure 7). Despite this local flexibility, the global protein structure remained compact, as indicated by stable Rg values. Together, these data suggest that compound **3e** forms a stable complex with DDL, potentially interfering with its activity, which could explain the observed antibacterial effect. However, further enzymatic assays would be necessary to confirm DDL inhibition as the mechanism of action.

The 100 ns molecular dynamics simulation revealed the excellent stability of the RmlA-**3f** complex. The RMSD trajectory (Figure 8a) stabilized within the first 20 ns and remained consistently between 1.5 and 2.0 Å, reflecting a stable ligand–protein conformation. The RMSF profile (Figure 8b) displayed limited fluctuations, with most residues remaining below 1.5 Å except for the highest peaks for flexible tail ends and moderate peaks for glycine, while active site residues showed restricted mobility. The radius of gyration (Figure 8c) remained steady at 31.6–32.0 Å, indicating no significant global conformational changes. Additionally, the *b*-factor analysis (Figure 8d) corroborated these findings, showing only localized flexibility in surface loops, while the catalytic core remained stable throughout the simulation. The compound 3f showed a significant zone of inhibition as well as a docking score and hence was subjected to MD simulations where the trajectory analyses confirmed that compound 3f binds firmly within the active site of RmlA, maintaining a tightly bound conformation throughout the 100 ns simulation without notable displacement. This high stability, coupled with minimal active-site flexibility, highlights the potential of 3f as an RmlA inhibitor (Figure 4). The computational evidence is in strong agreement with the in vitro antibacterial assays, where 3f produced inhibition zones larger than those of the standard cefixime. Since RmlA is essential for bacterial cell wall integrity, inhibition of this enzyme by 3f could explain its antibacterial efficacy despite the absence of β-lactamase inhibition. This highlights 3f as a distinct antibacterial scaffold with a mechanism separate from β-lactamase targeting, making it a promising lead for further optimization against resistant bacterial strains.

**Figure 8:**
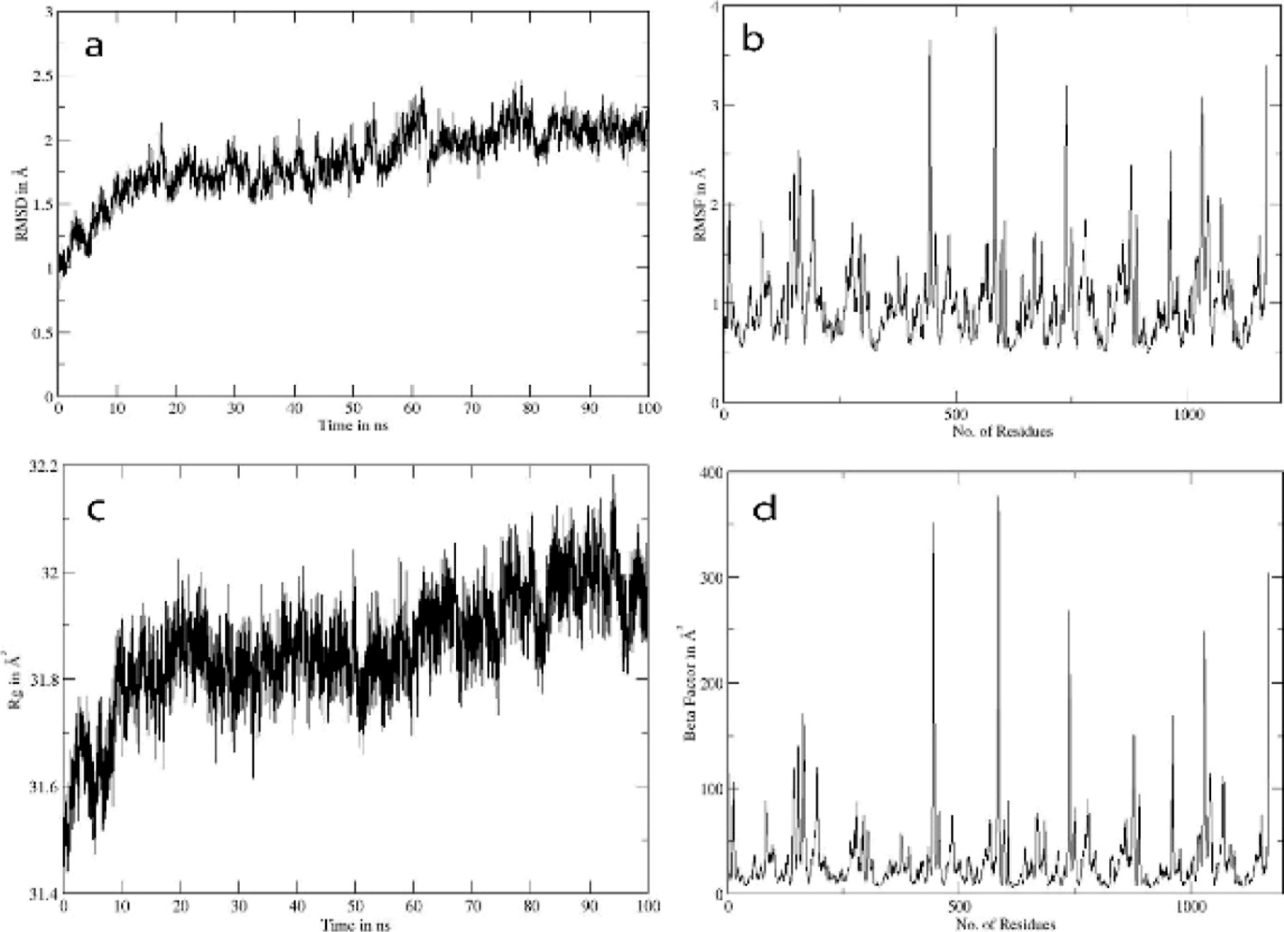
Trajectory analysis for RMLA-compound **3f**: (a) shows RMSD in angstroms, (b) shows RMSF in angstroms, (c) shows the radius of gyration, and (d) shows the beta factor.

Since compound **3g** showed the best result as an inhibitor of BL, MD simulations were performed on both these complexes, which demonstrated that compound **3g** maintained stable binding within both the BlaZ and OXA-10 active sites. The BlaZ-3g and OXA-10-**3g** complexes were subjected to 100 ns molecular dynamics simulations. In the BlaZ-**3g** complex, the RMSD (Figure 9a) stabilized after ∼20 ns and fluctuated around 1.3–1.5 Å, reflecting overall structural stability. The RMSF (Figure 9b) values were generally below 1.5 Å, with a pronounced peak around residues 215–225 of 2.5 Å. This region includes the catalytic serine, which is normally responsible for nucleophilic attack on the β-lactam ring. The radius of gyration (Figure 9c) was consistent at 18.3–18.6 Å, indicating stable global compactness. The *b*-factor profile (Figure 9d) showed moderate flexibility in the same catalytic loop region, confirming local but functionally relevant mobility.

**Figure 9:**
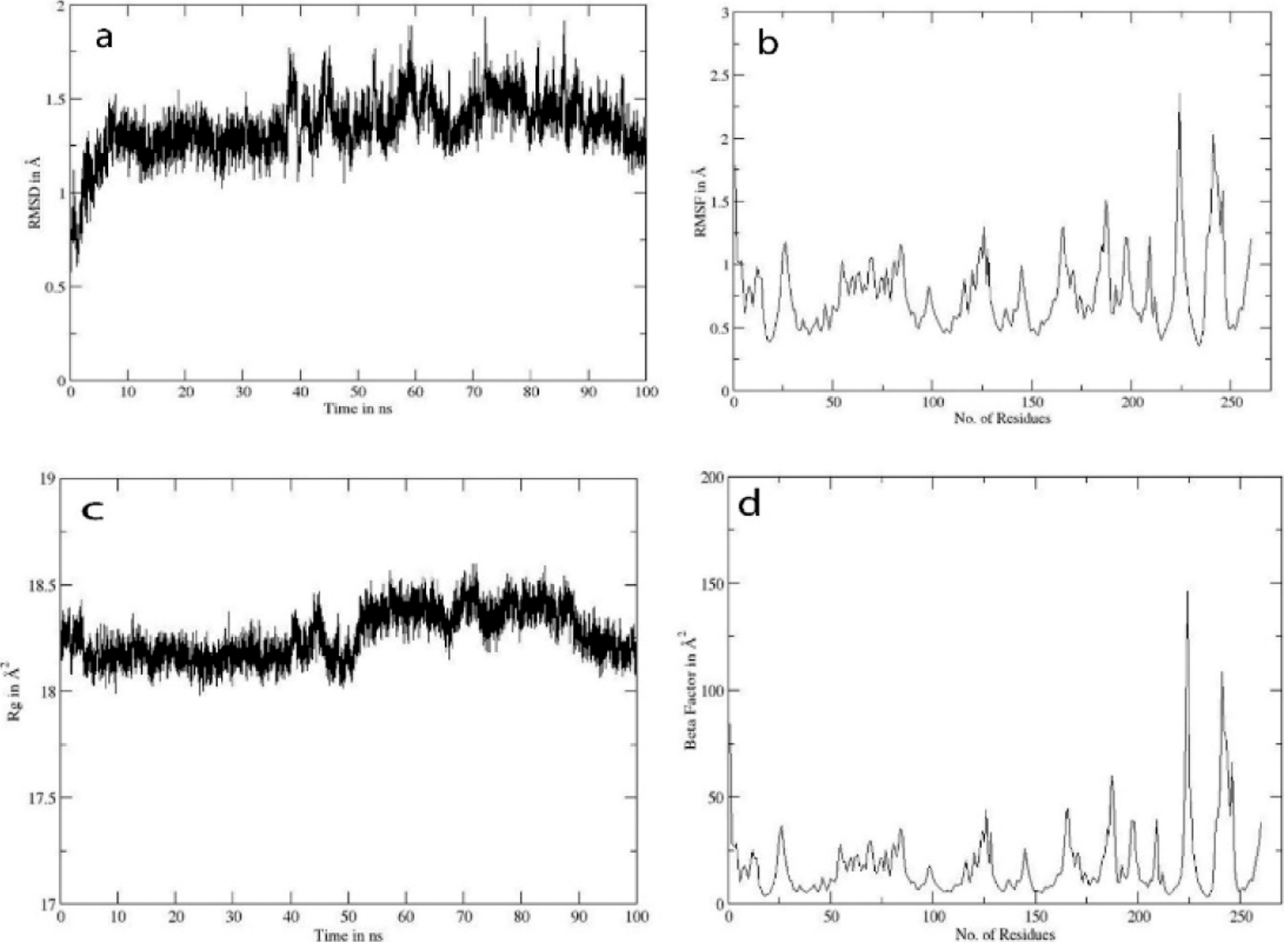
Trajectory analysis for BlaZ-**3g**: (a) shows RMSD in angstroms, (b) shows RMSF in angstroms, (c) shows the radius of gyration, and (d) shows the beta factor.

In the OXA-10-3g complex, the RMSD (Figure 10a) remained stable between 1.4 and 1.6 Å, without significant drift. The RMSF (Figure 10b) indicated low flexibility across most residues (<1.2 Å), except for a sharp spike near residue 414 (>5 Å) corresponding to catalytic serine. The radius of gyration (Figure 10c) remained stable at 24.2–24.4 Å^2^, and the *b*-factor profile (Figure 10d) revealed fluctuations at residue ∼200 and a marked spike at residue 414, again consistent with catalytic serine.

**Figure 10:**
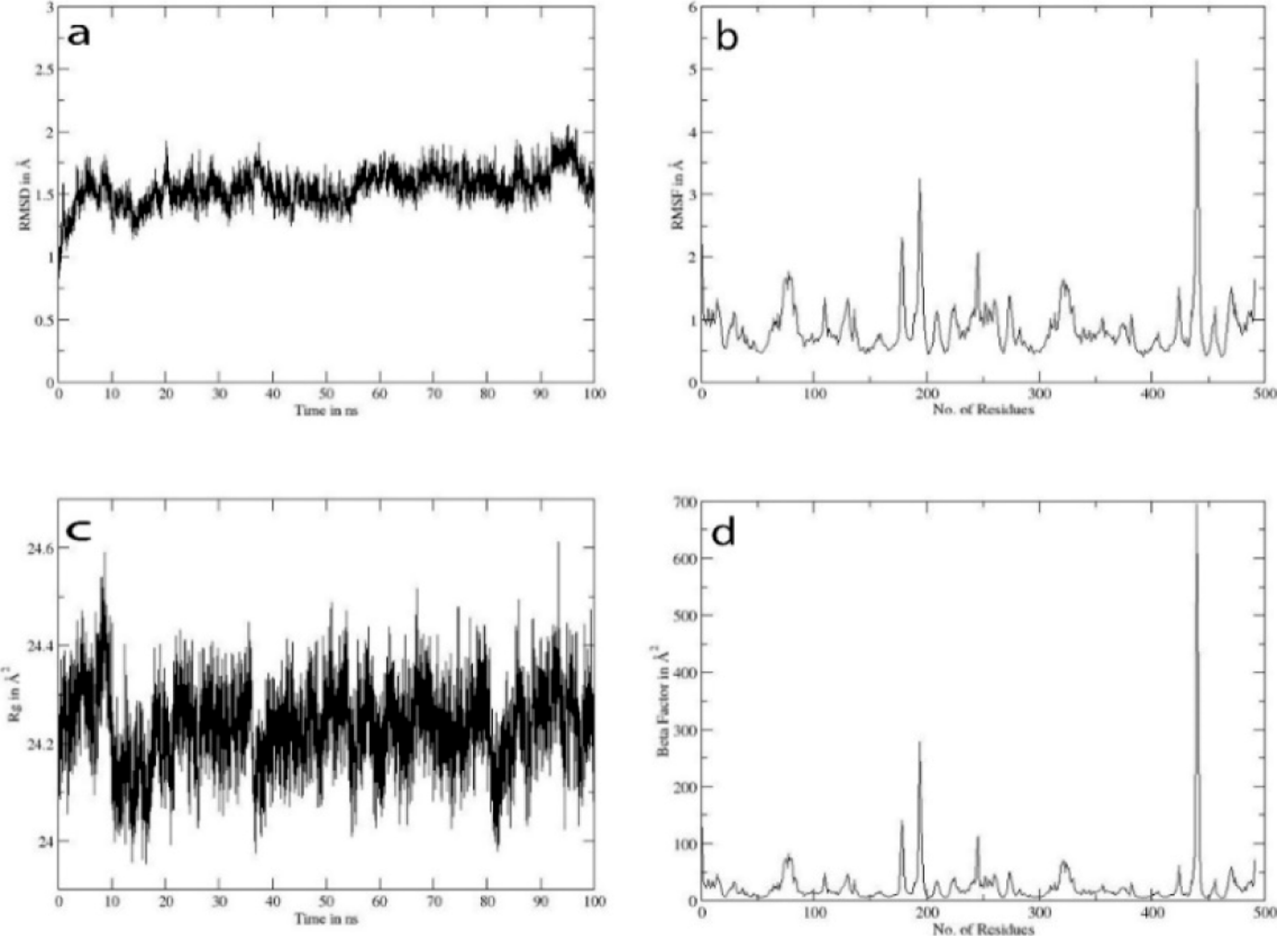
Trajectory analysis for OXA-10-**3g**: (a) shows RMSD in angstroms, (b) shows RMSF in angstroms, (c) shows the radius of gyration, and (d) shows the beta factor.

In both complexes, the elevated RMSF and *b*-factor peaks around the catalytic serine region suggest that **3g** interferes directly with the nucleophilic residue. Normally, this serine performs a nucleophilic attack on the β-lactam ring to hydrolyze the antibiotic; however, the presence of **3g** appears to restrict its mobility and disrupt its proper orientation, thereby impeding catalysis (Figures 8 and 10).

These findings suggest that some compounds exhibit direct antibacterial activity, while others function as β-lactamase inhibitors, ultimately enhancing the efficacy of β-lactam antibiotics and thereby playing a significant role in combating antibiotic resistance. The docking results further reinforce the experimental data, highlighting key interactions responsible for enzyme inhibition and antibacterial activity. The correlation between in vitro assays and computational insights strengthens the validity of these findings, supporting the potential of thienopyrimidine derivatives to address antimicrobial resistance. Further investigations, including in vivo studies and structure–activity relationship (SAR) analyses, will provide deeper insights into their therapeutic applications.

### Comparative SAR Study

A wide array of 4-substituted thienopyrimidine-based compounds were synthesized with various substitutions. From antibacterial assays, it was evident that 4-*O*-substituted products did not show any activity, including long alkyl chain acids. Some compounds with amino, thiol, and aryl groups at the fourth position showed optimum activity. In addition to interactions of the thienopyrimidine core with targets, they also showed π–alkyl or π–π interactions between aromatic substituents and aliphatic side chains of residual amino groups of the protein, as shown in 2D ligand–protein interaction diagrams. Imidazole-substituted thienopyrimidine **3e** showed activity against both bacterial strains. In docking studies, it was found that when it formed a complex with the DDL target in addition to other π–π interactions of the thienopyrimidine core, the imidazole group also showed π–alkyl interactions with residue His137, and in the case of the RMLA target, the imidazole showed the same types of interactions with the side chain of other residue Lec28 (Figure 11a). Similarly, compounds **5d** and **6a** showed the same types of interactions with both targets and were active against both bacterial strains (Figure 11b). In the case of compound **3g**, although there is no aryl group present at the fourth position, in addition to other interactions of the thienopyrimidine molecule, pi–alkyl interactions were shown by the tertiary butyl group present at the second position of the pyrimidine ring with other protein residues. These interactions could be responsible for the stability of the protein ligand complex.

**Figure 11:**
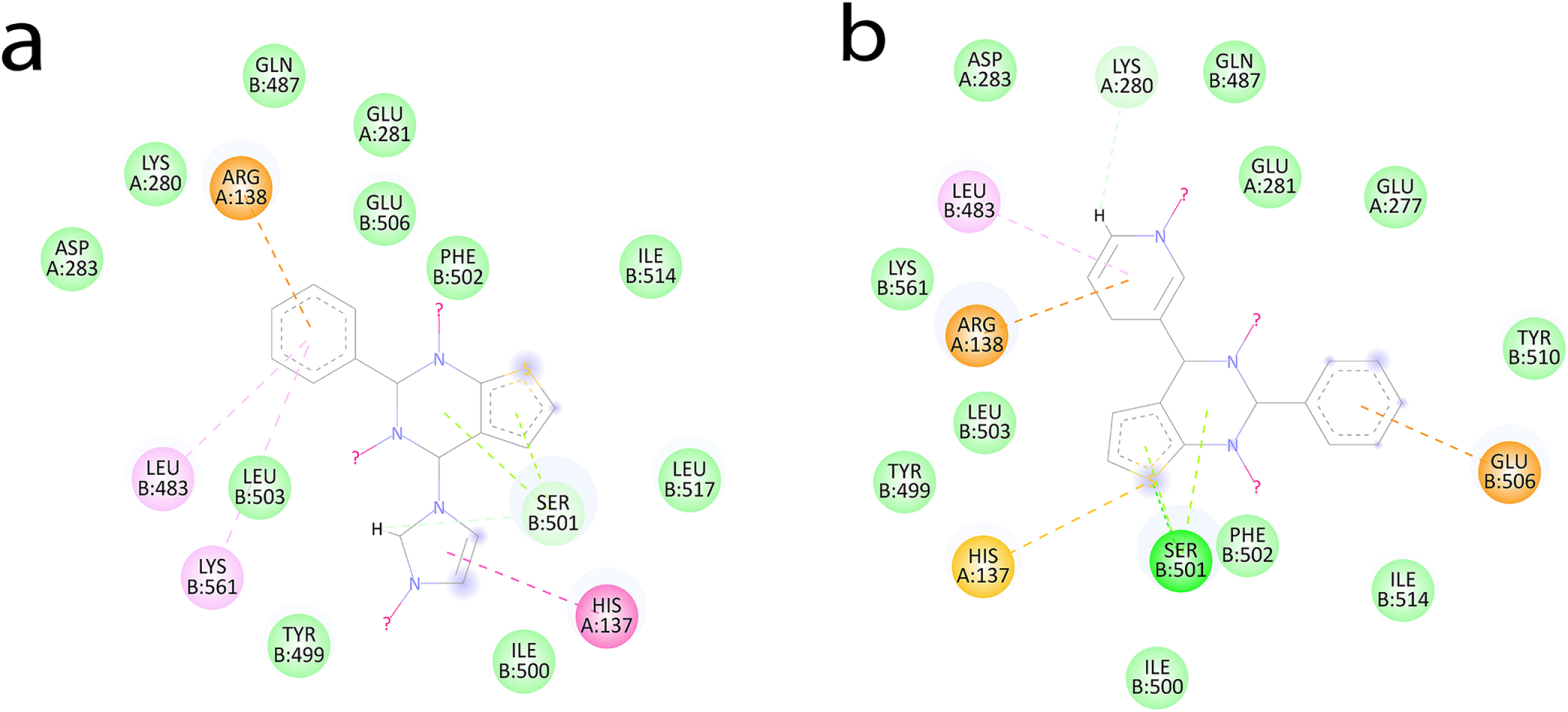
a) Interactions of **3e** with the DDL target and (b) interactions of **5d** with the DDL target.

Of 10 antibacterial compounds, 3 compounds were found to be potent β-lactamase inhibitors. Compound **4a,** having a thiol group at the fourth position, showed interactions between sulfur and hydrogen in the case of both targets, i.e., in the BLAZ complex, between sulfur and the ARG250 residue (Figure 12a), and in OXA-10 with two residues, ARG235 and SER226 (Figure 12b). Similarly, in **3g**, the additional π–alkyl interactions are present between the aliphatic butyl group present at the second position of pyrimidine and MET99 and TRP102 residues of the complex (Figure 12c). In **6a,** the phenyl group present adjacent to acetylene is involved in π–cation interactions by donating electrons to the electron-deficient group of Lys205 (Figure 12d). These interactions show that, in addition to the thienopyrimidine core, the presence of phenylacetylene, thiol, and a tertiary butyl group is also important for the β-lactamase inhibitory activity.

**Figure 12:**
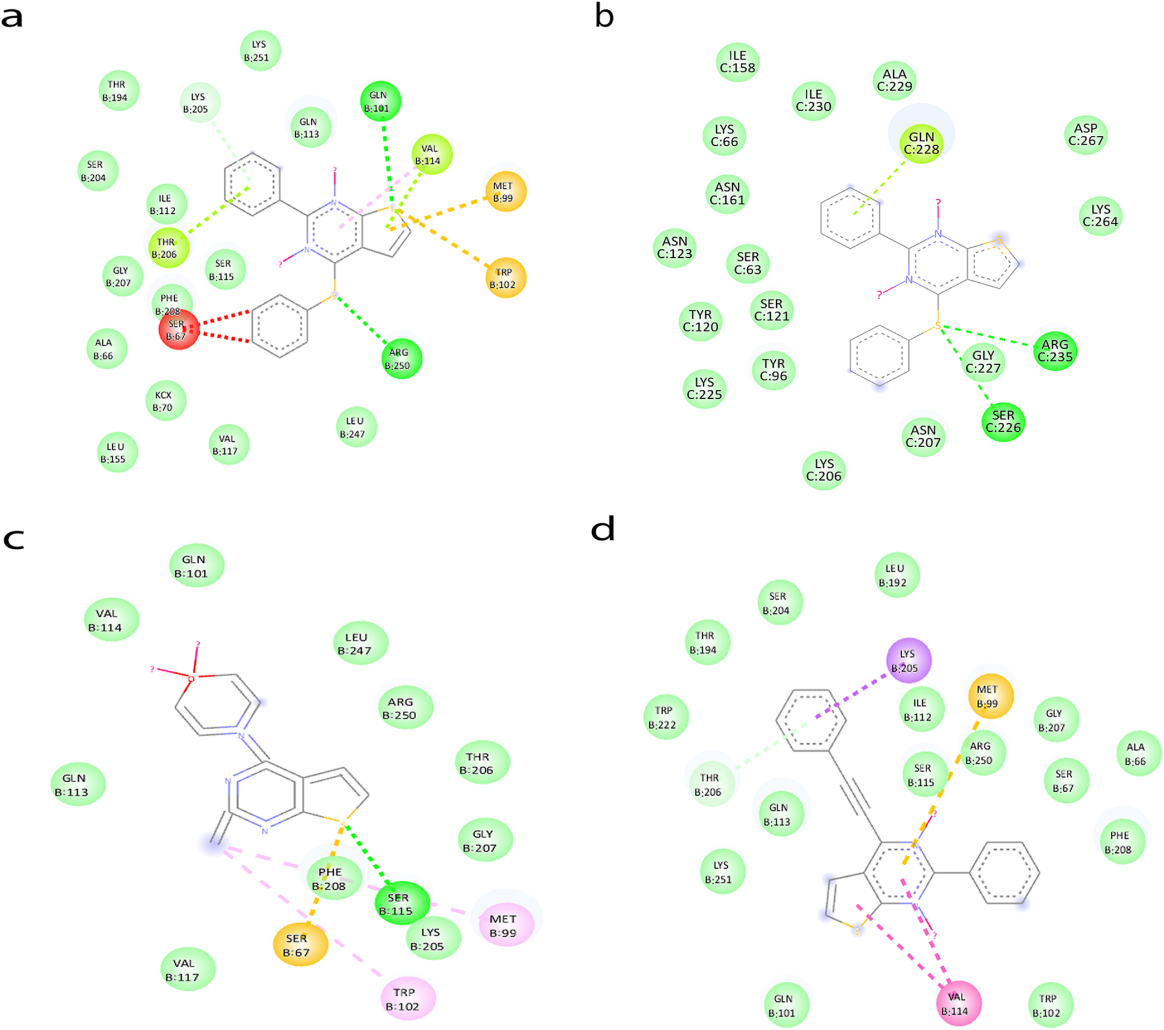
Comparative SAR studies: (a) interactions of **4a** with the BLAZ target, (b) interaction of **4a** with the OXA-10 target, (c) interactions of **3g** with the BLAZ target, and (d) **6a** with the BLAZ target.

### Toxicity Profile

Computational toxicity assessment via PkCSM provided useful early safety insights in the absence of experimental cytotoxicity data. While some thienopyrimidine derivatives displayed potential mutagenic or hepatotoxic alerts, their overall toxicity parameters remained within acceptable limits for antibacterial adjuvants. The predicted toxicity end points of the synthesized thienopyrimidine derivatives (**3c–6a**) and the control cefixime are summarized in Table 6. All compounds were predicted to be noninhibitory to hERG I, indicating low cardiotoxic risk, while a few showed mild hERG II inhibition potential. None of the compounds were predicted to cause skin sensitization. AMES mutagenicity results varied, with compounds **3d**, **3g**, **4a**, **5b**, and **6a** identified as nonmutagenic. The predicted maximum tolerated doses ranged between 0.208 and 0.786 (log mg/kg/day), generally lower than that of cefixime (1.296), suggesting moderate systemic safety. LD_50_ values ranged from 1.893 to 2.634 mol/kg, indicating comparable or slightly lower acute toxicity than the control. Chronic toxicity (LOAEL) values varied between 0.462 and 2.252 (log mg/kg_bw/day). Among these, compound **3g** exhibited a balanced safety profile with no AMES or hERG liability and moderate tolerance parameters. Notably, compound **3g** demonstrated a favorable balance between low predicted acute toxicity, the absence of mutagenicity and cardiotoxicity, and manageable hepatotoxic risk comparable to cefixime. These results suggest that **3g** may be a safer candidate for coadministration with imipenem, although experimental validation through standard in vitro cytotoxicity assays (e.g., HepG2, MTT) and in vivo toxicity studies will be necessary to confirm its safety profile.

**Table 6:** In Silico Toxicity Prediction of Synthesized Compounds and Cefixime

compound	AMES toxicity	max. tolerated dose (human)	hERG I inhibitor	hERG II inhibitor	oral rat acute toxicity (LD50)	oral rat chronic toxicity (LOAEL)	hepatotoxicity	skin sensitization	*T. Pyriformis* toxicity	minnow toxicity
**3c**	Yes	0.697	No	No	2.446	1.011	No	No	0.336	0.115
**3d**	No	0.784	No	Yes	2.634	0.857	Yes	No	0.288	1.369
**3e**	Yes	0.336	No	Yes	2.486	1.044	No	No	0.285	0.045
**3f**	Yes	0.208	No	No	2.521	1.272	No	No	0.826	0.998
**3g**	No	0.259	No	No	2.488	1.423	Yes	No	1.514	0.325
**4a**	No	0.786	No	Yes	1.893	2.213	Yes	No	0.286	–0.6
**4b**	Yes	0.726	No	Yes	2.063	0.462	No	No	0.287	–1.439
**5b**	No	0.747	No	Yes	2.092	2.252	Yes	No	0.285	–1.875
**5d**	Yes	0.46	No	No	2.193	2.039	Yes	No	0.291	–0.137
**6a**	No	0.6	No	Yes	2.27	1.936	Yes	No	0.302	–1.313
**cefixime**	No	1.296	No	No	1.928	1.759	Yes	No	0.284	3.971

## Methodology

### Experimental Analyses

#### Antibacterial Assay

To assess the potential of the compounds for antibacterial activity, an agar disc diffusion assay was performed on Mueller–Hinton Agar (MHA) from Oxoid England. Both the clinical and ATCC strains of *S. aureus* and *P. aeruginosa* were cultured and incubated at 37 °C for 24 h. The stock solution of compounds and positive control cefixime was prepared by adding 1 mL of DMSO to 1 mg of compounds. The cultured bacteria were then inoculated in 1 mL of sterilized normal saline and adjusted to 0.5 McFarland (1% H_2_SO_4_ and 1% BaCl_2_) for turbidity. This was followed by preparing a bacterial lawn with the placement of 6 mm sterilized filter paper discs at a 90° angle. A 5 μL portion of compounds and control was added to the discs, which were then incubated at 37 °C overnight. The zone of inhibition was measured in millimeters and calculated statistically after running three similar experiments. The MIC assays were performed by preparing 2-fold serial dilutions of the stock solution of the compounds in sterilized nutrient broth using a 96-well microplate. After inoculation, microplates were incubated at 37 °C overnight.

#### β-Lactamase Activity Assay

β-Lactamase inhibition was evaluated using the chromogenic cephalosporin substrate nitrocefin, which yields a colored product upon hydrolysis detectable at 490 nm. Assays were performed following the Abcam β-lactamase kit protocol (ab197003). A standard curve was established, and the reaction mix and nitrocefin control solution were prepared as instructed. A stock solution (0.5 g in 100 μL) was prepared and converted to molar concentrations for subsequent assays. The effect of DMSO on β-lactamase activity was evaluated as a negative control, and no inhibition was observed under the assay conditions. Enzyme activity was monitored as the rate of nitrocefin hydrolysis was monitored at 490 nm. IC_50_ values were obtained by plotting residual enzyme activity against the logarithm of inhibitor concentration and fitting the resulting dose–response curves.

#### Synergistic Assay

To check the antagonistic effect of compounds, a synergistic assay was performed to find the inhibitory activity of compound **3g** for the ATCC strain of *S. aureus*. A bacterial lawn was prepared using the same protocol as that for the antibacterial assay. The concentration of inhibitory molecules with the cefixime drug is achieved with 1:1 μg/μL along with a positive control of cefixime only in a 1 μg/μL concentration. The tested strains were incubated for 24 h at 37 °C. The resulting inhibitory activity was measured in mm.

### Computational Analyses

#### Toxicity Profile

The toxicity profiles of the synthesized thienopyrimidine derivatives and the reference drug cefixime were evaluated using the PkCSM web server,^26^ which predicts pharmacokinetic and toxicity end points based on graph-based signatures. This algorithm represents each molecule as a distance-based graph of atomic interactions and applies machine-learning models trained on large experimental data sets to estimate parameters such as mutagenicity (AMES), cardiotoxicity (hERG inhibition), hepatotoxicity, maximum tolerated dose, and acute/chronic toxicity in rodents. The predictions provide an initial indication of systemic safety and cytotoxic potential prior to in vitro validation.

#### Molecular Docking and Simulation

Molecular docking provides computational insight into the interactions between a protein and a ligand. To further assess the mechanism of action of the active compounds, druggable targets were selected from *S. aureus* and *P. aeruginosa*. *d* -Alanine– *d* -alanine ligase (DDl) from *S. aureus* was selected, and for *P. aeruginosa*, glucose-1-phosphate thymidylyltransferase (RMLA) was selected as a drug target. To assess interactions against β-lactamase, BlaZ was selected as the target from *S. aureus*, and OXA-10 was selected as the target for *P. aeruginosa*. Docking was performed into the active center using Genetic Optimization for Ligand Docking (GOLD),^27^ and interactions were visualized using BIOVIA Discovery Studio Visualizer^28^ and UCSF Chimera.^29^

The selected conformers were used in MD simulations carried out through the Sander module of AMBER.^30^ Molecular dynamics simulations were carried out following sequential steps of energy minimization (applied to the complete system, water molecules, and heavy atoms), heating to 300 K over 20 ps, equilibration for 100 ps with a 2 fs time step, pressure equilibration for 50 ps, and a production run of 100 ns. The inhibitor library was generated using the Antechamber module. The general amber force field (GAFF)^31^ and the ff03.r1 ^32^ were used to obtain parameters for inhibitors and the enzyme, respectively.

Each complex was solvated in a TIP3P water box with a 12 Å buffer between the solute and the box boundaries. Counterions (Na^+^) were added at random positions to neutralize the systems. For temperature and pressure control, Langevin dynamics^33^ was used, whereas the SHAKE algorithm^34^ was applied to correct bond lengths. The production run was carried out under the constant volume and temperature (NVT) ensemble using a Berendsen thermostat. Trajectory coordinates were saved at 1 ns intervals and subsequently analyzed using Visual Molecular Dynamics (VMD).^35^

## Conclusion

Thienopyrimidines synthesized as part of this study exhibited promising antibacterial activity and potential β-lactamase inhibitory effects. Among these, compound **3g** exhibited the more pronounced β-lactamase inhibition along with synergistic activity when combined with selected β-lactam antibiotics. Molecular docking studies also provided valuable insights into the binding interactions of these compounds with bacterial enzymes, supporting their proposed mechanism of inhibition. A good correlation between experimental observations and computational predictions was observed, which further strengthens the reliability of these findings. Overall, compound **3g** emerges as a promising candidate for future exploration as a β-lactamase inhibitor. However, additional biochemical and in vivo studies are required to confirm their inhibitory potential and therapeutic applicability. The findings thus highlight thienopyrimidines as promising scaffolds for the development of new β-lactamase inhibitors.

## Supplementary Material



## Notes

Analytical data for the synthesized derivatives, including ^1^H and ^13^C NMR, GC–MS, and FTIR spectra (PDF)
