# Supplementary material for: Design, Synthesis, and Evaluation of β‑Lactamase Inhibitors as Potential Therapeutics for Antimicrobial Resistance
Source: ACS Omega. 2025 Dec 1;10(49):61032–47. doi: 10.1021/acsomega.5c10798 (PMC12713499; doi:10.1021/acsomega.5c10798)
Supplement: Supplementary file 1 [file ao5c10798_si_001.pdf]

Supporting Information

Design, Synthesis and Evaluation of β-Lactamase Inhibitors as Potential Therapeutics for Antimicrobial Resistance

Sania Batool[a1], Rabia Farid[b1], Syed Sikander Azam[b*], Abbas Hassan[a,c*]

[a] *Department of Chemistry, Quaid-i-Azam University, Islamabad. 45320, Islamabad, Pakistan.*

[b] *Computational Biology Lab, National Center for Bioinformatics, Quaid-i-Azam University, Islamabad. 45320, Islamabad, Pakistan.*

[c] *Department of Chemistry, College of Science, United Arab Emirates University, Al Ain 1551, Abu Dhabi, United Arab Emirates*

[1] *Both authors contributed equally.*

*Corresponding authors: syedazam2008@gmail.com and abbashassan@uaeu.ac.ae

Contents

1.	General Considerations	S-3
2.	Procedures for the synthesis of the substrate	S-3
3.	Synthesis of 2-aminothiophen-3-carboxamide	S-3
4.	Synthesis of 2-substituted amidothiophen-3-carboxamides	S-4
5.	Synthesis of 2-substituted thienopyrimidinones	S-4
6.	Synthesis of 4-chloro-2-substituted thienopyrimidines	S-4
7.	General procedure for S_NAr reaction	S-5
8.	General procedure for thiolation reaction	S-5
9.	General procedure for the Suzuki reaction	S-6
10.	General procedure for Sonogashira reaction	S-6
11.	General procedure for alkylation of 2-substituted thienopyrimidinones	S-7
12.	General procedure for the hydrolysis of ester-containing derivatives	S-7
13.	Analytical data of substrate molecules	S-7
14.	Analytical data of final products	S-33
15.	References	S-140

General Considerations

The reagents and solvents used for reactions were of analytical grade. Thin layer chromatography was used to monitor the reaction progress, using Merck silica gel-60 F_{254} 0.2 mm pre-coated aluminum plates. U.V lamp at 254 nm wavelength was used to visualize the U.V active compounds on TLC plates. To analyze the U.V inactive compounds, various staining agents were used such as potassium permanganate, anisaldehyde and ninhydrin. Melting points of all the prepared compounds were measured using Gallenkamp melting point apparatus and are uncorrected. IR spectra were recorded as KBr pellets on Schimadzu Fourier transform model 270 IR spectrophotometer. Absorption frequencies for different functional groups were expressed in wave number (cm^{-1}). ^{1}H NMR and ^{13}C NMR spectra were recorded at 300 MHz and 75 MHz respectively on Bruker Avance 300 MHz spectrophotometer. Chemical shifts were reported in parts per million (ppm) relative to TMS as internal reference and coupling constants were calculated in Hertz (Hz) units. EI mass spectra were recorded on GC-MS after dissolving compounds in acetone, hexane, and chloroform.

Procedures for the Synthesis of Substrate

i. Synthesis of 2-aminothiophen-3-carboxamide

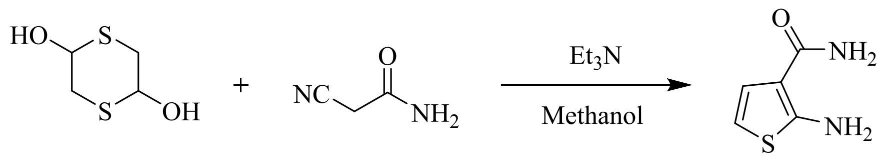

Dithane-2,5-diol (2g, 13.1 mmol, 100 mol%) was added to solution of cyanoacetamide (2.2g, 26.2 mmol, 200 mol%) in methanol (26 mL, 0.5 M) containing triethylamine (182 µL, 1.31 mmol, 10 mol%). The reaction mixture was refluxed for 2 h (TLC checked, $CHCl_3$:MeOH, 9:1). The resulting mixture was cooled to room temperature and excess solvent was evaporated in *vacuo*. The obtained solid was filtered through silica using $CHCl_3$: MeOH (9:1) as eluent to obtain product.[1]

ii. Synthesis of 2-substituted amidothiophen-3-carboxamides.

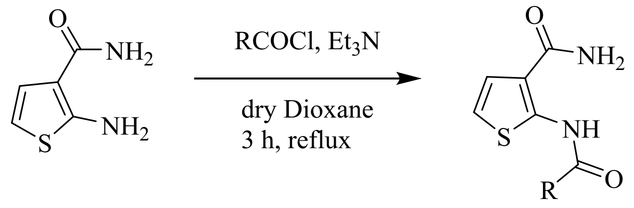

A mixture of 2-aminothiophen-3-carboxamide (100 mol%) and an aliphatic or aromatic acid chloride (200 mol%) in dry dioxane (0.5 M) was refluxed for 3 h in the presence of catalytic amount of triethylamine (TLC checked, $CHCl_3$:MeOH, 0.5:9.5). The excess of solvent was distilled off under reduced pressure. The resulting mixture was extracted with ethyl acetate and purified by flash chromatography (30-40% ethyl acetate in hexane) to acquire the product.[2]

iii. Synthesis of 2-substituted thienopyrimidinones.

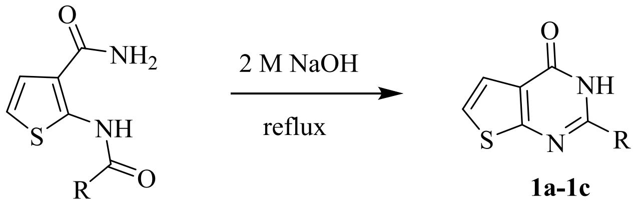

1a-1c

2-Substituted amidothiophen-3-carboxamide (1 mmol) was added to 2 M aqueous solution of sodium hydroxide (3 mL). The resulting mixture was heated at 100 °C till the completion of reaction. (TLC checked, EtOAc:hexane, 1:1) On cooling to room temperature, reaction mixture was neutralized with 6 M aqueous solution of hydrochloric acid (6 mL). As a result, desired product was precipitated out and filtration was used to collect the product.[3]

iv. Synthesis of 4-Chloro-2-substitutedthienopyrimidines

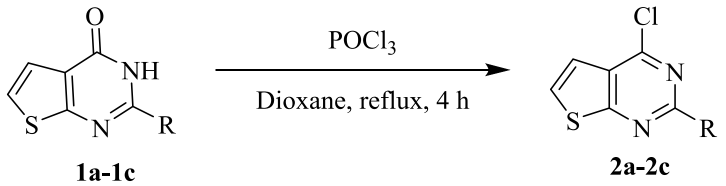

1a-1c **2a-2c**

To a solution of 2-substituted thienopyrimidinone (1 mmol) in dry dioxane (5 mL), phosphorus oxychloride (3 mL) was added. The mixture was refluxed under nitrogen atmosphere till the completion of reaction. (TLC checked, EtOAc : hexane, 1:9) The resulting black solution was

allowed to stand at room temperature. Then, it was poured into saturated aqueous solution of sodium bicarbonate to neutralize the reaction mixture. The aqueous mixture was extracted with ethyl acetate. The combined organic layers were washed with brine solution, and the excess solvent was evaporated under reduced pressure to obtain solid product.[4]

General procedure for S_NAr reaction:

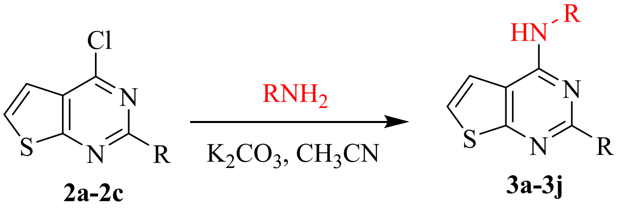

4-Chloro-2-substitutedthienopyrimidine (100 mol%) and an appropriate secondary amine (200 mol%) were refluxed in acetonitrile in the presence of anhydrous potassium carbonate (200 mol%) (TLC checked, 20-50% ethyl acetate in hexane). The reaction mixture was cooled to room temperature and excess acetonitrile was distilled off in *vacuo*. The resulting mixture was extracted with ethyl acetate and purified by flash chromatography using ethyl acetate and hexane as eluent.[5]

General procedure for thiolation reaction:

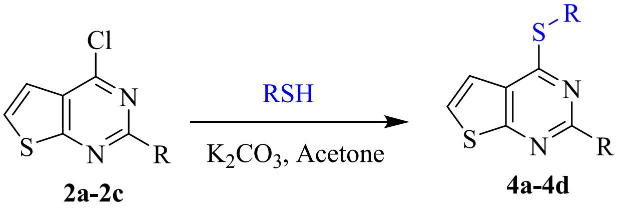

4-Chloro-2-substitutedthienopyrimidine (100 mol%) and aromatic or heteroaromatic thiophenol (200 mol%) were dissolved in dry acetone and anhydrous potassium carbonate (200 mol%) was added to solution. The resulting mixture was refluxed under nitrogen atmosphere till the completion of reaction (TLC, 10-50% ethyl acetate in hexane). On cooling to room temperature, excess solvent was evaporated under reduced pressure. Solvent extraction with ethyl acetate was performed followed by flash chromatography to afford the desired product.[6]

General procedure for Suzuki reaction:

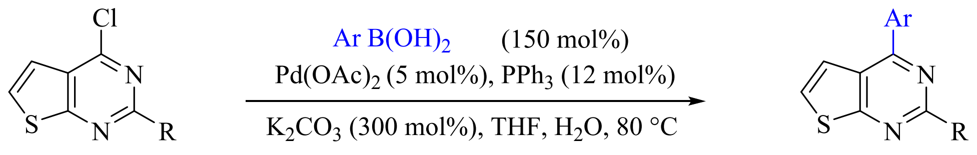

2a-2c **5a-5g**

To an oven dried 100×13 mm sealed tube equipped with magnetic bar, was charged with aryl halide (100 mol%), boronic acid (120 mol%), $Pd(OAc)_2$ (5 mol%), PPh_3 (12 mol%) and potassium carbonate (300 mol%) under inert atmosphere. Binary solvent comprised of analytical grade THF (0.3 M) and H_2O (40 μL), was added, and the reactions were stirred at 80 °C for 24 h. After reaction completion, the mixture was allowed to cool to room temperature and purified by flash column chromatography, using hexane: ethyl acetate (10-30%) as eluent to obtain the desired products.[7]

General procedure for Sonogashira reaction:

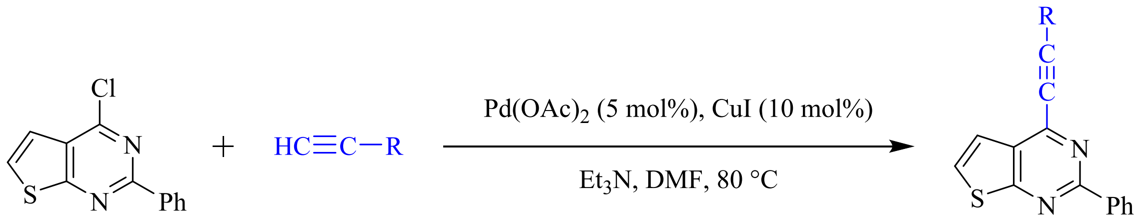

2a-2c **6a-6c**

The suspension of aryl halide (100 mol%), substituted acetylene (200 mol%), CuI (20 mol%), $Pd(OAc)_2$ (5 mol%), triethylamine (200 mol%) in DMF (0.5 M) was heated at 80 °C for 24 h in sealed tube. The sealed tube was purged with nitrogen. The reaction mixture was allowed to stand at ambient temperature and purified by flash column chromatography using hexane to acquire the product.[8]

General procedure for alkylation of 2-substituted thienopyrimidinones.

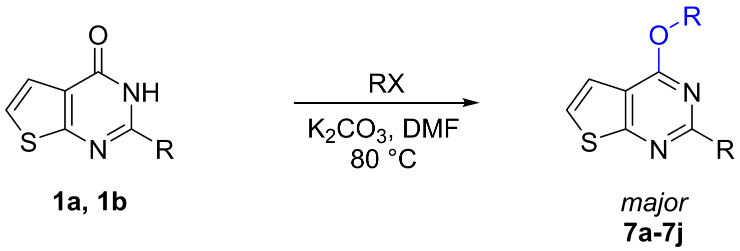

2-Substituted thienopyrimidinone (100 mol%) and alkyl halide (150 mol%) was dissolved in DMF (0.2 M). The reaction mixture was stirred at 80 °C overnight in the presence of potassium carbonate (200 mol%). (TLC checked 10-30% ethyl acetate in hexane) The resulting mixture was extracted with ethyl acetate and purified by flash chromatography using ethyl acetate and hexane (5-10%) as eluent.[9]

General procedure for hydrolysis of ester containing derivatives.

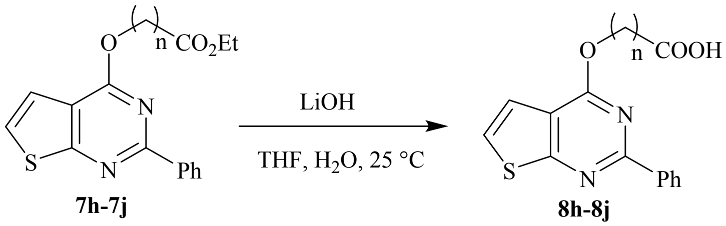

A suspension of 4-*O*-alkylated ester containing thienopyrimidines (100 mol%) and lithium hydroxide (300 mol%) was stirred at room temperature in H_2O: THF (1:1). Upon completion of reaction (TLC checked, $CHCl_3$: MeOH, 9:1), acid base extraction was performed to purify the desired compound. [10]

Analytical Data for intermediates and substrate molecules:

2-Aminothiophen-3-carboxamide

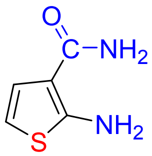

Yield: 75%

mp = 140-143 ˚C (lit.[11] mp = 138-139 ˚C)

IR: (*v,* cm^{-1}) 3443, 3312, 3051, 1661, 1617-1595, 1241-1126, 651

^{1}H NMR (300 MHz, Acetone d_6): δ (ppm) 7.06-7.04 (d, J = 6.0 Hz, 2H), 6.52 (s, 1H), 6.24-6.22 (d, J = 6.0 Hz, 1H)

^{13}C NMR (75 MHz, Acetone d_6): δ (ppm) 168.8, 163.1, 125.2, 108.6, 106.6

GC-MS Analysis (m/z): M^+ = 142, 125, 97

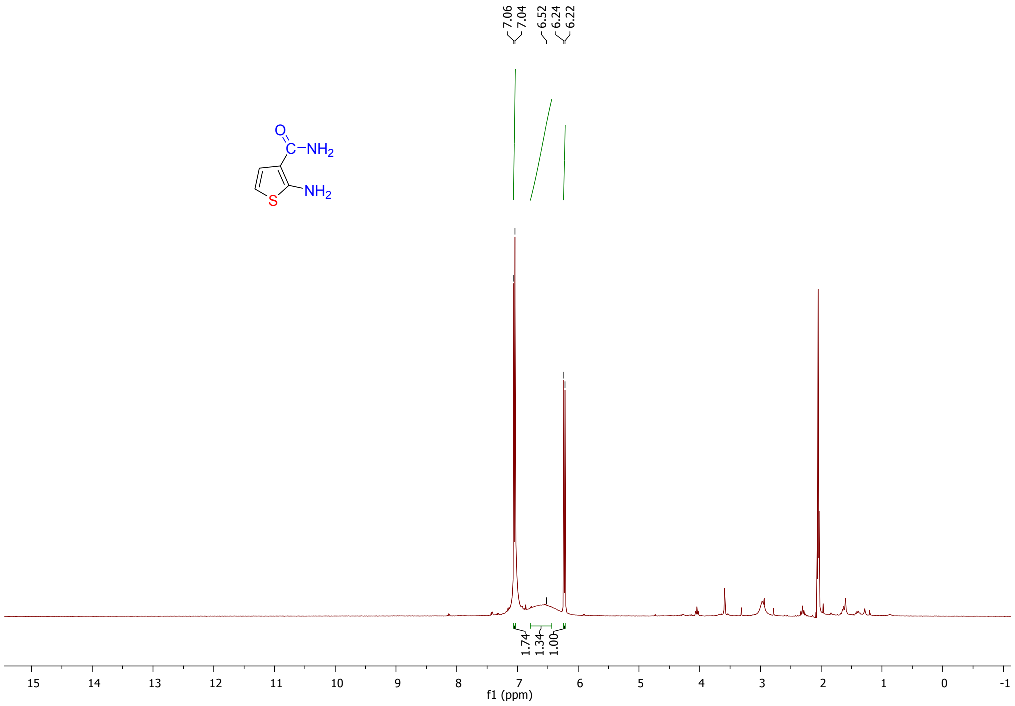

Fig. S-1: ^{1}HNMR Spectrum of 2-Aminothiophen-3-carboxamide

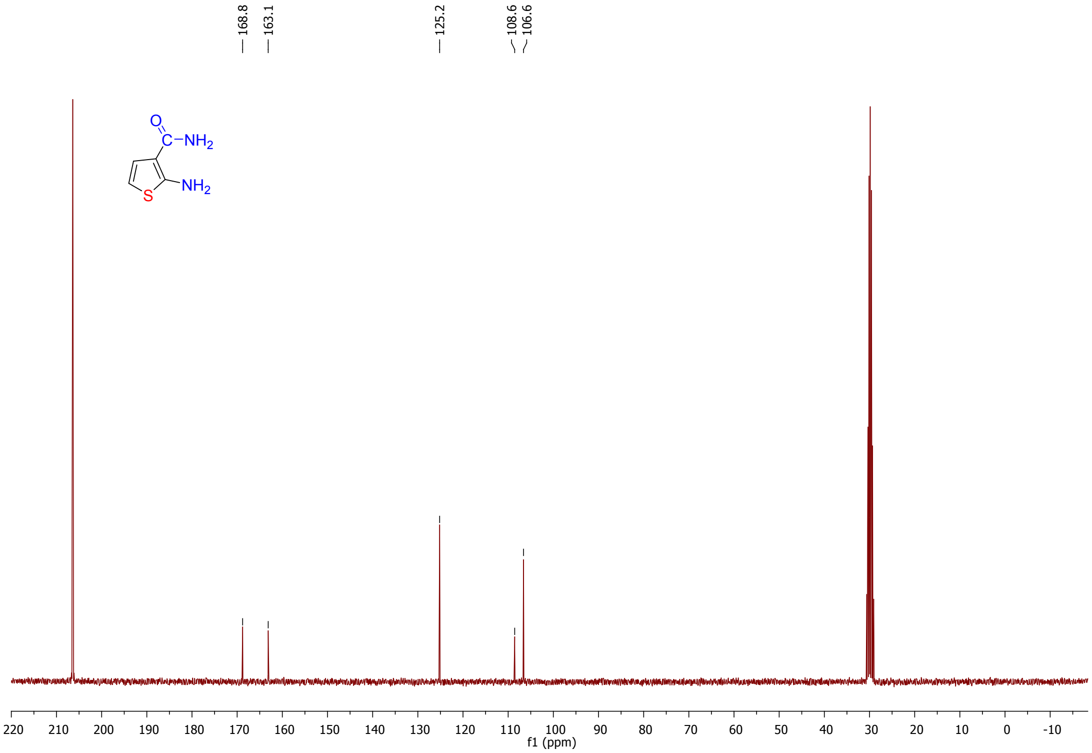

Fig. S-2: ^{13}C NMR Spectrum of 2-Aminothiophen-3-carboxamide

2-Benzamidothiophene-3-carboxamide

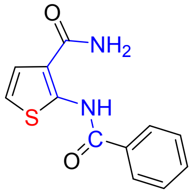

Yield: 80%

mp = 204-206 °C

IR: (*v,* cm^{-1}) 3416, 3321, 3079, 1667, 1595-1447, 774-673.

^{1}H NMR[12] (300 MHz, Acetone d_6): δ (ppm) 13.44 (s, 1H), 8.04-8.01 (m, 2H), 7.67-7.61 (m, 3H), 7.48-7.46 (d, J = 6.0 Hz, 1H), 7.00-6.98 (d, J = 6.0 Hz, 1H)

^{13}C NMR[12] (75 MHz, Acetone d_6): δ (ppm) 168.7, 163.7, 148.3, 133.5, 133.4, 129.9, 128.1, 123.4, 117.2, 116.1

GC-MS Analysis (m/z) = M^+ = 246, 105, 77, 51

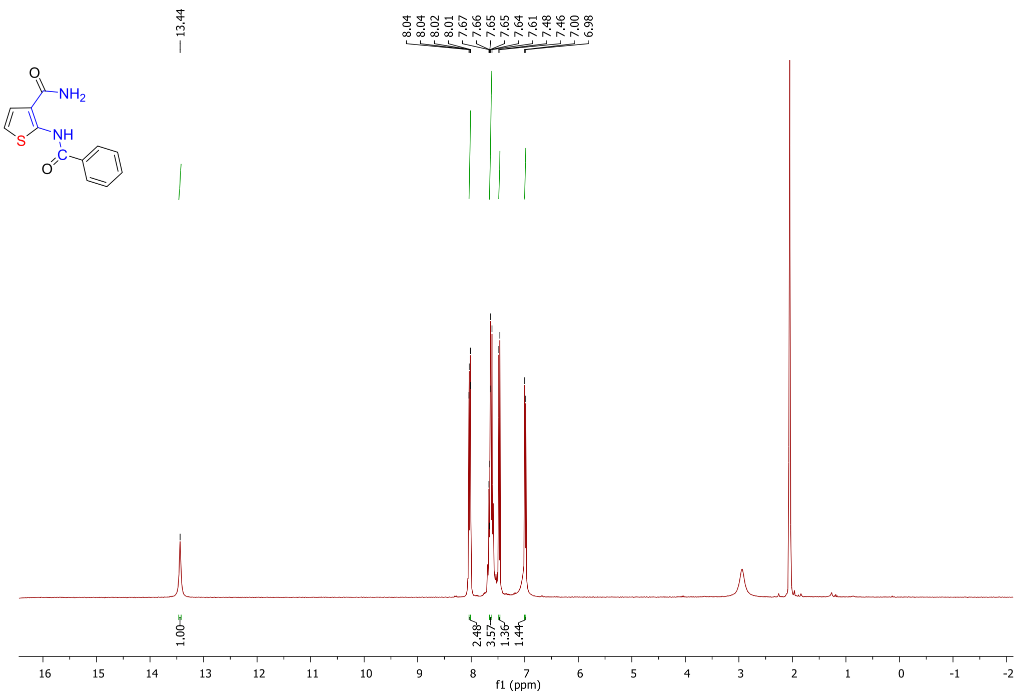

Fig. S-3: ^{1}HNMR Spectrum of 2-Benzamidothiophene-3-carboxamide

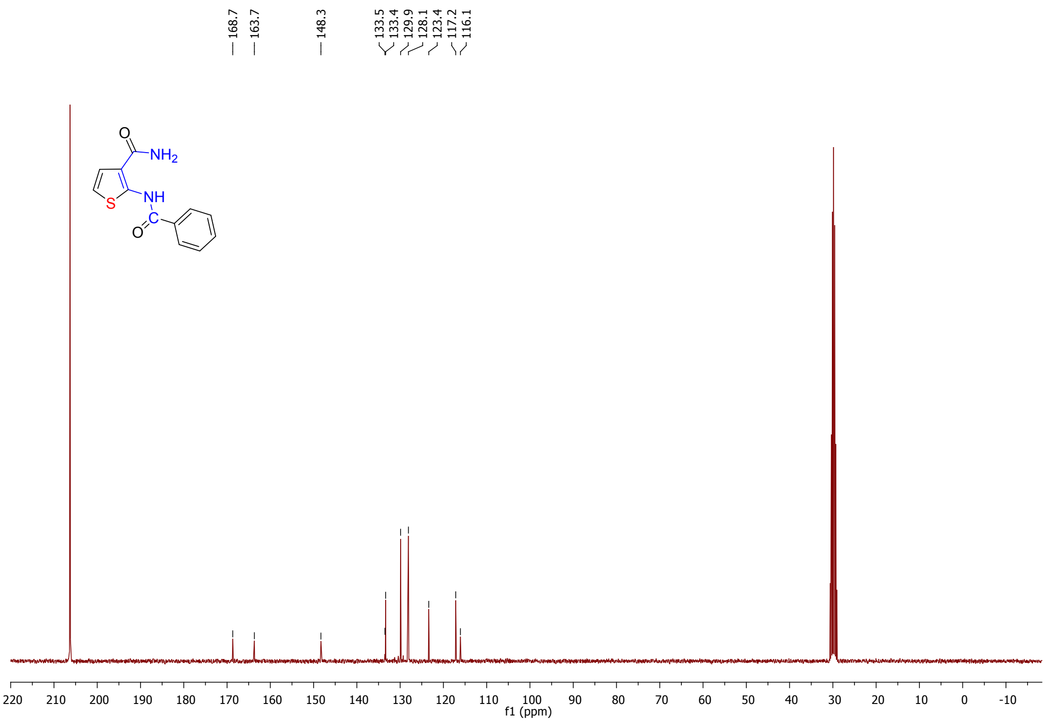

Fig. S-4: ^{13}C NMR Spectrum of 2-Benzamidothiophene-3-carboxamide

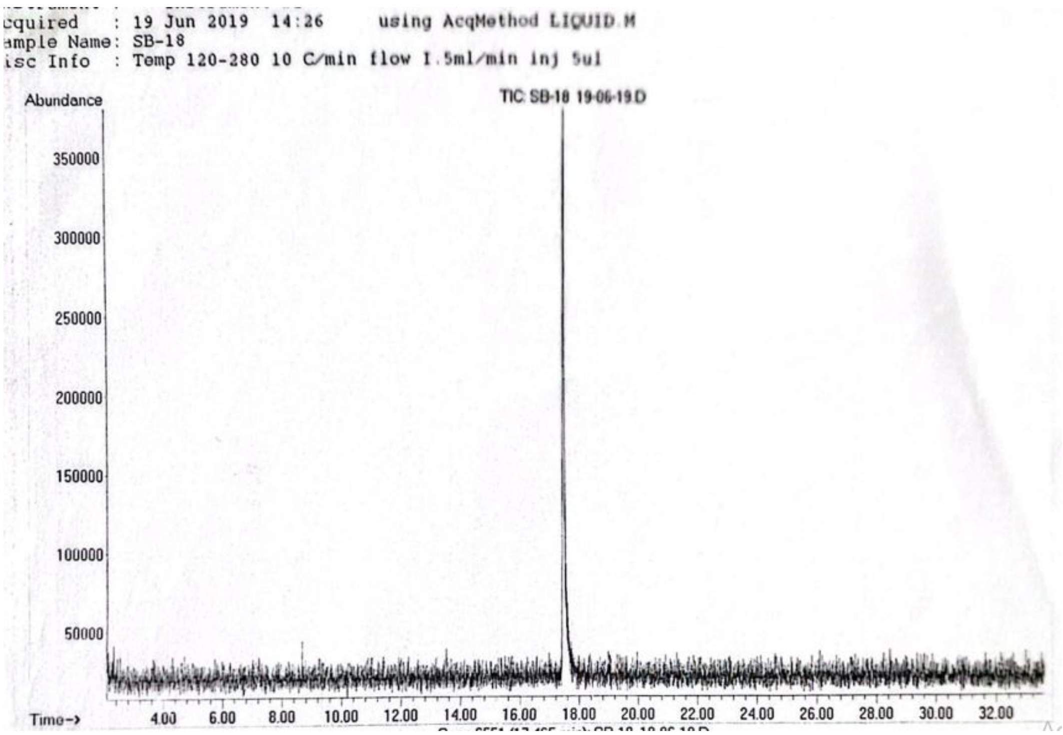

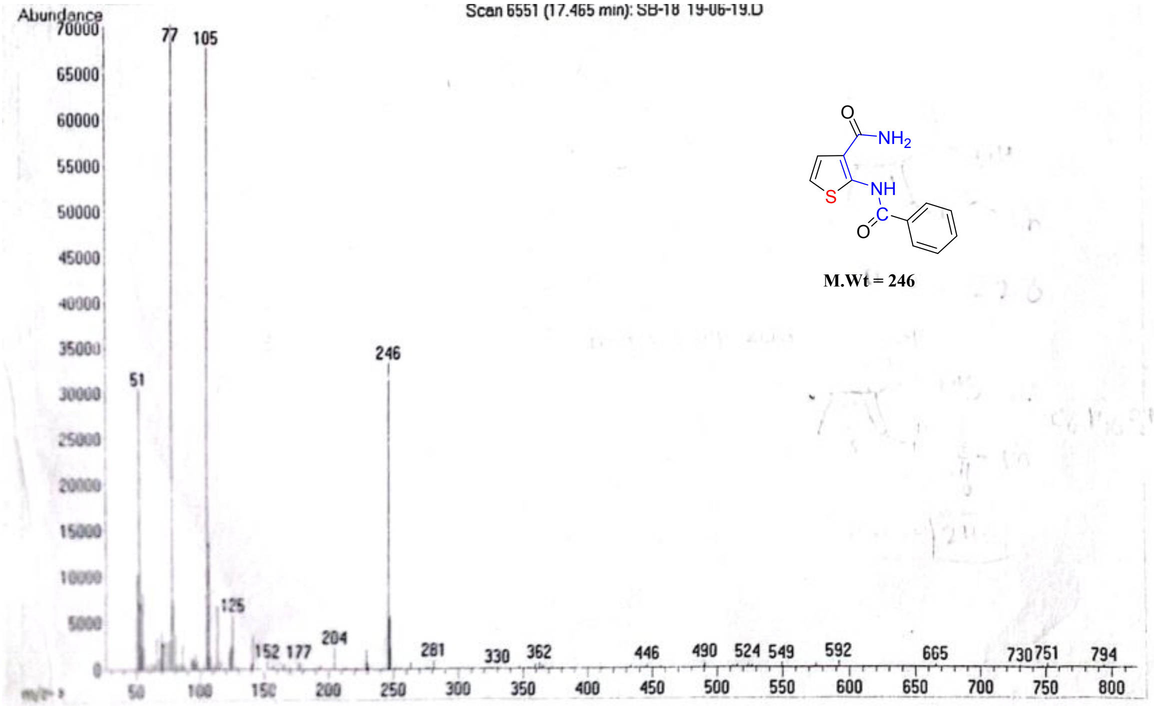

Fig. S-5: GCMS Spectrum of 2-Benzamidothiophene-3-carboxamide

2-Acetamidothiophene-3-carboxamide

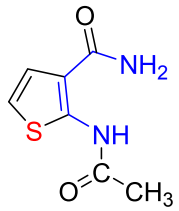

Yield: 52%

mp = 160-162 ˚C (lit.[13] 188-190 ˚C)

IR: (*v,* cm^{-1}) 3416, 3334, 3046, 1661, 1659, 1589-1497, 1278-1051, 676

GC-MS Analysis (m/z) = M^{+} = 184, 125, 97

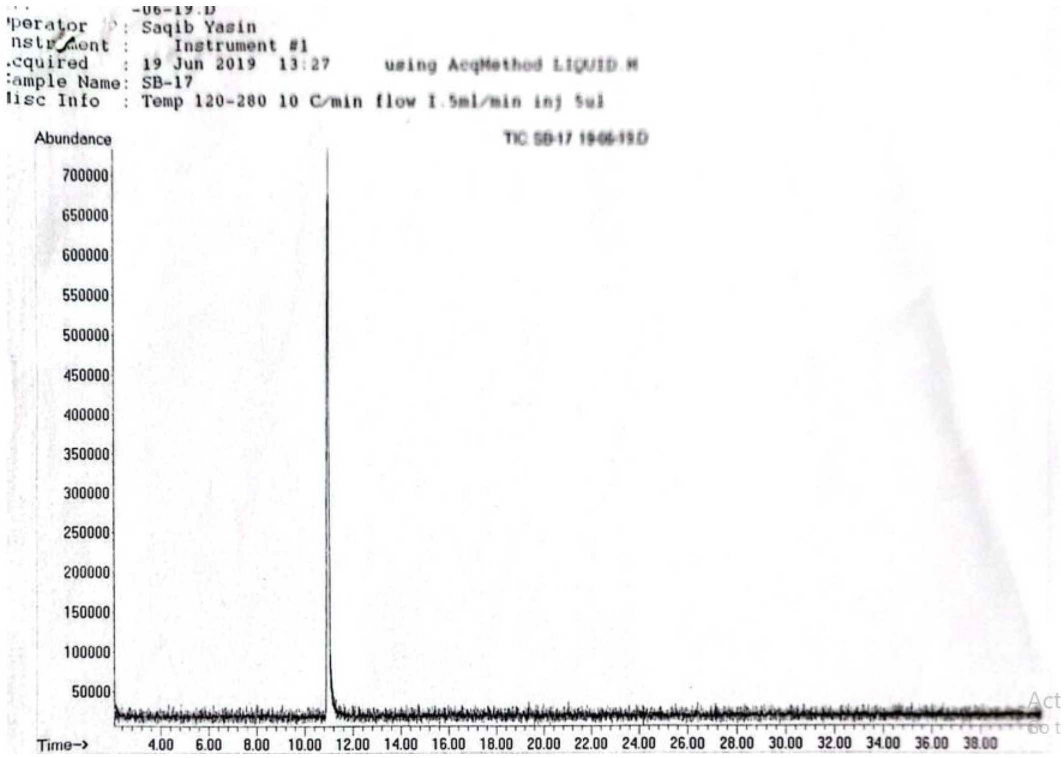

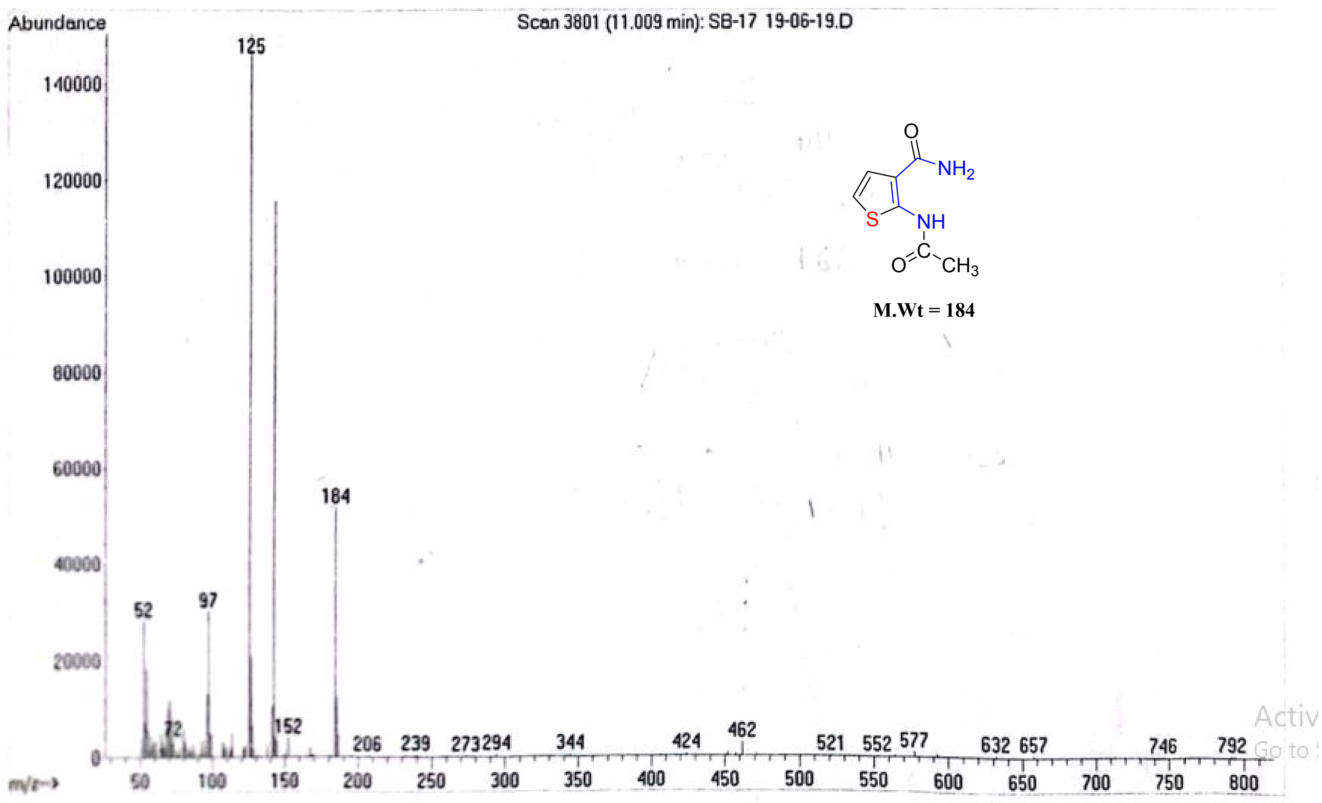

Fig. S-6: GCMS Spectrum of 2-Acetamidothiophene-3-carboxamide

2-Pivalamidothiophene-3-carboxamide [12]

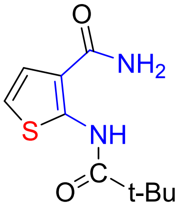

Yield: 55%

mp = 156-159 °C

IR: (*v,* cm^{-1}) 3427, 3376, 3051, 2903, 1671, 1664, 1602-1548, 1309-1196, 667

GC-MS Analysis (m/z) = M^+ = 226, 169, 125, 57

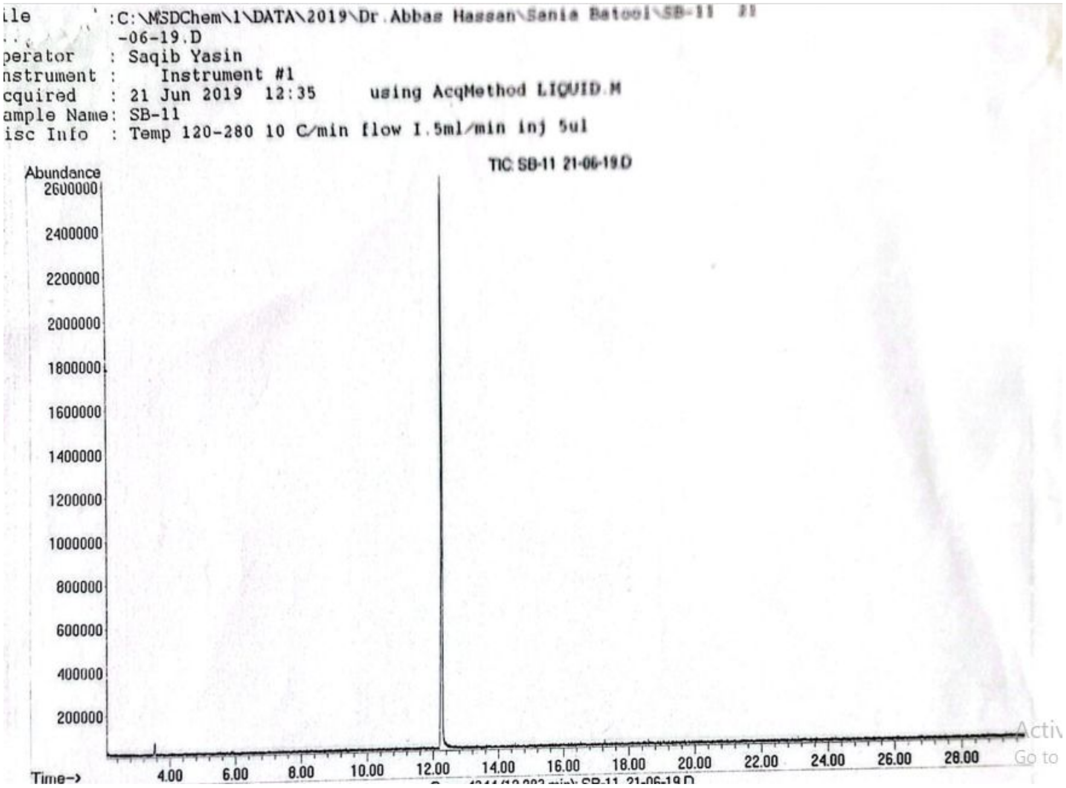

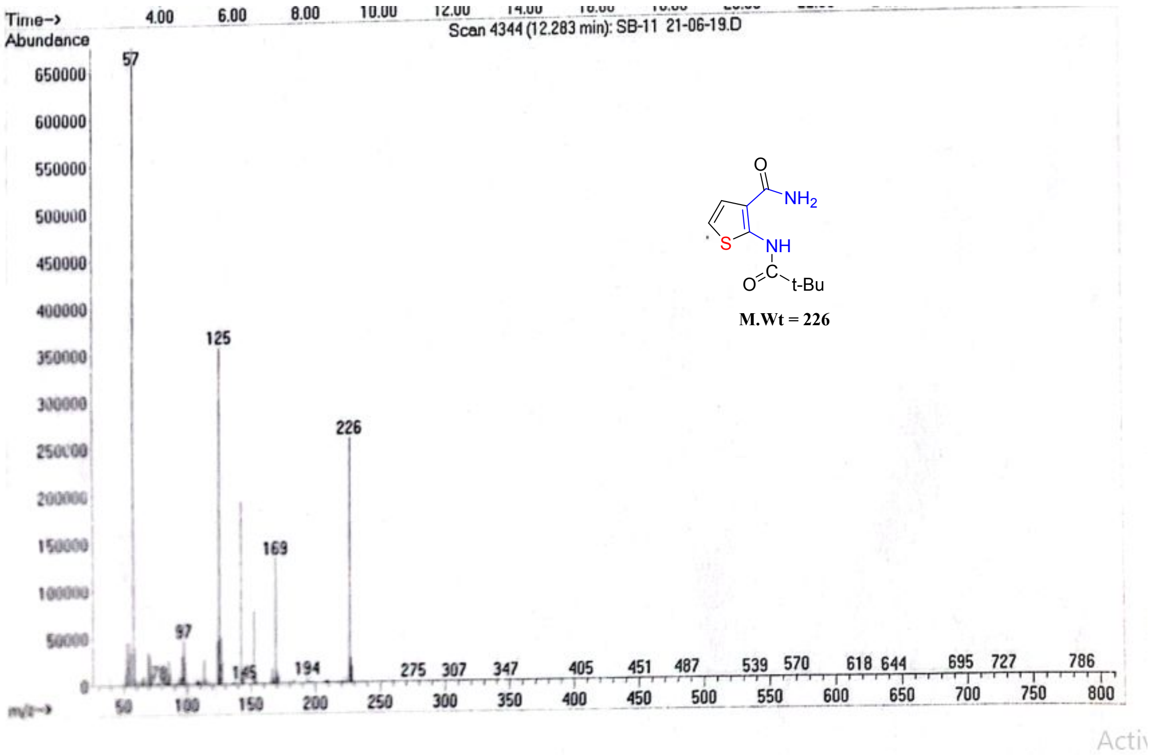

Fig. S-7: GCMS Spectrum of 2-Pivalamidothiophene-3-carboxamide

2-Phenylthieno[2,3-*d*]pyrimidin-4(3*H*)-one (1a)

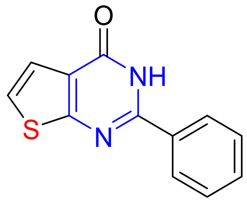

Yield: 87%

mp = 241-244 °C (lit.[14] 238-240 °C)

IR: (*v*, cm^{-1}) 3079, 1660, 1592-1441, 772-670.

^{1}H NMR (300 MHz, DMSO d_6): δ (ppm) 12.73 (s, 1H), 8.16-8.12 (m, 2H), 7.60-7.50 (m, 4H) 7.43 (d, J = 6.0 Hz, 1H)

^{13}C NMR (75 MHz, DMSO d_6): δ (ppm) 164.8, 158.7, 153.0, 132.0, 131.6, 128.7, 127.9, 124.0, 122.8, 121.7

GC-MS Analysis (m/z): M^+ = 228, 151, 125, 104, 77, 51

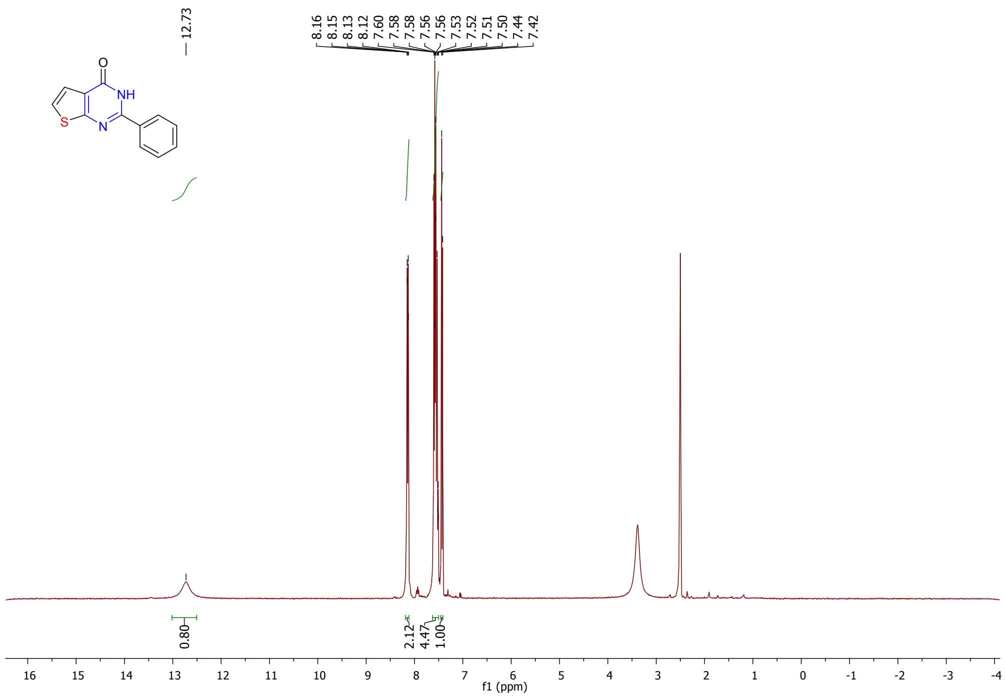

Fig. S-8: ^{1}HNMR Spectrum of **1a**

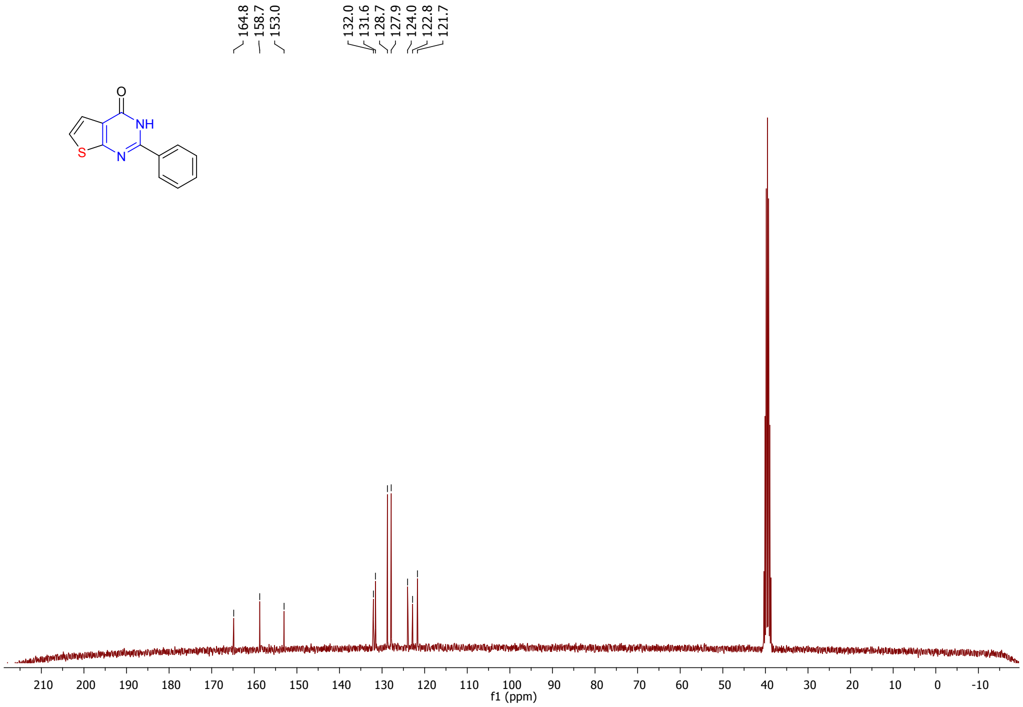

Fig. S-9: ^{13}C NMR Spectrum of **1a**

```
'ile        :C:\MSDChem\1\DATA\2019\Dr.Abbas Hassan\Sania Batool\SB-42 17-
             10-19.D
)perator    : Saqib Yasin
nstrument   :    Instrument #1
.cquired    : 17 Oct 2019  14:14     using AcqMethod LIQUID 50 TO 500.M
:ample Name: SB-42
lisc Info   : Temp 120-280 10 C/min flow 1.5ml/min  Inj 5ul
```

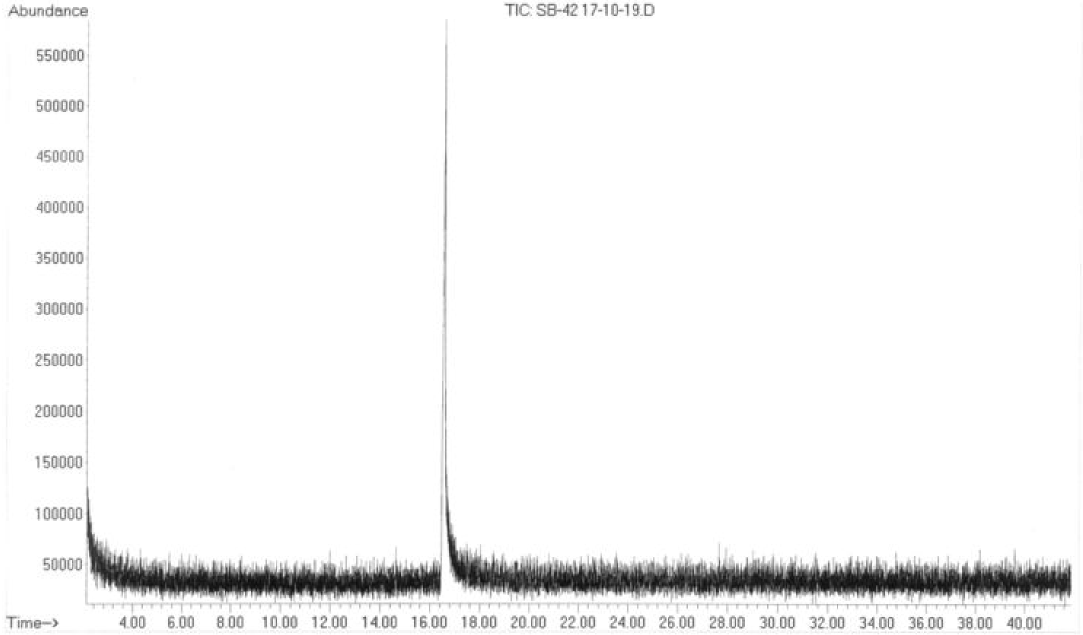

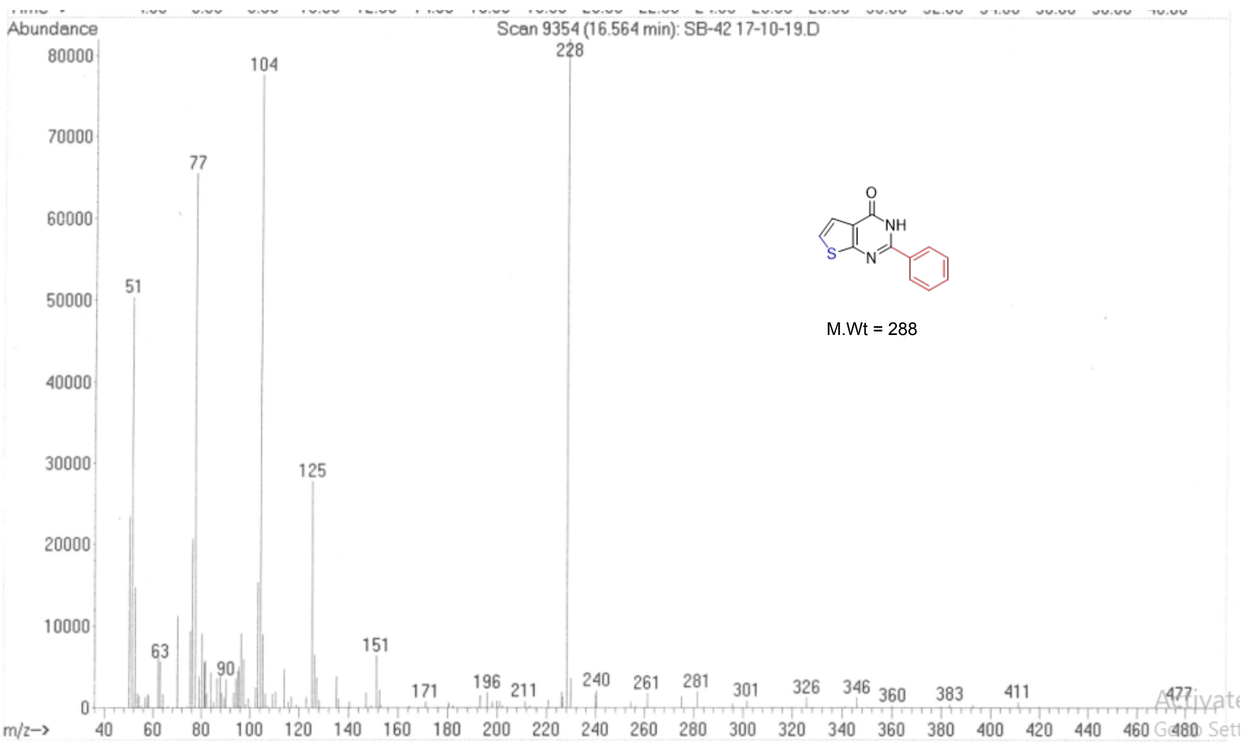

Fig. S-10: GCMS Spectrum of **1a**

2-Methylthieno[2,3-*d*]pyrimidin-4(3*H*)-one (1b)

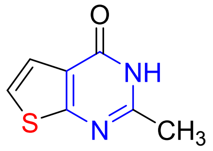

Yield: 86%

mp = 221-224 °C

IR: (*ν*, cm^{-1}) 3076, 2940, 1667, 1597-1465, 1268-1076, 774-658

^{1}H NMR[15] (300 MHz, DMSO d_6): δ (ppm) 12.39 (s, 1H), 7.45 (d, *J* = 6.0 Hz, 1H), 7.32 (d, *J* = 6.0 Hz, 1H), 2.36 (s, 3H)

^{13}C NMR[15] (75 MHz, DMSO d_6): δ (ppm) 164.9, 158.3, 155.1, 122.5, 122.1, 121.4, 21.0

GC-MS Analysis (m/z): M^+ = 166, 125, 97

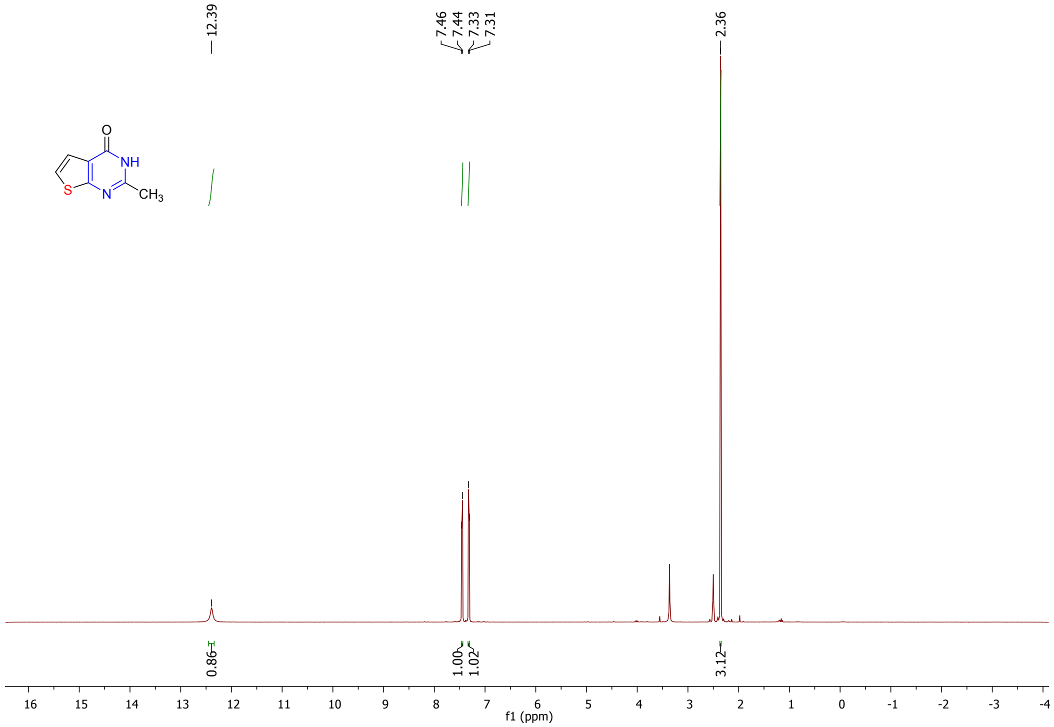

Fig. S-11: ^{1}HNMR Spectrum of **1b**

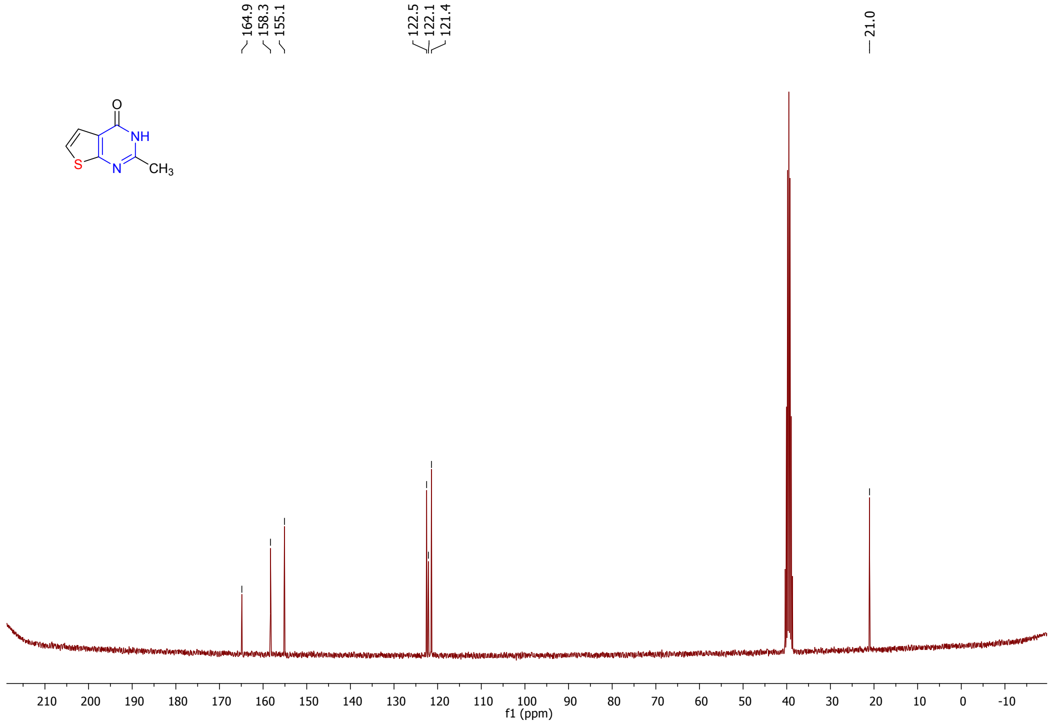

Fig. S-12: ^{13}C NMR Spectrum of **1b**

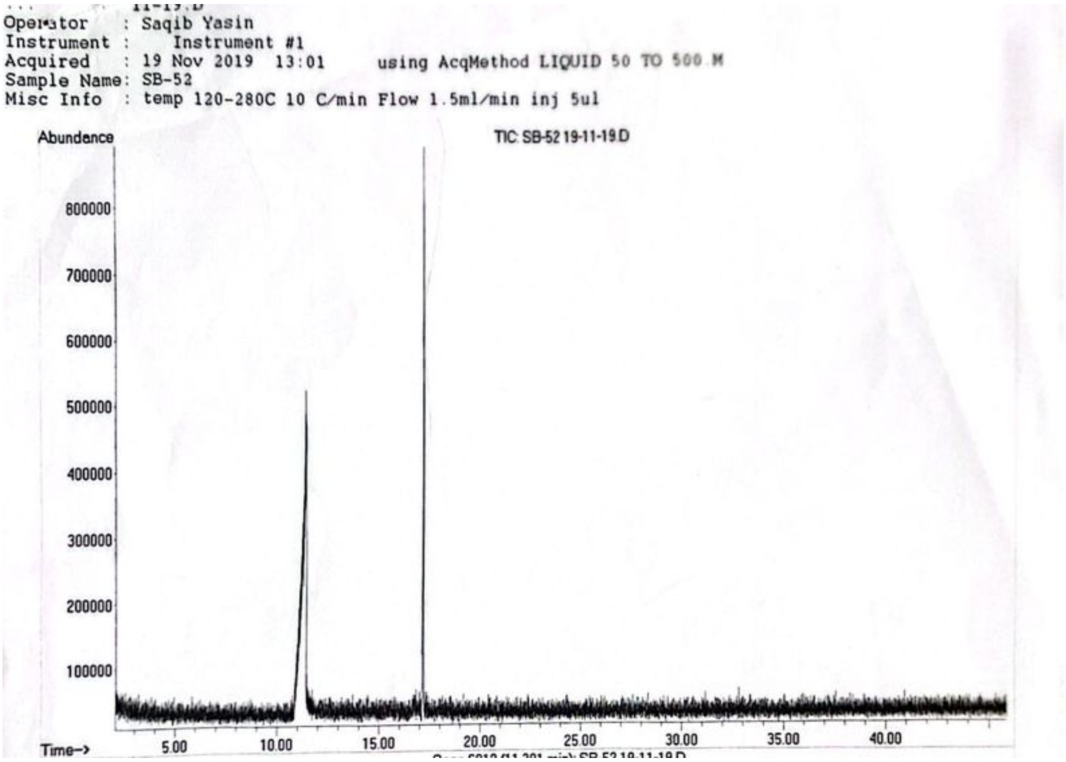

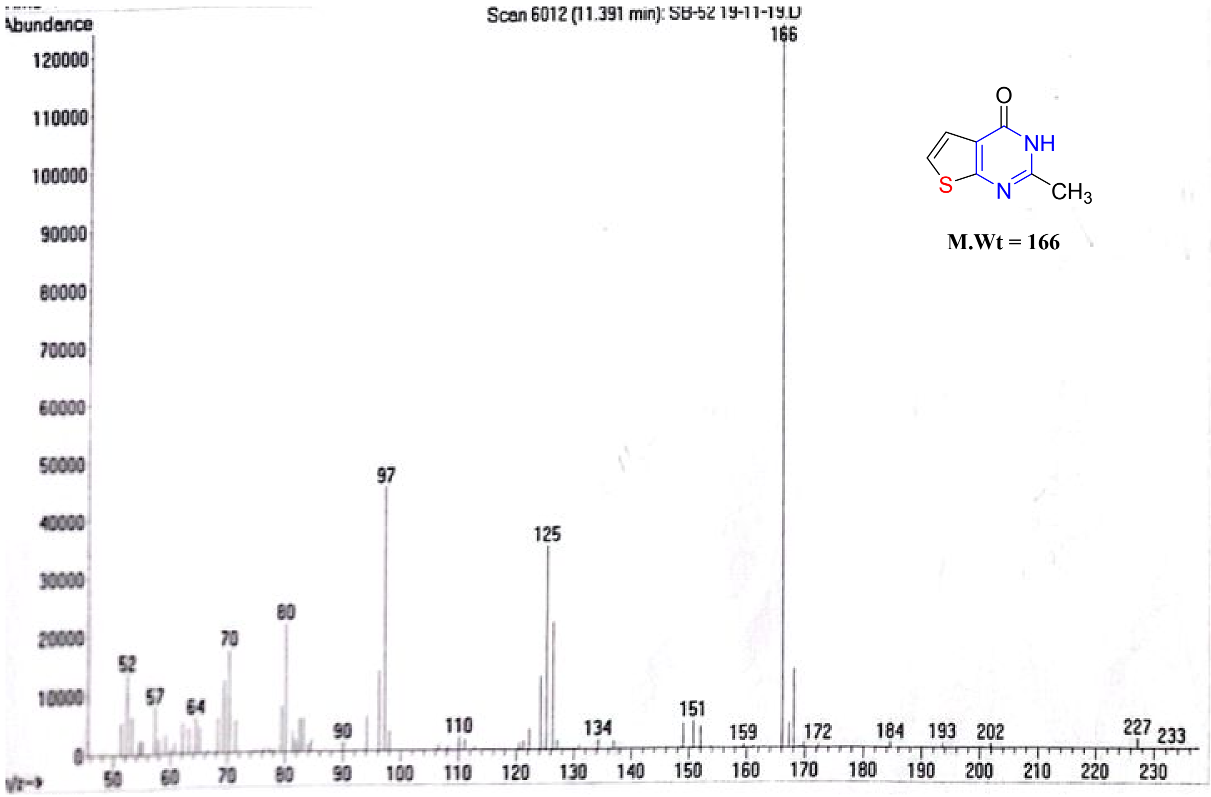

Fig. S-13: GCMS Spectrum of **1b**

2-(*tert*-Butyl)thieno[2,3-*d*]pyrimidin-4(3*H*)-one (1c)

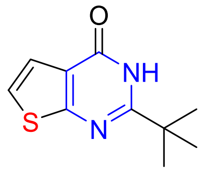

Yield: 82%

mp = 207-211 °C

IR: (*v,* cm^{-1}) 3110, 2867, 1669, 1587-1437, 1311-1051, 771-665.

^{1}H NMR (300 MHz, $CDCl_3$): δ (ppm) 11.43 (s, 1H), 7.46-7.45 (d, *J* = 5.7 Hz, 1H), 7.21-7.19 (d, *J* = 6.0 Hz, 1H) 1.46 (s, 9H)

^{13}C NMR (75 MHz, $CDCl_3$): δ (ppm) 166.2, 163.4, 160.3, 122.7, 122.3, 121.6, 37.7, 28.6

GC-MS Analysis (m/z): M^+ = 208, 193, 166, 125

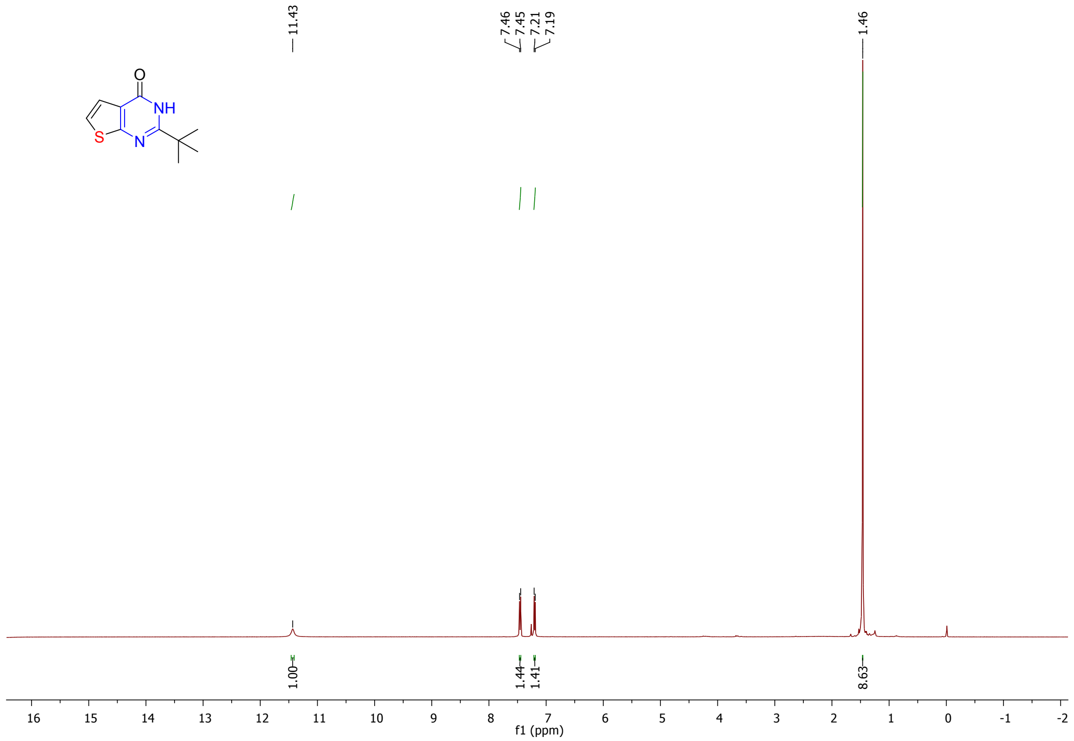

Fig. S-14: ¹HNMR Spectrum of **1c**

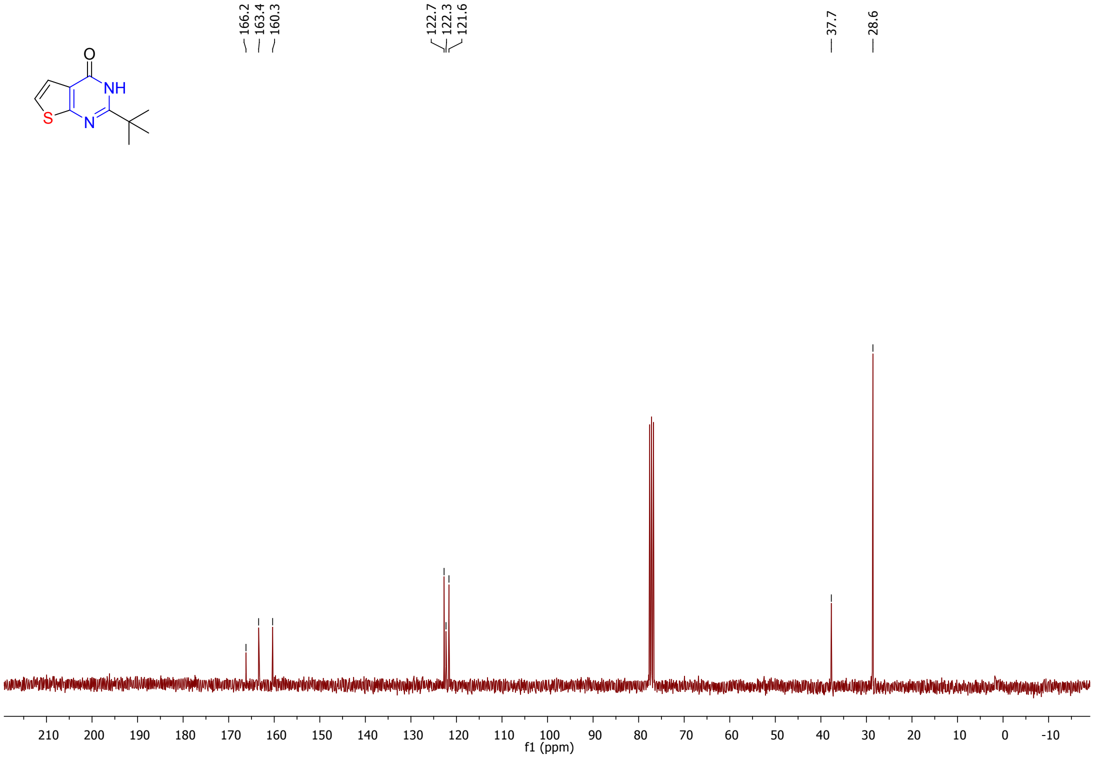

Fig. S-15: ¹³C NMR Spectrum of **1c**

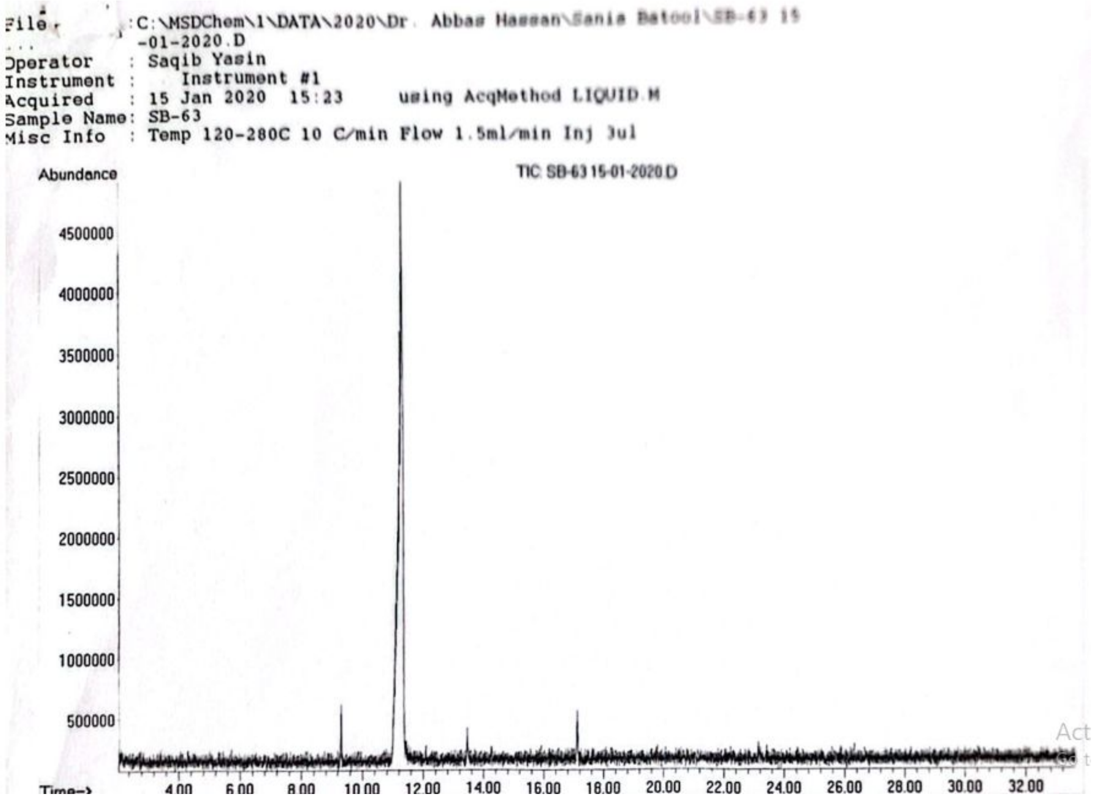

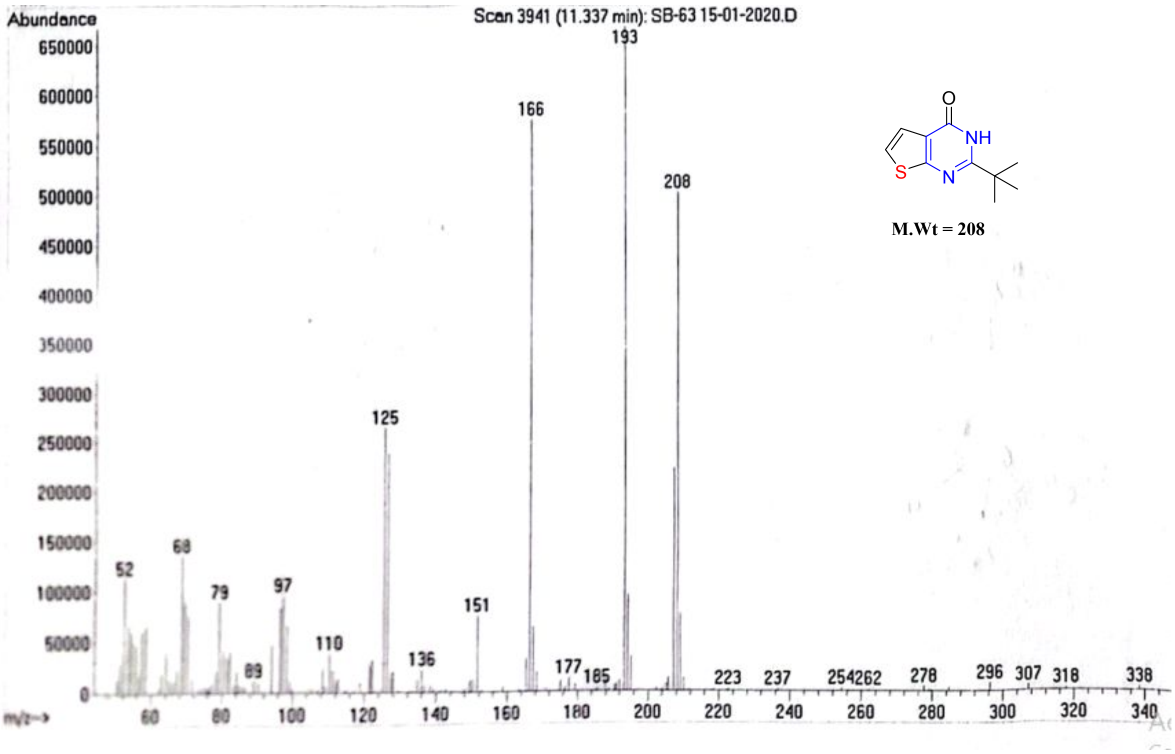

Fig. S-16: GCMS Spectrum of **1c**

4-Chloro-2-phenylthieno[2,3-*d*]pyrimidine (2a)

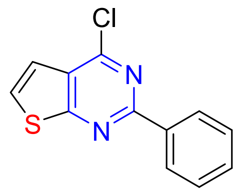

Yield: 75%

mp = 142-144 ˚C (lit. [16] 143-146 ˚C)

I.R: (*v,* cm^{-1}) 3094-3079, 1556-1407, 764-583, 707-688

^{1}H NMR (300 MHz, $CDCl_3$): δ (ppm) 8.55-8.49 (m, 2H), 7.55-7.48 (m, 4H), 7.43-7.41 (d, *J* = 6.0 Hz, 1H)

^{13}C NMR (75 MHz, $CDCl_3$): δ (ppm) 169.9, 160.1, 155.2, 136.4, 131.2, 128.8, 128.7, 127.5, 120.1

GC-MS Analysis (m/z): M^+ = 246, 211, 103, 77, 51

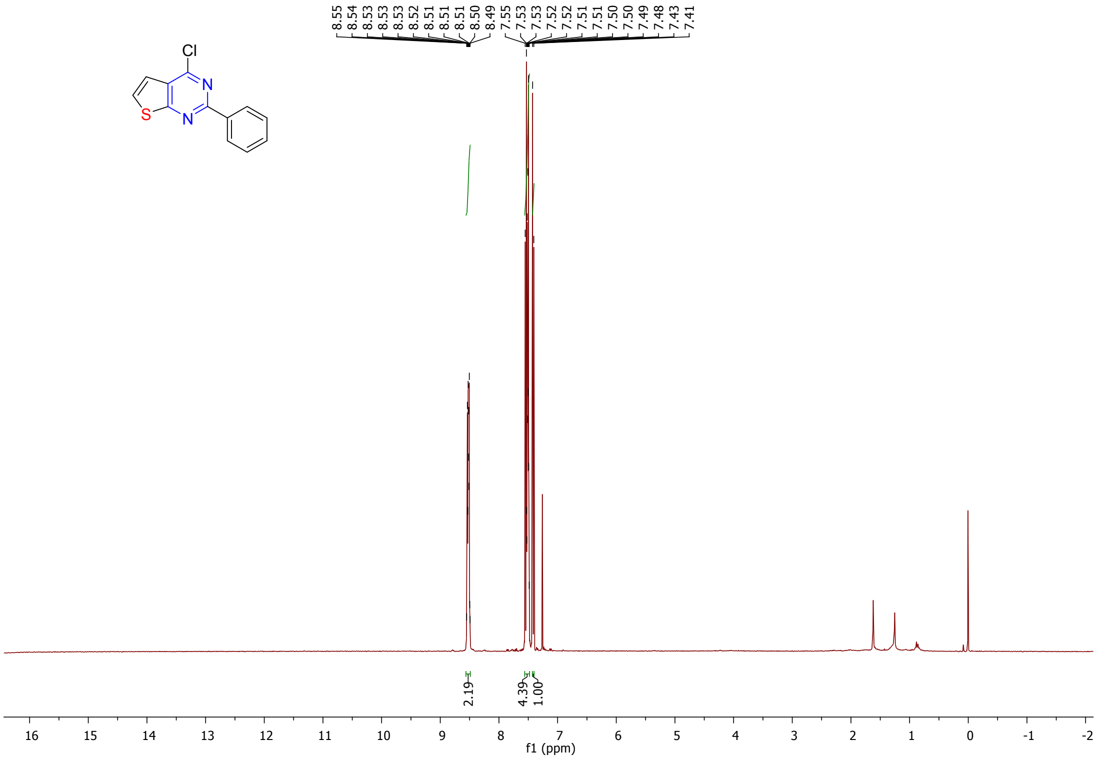

Fig. S-17: ^{1}HNMR Spectrum of **2a**

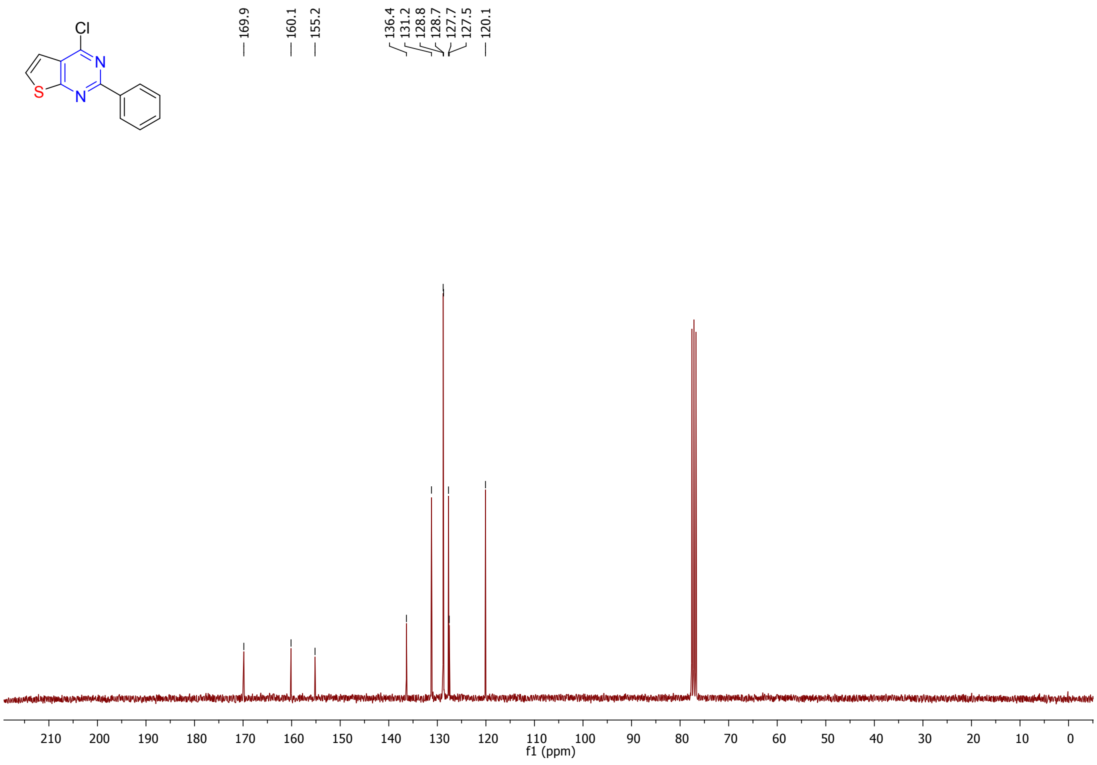

Fig. S-18: ^{13}C NMR Spectrum of **2a**

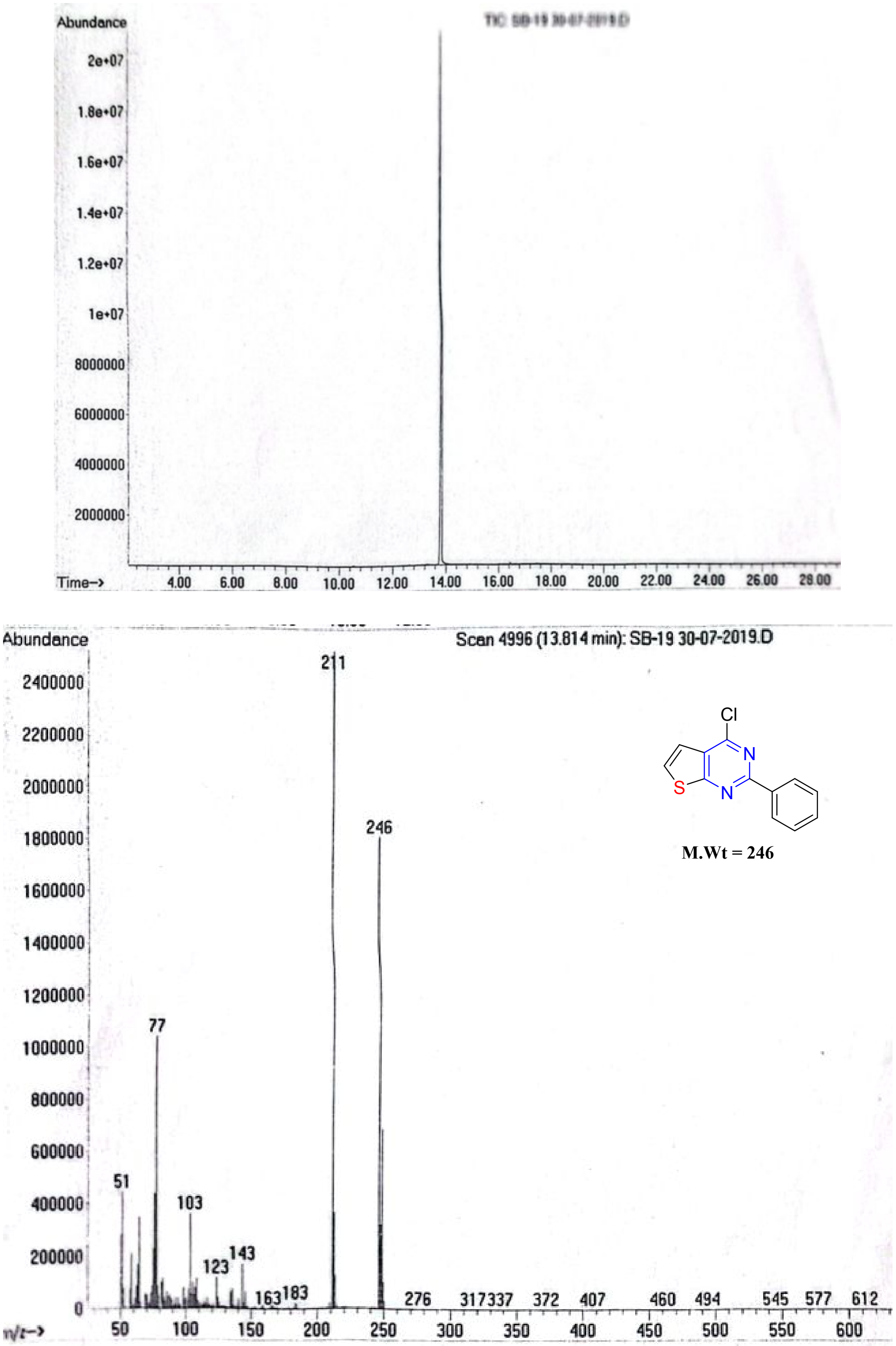

Fig. S-19: GCMS Spectrum of **2a**

4-Chloro-2-methylthieno[2,3-*d*]pyrimidine (2b)

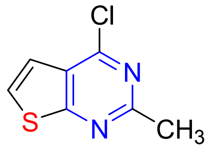

Yield: 72%

mp = 90-95 °C (lit. [17] 92-94 °C)

I.R: (*v*, cm^{-1}) 3058-3024, 1589-1434, 767-577, 714-667

^{1}H NMR (300 MHz, $CDCl_3$): δ (ppm) 7.50-7.48 (d, *J* = 6.0 Hz, 1H), 7.38-7.36 (d, *J* = 6.0 Hz, 1H), 2.80 (s, 3H)

^{13}C NMR (75 MHz, $CDCl_3$): δ (ppm) 169.6, 163.3, 154.8, 127.0, 119.9, 25.7

GC-MS Analysis (m/z): M^+ = 184, 149, 41

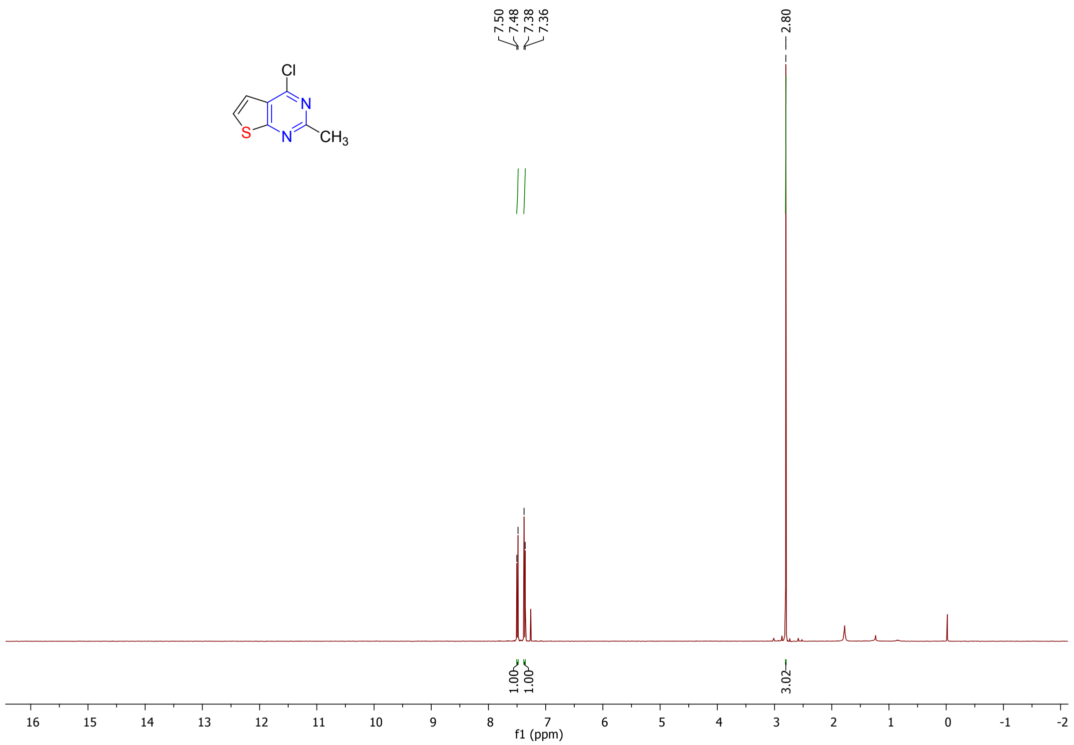

Fig. S-20: ^{1}HNMR Spectrum of **2b**

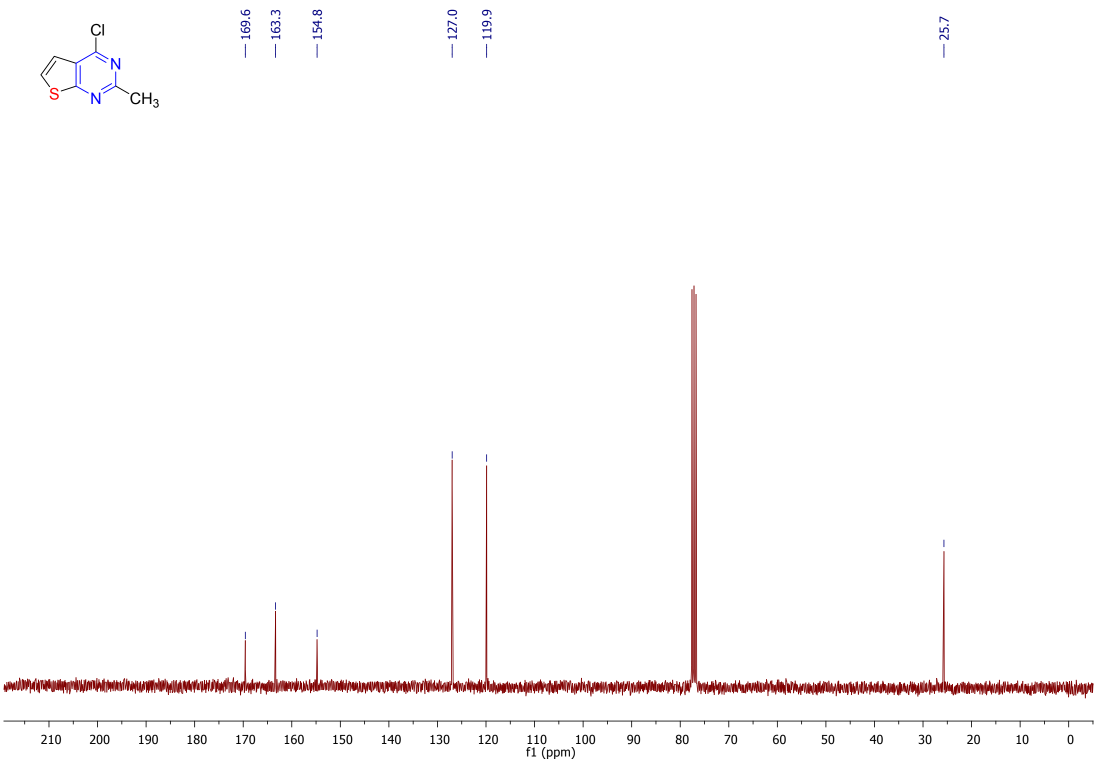

Fig. S-21: ^{13}C NMR Spectrum of **2b**

2-(*tert*-Butyl)-4-chlorothieno[2,3-*d*]pyrimidine (2c)

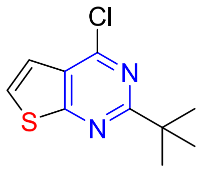

Yield: 60%

mp = 105-108 ˚C

I.R: (*v,* cm^{-1}) 3067-3015, 1582-1413, 762-567, 718-654

^{1}H NMR (300 MHz, $CDCl_3$): δ (ppm) 7.51-7.49 (d, *J* = 6.0 Hz, 1H), 7.38-7.36 (d, *J* = 6.0 Hz, 1H), 1.46 (s, 9H)

^{13}C NMR (75 MHz, $CDCl_3$): δ (ppm) 173.0, 169.2, 154.6, 127.7, 119.7, 39.6, 29.7

GC-MS Analysis (m/z): M^+ = 226, 191, 125, 83, 57

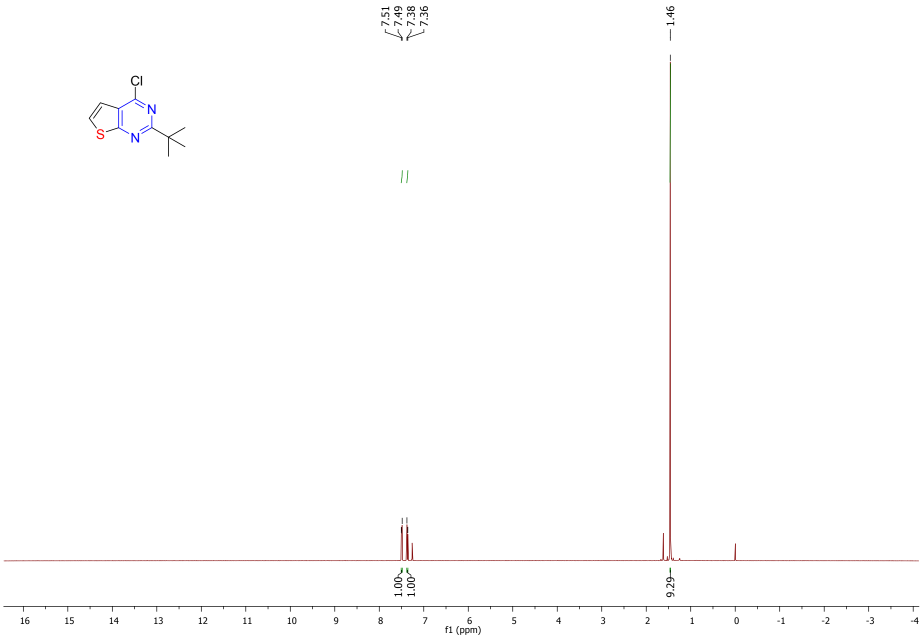

Fig. S-22: ^{1}HNMR Spectrum of **2c**

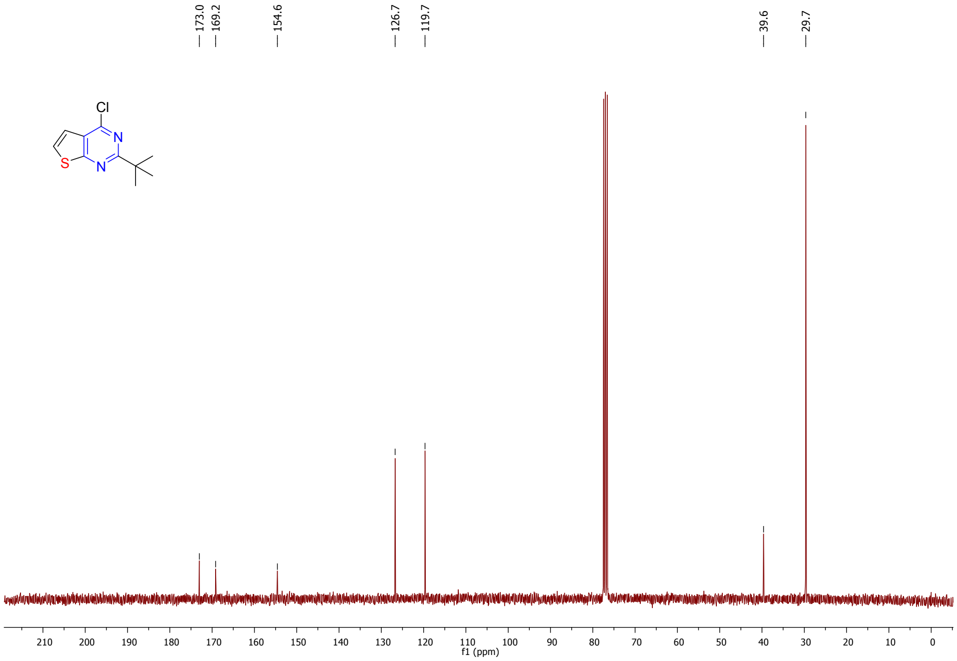

Fig. S-23: ^{13}C NMR Spectrum of **2c**

Analytical Data of Final Products

N-Benzyl-*N*-methyl-2-phenylthieno[2,3-*d*]pyrimidin-4-amine (3a)

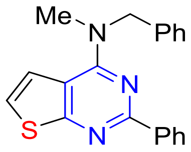

Yield: 64%

mp = 159-162 °C

IR: (*v*, cm^{-1}) 3100, 2970, 1550-1349, 1217-1014, 752-681

^{1}H NMR (300 MHz, $CDCl_3$): δ (ppm) 8.51-8.48 (m, 2H), 7.48-7.43 (m, 3H), 7.37-7.30 (m, 6H), 7.13-7.11 (d, *J* = 6.0 Hz, 1H), 5.13 (s, 1H), 3.43 (s, 3H)

^{13}C NMR (75 MHz, $CDCl_3$): δ (ppm) 170.9, 159.0, 158.7, 138.4, 137.8, 130.1, 129.0, 128.4, 128.4, 127.6, 127.3, 121.5, 120.7, 114.1, 54.8, 38.3

GC-MS Analysis (m/z): M^+ = 331, 316, 302, 240, 211, 120, 91, 77, 65

HRMS-ESI (m/z): $[M+H]^+$ calc'd for $C_{20}H_{18}N_3S^+$, 332.1154; found, 332.1159

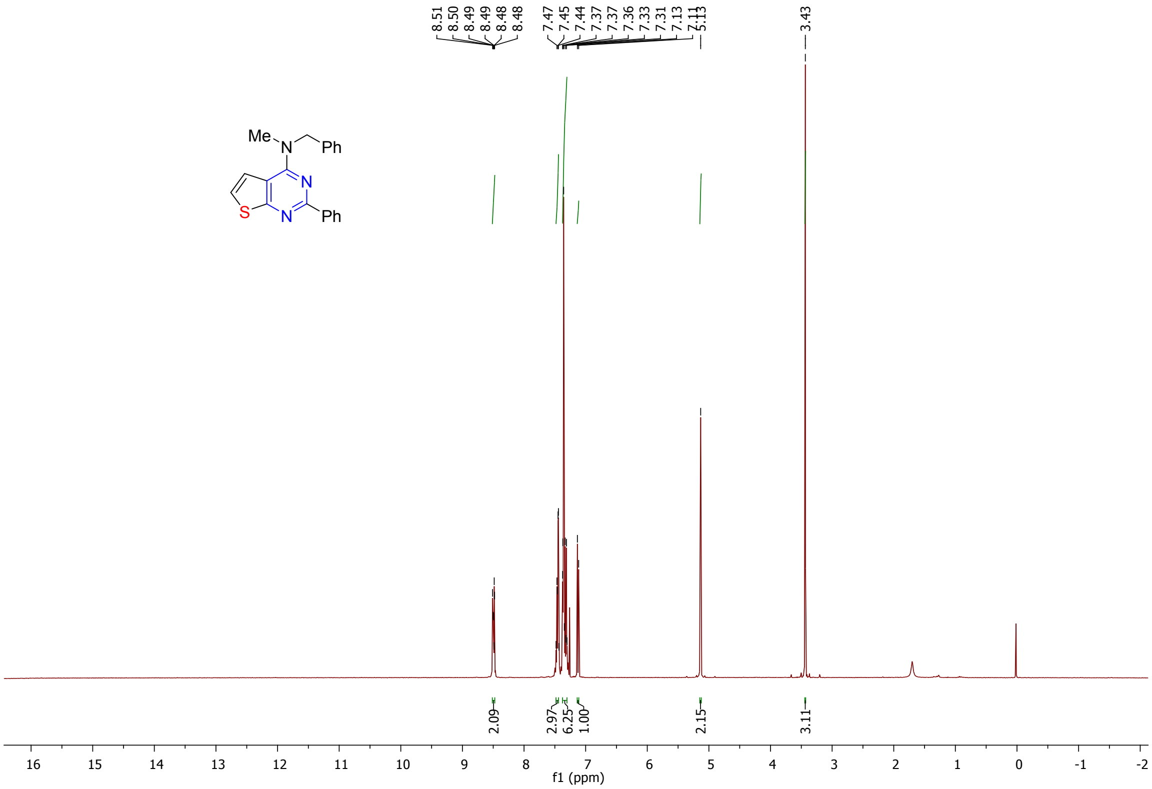

Fig. S-24: ^{1}HNMR Spectrum of **3a**

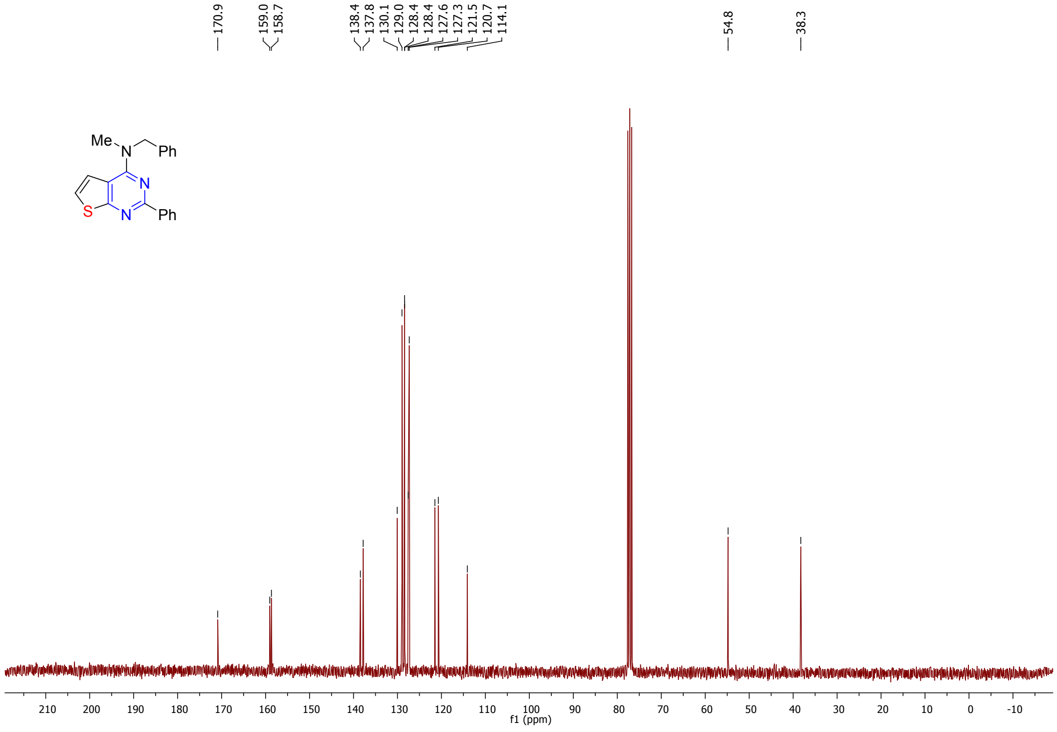

Fig. S-25: ^{13}C NMR Spectrum of **3a**

File : C:\MSDChem\1\DATA\2019\Dr.Abbas Hassan\Sania Batool\SB-35 10
-10-19.D
Operator : Saqib Yasin
Instrument : Instrument #1
Acquired : 10 Oct 2019 10:25 using AcqMethod LIQUID.M
Sample Name: SB-35
Misc Info : Temp 120-280 10 C/min flow 1.5ml/min inj 5ul

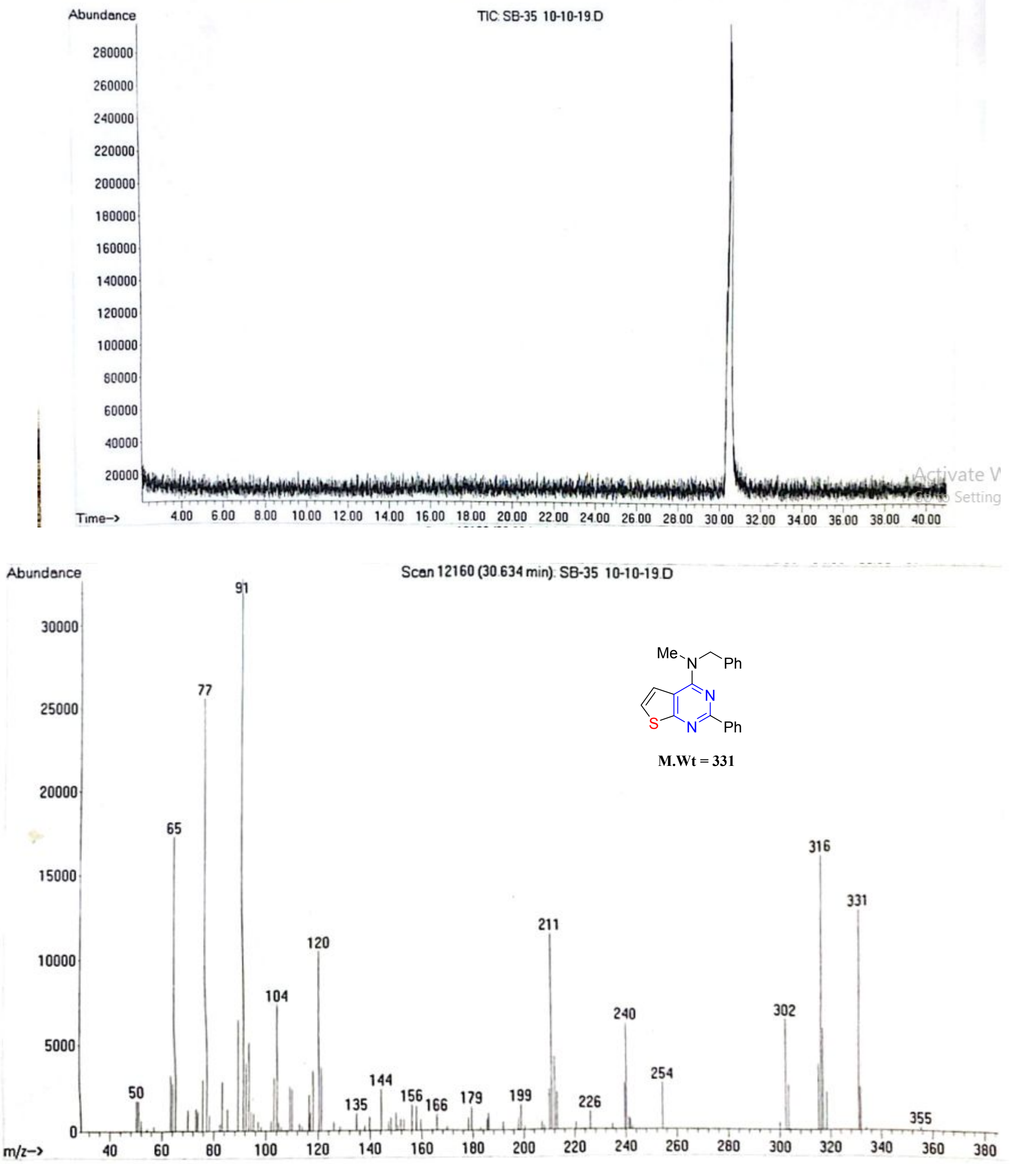

Fig. S-26: GCMS Spectrum of **3a**

2-Phenyl-4-(pyrrolidin-1-yl)thieno[2,3-*d*]pyrimidine (3b)

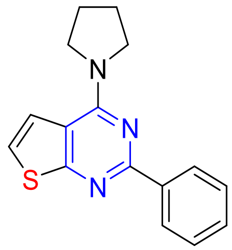

Yield: 66%

Mp = 152-154 °C

IR: (*v,* cm^{-1}) 2921, 1545-1388, 1291-1020, 769-675

^{1}H NMR (300 MHz, $CDCl_3$): δ (ppm) 8.51-8.48 (2H, m), 7.49-7.40 (4H, m), 7.11-7.09 (d, *J* = 6.0 Hz, 1H), 3.90-3.86 (m, 4H), 2.04 (*ap.* s, 4H)

^{13}C NMR (75 MHz, $CDCl_3$): δ (ppm) 169.7, 159.3, 156.1, 138.6, 129.9, 128.3, 121.2, 119.9, 114.5, 48.8, 25.6

GC-MS Analysis (m/z): M^+ = 281, 252, 211, 77, 70, 51

HRMS-ESI (m/z): $[M+H]^+$ calc'd for $C_{16}H_{16}N_3S^+$, 282.0961; found, 282.0967

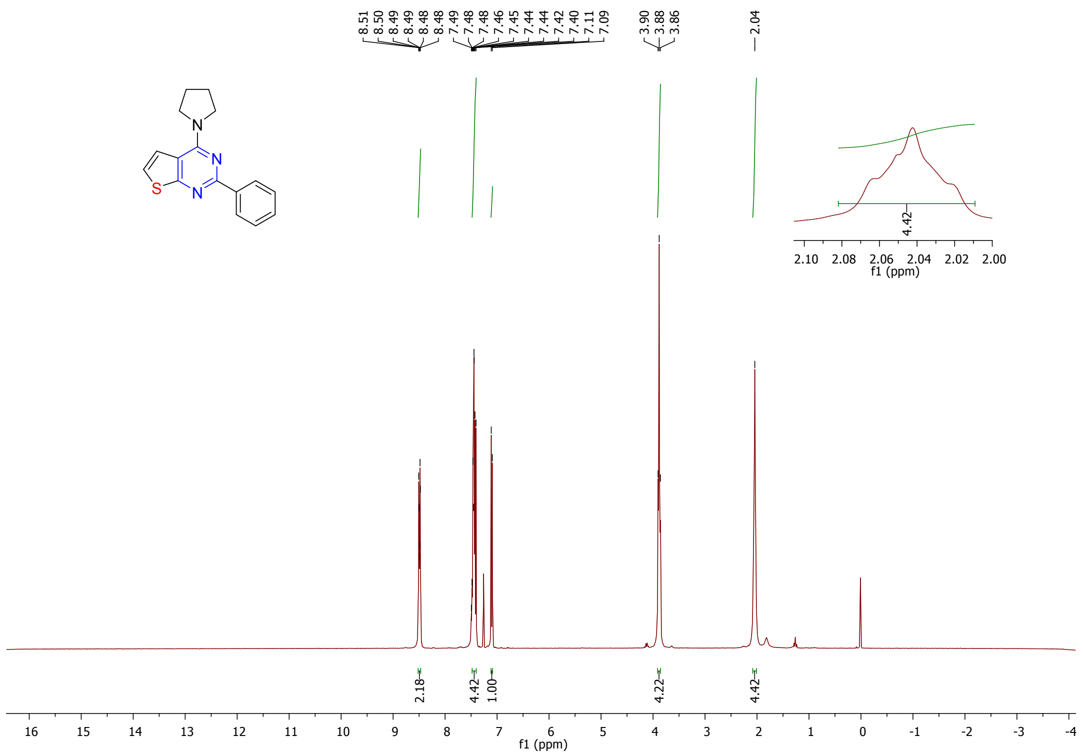

Fig. S-27: ^{1}HNMR Spectrum of **3b**

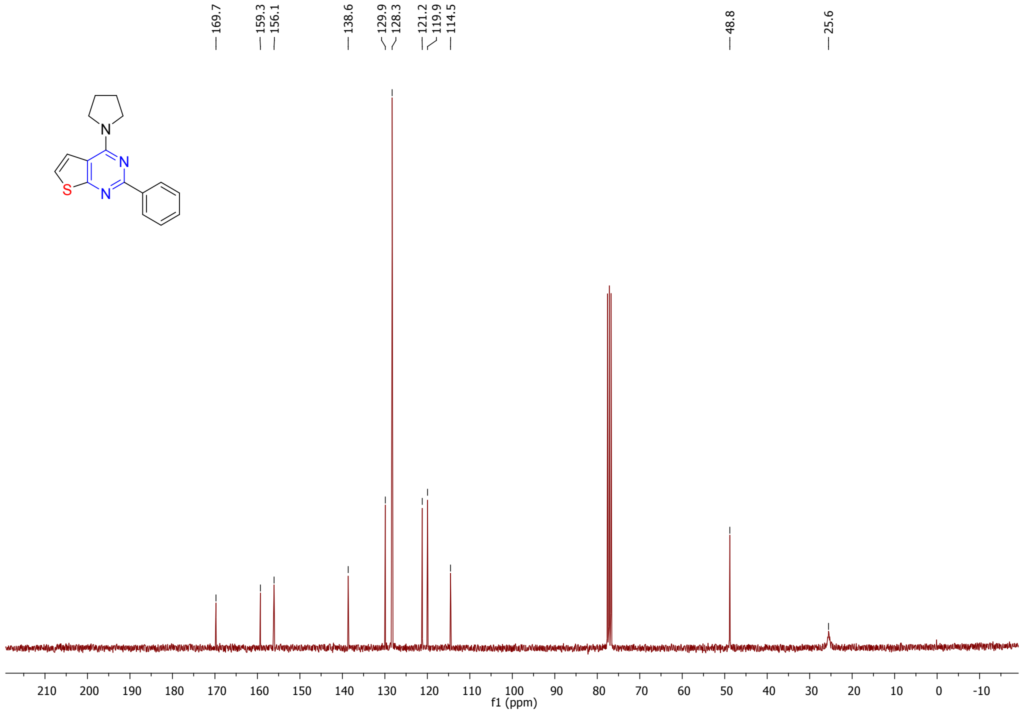

Fig. S-28: ^{13}C NMR Spectrum of **3b**

4-(2-Phenylthieno[2,3-*d*]pyrimidin-4-yl)morpholine (3c)

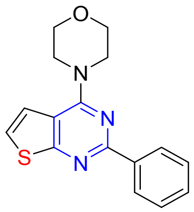

Yield: 69%

mp = 143-146 ˚C (lit.[14] 140 ˚C)

IR: (*v,* cm^{-1}) 3081, 2959-2846, 1514-1362, 1305-985, 716-698, 689

^{1}H NMR (300 MHz, Acetone d_6): δ (ppm) 8.53-8.48 (2H, m), 7.62-7.60 (d, *J* = 6.0 Hz, 1H), 7.52-7.46 (m, 4H), 4.05-4.01 (m, 4H), 3.87-3.84 (m, 4H)

^{13}C NMR (75 MHz, Acetone d_6): δ (ppm) 171.5, 159.6, 159.2, 139.1, 130.9, 129.1, 128.9, 122.8, 122.0, 115.7, 67.3, 48.0

GC-MS Analysis (m/z): M^+ = 297, 266, 240, 211, 135, 104

HRMS-ESI (m/z): $[M+H]^+$ calc'd for $C_{16}H_{16}N_3OS^+$, 298.0923; found, 298.0926

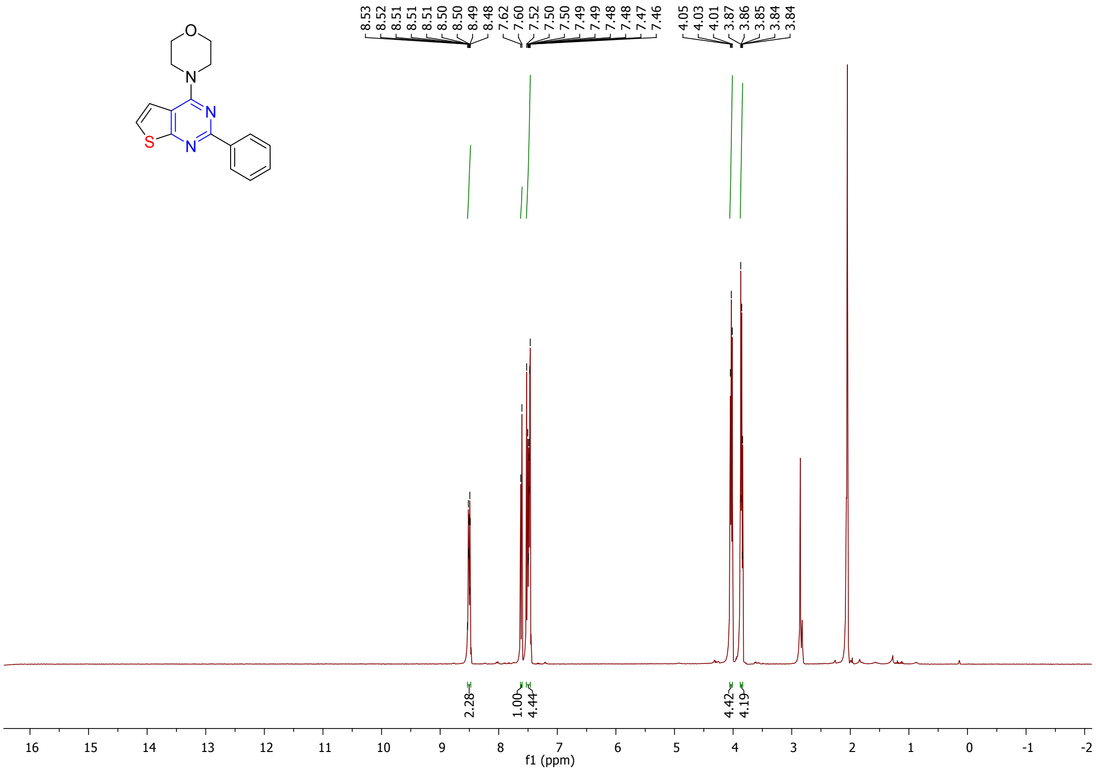

Fig. S-29: ^{1}HNMR Spectrum of **3c**

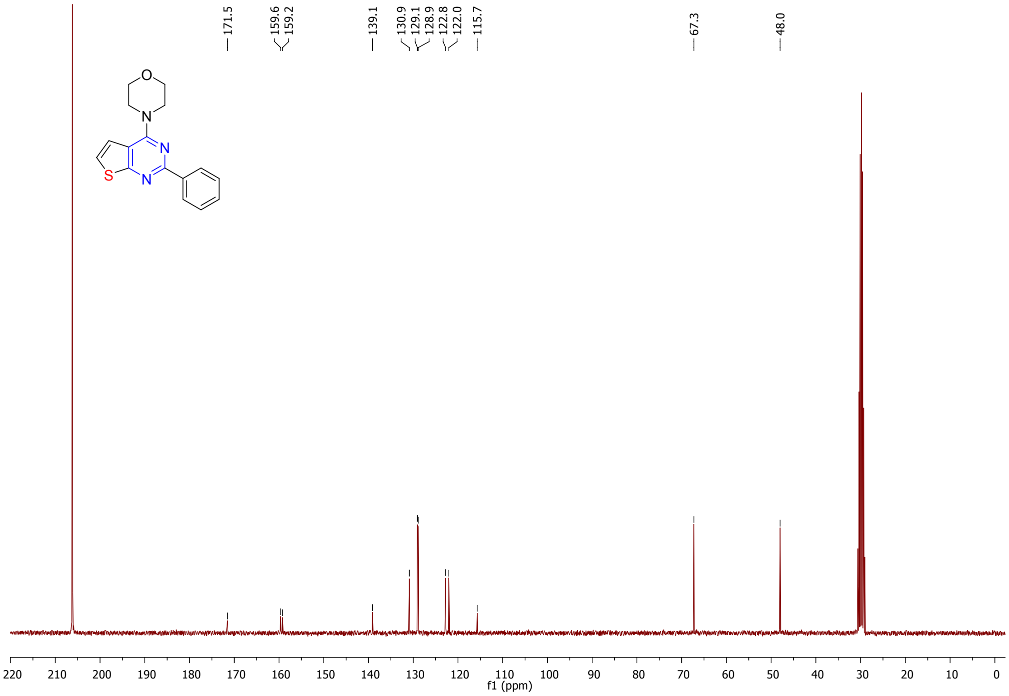

Fig. S-30: ^{13}C NMR Spectrum of **3c**

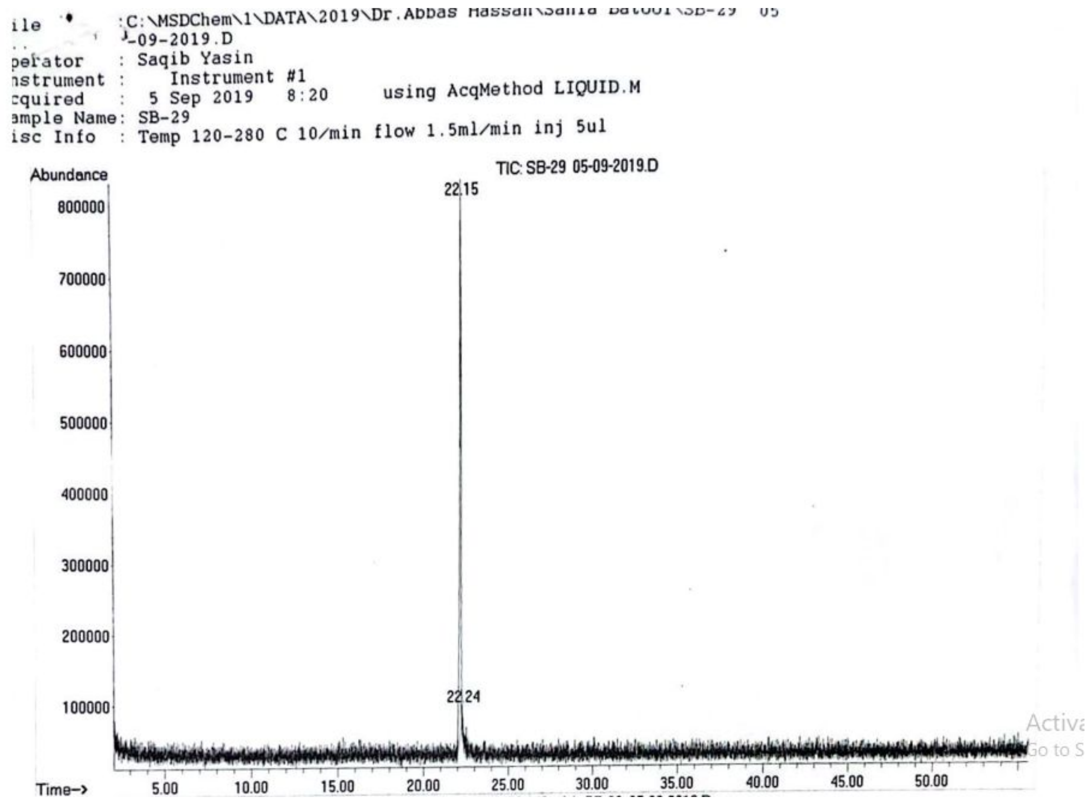

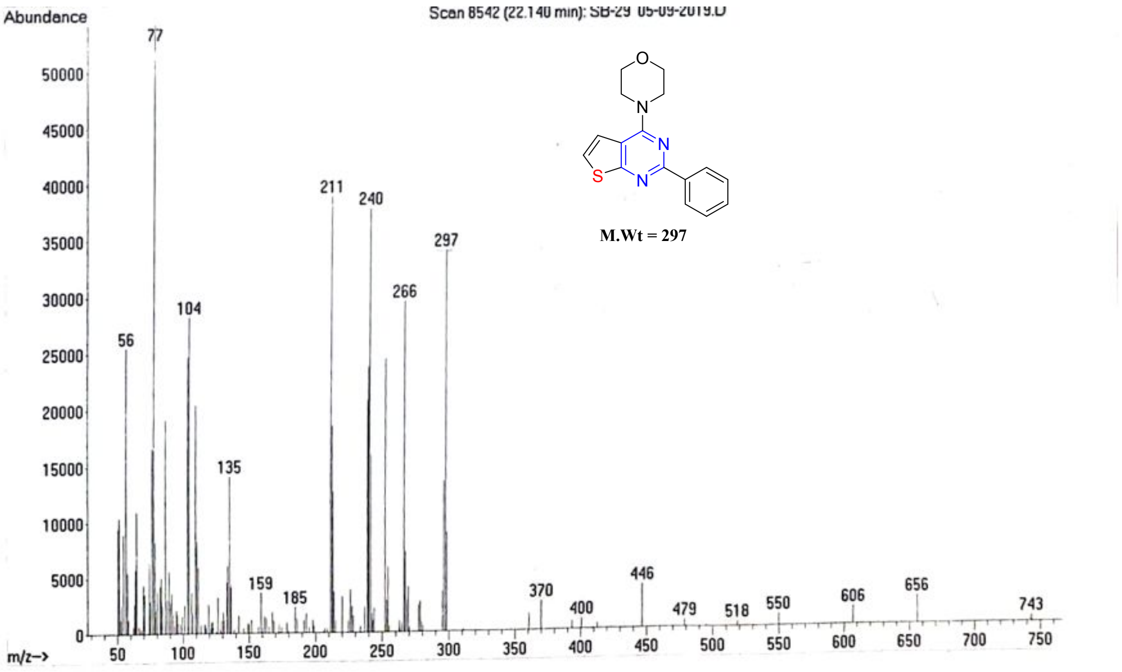

Fig. S-31: GCMS Spectrum of **3c**

2-Phenyl-*N*-(pyridin-2-ylmethyl)thieno[2,3-*d*]pyrimidin-4-amine (3d)

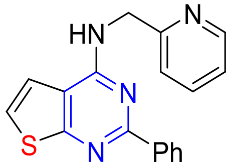

Yield: 67%

mp = 217-221 °C

IR: (*v,* cm^{-1}) 3231-3068, 2906, 1589-1396, 1332-1028, 998-602

^{1}H NMR (300 MHz, $CDCl_3$): δ (ppm) 8.66-8.65 (*ap.* d, 1H), 8.57-8.54 (m, 2H), 7.79-7.73 (td, *J* = 9.0 Hz, 3.0 Hz, 1H), 7.57-7.50 (m, 4H), 7.32-7.24 (m, 3H), 7.03-7.00 (*ap.* t, 1H) 5.10-5.09 (s, 2H)

^{13}C NMR (75 MHz, $CDCl_3$): δ (ppm) 167.9, 160.1, 157.2, 156.8, 149.1, 138.6, 137.0, 130.1, 128.4, 122.7, 122.6, 122.5, 117.5, 115.1, 45.7

GC-MS Analysis (m/z): M^+ = 318, 240, 211, 107, 93, 77, 65, 52

HRMS-ESI (m/z): $[M+H]^+$ calc'd for $C_{18}H_{15}N_4S^+$, 319.0946; found, 319.0951

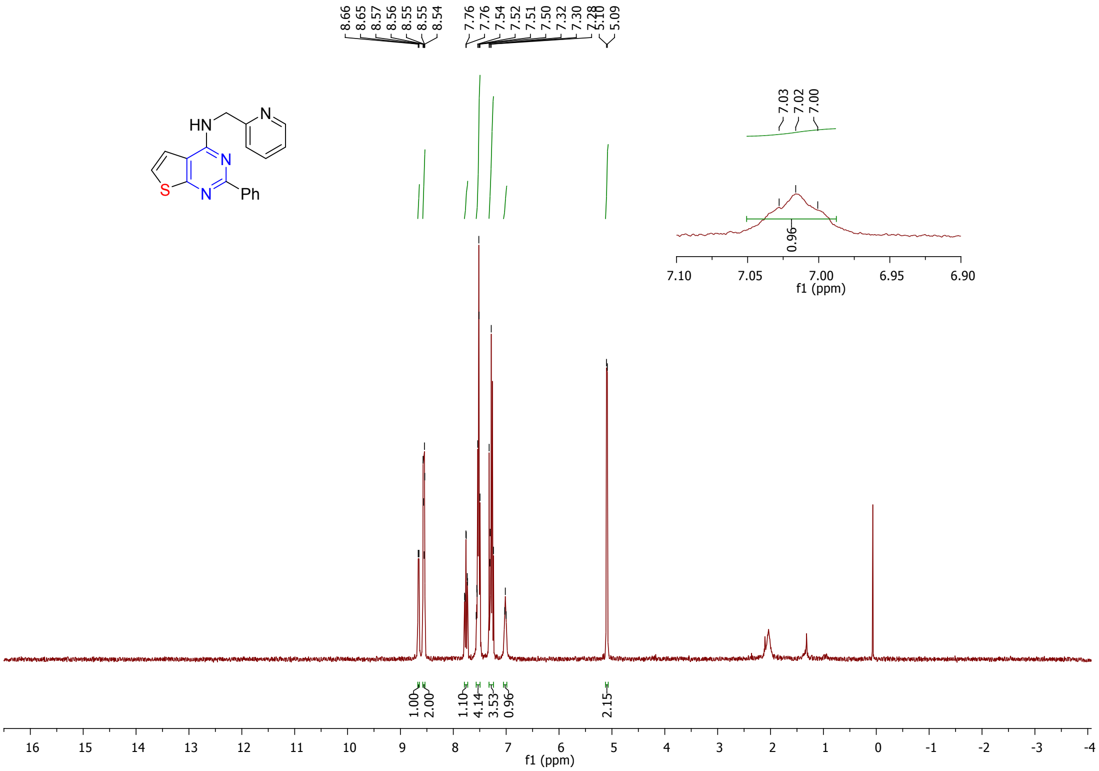

Fig. S-32: ^{1}HNMR Spectrum of **3d**

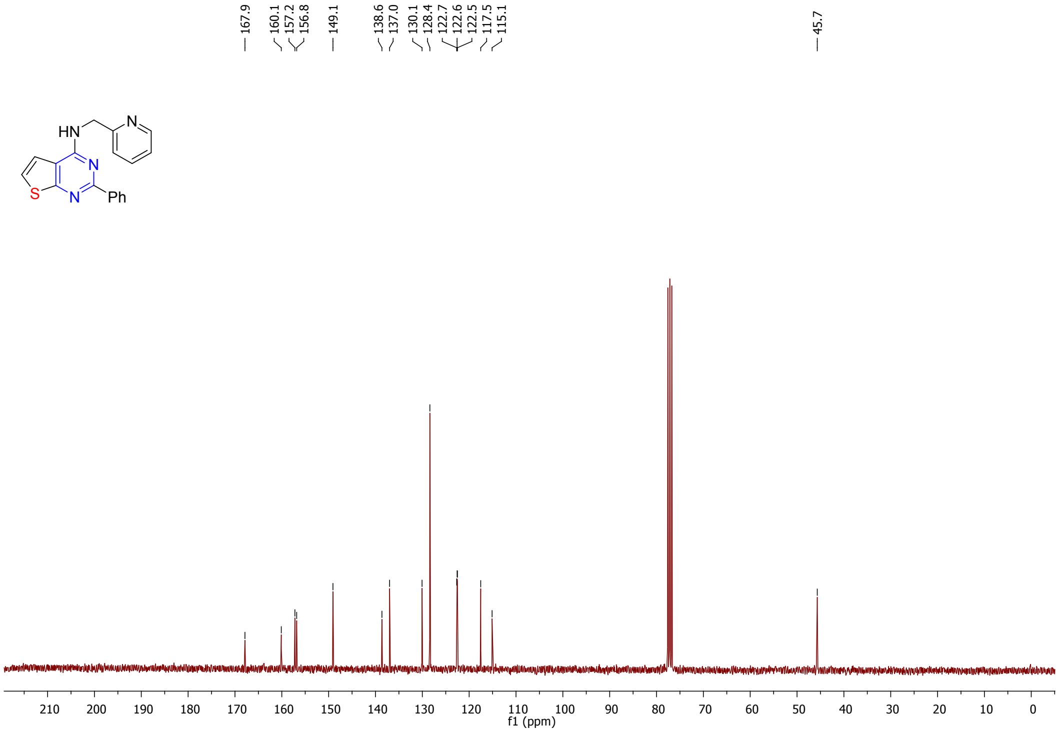

Fig. S-33: ^{13}C NMR Spectrum of **3d**

:C:\MSDChem\1\DATA\2019\Dr.Abbas Hassan\Sania Batool\SB-36 16-[illegible]-19.D
[illegible]or : Saqib Yasin
[illegible]ument : Instrument #1
[illegible]quired : 16 Oct 2019 10:57 using AcqMethod LIQUID 50 TO 500.M
[illegible]ample Name: SB-36
[illegible]isc Info : Temp 120-280 10 C/min flow 1.5ml/min Inj 5ul

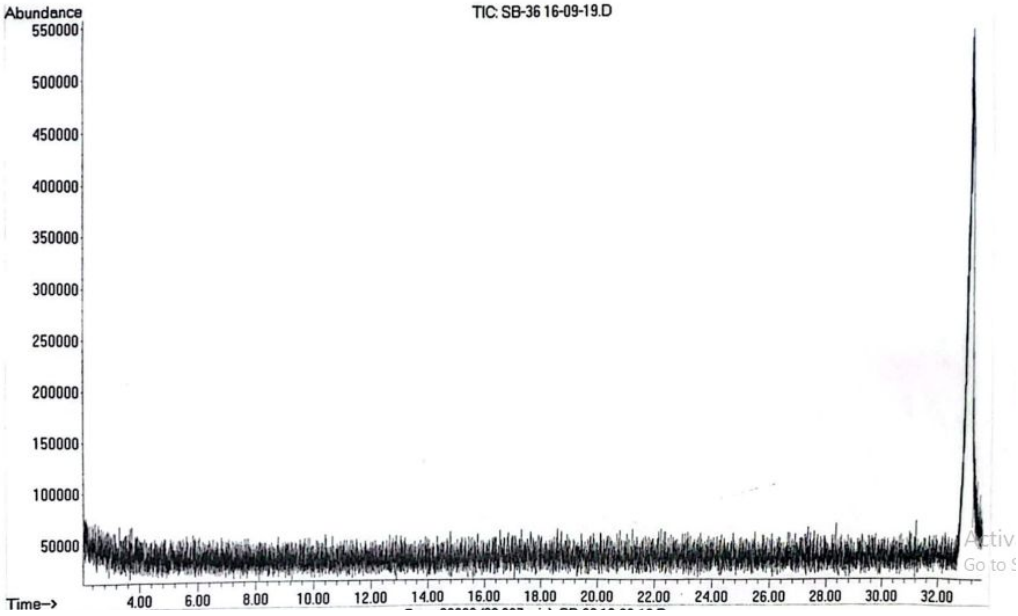

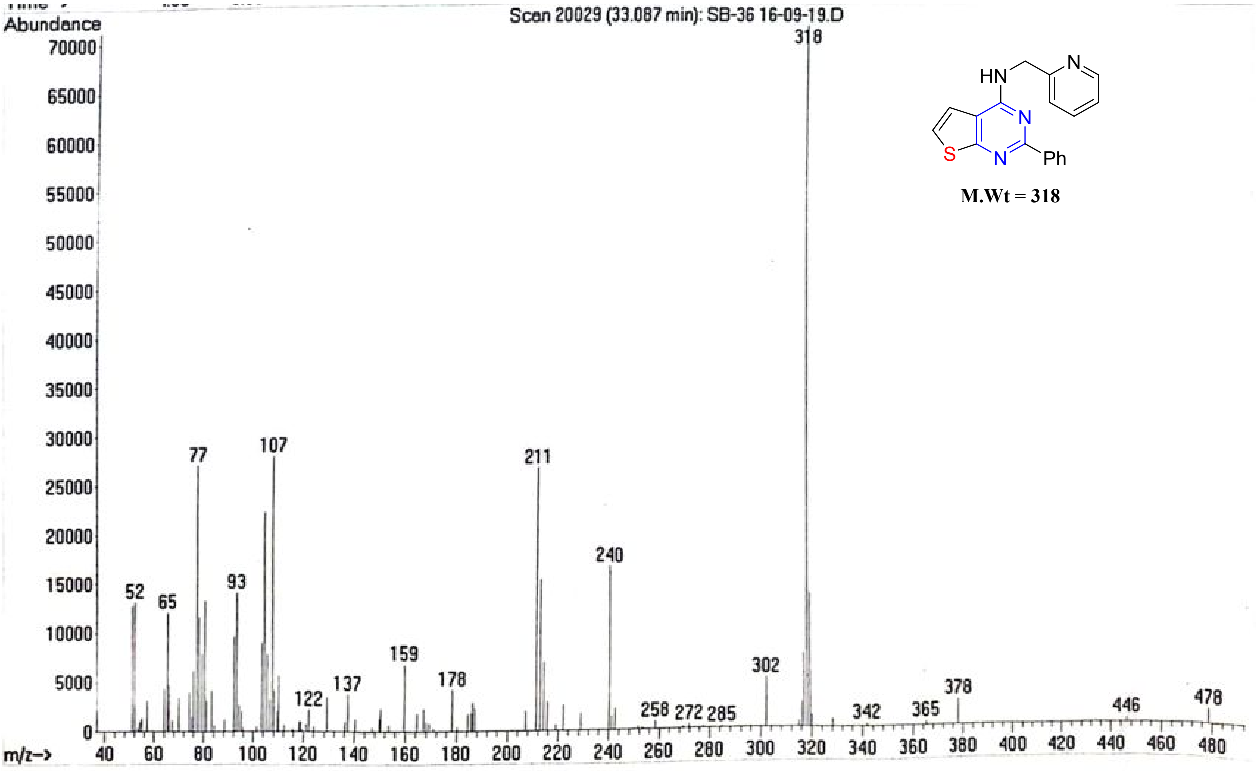

M.Wt = 318

Fig. S-34: GCMS Spectrum of **3d**

4-(1*H*-Imidazol-1-yl)-2-phenylthieno[2,3-*d*]pyrimidine (3e)

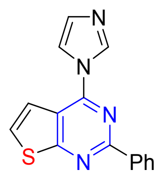

Yield: 64%

mp = 163-164 °C

IR: (*v,* cm^{-1}) 3148-3104, 1551-1403, 1305-1024, 993-648

^{1}H NMR (300 MHz, $CDCl_3$): δ (ppm) 8.56-8.52 (m, 3H), 7.89 (s, 1H), 7.64-7.62 (d, *J* = 6.0 Hz, 1H), 7.55-7.50 (m, 4H), 7.32 (s, 1H)

^{13}C NMR (75 MHz, $CDCl_3$): δ (ppm) 172.8, 159.8, 149.7, 136.8, 136.5, 131.3, 131.2, 128.8, 128.6, 128.1, 118.7, 118.1, 118.1

GC-MS Analysis (m/z): M^+ = 278, 250, 211, 104, 77, 64, 51

HRMS-ESI (m/z): $[M+H]^+$ calc'd for $C_{15}H_{11}N_4S^+$, 279.0535; found, 279.0538

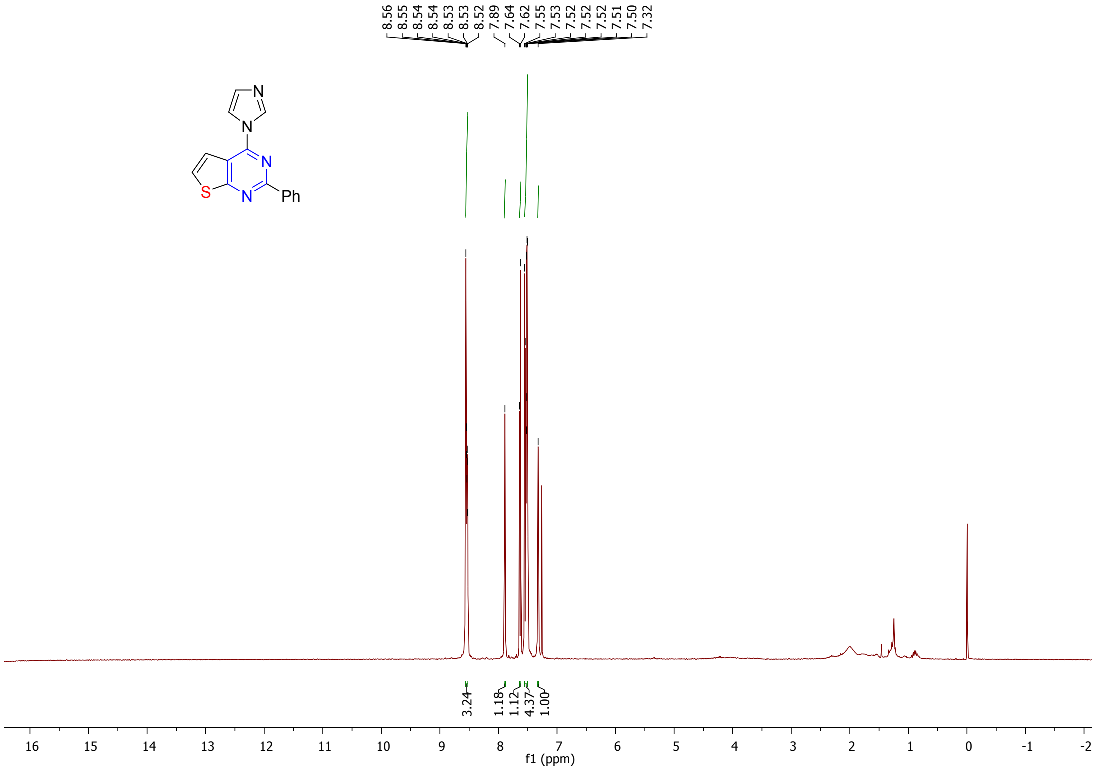

Fig. S-35: ^{1}HNMR Spectrum of **3e**

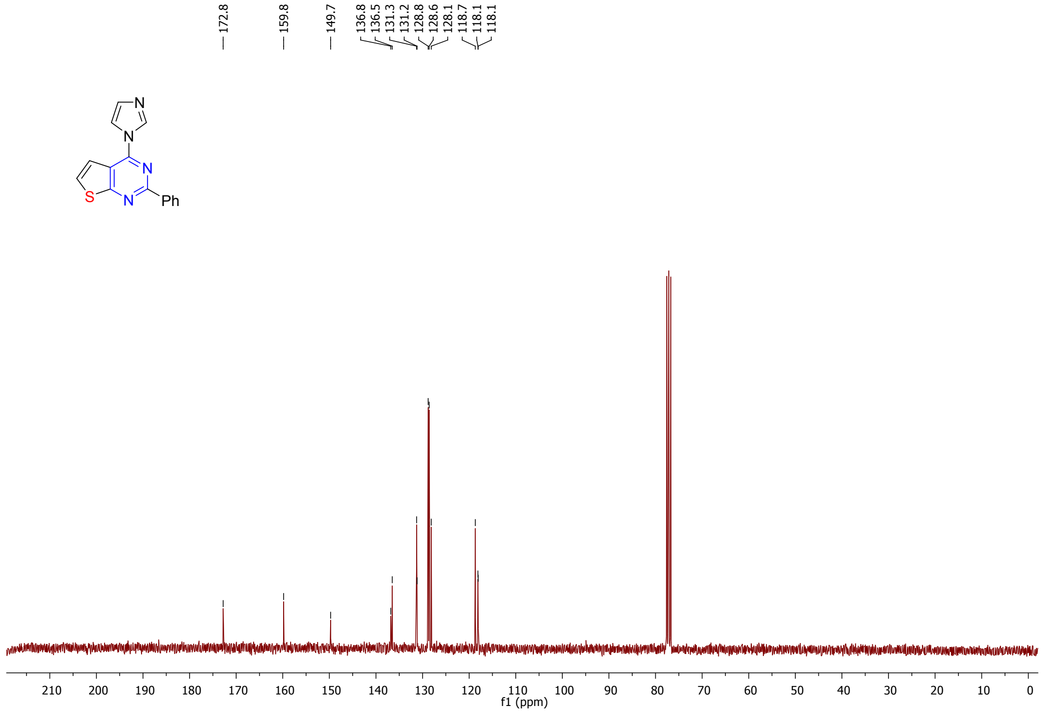

Fig. S-36: ^{13}C NMR Spectrum of **3e**

File[redacted] :C:\MSDChem\1\DATA\2019\Dr.Abbas Hassan\Sania Batool\SB-33 10
[redacted]-10-19.D
Op[redacted] : Saqib Yasin
Instrument : Instrument #1
Acquired : 10 Oct 2019 9:43 using AcqMethod LIQUID.M
Sample Name: SB-33
Misc Info : Temp 120-280 10 C/min flow 1.5ml/min inj 5ul

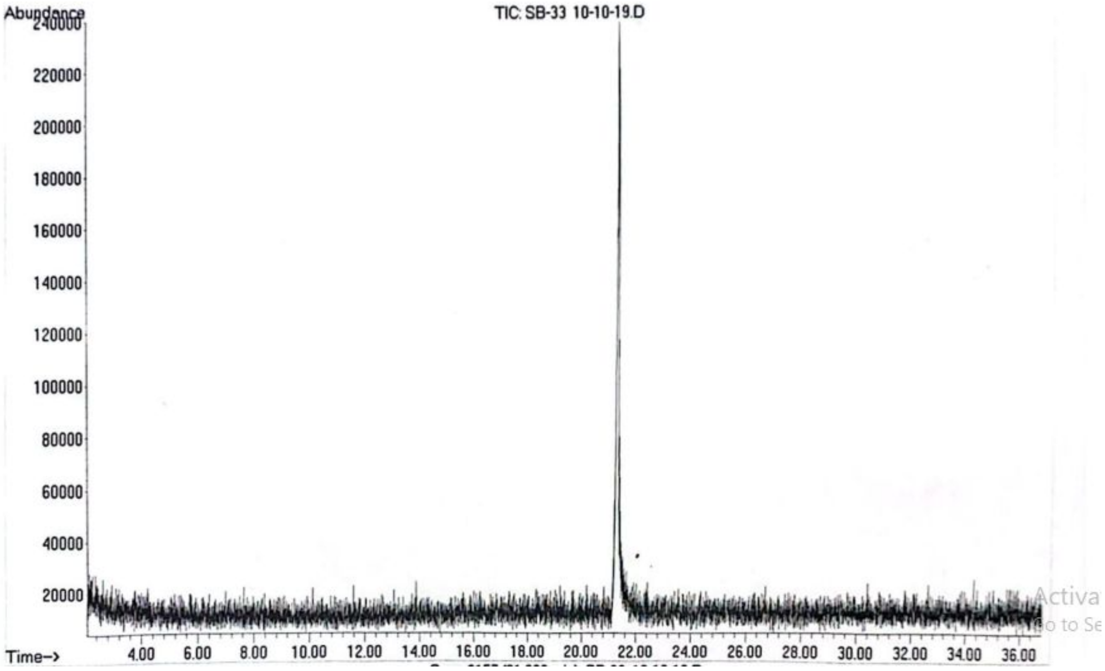

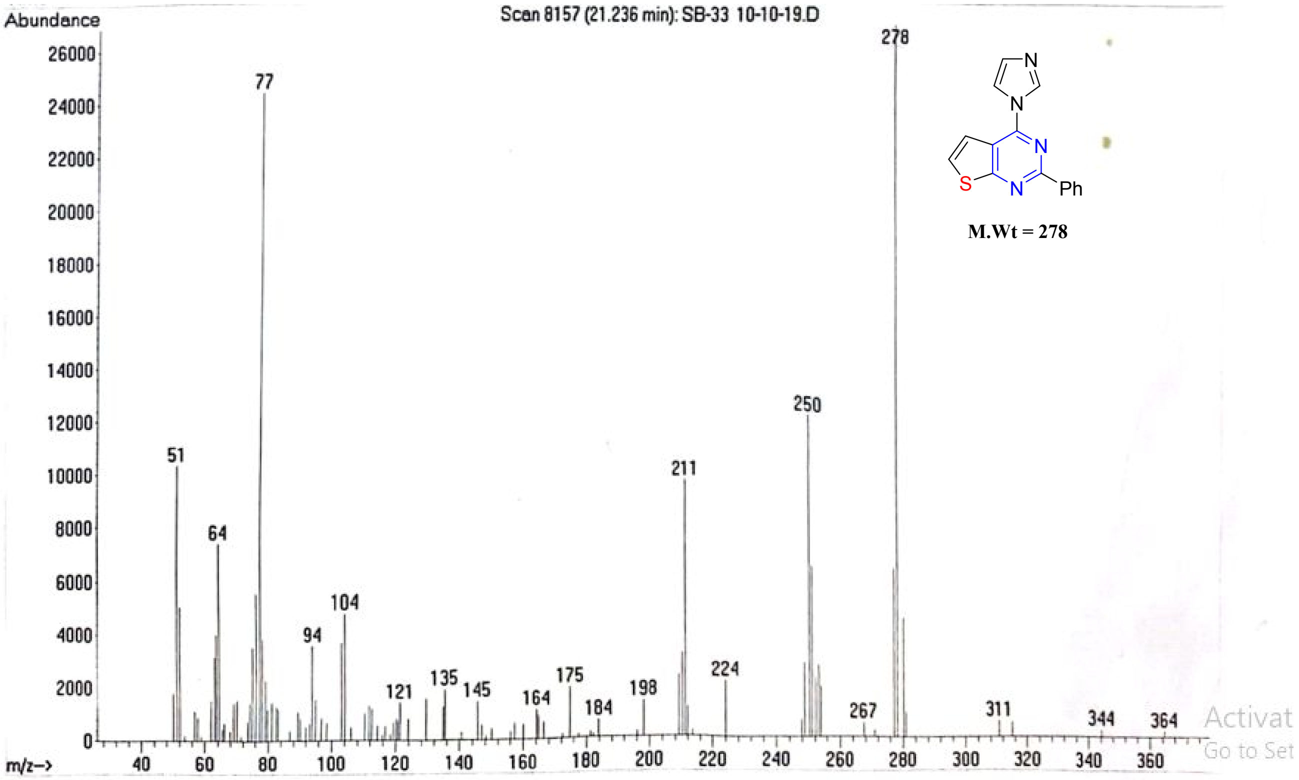

Fig. S-37: GCMS Spectrum of **3e**

4-(2-Methylthieno[2,3-*d*]pyrimidin-4-yl)morpholine (3f)

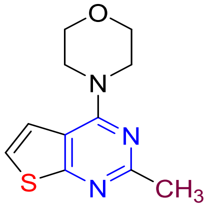

Yield: 71%

mp = 143-147 ˚C

IR: (*v,* cm^{-1}) 3083, 2958-2856, 1523-1357, 1303-900, 818-697

^{1}H NMR (300 MHz, $CDCl_3$): δ (ppm) 7.29-7.27 (d, *J* = 6.0 Hz, 1H), 7.19-7.17 (d, *J* = 6.0 Hz, 1H), 3.92-3.89 (m, 4H), 3.85-3.82 (m, 4H), 2.60 (s, 3H)

^{13}C NMR (75 MHz, $CDCl_3$): δ (ppm) 170.4, 162.4, 158.8, 121.0, 120.2, 114.1, 66.9, 47.2, 25.9

GC-MS Analysis (m/z): M^+ = 235, 204, 178, 149, 109, 86, 56

HRMS-ESI (m/z): $[M+H]^+$ calc'd for $C_{11}H_{14}N_3OS^+$ 236.0771; found, 236.0774

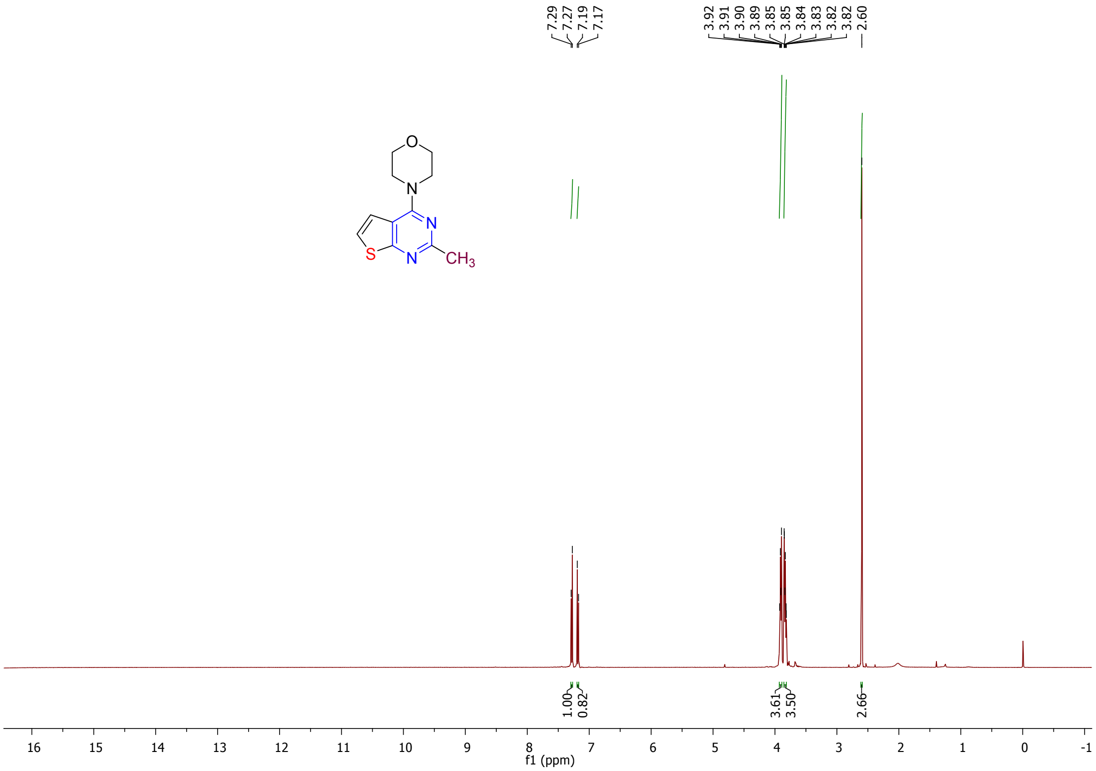

Fig. S-38: ^{1}HNMR Spectrum of **3f**

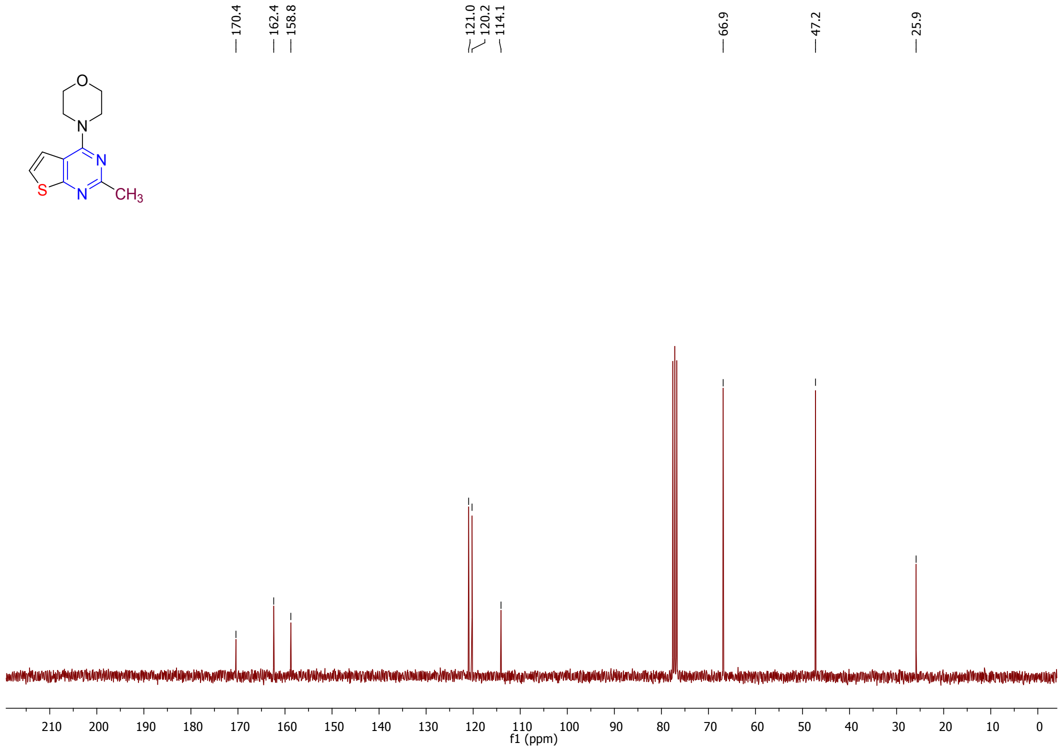

Fig. S-39: ^{13}C NMR Spectrum of **3f**

File : C:\MSDChem\1\DATA\2020\Dr. Abbas Hassan\Sania Batool\SB-71 18-03-20.D
Operator : Saqib Yasin
Instrument : Instrument #1
Acquired : 18 Mar 2020 15:34 using AcqMethod LIQUID.M
Sample Name: SB-71
Misc Info : Temp 120-280 10C/min Flow 1.5ml/min Inj 2ul

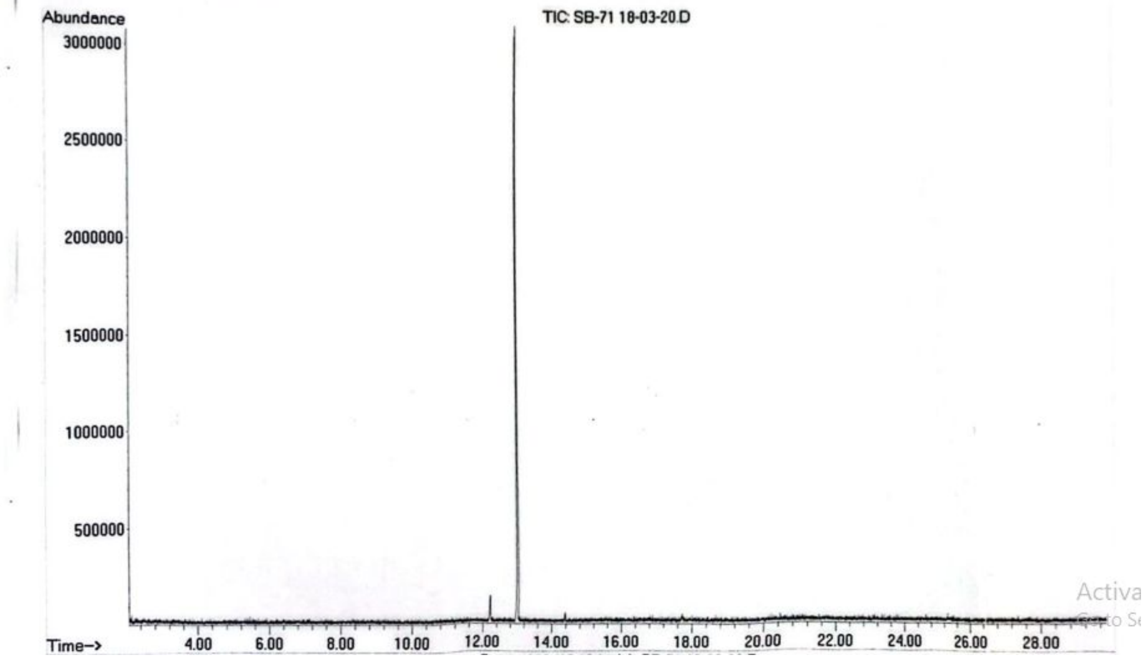

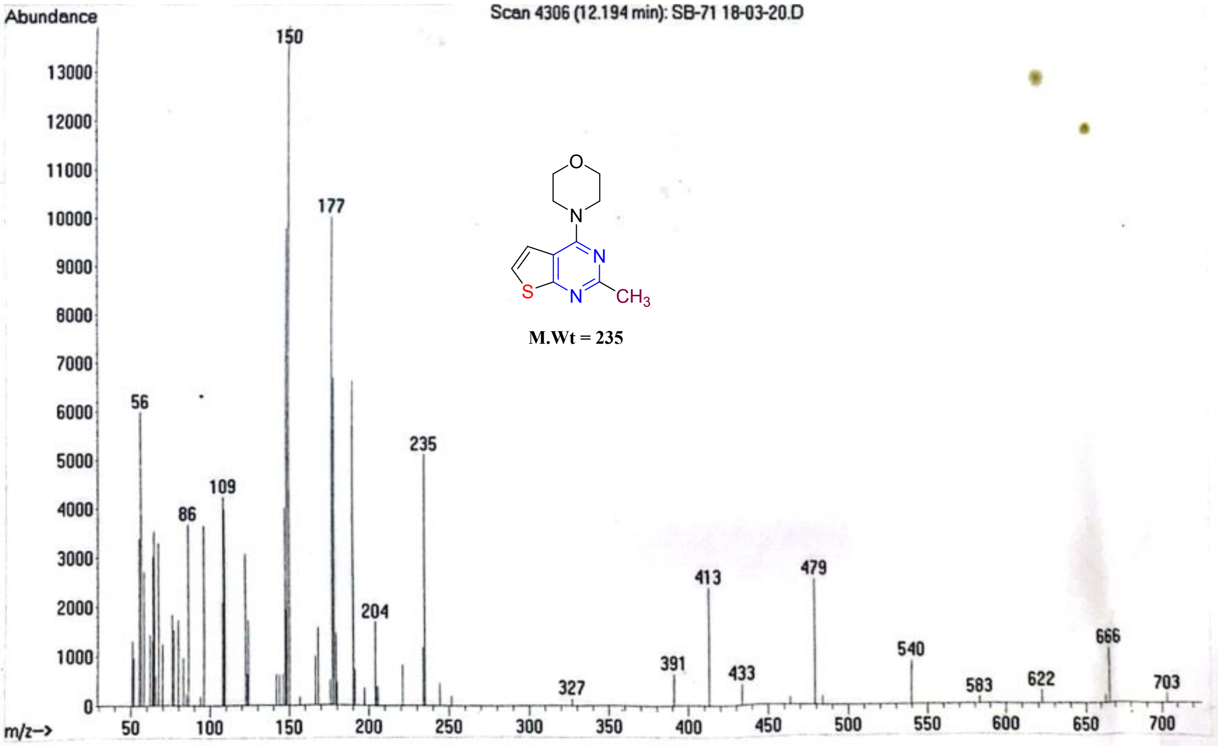

Fig. S-40: GCMS Spectrum of **3f**

4-(2-(*tert*-Butyl)thieno[2,3-*d*]pyrimidin-4-yl)morpholine (3g)

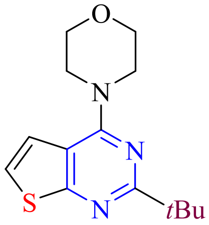

Yield: 67 %

mp = 135-138 ˚C

IR: (*v,* cm^{-1}) 3073, 2947-2854, 1535-1355, 1265-991, 816-633

^{1}H NMR (300 MHz, $CDCl_3$): δ (ppm) 7.56-7.54 (d, *J* = 6.0 Hz, 1H), 7.44-7.42 (d, *J* = 6.0 Hz, 1H), 3.94-3.91 (m, 4H), 3.81-3.78 (m, 4H), 1.37 (s, 9H)

^{13}C NMR (75 MHz, $CDCl_3$): δ (ppm) 171.8, 170.2, 159.4, 121.9, 121.7, 114.7, 67.2, 47.9, 39.7, 30.0

GC-MS Analysis (m/z): M^+ = 235, 178, 149, 109, 86, 56

HRMS-ESI (m/z): $[M+H]^+$ calc'd for $C_{14}H_{20}N_3OS^+$, 278.1356; found, 278.1359

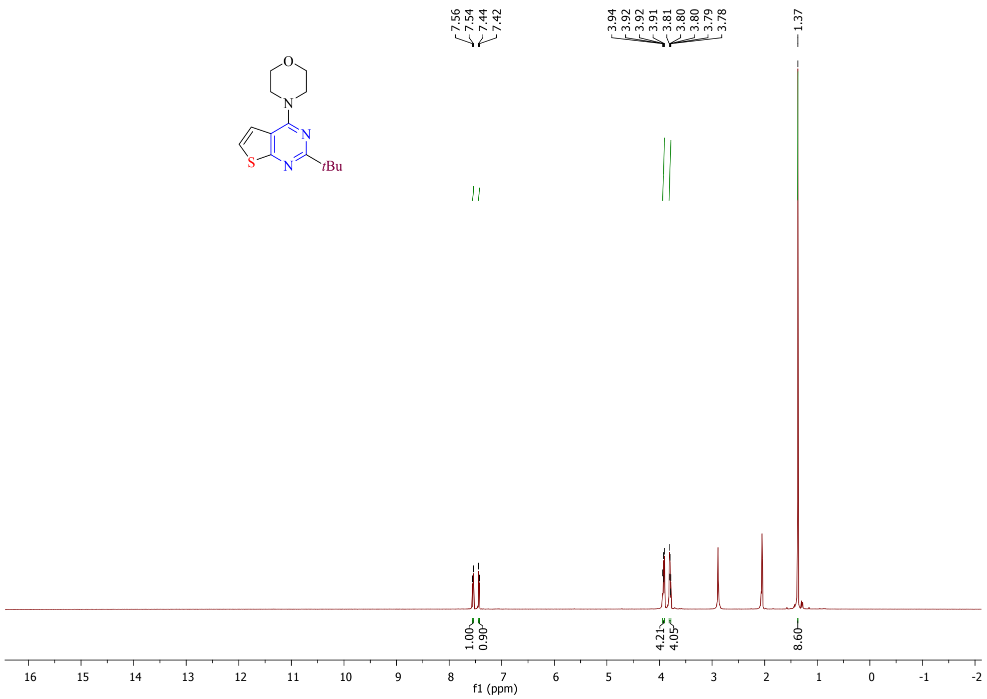

Fig. S-41: ^{1}HNMR Spectrum of **3g**

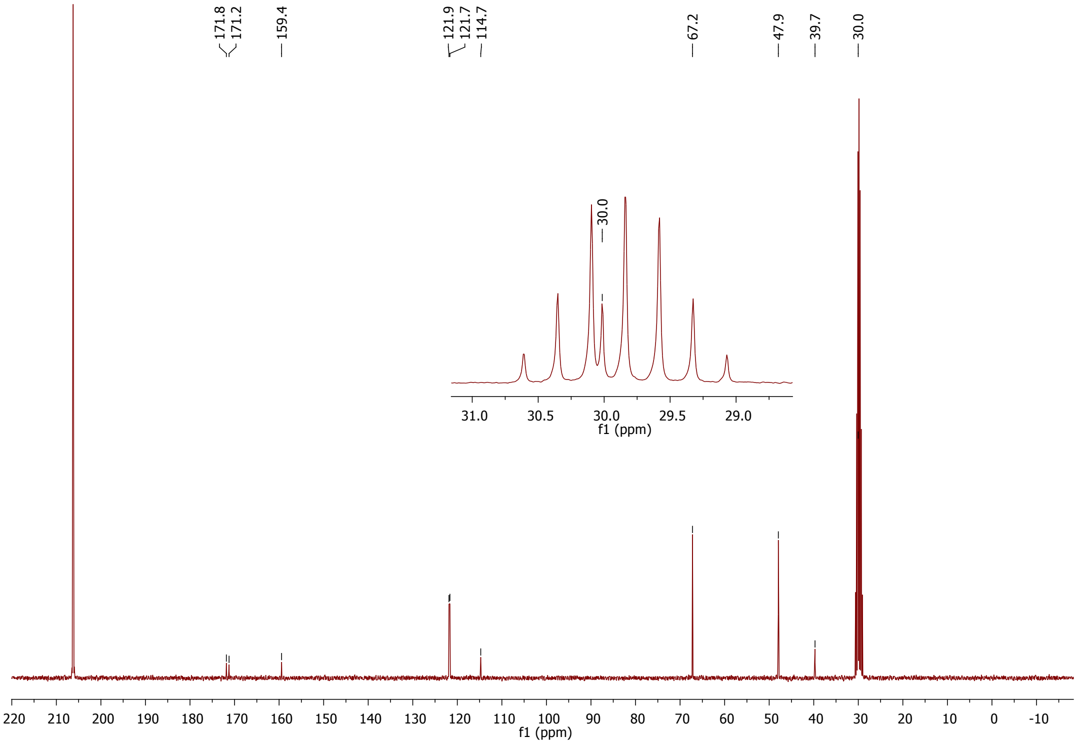

Fig. S-42: ^{13}C NMR Spectrum of **3g**

File :C:\MSDCHEM\1\DATA\2020\DR. ABBAS HASSAN\SANIA BATOOL\Snapshot
\SB-70 18-03-20.D
Operator : Saqib Yasin
Instrument : Instrument #1
Acquired : 18 Mar 2020 14:51 using AcqMethod LIQUID.M
Sample Name: SB-70
Misc Info : Temp 120-280 10C/min Flow 1.5ml/min Inj 2ul

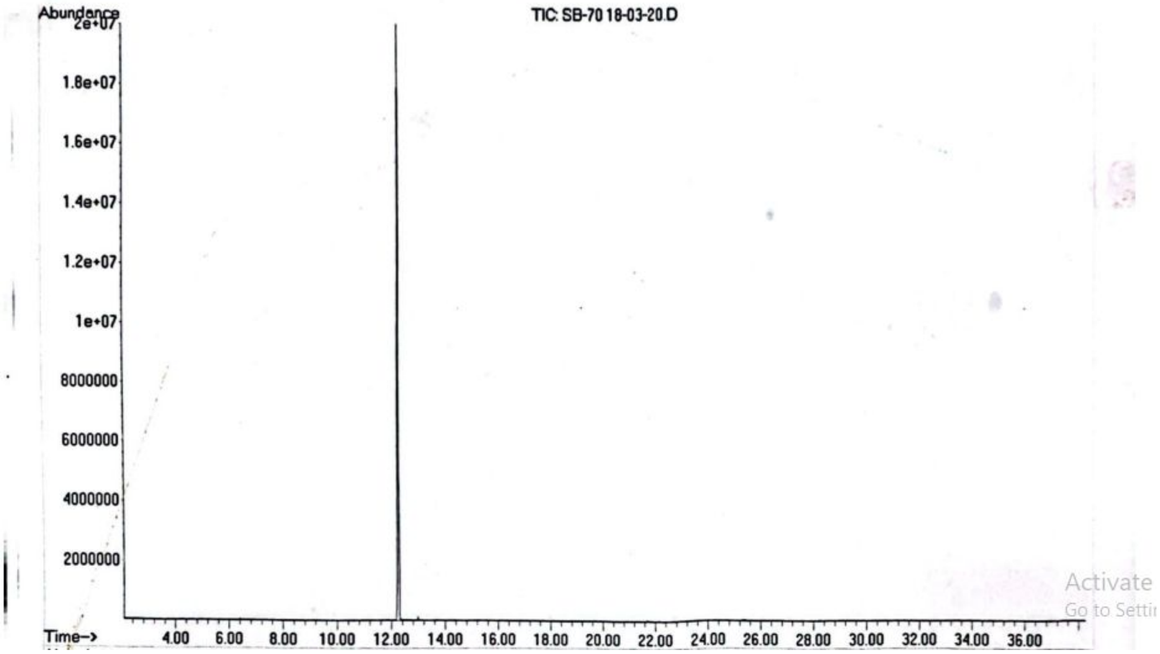

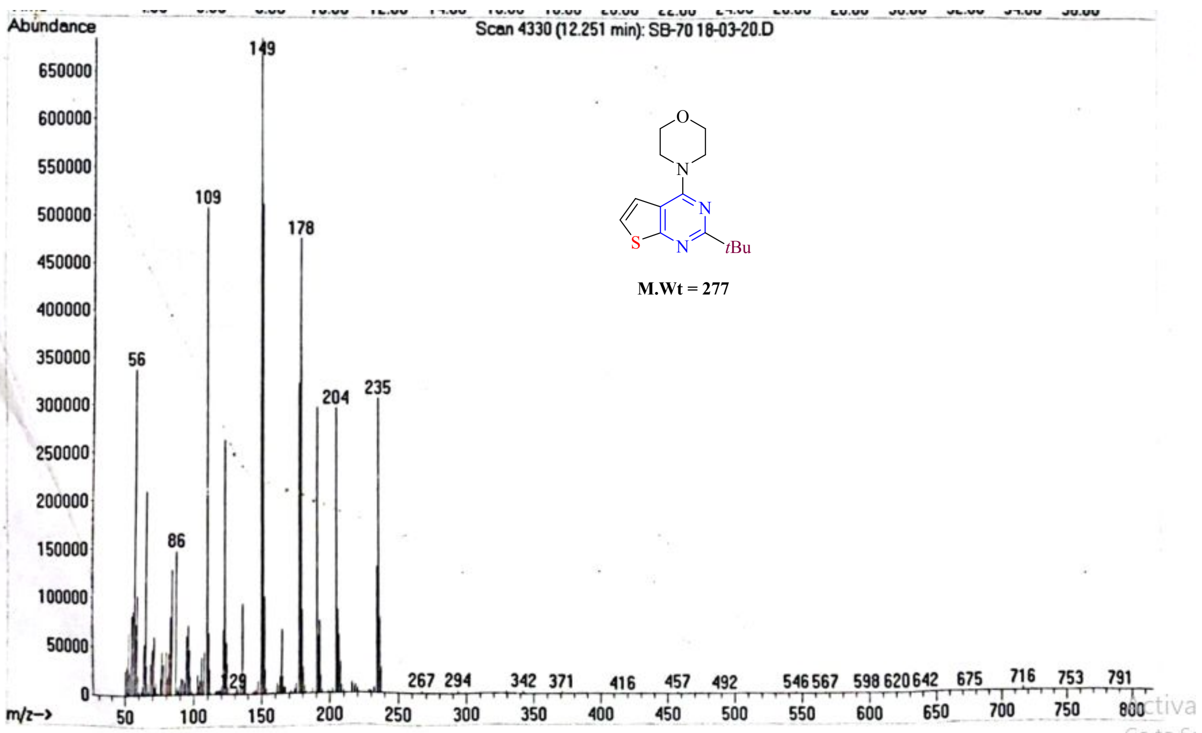

Fig. S-43: GCMS Spectrum of **3g**

2-Methyl-4-(pyrrolidin-1-yl)thieno[2,3-*d*]pyrimidine (3h)

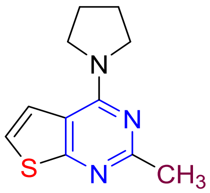

Yield: 73 %

mp = 152-155 °C

IR: (*v,* cm^{-1}) 3081, 2956-2852, 1521-1354, 1301-1051, 815-691

^{1}H NMR (300 MHz, $CDCl_3$): δ (ppm) 7.39 (d, *J* = 6.0 Hz, 1H), 7.04 (d, *J* = 6.0 Hz, 1H), 3.81-3.78 (m, 4H), 2.56 (s, 3H), 2.02 (m, 4H)

^{13}C NMR (75 MHz, $CDCl_3$): δ (ppm) 170.4, 162.4, 158.8, 121.0, 120.2, 114.1, 66.9, 47.2, 25.9

GC-MS Analysis (m/z): M^+ = 219, 190, 149, 122, 70

HRMS-ESI (m/z): $[M+H]^+$ calc'd for $C_{11}H_{14}N_3S^+$, 220.0921; found, 220.0923

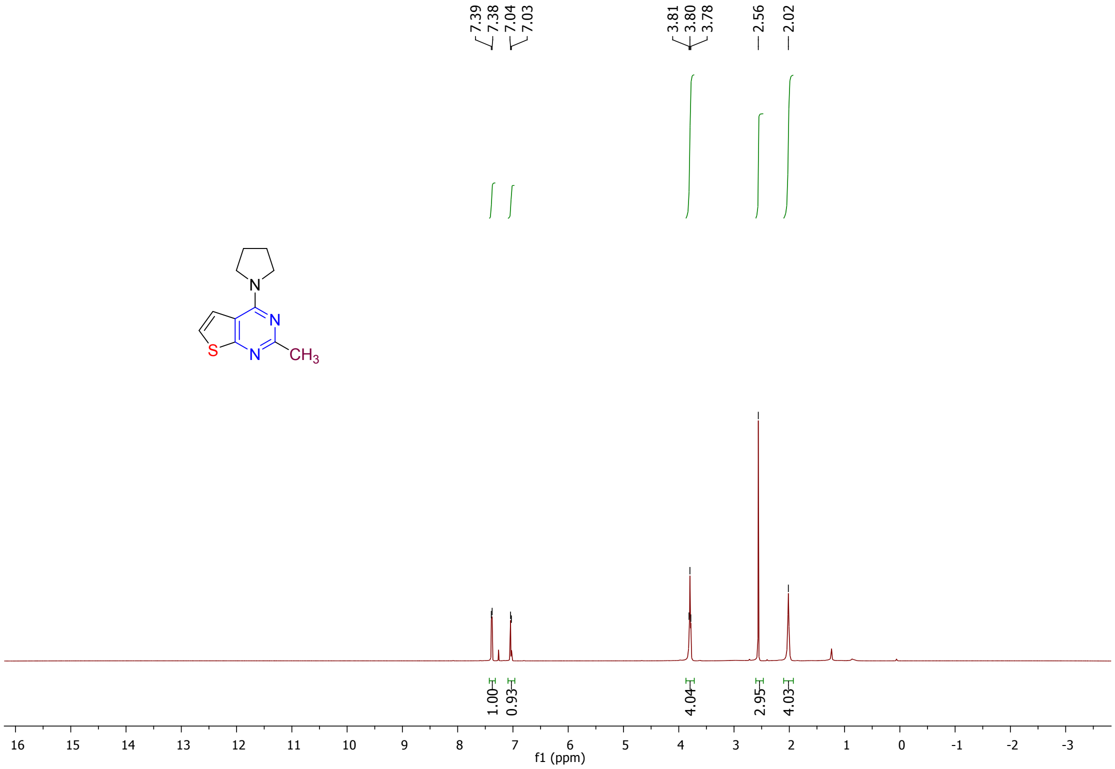

Fig. S-44: ^{1}HNMR Spectrum of **3h**

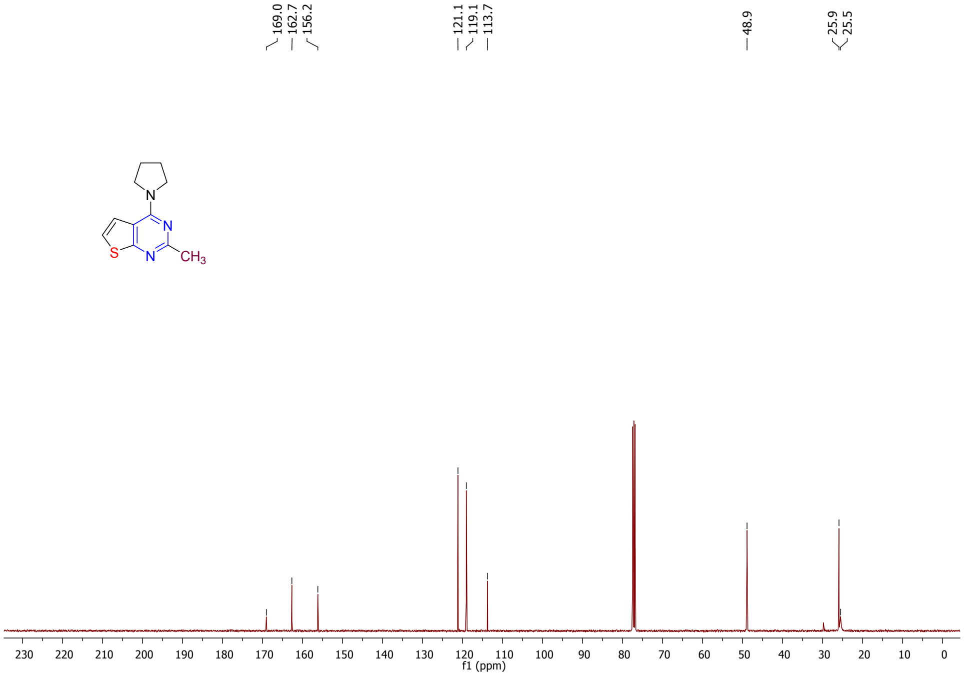

Fig. S-45: ^{13}C NMR Spectrum of **3h**

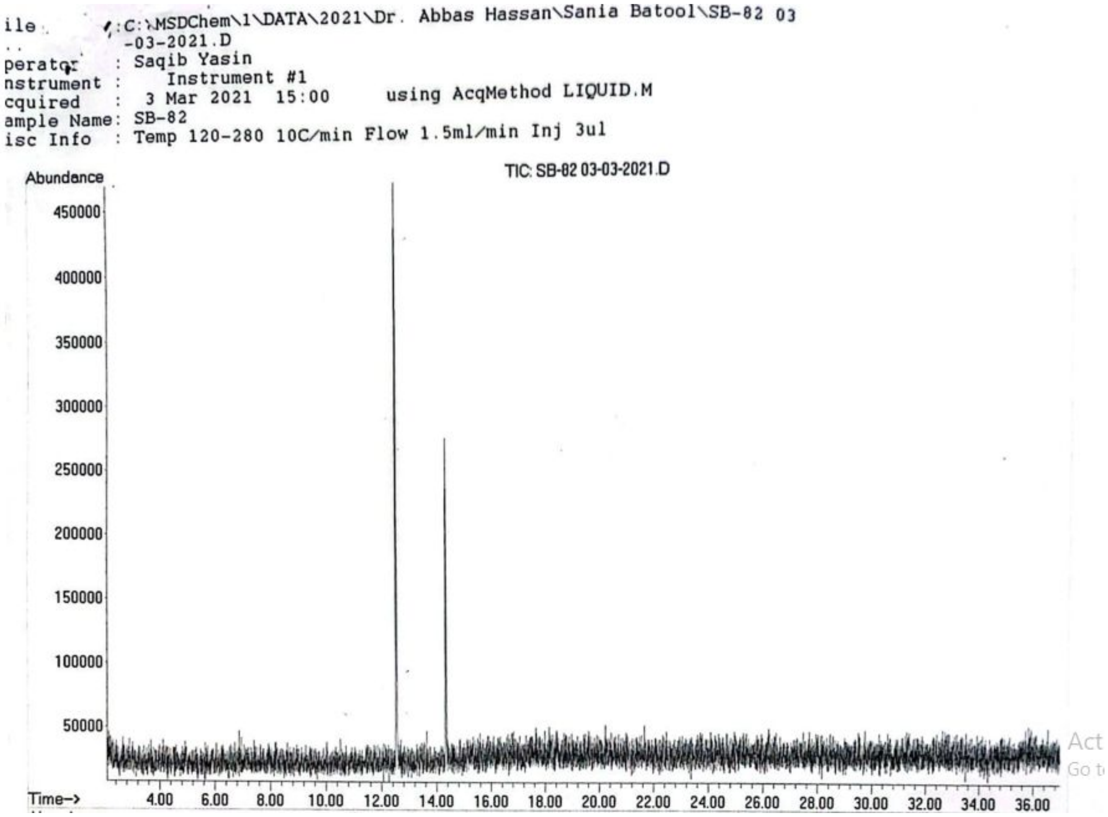

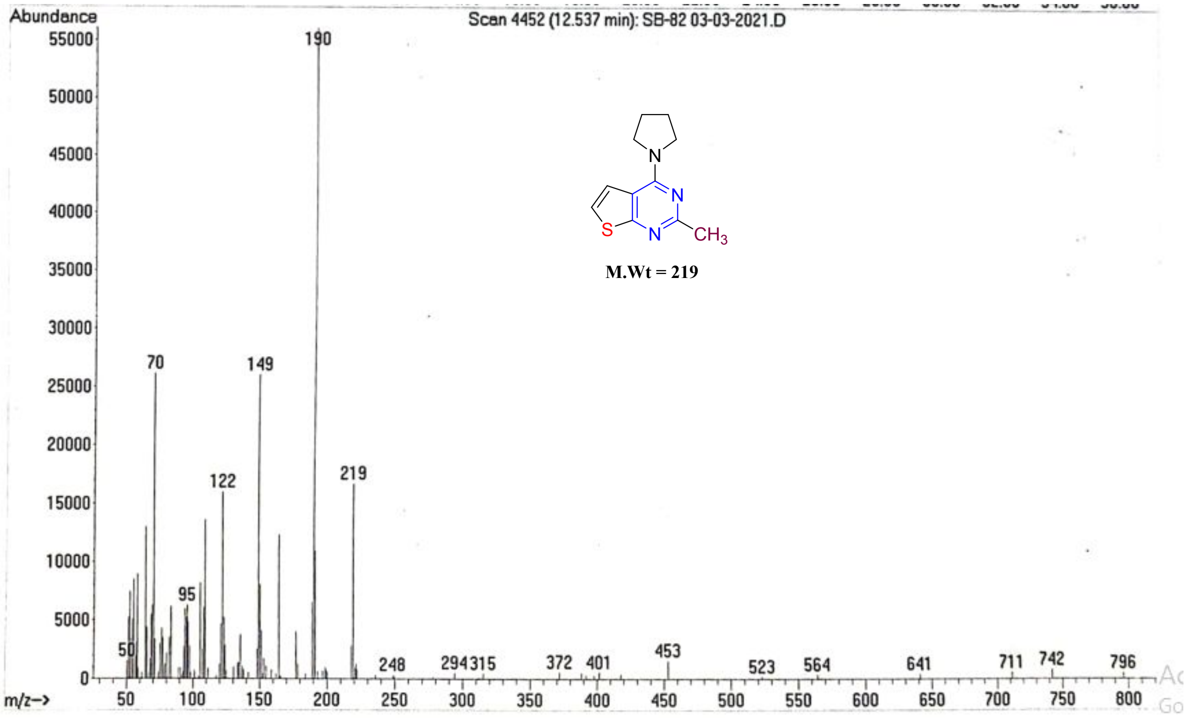

Fig. S-46: GCMS Spectrum of **3h**

2-(*tert*-Butyl)-4-(pyrrolidin-1-yl)thieno[2,3-*d*]pyrimidine (3i)

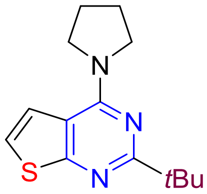

Yield: 71%

mp = 142-145 °C

IR: (*v*, cm^{-1}) 3071, 2944-2851, 1531-1353, 1263-1048, 813-631

^{1}H NMR (300 MHz, $CDCl_3$): δ (ppm) 7.41-7.39 (d, *J* = 6.0 Hz, 1H), 7.07-7.04 (d, *J* = 9.0 Hz, 1H), 3.85-3.80 (m, 4H), 2.05-2.01 (m, 4H), 1.40 (s, 9H)

^{13}C NMR (75 MHz, $CDCl_3$): δ (ppm) 172.1, 169.7, 155.9, 120.9, 119.1, 113.6, 98.7, 39.2, 29.9, 25.6

GC-MS Analysis (m/z): M^+ = 261, 233, 191, 135, 109, 70

HRMS-ESI (m/z): $[M+H]^+$ calc'd for $C_{14}H_{20}N_3S^+$, 262.1312; found, 262.1315

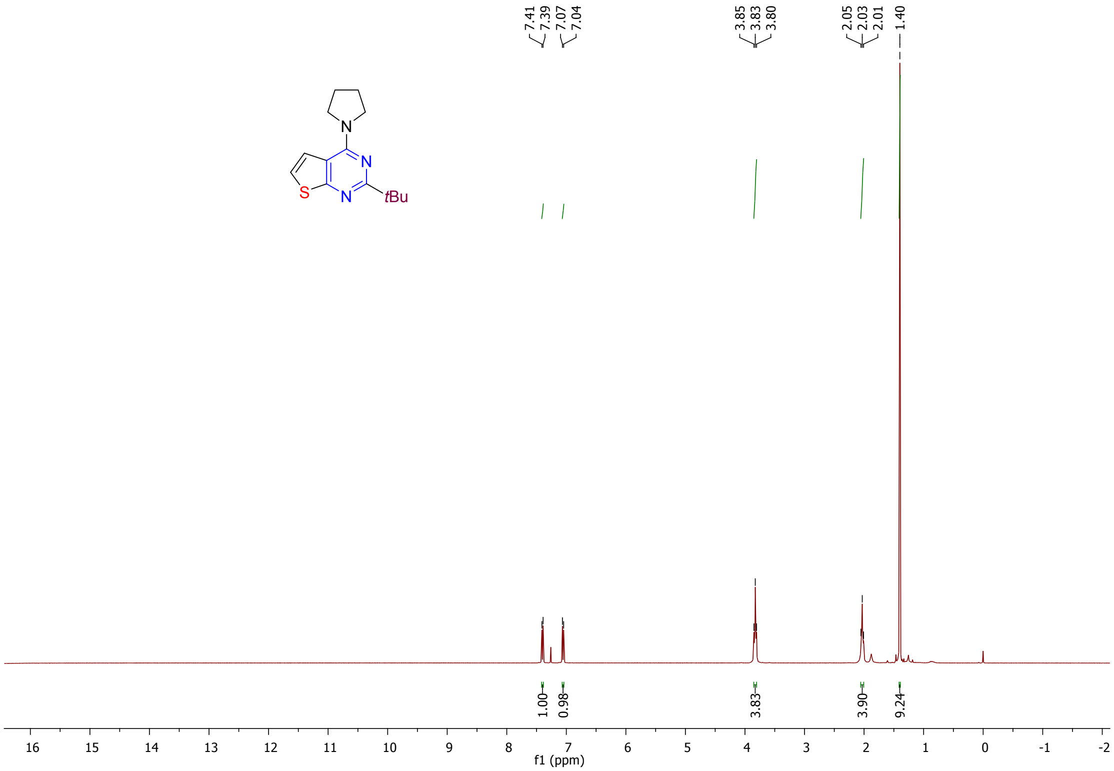

Fig. S-47: ^{1}HNMR Spectrum of **3i**

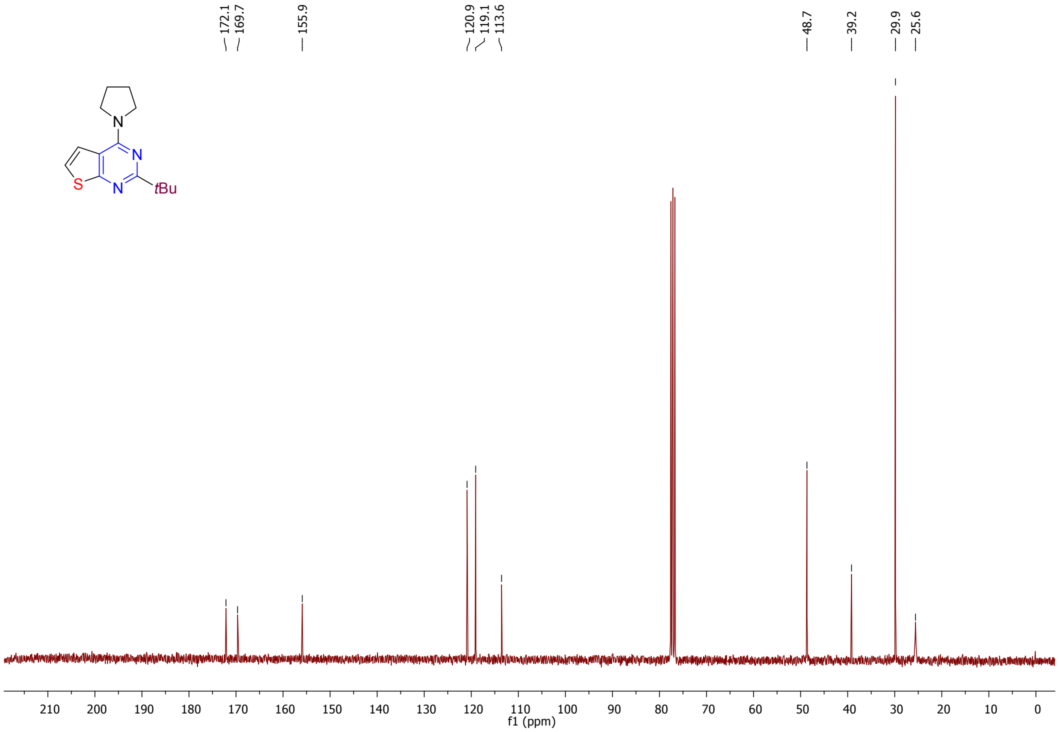

Fig. S-48: ^{13}C NMR Spectrum of **3i**

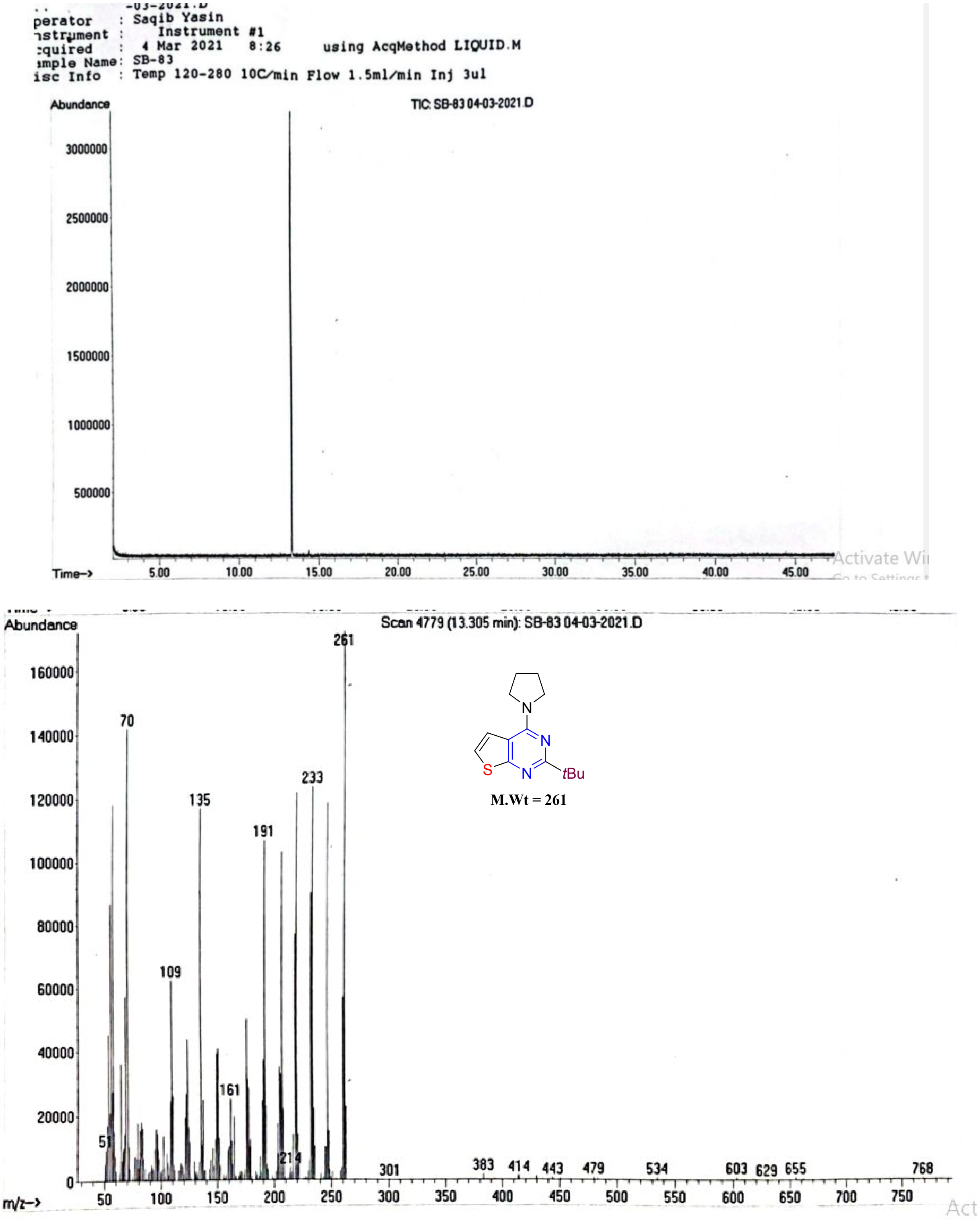

Fig. S-49: GCMS Spectrum of **3i**

2-((2-(*tert*-Butyl)thieno[2,3-*d*]pyrimidin-4-yl)amino)acetamide (3j)

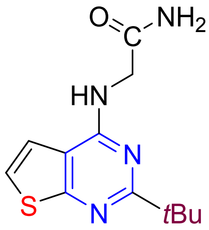

Yield: 74%

mp = 180-184 °C

IR: (*v,* cm^{-1}) 3447, 3319, 3071, 2944-2817, 1664, 1531-1353, 1261-1081, 814-637

^{1}H NMR (300 MHz, DMSO d_6): δ (ppm) 8.15-8.11 (*ap.*t, *J* = 6.0 Hz, 1H), 7.57-7.55 (d, *J* = 6.0 Hz, 1H), 7.47-7.45 (d, *J* = 6.0 Hz, 1H), 7.43 (s, 1H), 7.03 (s, 1H), 4.02-4.00 (d, *J* = 6.0 Hz, 2H), 1.31 (s, 9H)

^{13}C NMR (75 MHz, DMSO d_6): δ (ppm) 171.6, 171.5, 166.4, 156.7, 121.6, 119.3, 114.0, 43.2, 38.9, 29.6

GC-MS Analysis (m/z): M^+ = 264, 248, 276, 220, 211, 74, 57

HRMS-ESI (m/z): $[M+H]^+$ calc'd for $C_{12}H_{17}N_4OS^+$, 265.1067; found, 265.1071

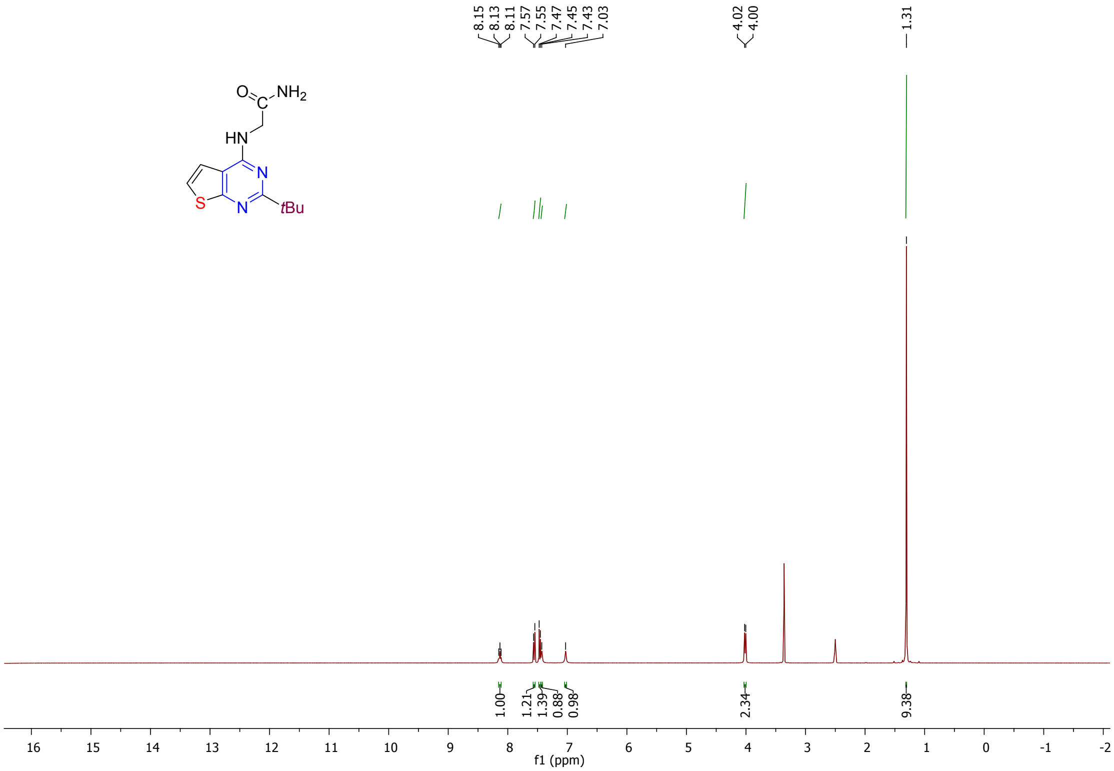

Fig. S-50: ^{1}HNMR Spectrum of **3j**

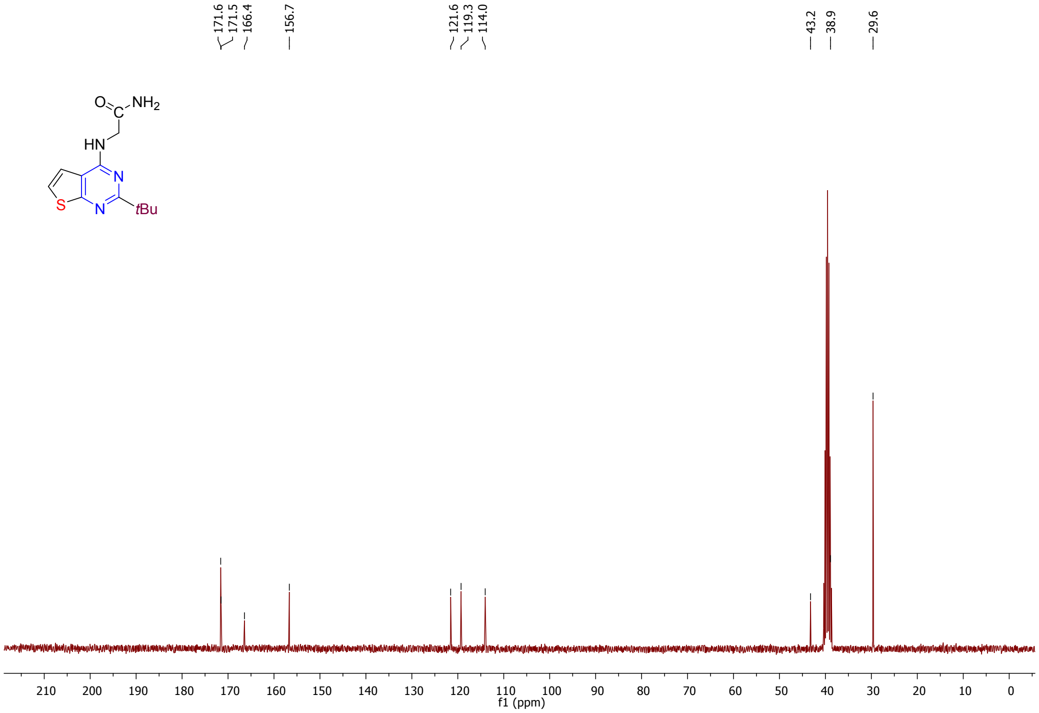

Fig. S-51: ^{13}C NMR Spectrum of **3j**

2-Phenyl-4-(phenylthiol)thieno[2,3-*d*]pyrimidine (4a)

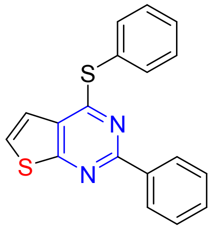

Yield: 75%

mp = 134-137 °C

IR: (*v,* cm^{-1}) 3096-3059, 2920-2850, 1524-1398, 734-670

^{1}H NMR (300 MHz, $CDCl_3$): δ (ppm) 8.22-8.19 (m, 2H), 7.73-7.70 (m, 2H) 7.56-7.50 (m, 3H), 7.47-7.45 (d, J = 6.0 Hz, 1H), 7.41-7.33 (m, 4H)

^{13}C NMR (75 MHz, $CDCl_3$): δ (ppm) 167.8, 163.9, 158.8, 137.3, 135.9, 130.5, 129.7, 129.3, 128.5, 128.4, 127.7, 126.2, 125.5, 119.1

GC-MS Analysis (m/z): M^+ = 320, M^{+1} = 321, M^{+2} = 322, 319, 211, 109, 77, 65, 51

HRMS-ESI (m/z): $[M+H]^+$ calc'd for $C_{18}H_{13}N_2S_2^+$, 321.0543; found, 321.0546

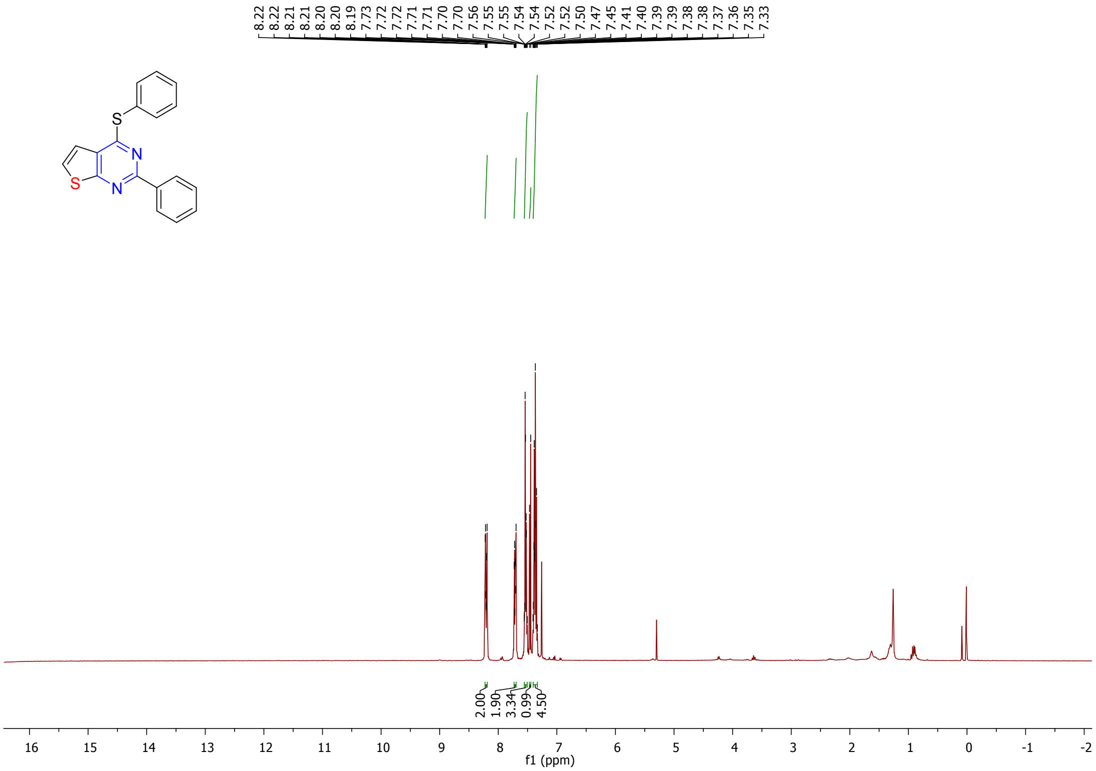

Fig. S-52: ^{1}HNMR Spectrum of **4a**

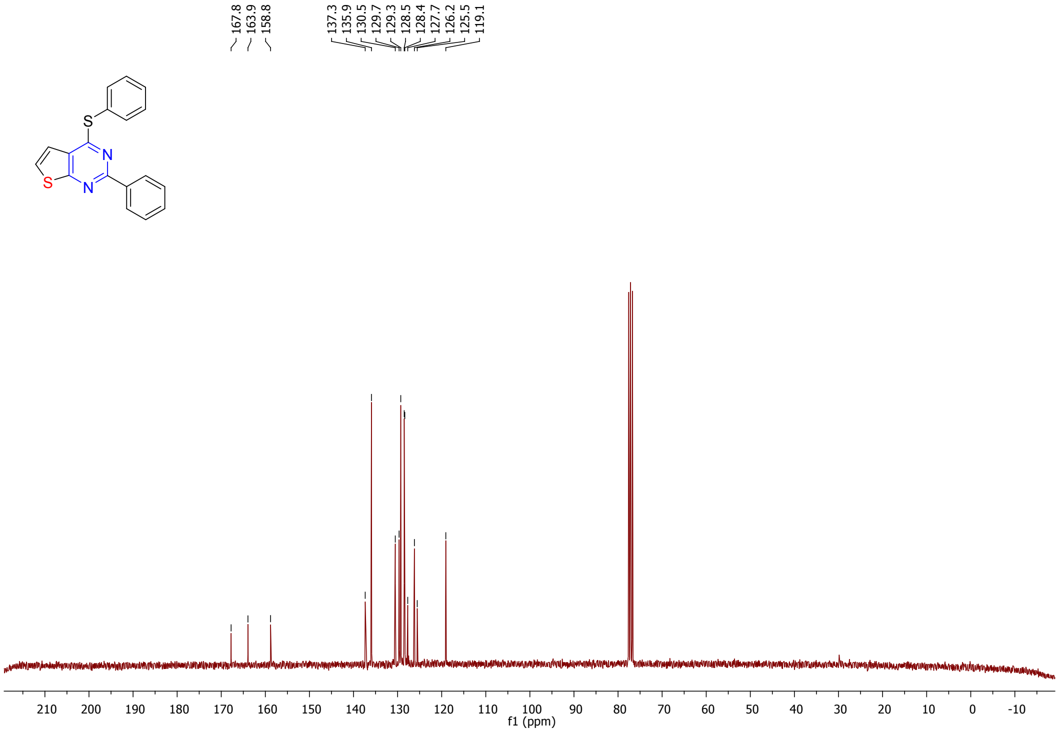

Fig. S-53: ^{13}C NMR Spectrum of **4a**

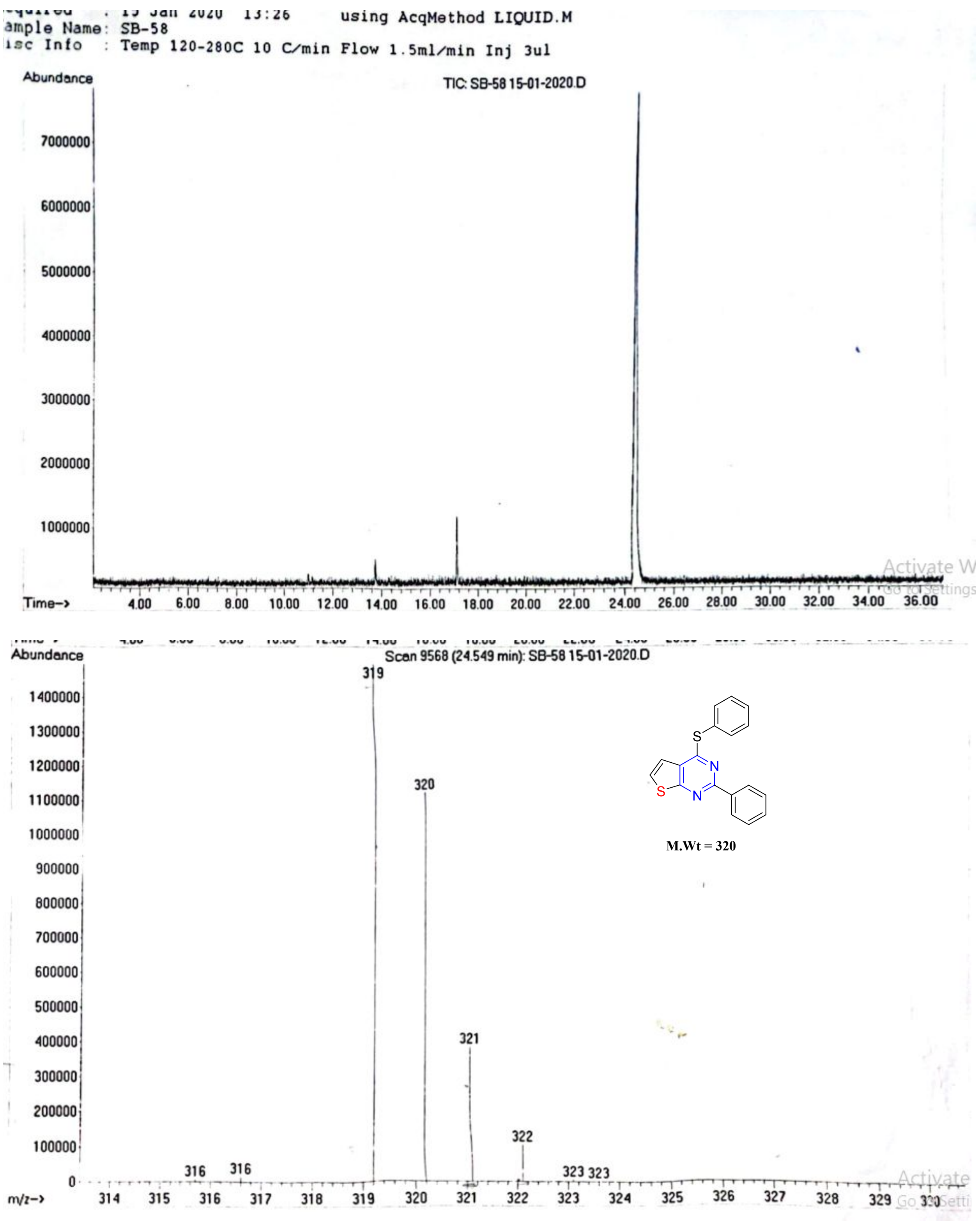

Fig. S-54: GCMS Spectrum of **4a**

2-Phenyl-4-(pyridin-2-ylthio)thieno[2,3-*d*]pyrimidine (4b)

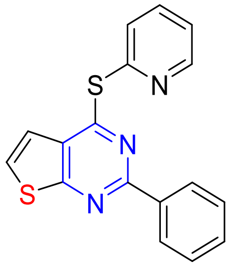

Yield: 66%

mp = 142-145 ˚C

IR: (*v,* cm^{-1}) 3094-3056, 2925-2854, 1521-1395, 732-673

^{1}H NMR (300 MHz, $CDCl_3$): δ (ppm) 8.70 (*ap.* d, *J* = 6.0, 1H), 8.24-8.21 (m, 2H), 7.92-7.80 (2H, m), 7.48 (*ap.* d, *J* = 6.0, 1H), 7.40-7.36 (m, 1H), 7.51-7.37 (m, 4H)

^{13}C NMR (75 MHz, $CDCl_3$): δ (ppm) 168.2, 162.2, 159.0, 152.4, 150.4, 137.3, 137.2, 130.6, 128.6, 128.4, 126.7, 126.4, 123.5, 119.3

GC-MS Analysis (m/z): M^{+} = 321, M^{+1} = 322, M^{+2} = 323, 211, 77, 51

HRMS-ESI (m/z): $[M+H]^{+}$ calc'd for $C_{17}H_{12}N_3S_2^{+}$,322.0417; found, 322.0422

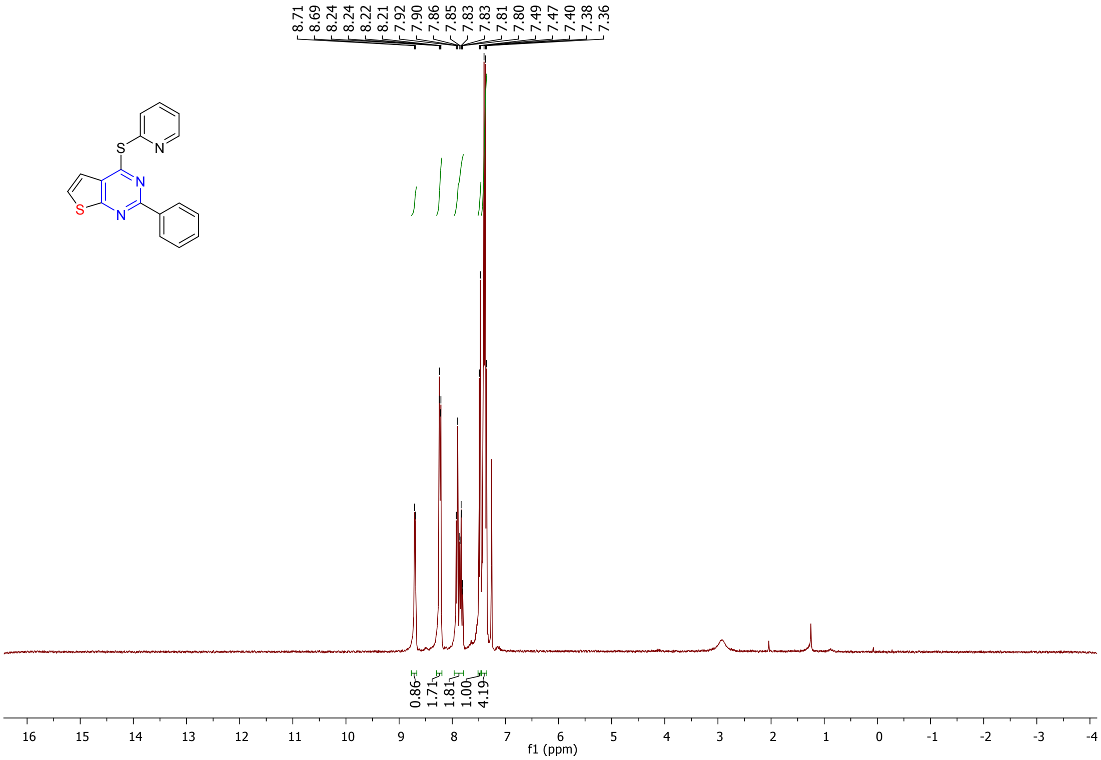

Fig. S-55: ^{1}HNMR Spectrum of **4b**

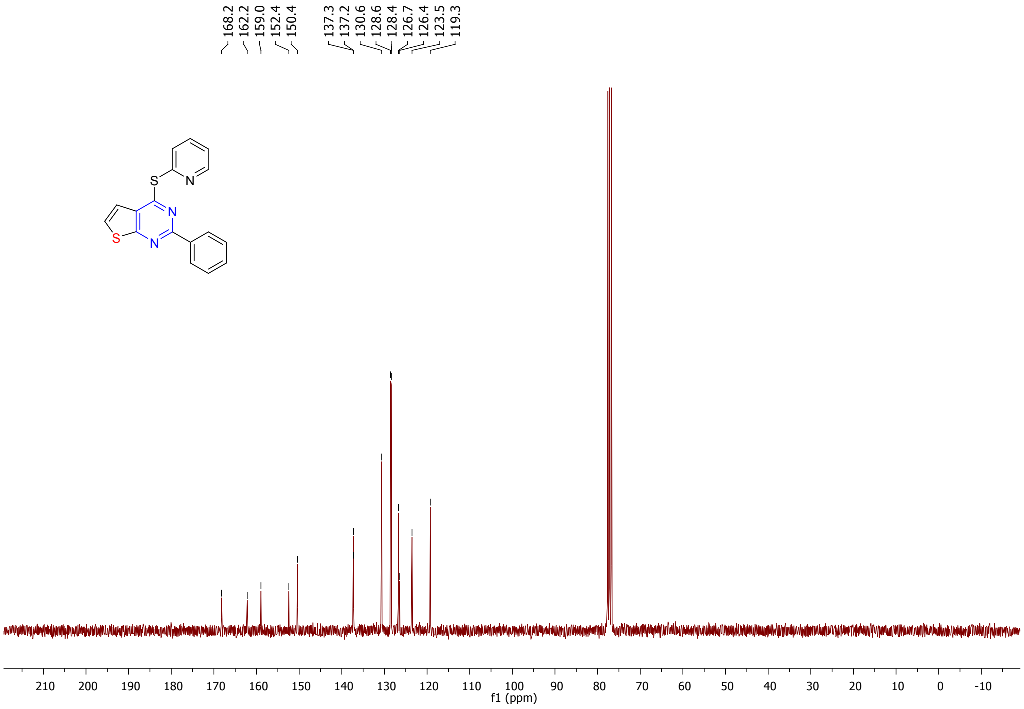

Fig. S-56: ^{13}C NMR Spectrum of **4b**

.. -03-20.D
perator : Saqib Yasin
nstrument : Instrument #1
cquired : 18 Mar 2020 16:08 using AcqMethod LIQUID.M
ample Name: SB-75
isc Info : Temp 120-280 10C/min Flow 1.5ml/min Inj 2ul

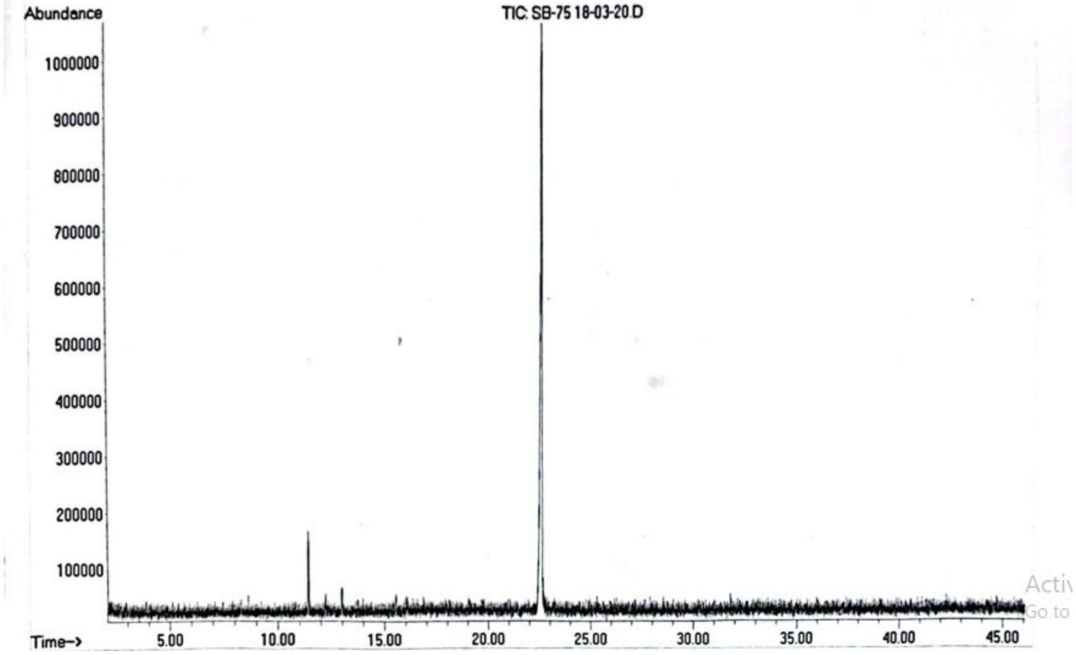

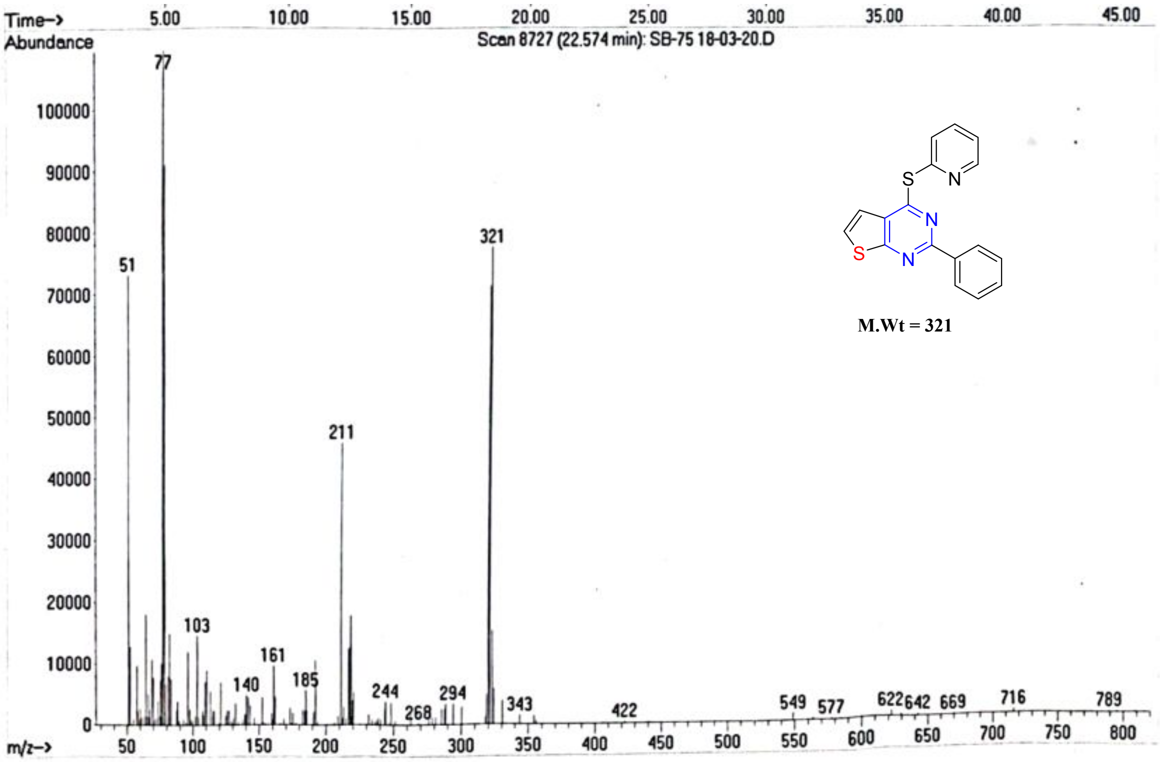

Fig. S-57: GCMS Spectrum of **4b**

4-(Benzo[d]thiazol-2-ylthio)-2-methylthieno[2,3-*d*]pyrimidine (4c)

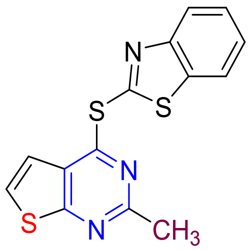

Yield: 78%

mp = 202-206 °C

IR: (*v,* cm^{-1}) 3094-3053, 2919-2843, 1517-1385, 724-688

^{1}H NMR (300 MHz, $CDCl_3$): δ (ppm) 8.02-8.00 (d, *J* = 6.0 Hz, 1H), 7.93-7.90 (d, *J* = 9.0 Hz, 1H), 7.51-7.47 (m, 2H), 7.43-7.38 (m, 1H), 7.34-7.32 (d, *J* = 6.0 Hz, 1H), 2.87 (s, 3H)

^{13}C NMR (75 MHz, $CDCl_3$): δ (ppm) 168.0, 162.2, 159.0, 158.1, 151.4, 136.2, 126.7, 126.4, 125.3, 125.2, 122.7, 121.1, 118.5, 25.5

GC-MS Analysis (m/z): M^+ = 315, M^{+1} = 316, M^{+2} = 317, 149, 122, 90, 64

HRMS-ESI (m/z): $[M+H]^+$ calc'd for $C_{14}H_9N_3S_3^+$, 315.021; found, 315.026

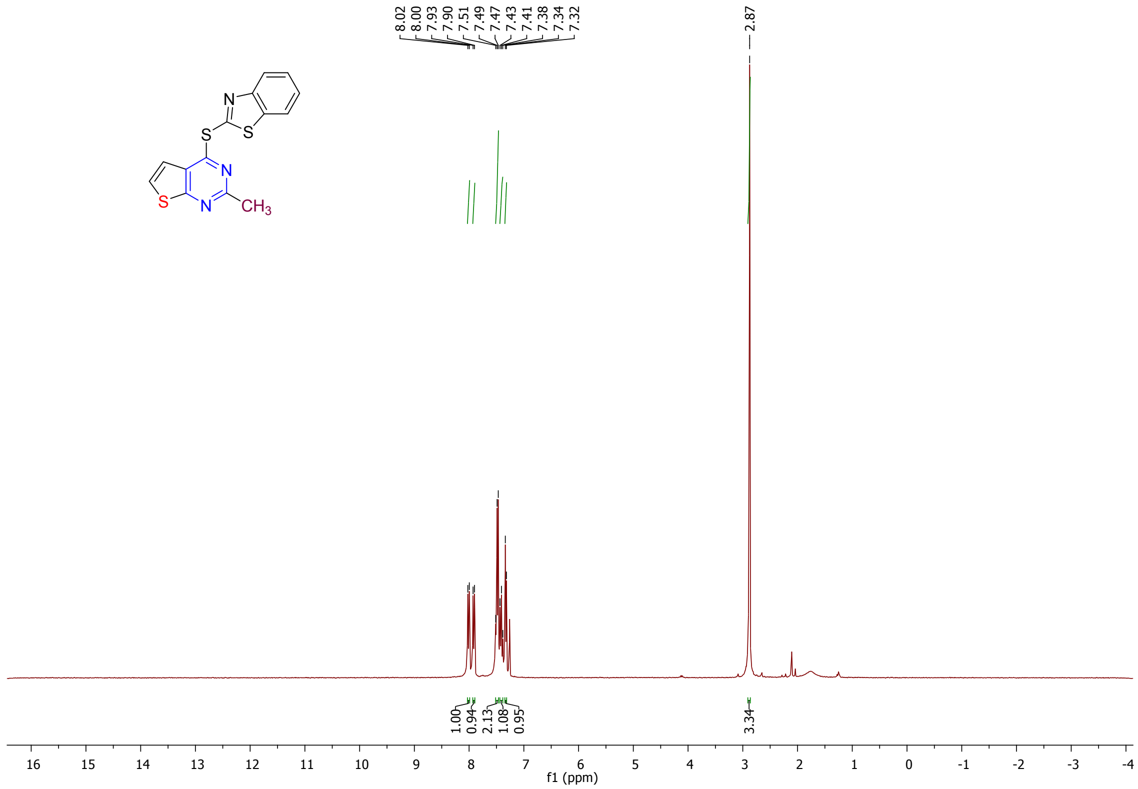

Fig. S-58: ^{1}HNMR Spectrum of **4c**

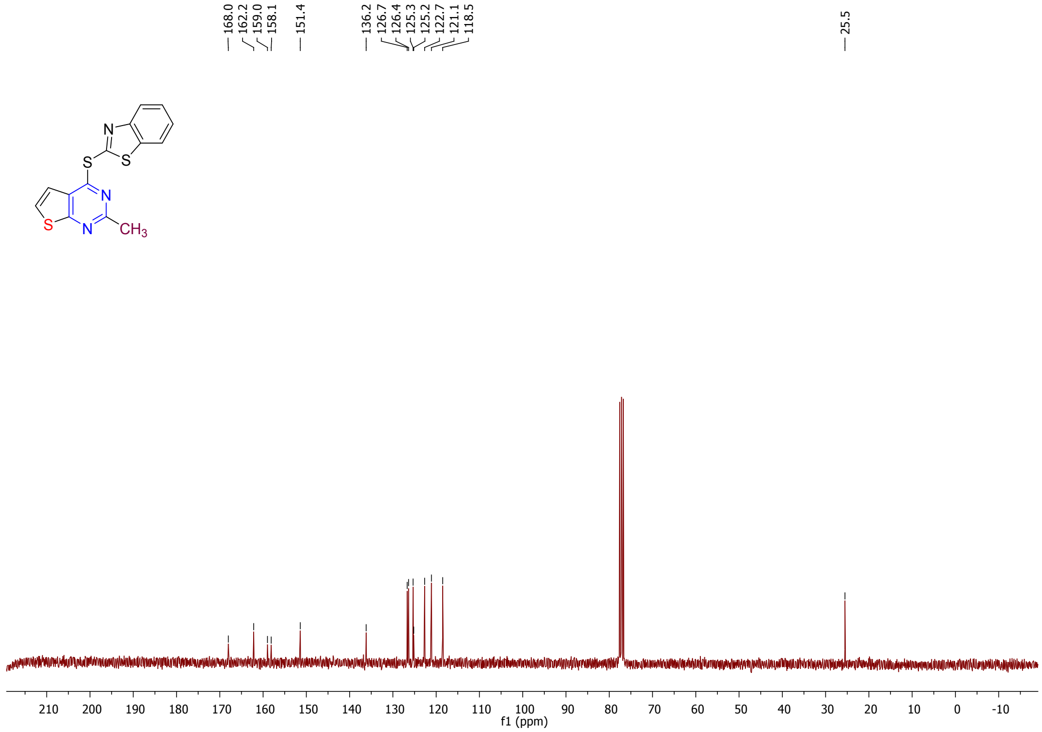

Fig. S-59: ^{13}C NMR Spectrum of **4c**

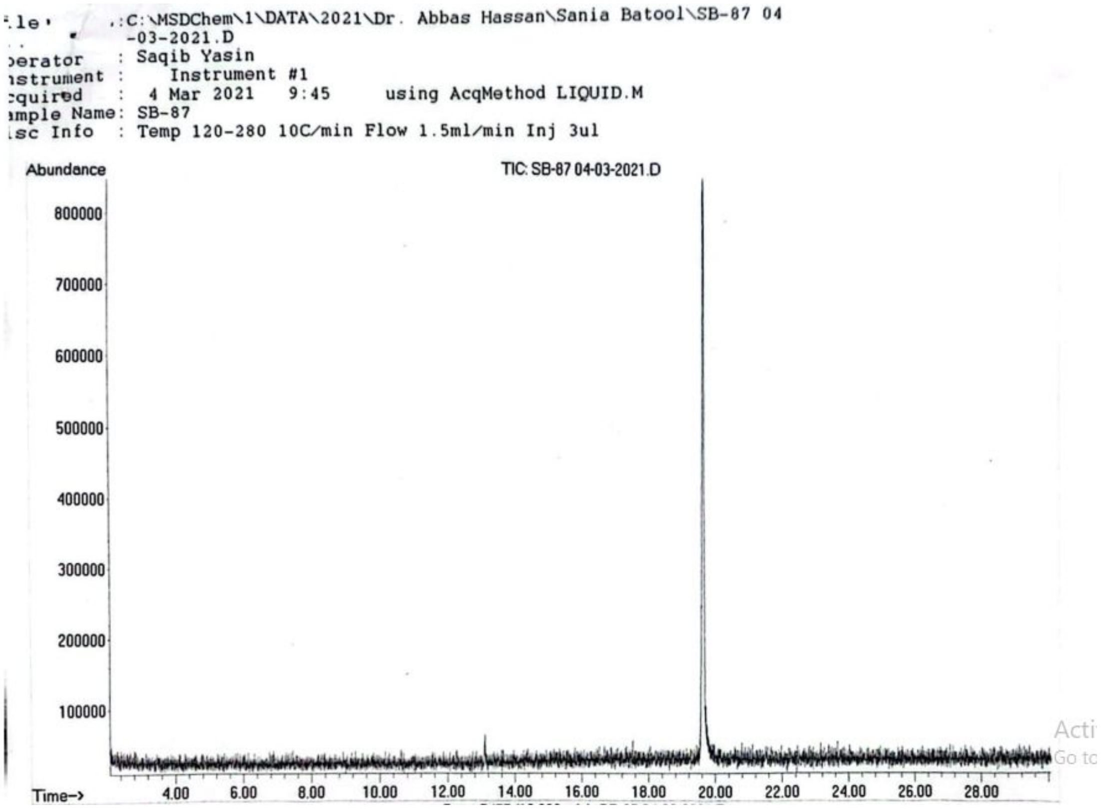

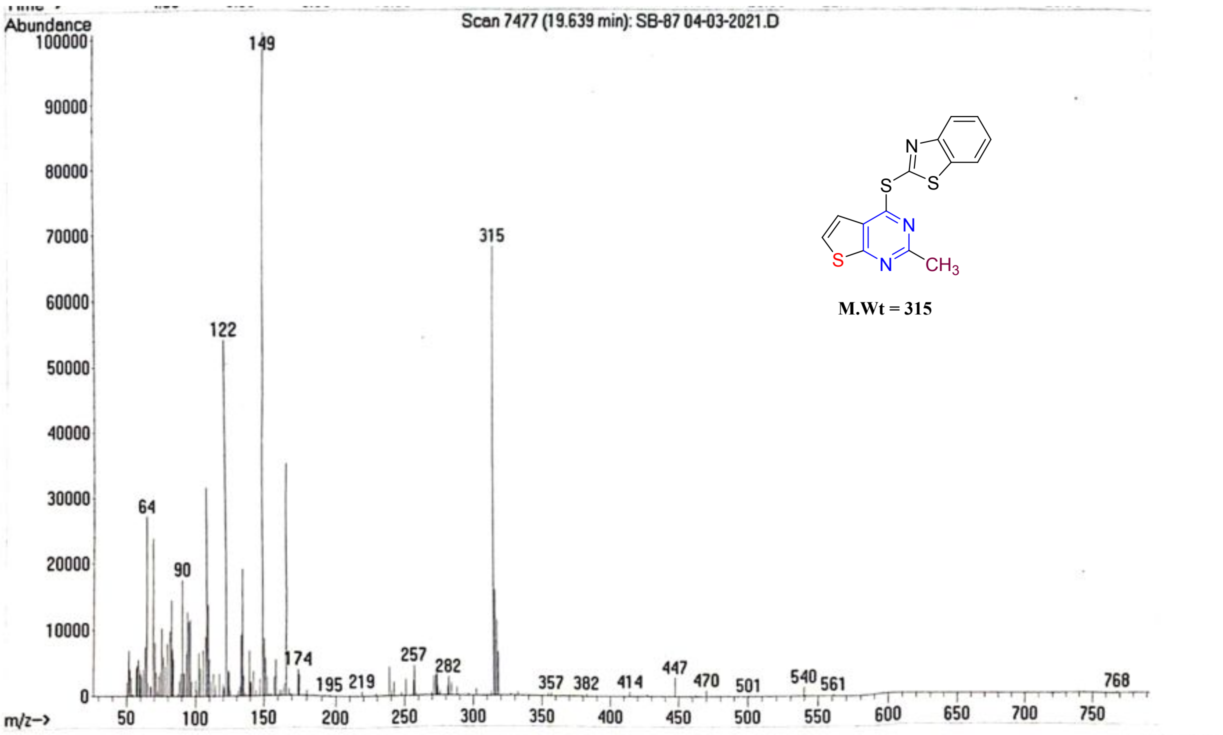

Fig. S-60: GCMS Spectrum of **4c**

2-(Tert-butyl)-4-(pyridin-2-ylthio)thieno[2,3-*d*]pyrimidine (4d)

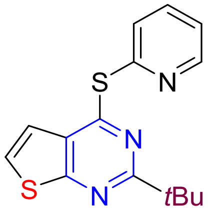

Yield: 68%

mp = 155-158 ˚C

IR: (*v,* cm^{-1}) 3087-3053, 2967-2834, 1518-1392, 731-689

^{1}H NMR (300 MHz, $CDCl_3$): δ (ppm) 8.65-8.63 (*ap*. d, *J* = 6.0 Hz, 1H), 7.87-7.85 (*ap*. d, *J* = 6.0 Hz, 1H), 7.79-7.73 (*ap*. td, *J* = 12.0 Hz, 3.0 Hz, 1H), 7.43-7.41 (d, *J* = 6.0, 1H), 7.34-7.30 (m, 2H), 1.28 (s, 9H), 1.26 (s, 9H)

^{13}C NMR (75 MHz, $CDCl_3$): δ (ppm) 171.5, 167.4, 161.4, 152.4, 150.1, 136.8, 130.6, 125.6, 125.3, 123.2, 118.7, 39.6, 29.5

GC-MS Analysis (m/z): M^+ = 301, M^{+1} = 302, M^{+2} = 303, 286, 191, 135, 78, 51

HRMS-ESI (m/z): $[M+H]^+$ calc'd for $C_{15}H_{16}N_3S_2^+$, 302.0794; found, 302.0798

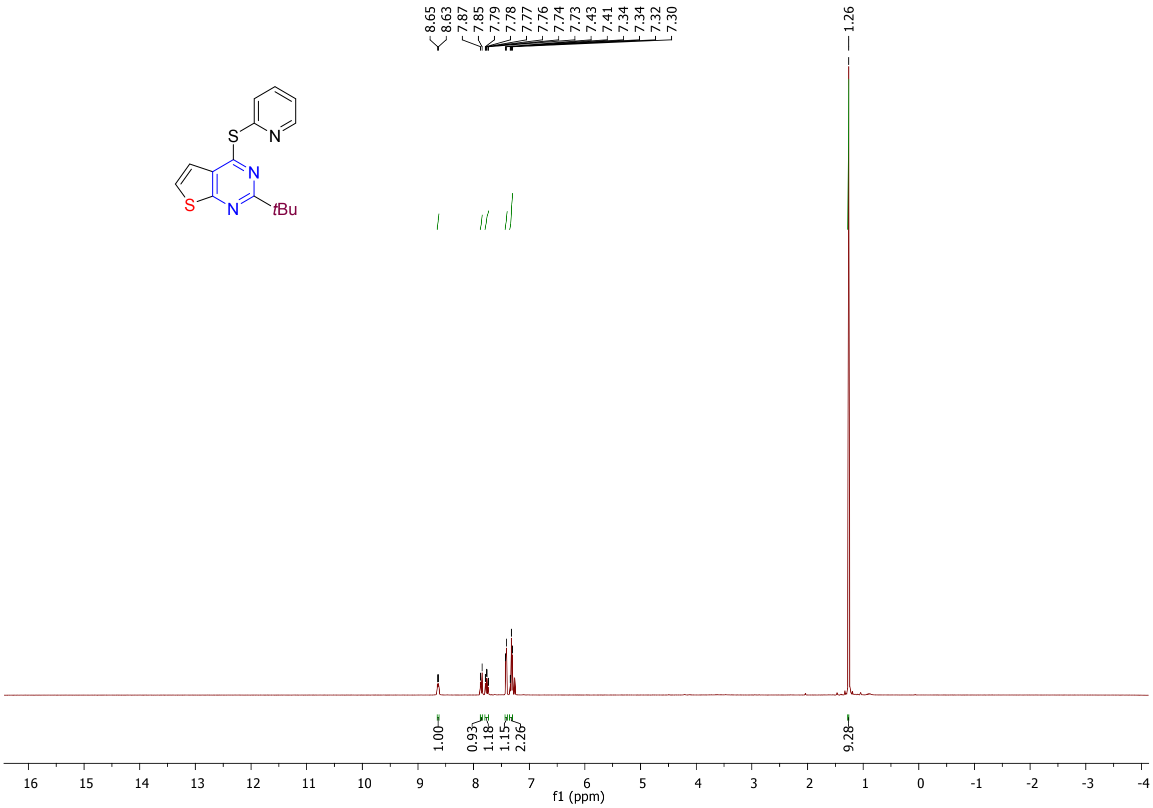

Fig. S-61: ^{1}HNMR Spectrum of **4d**

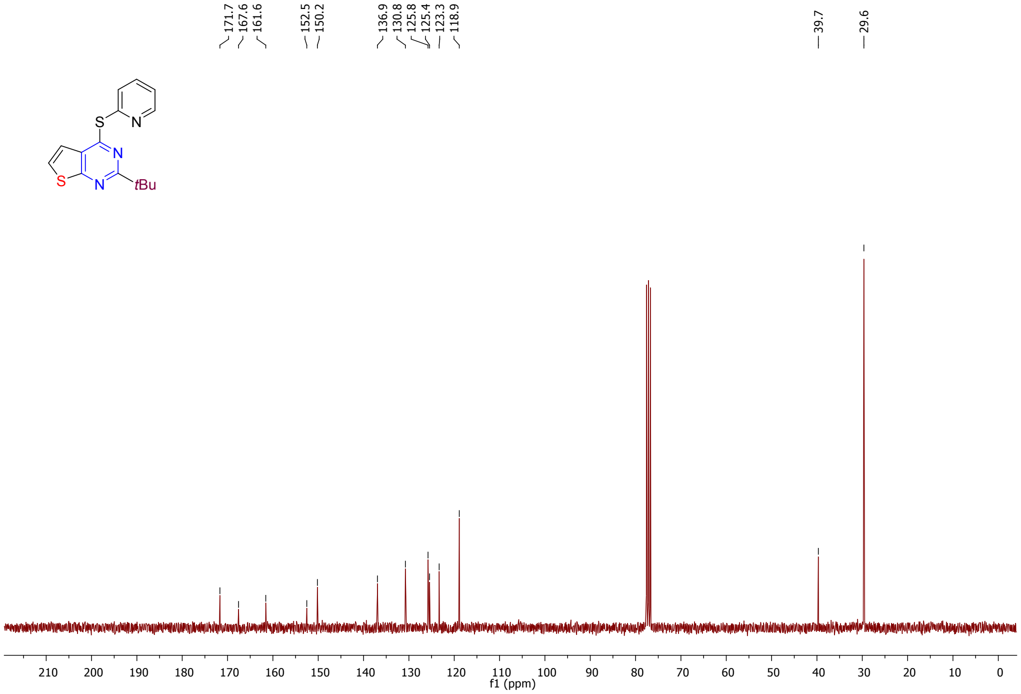

Fig. S-62: ^{13}C NMR Spectrum of **4d**

```
le        :C:\MSDChem\1\DATA\2021\Dr. Abbas Hassan\Sania Batool\SB-91 04
.          -03-2021.D
erator    : Saqib Yasin
strument  :    Instrument #1
quired    :  4 Mar 2021   10:50     using AcqMethod LIQUID.M
mple Name: SB-91
sc Info   : Temp 120-280 10C/min Flow 1.5ml/min Inj 3ul
```

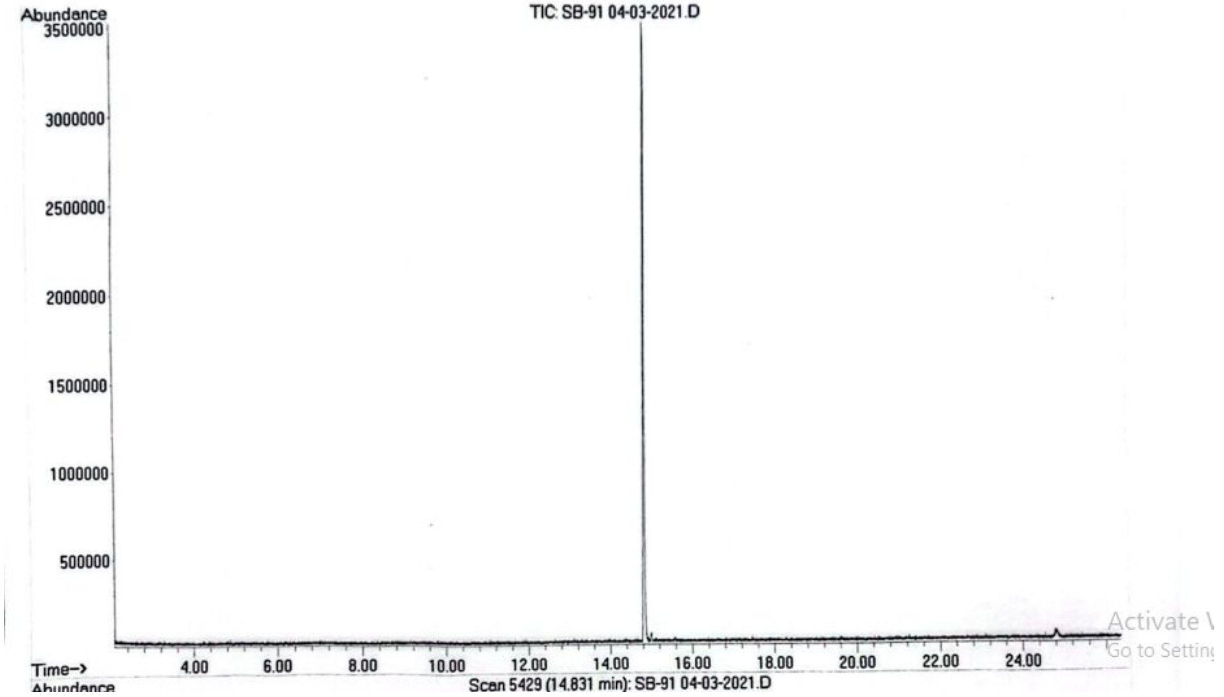

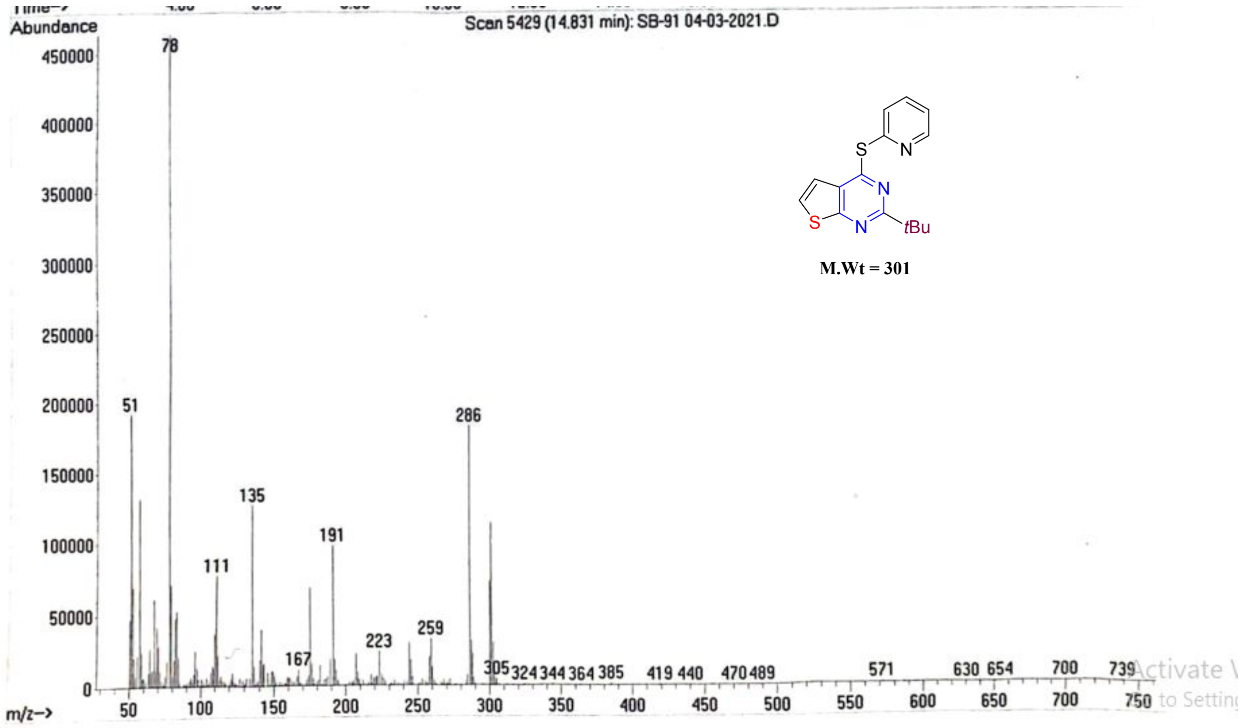

Fig. S-63: GCMS Spectrum of **4d**

2,4-Diphenylthieno[2,3-*d*]pyrimidine (5a)

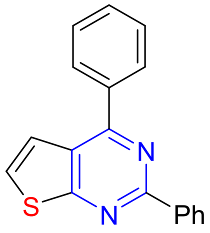

Yield: 81%

mp = 166-169 ˚C

IR: (*v,* cm^{-1}) 3090-3043, 1593-1367, 1339-1187, 735-696

^{1}H NMR [18] (300 MHz, $CDCl_3$): δ (ppm) 8.68-8.64 (m, 2H), 8.09-8.05 (m, 2H), 7.62-7.49 (m, 8H)

^{13}C NMR [18] (75 MHz, $CDCl_3$): δ (ppm) 171.1, 160.7, 159.8, 138.3, 137.9, 130.6, 130.4, 129.4, 128.9, 128.7, 128.6, 126.6, 126.0, 121.1

GC-MS Analysis (m/z): M^+ = 288, 185, 140, 103, 77, 51

HRMS-ESI (m/z): $[M+H]^+$ calc'd for $C_{18}H_{13}N_2S^+$, 289.0831; found, 289.0833

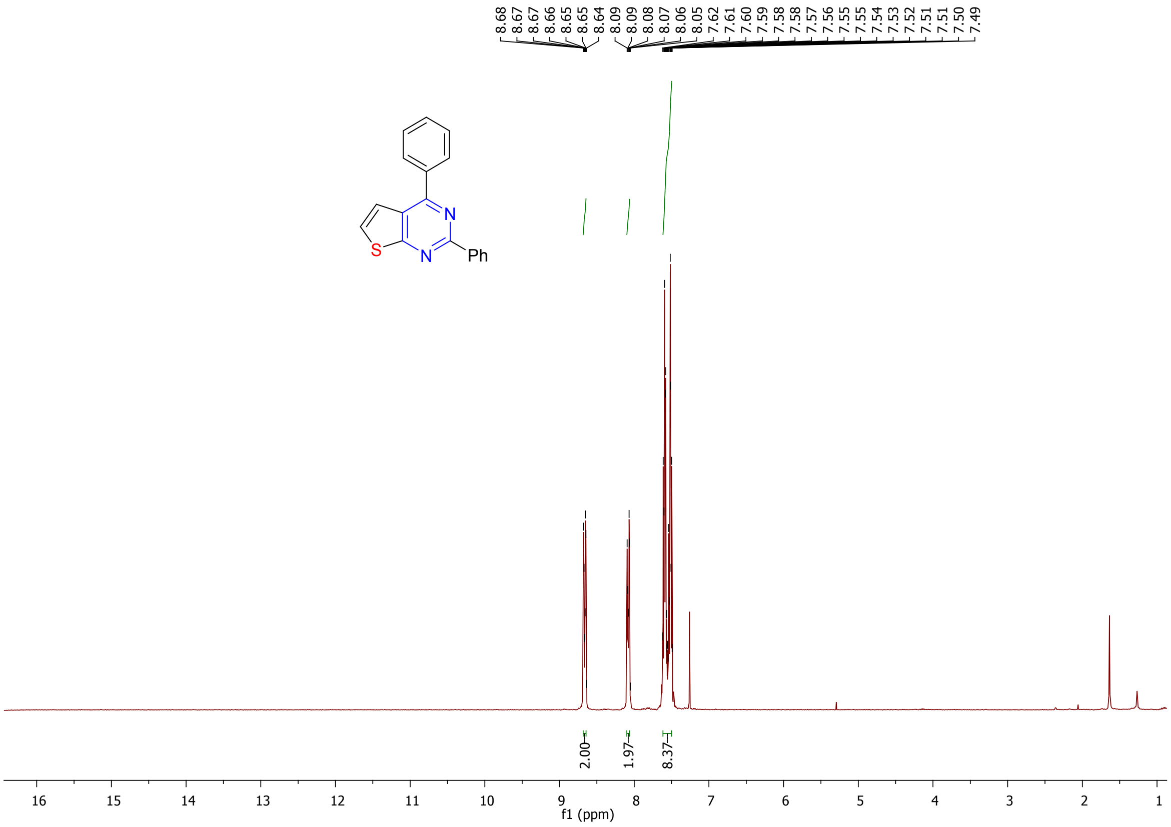

Fig. S-64: ^{1}HNMR Spectrum of **5a**

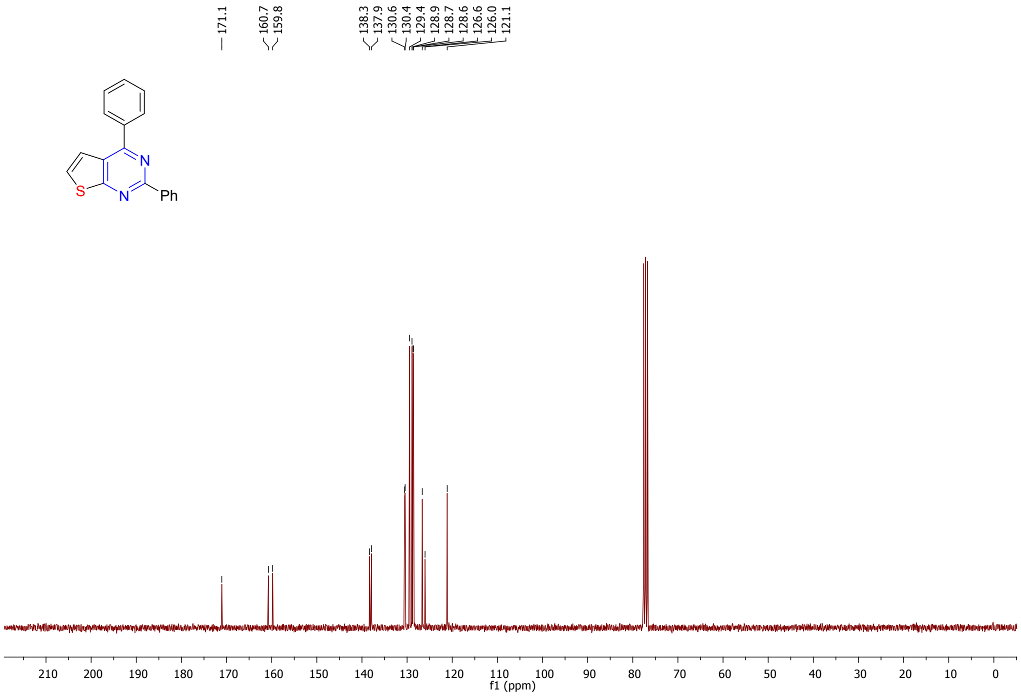

Fig. S-65: ^{13}C NMR Spectrum of **5a**

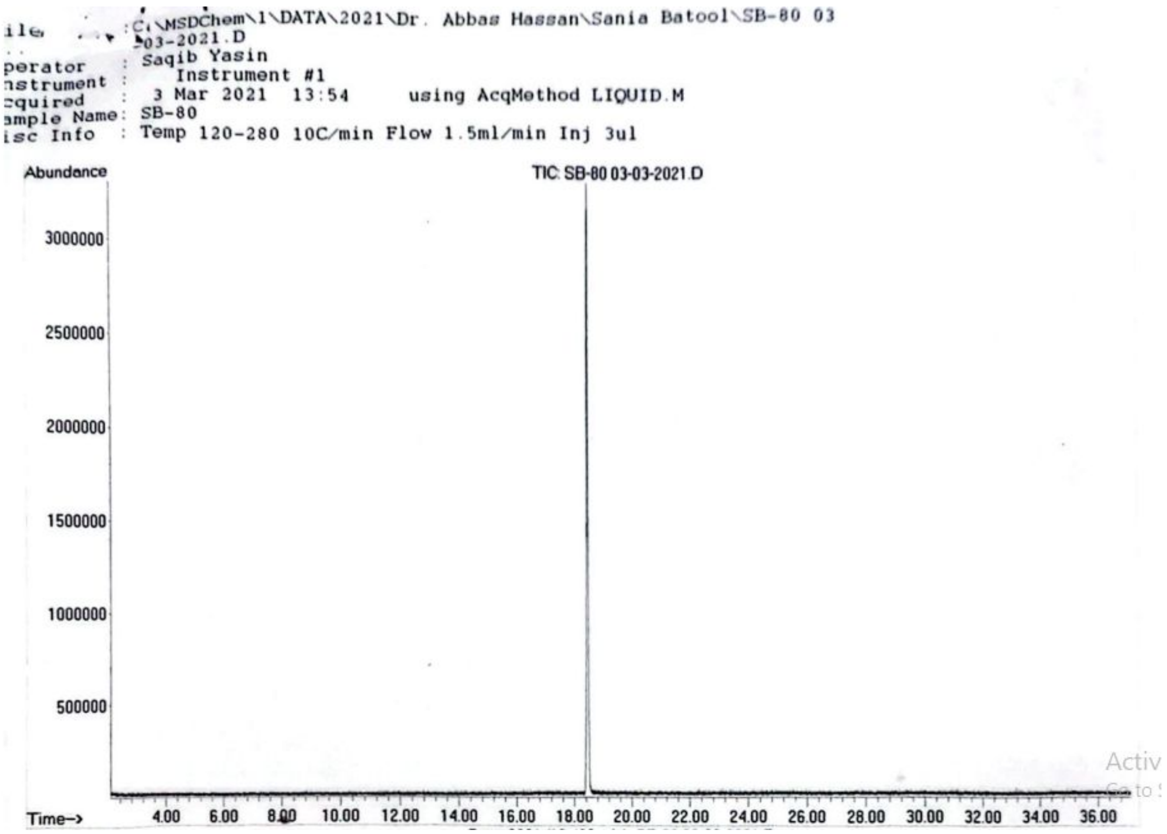

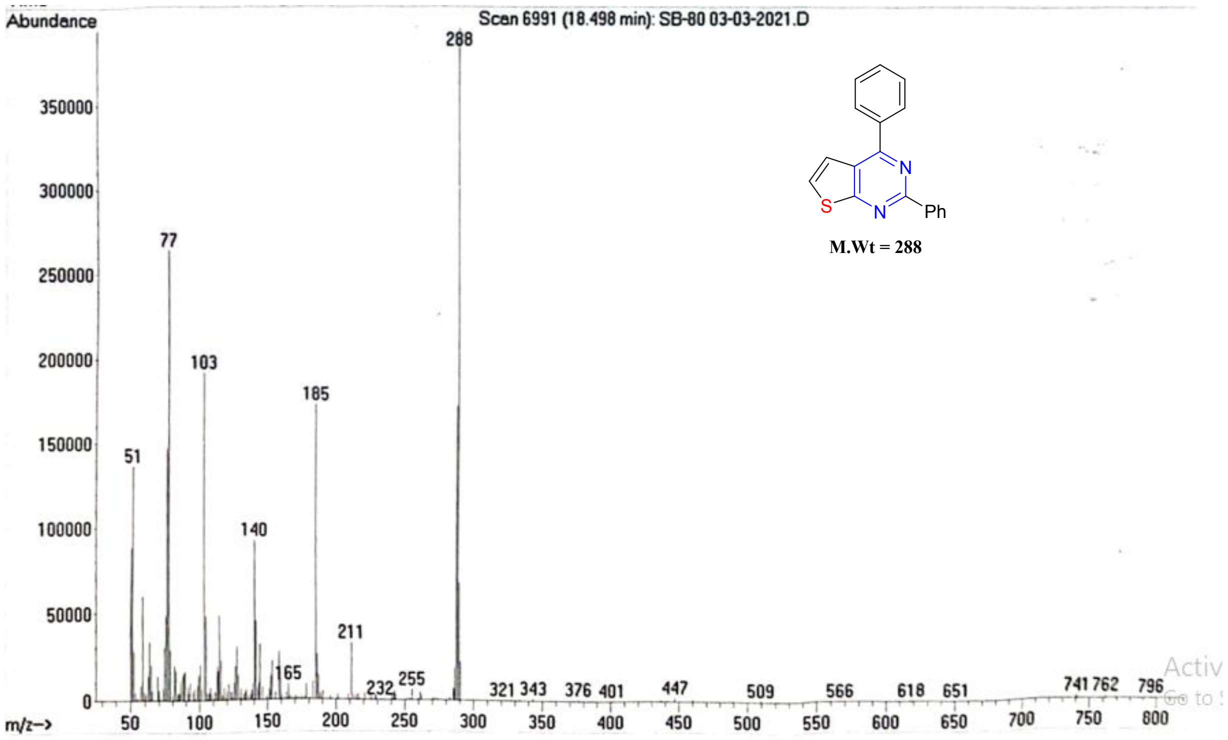

Fig. S-66: GCMS Spectrum of **5a**

4-(4-Methoxyphenyl)-2-phenylthieno[2,3-*d*]pyrimidine (5b)

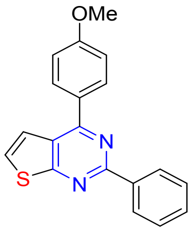

Yield: 80%

mp = 190-194 °C

IR: (*v,* cm^{-1}) 3091-3059, 1583-1378, 1327-1007, 721-677

^{1}H NMR (300 MHz, $CDCl_3$): δ (ppm) 8.66-8.63 (m, 2H), 8.10-8.05 (m, 2H), 7.62-7.60 (d, *J* = 6.0 Hz, 1H), 7.53-7.48 (m, 4H), 7.12-7.07 (m, 2H), 3.91 (s, 3H)

^{13}C NMR (75 MHz, $CDCl_3$): δ (ppm) 170.9, 161.6, 160.2, 159.6, 137.9, 131.0, 130.8, 130.5, 128.6, 128.6, 126.3, 125.6, 121.2, 114.3, 55.6

GC-MS Analysis (m/z): M^+ = 318, 215, 200, 103

HRMS-ESI (m/z): $[M+H]^+$ calc'd for $C_{19}H_{15}N_2OS^+$, 319.0947; found, 319.0949

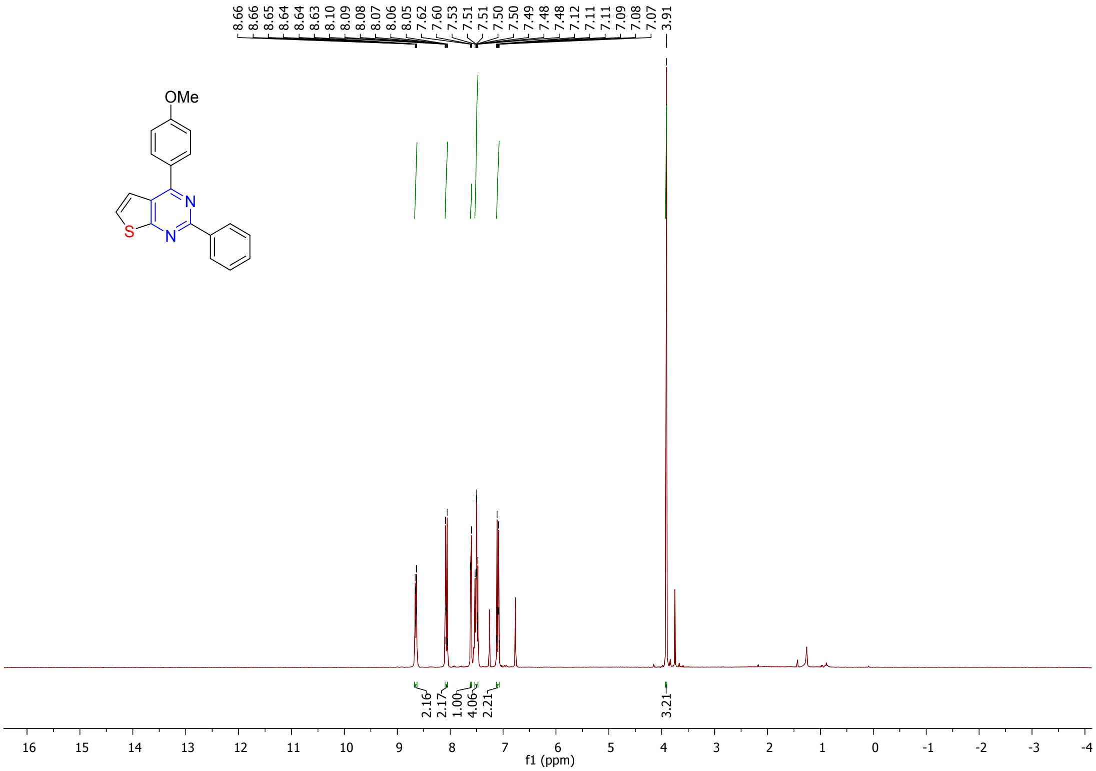

Fig. S-67: ^{1}HNMR Spectrum of **5b**

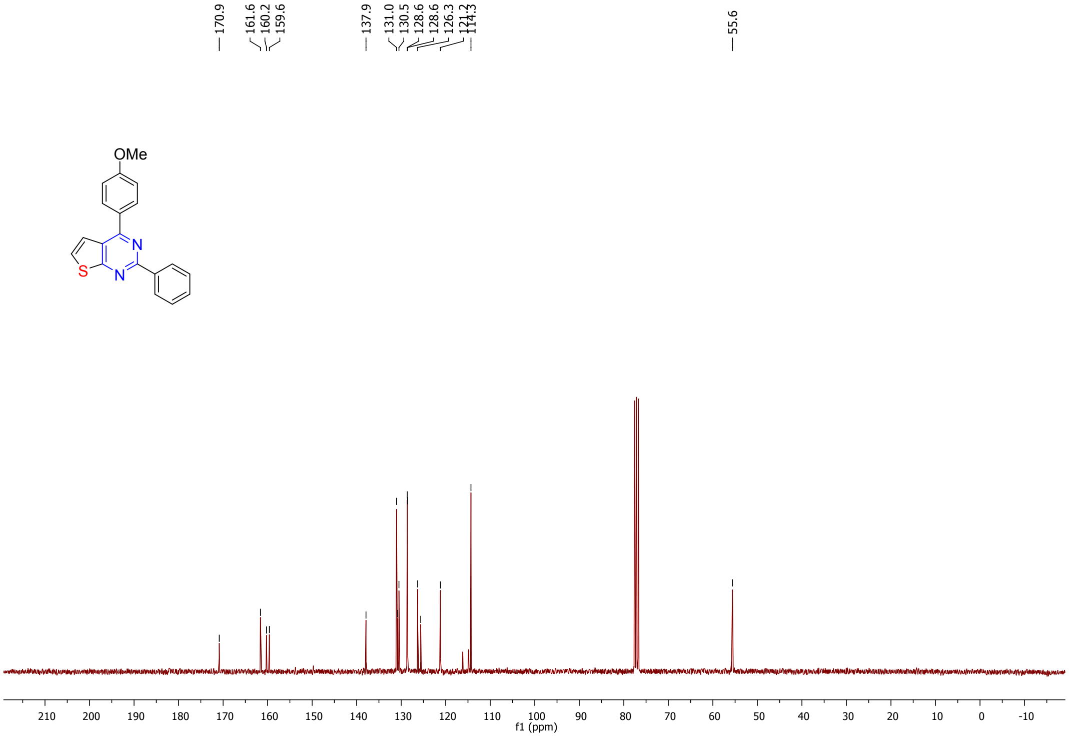

Fig. S-68: ^{13}C NMR Spectrum of **5b**

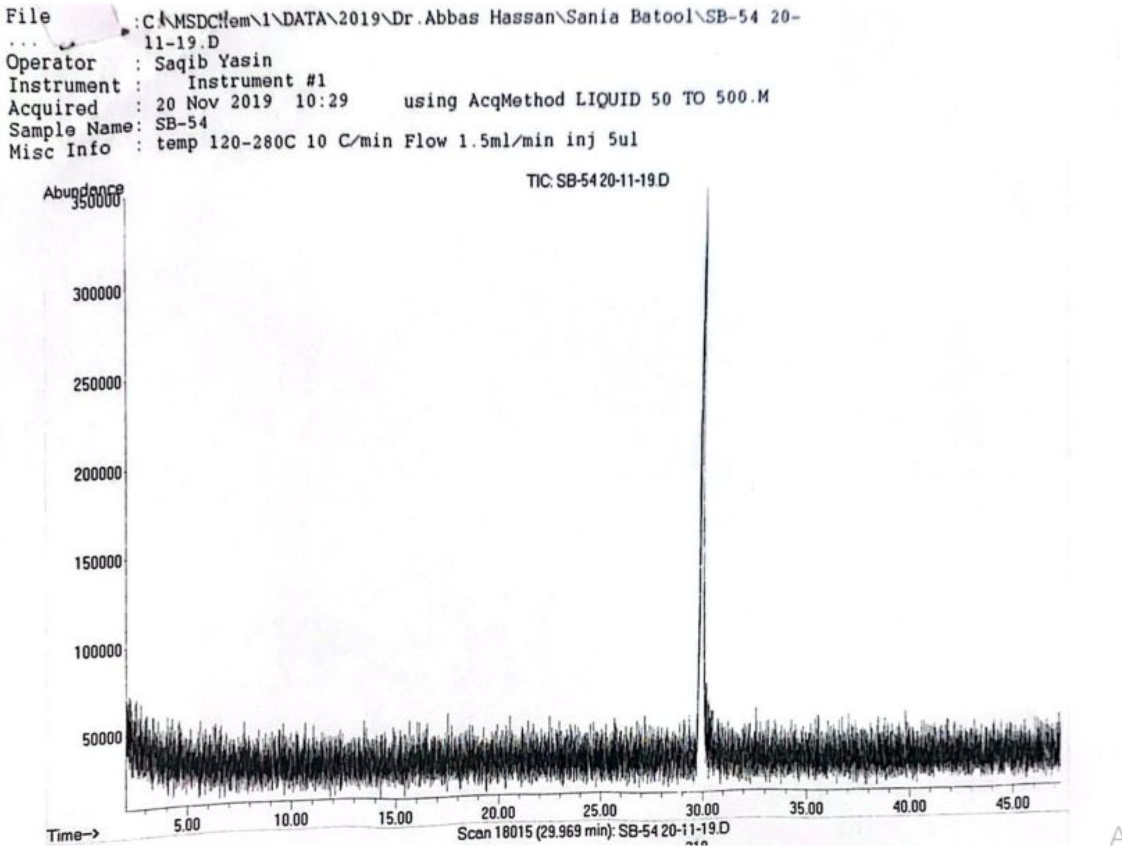

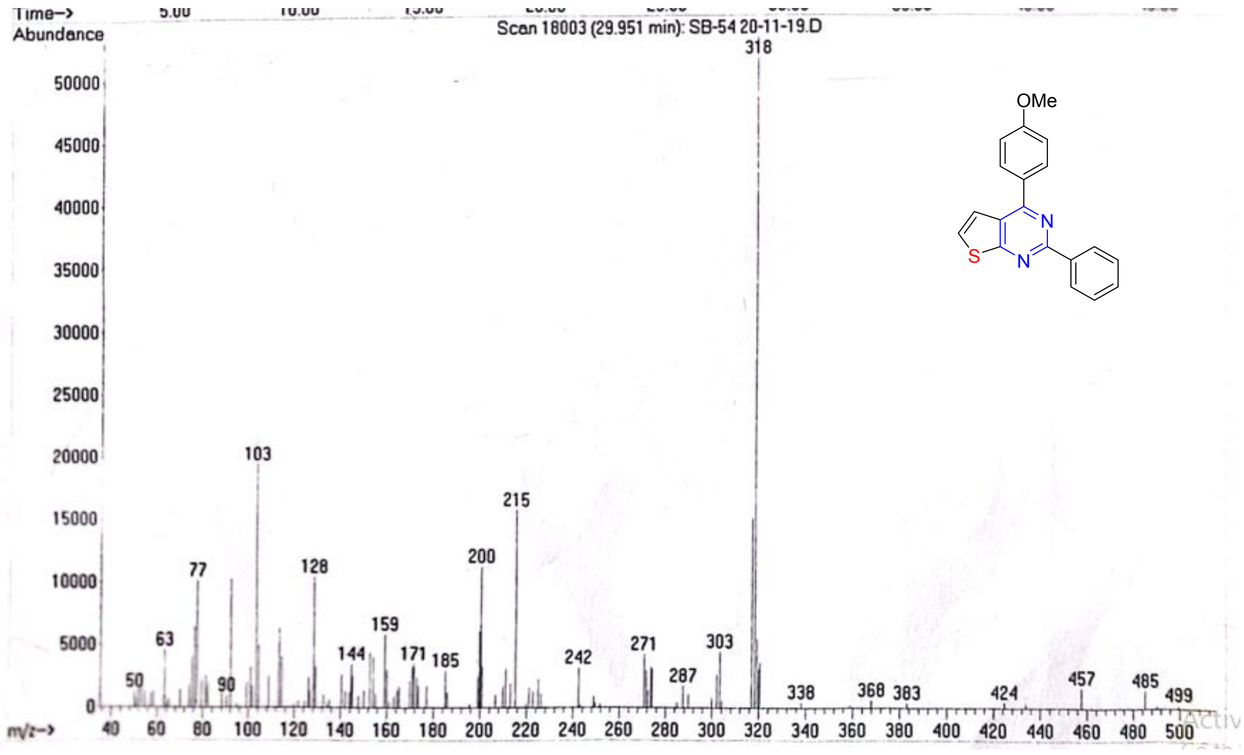

Fig. S-69: GCMS Spectrum of **5b**

4-(4-(Benzyloxy)phenyl)-2-phenylthieno[2,3-*d*]pyrimidine (5c)

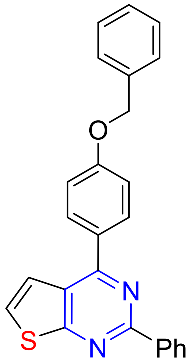

Yield: 83%

mp = 188-192 °C

IR: (*v,* cm^{-1}) 3086-3012, 1594-1355, 1347-996, 731-687

^{1}H NMR (300 MHz, $CDCl_3$): δ (ppm) 8.67-8.64 (m, 2H), 8.10-8.05 (m, 2H), 7.62-7.60 (d, J = 6 Hz, 1H), 7.60-7.37 (m, 9H), 7.20-7.15 (m, 2H), 5.18 (s, 2H)

^{13}C NMR (75 MHz, $CDCl_3$): δ (ppm) 171.0, 160.8, 160.2, 159.7, 138.0, 136.7, 130.0, 130.5, 128.8, 128.6, 128.6, 128.3, 127.6, 126.3, 125.6, 121.2, 115.2, 70.2

GC-MS Analysis (m/z): M^+ = 394, 303, 291, 287, 103, 91, 77, 65, 51

HRMS-ESI (m/z): $[M+H]^+$ calc'd for $C_{25}H_{19}N_2OS^+$, 395.4955; found, 395.4959

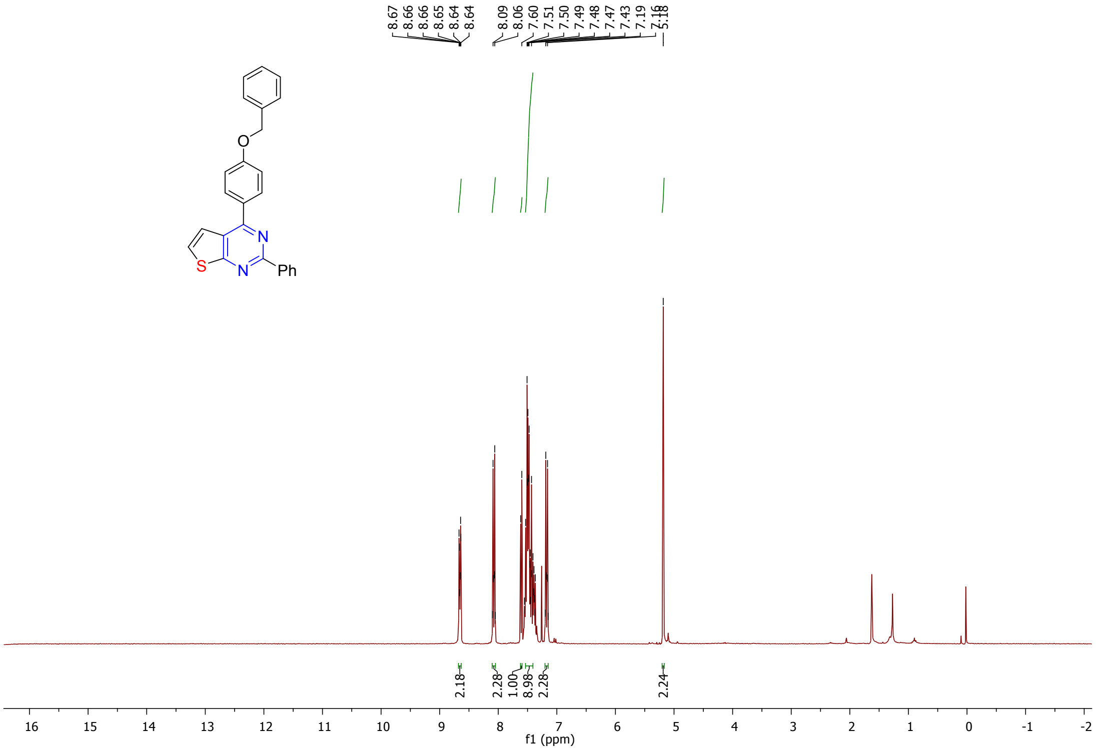

Fig. S-70: ^{1}HNMR Spectrum of **5c**

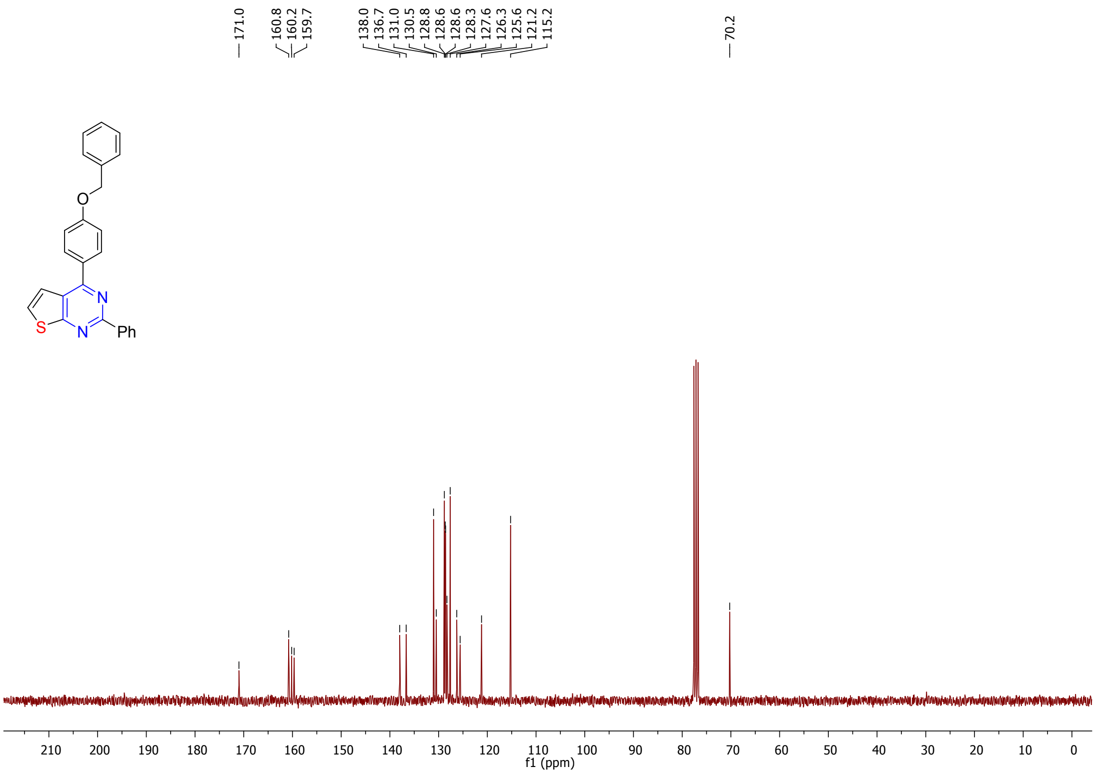

Fig. S-71: ^{13}C NMR Spectrum of **5c**

2-Phenyl-4-(pyridin-3-yl) thieno[2,3-*d*]pyrimidine (5d)

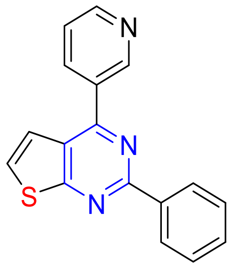

Yield: 43%

mp = 186-189 °C

IR: (*v,* cm^{-1}) 3093-3057, 1573-1396, 1327-1027, 724-690

^{1}H NMR (300 MHz, $CDCl_3$): δ (ppm) 9.30-9.29 (*ap*. d, *J* = 3.0 Hz, 1H), 8.83-8.81 (dd, *J* = 6.0 Hz, 3.0 Hz, 1H), 8.64-8.60 (m, 2H), 8.44-8.40 (*ap*. dt, *J* = 6.0 Hz, 3.0 Hz, 1H), 7.58-7.50 (m, 6H)

^{13}C NMR (75 MHz, $CDCl_3$): δ (ppm) 171.2, 159.8, 157.7, 151.2, 150.1, 137.4, 136.8, 134.1, 130.8, 128.7, 128.5, 127.7, 126.0, 123.9, 120.3

GC-MS Analysis (m/z): M^+ = 289, 263, 186, 103, 77, 65

HRMS-ESI (m/z): $[M+H]^+$ calc'd for $C_{17}H_{12}N_3S^+$, 290.0765; found, 290.0768

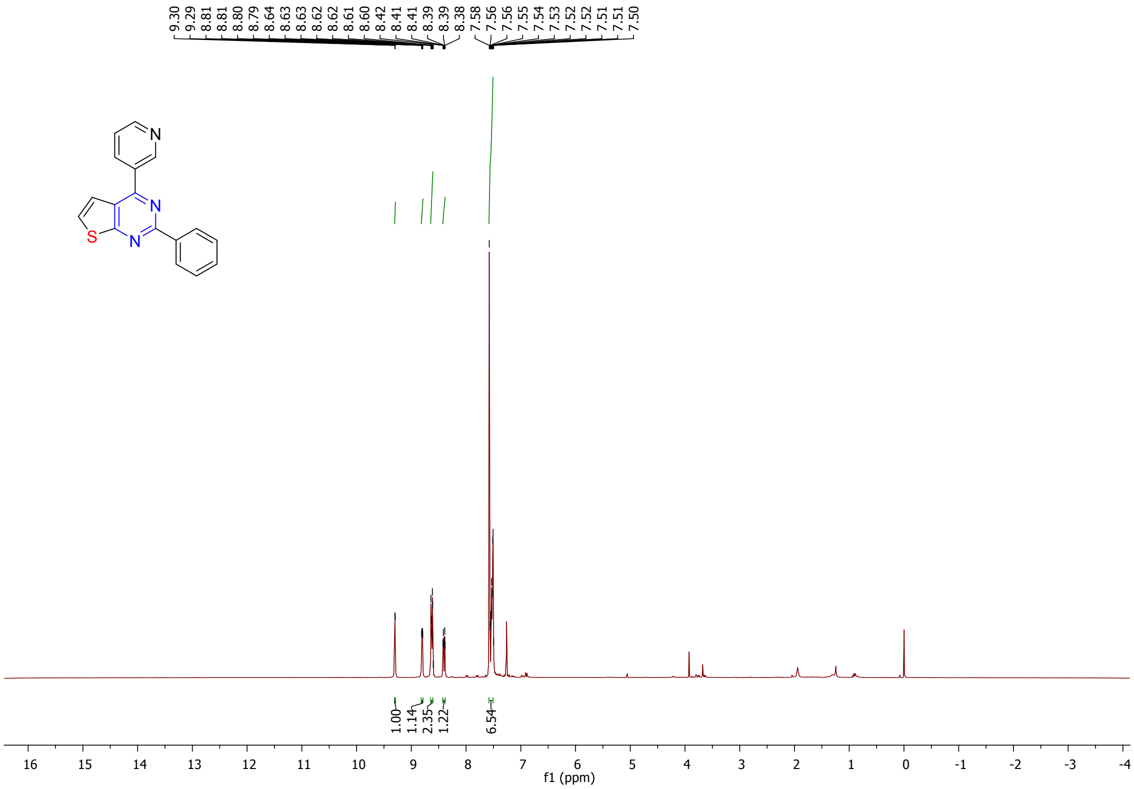

Fig. S-72: ^{1}HNMR Spectrum of **5d**

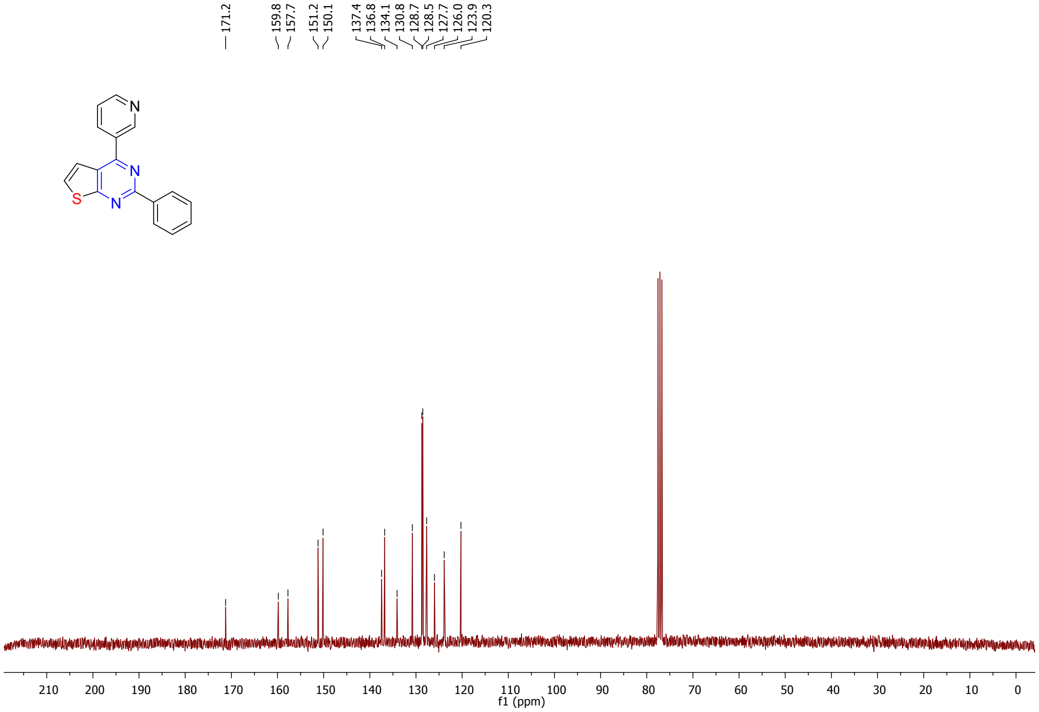

Fig. S-73: ^{13}C NMR Spectrum of **5d**

4-(4-Methoxyphenyl)-2-methylthieno[2,3-*d*]pyrimidine (5e)

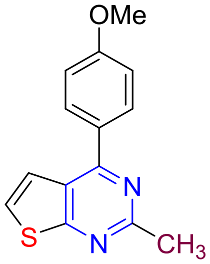

Yield: 79%

mp = 172-175 °C

IR (*ν*, cm^{-1}) 3088-3067, 1578-1365, 1327-1017, 728-685

^{1}H NMR (300 MHz, $CDCl_3$): δ (ppm) 7.95-7.90 (m, 2H), 7.55-7.53 (d, *J* = 6.0 Hz, 1H), 7.44-7.42 (d, *J* = 6.0 Hz, 1H), 7.10-7.05 (m, 2H), 3.90 (s, 3H), 2.89 (s, 3H)

^{13}C NMR (75 MHz, $CDCl_3$): δ (ppm) 170.3, 162.9, 161.4, 160.4, 130.7, 130.4, 125.2, 124.9, 120.9, 114.2, 55.4, 26.1

GC-MS Analysis (m/z): M^+ = 256, 215, 149, 122, 92, 58

HRMS-ESI (m/z): $[M+H]^+$ calc'd for $C_{14}H_{13}N_2OS^+$, 257.0774; found, 257.0778

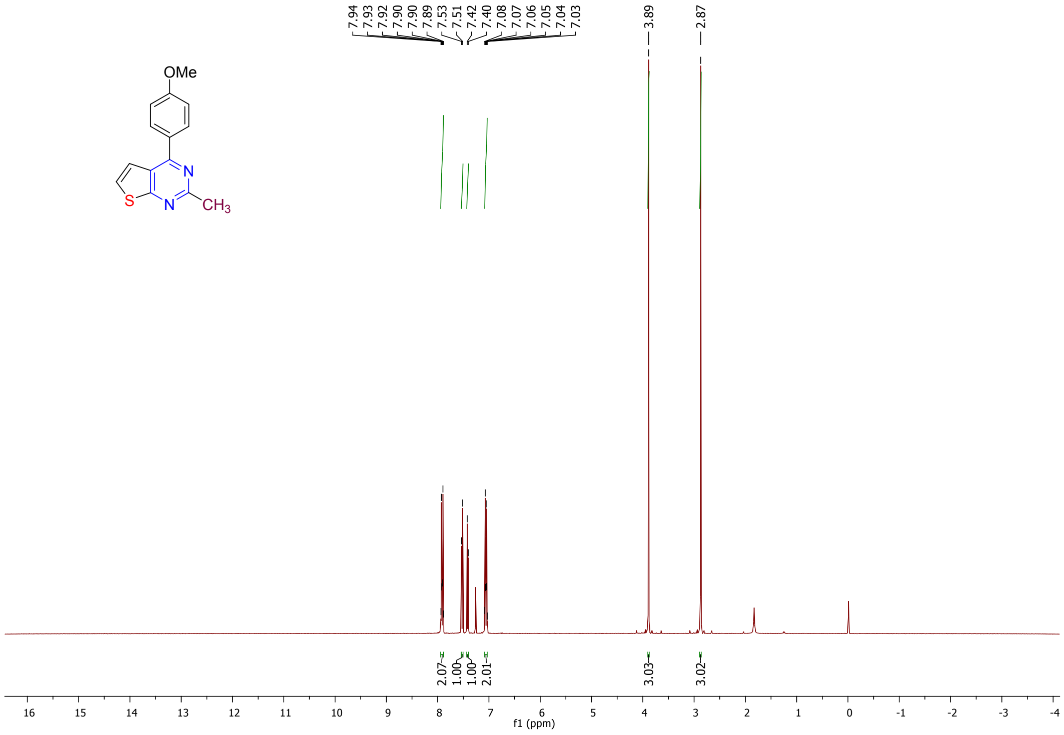

Fig. S-74: ^{1}HNMR Spectrum of **5e**

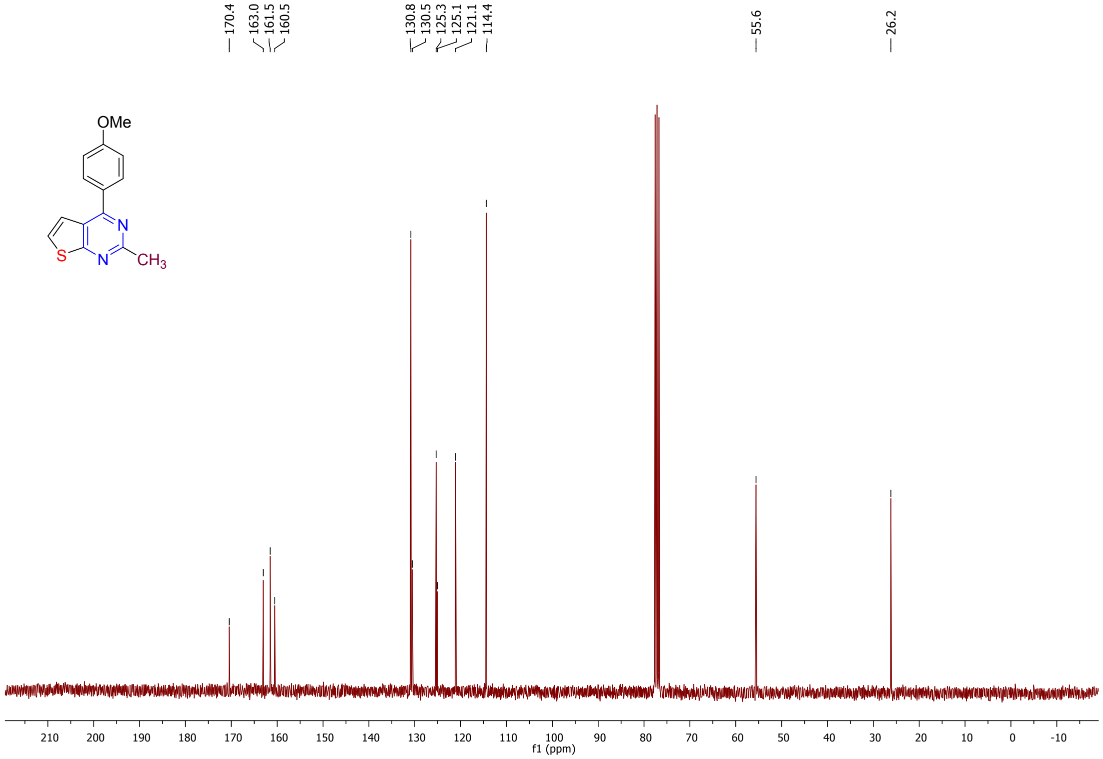

Fig. S-75: ^{13}C NMR Spectrum of **5e**

ile • ;C:\MSDChem\1\DATA\2021\Dr. Abbas Hassan\Sania Batool\SB-81 03
-03-2021.D
perator : Saqib Yasin
nstrument : Instrument #1
cquired : 3 Mar 2021 14:34 using AcqMethod LIQUID.M
ample Name: SB-81
isc Info : Temp 120-280 10C/min Flow 1.5ml/min Inj 3ul

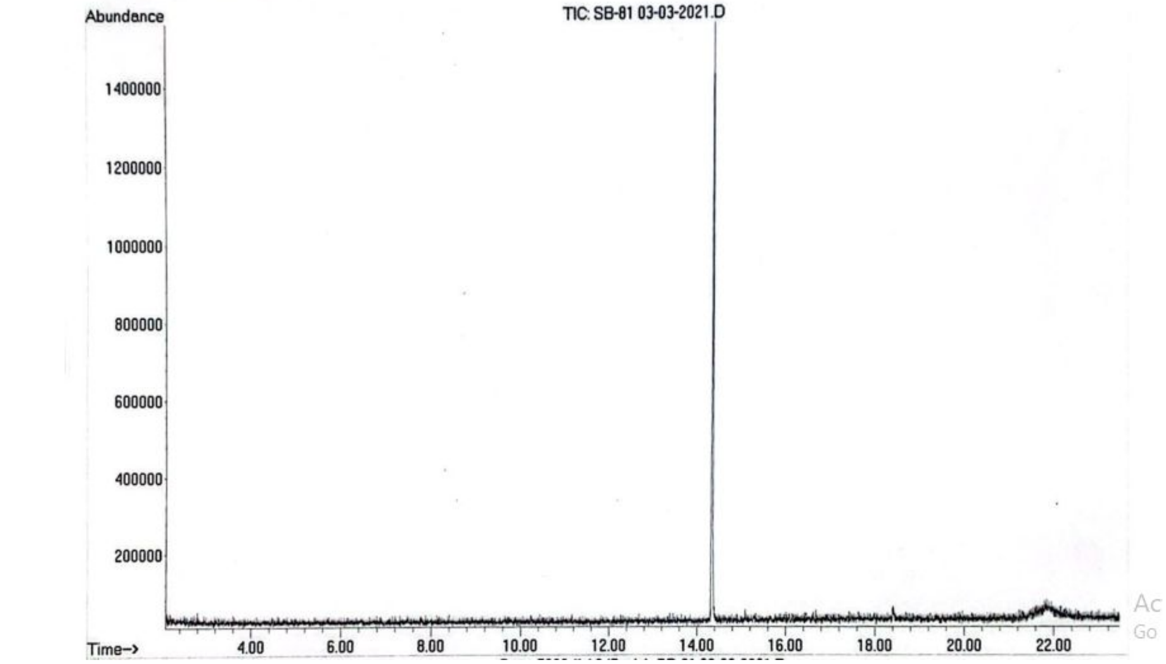

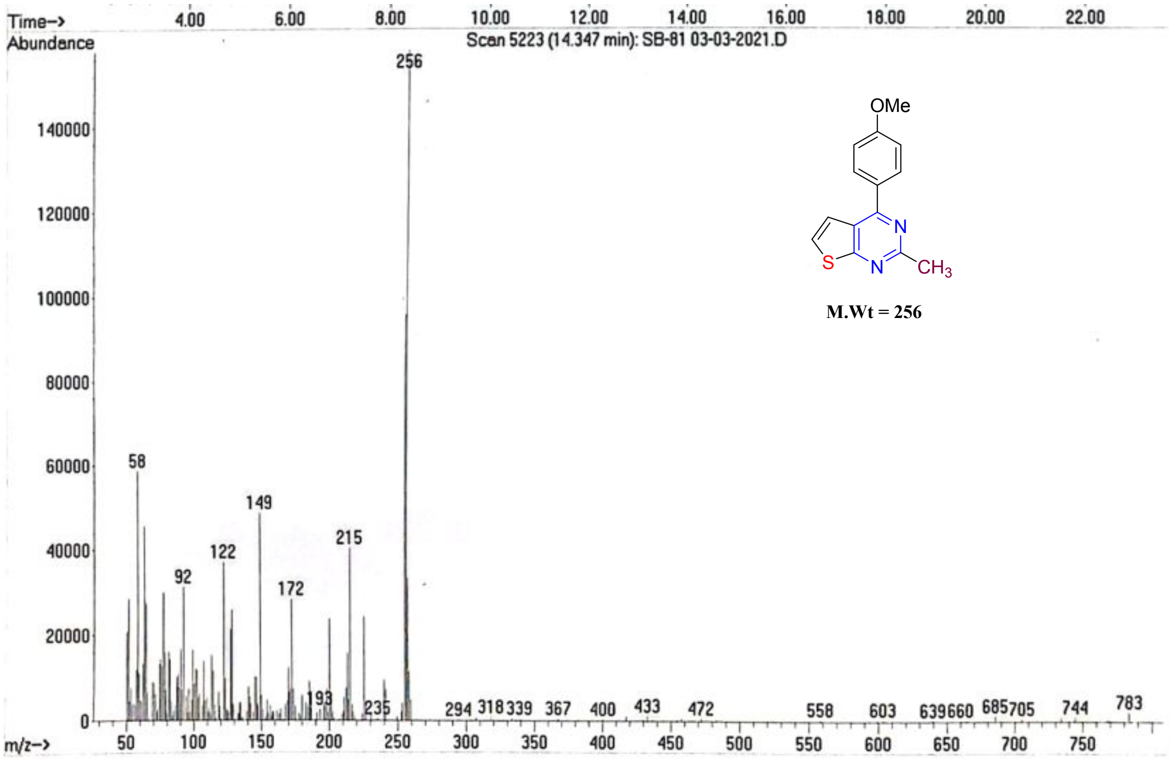

Fig. S-76: GCMS Spectrum of **5e**

2-(*tert*-Butyl)-4-(4-methoxyphenyl)thieno[2,3-*d*]pyrimidine (5f)

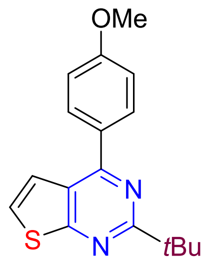

Yield: 76%

mp = 165-168 °C

IR (*ν*, cm^{-1}) 3087-3034, 1567-1398, 1314-1047, 728-695

^{1}H NMR (300 MHz, $CDCl_3$): δ (ppm) 8.04-7.99 (m, 2H), 7.58-7.56 (d, *J* = 6.0 Hz, 1H), 7.44-7.42 (d, *J* = 6.0 Hz, 1H), 7.10-7.05 (m, 2H), 3.90 (s, 3H), 1.53 (s, 9H)

^{13}C NMR (75 MHz, $CDCl_3$): δ (ppm) 172.3, 170.4, 161.4, 159.5, 131.2, 131.0, 125.4, 124.6, 120.9, 114.3, 55.6, 39.7, 30.0

GC-MS Analysis (m/z): M$^+$ = 298, 283, 256, 135, 68

HRMS-ESI (m/z): [M+H]$^+$ calc'd for $C_{17}H_{19}N_2OS^+$, 299.1146; found, 299.1149

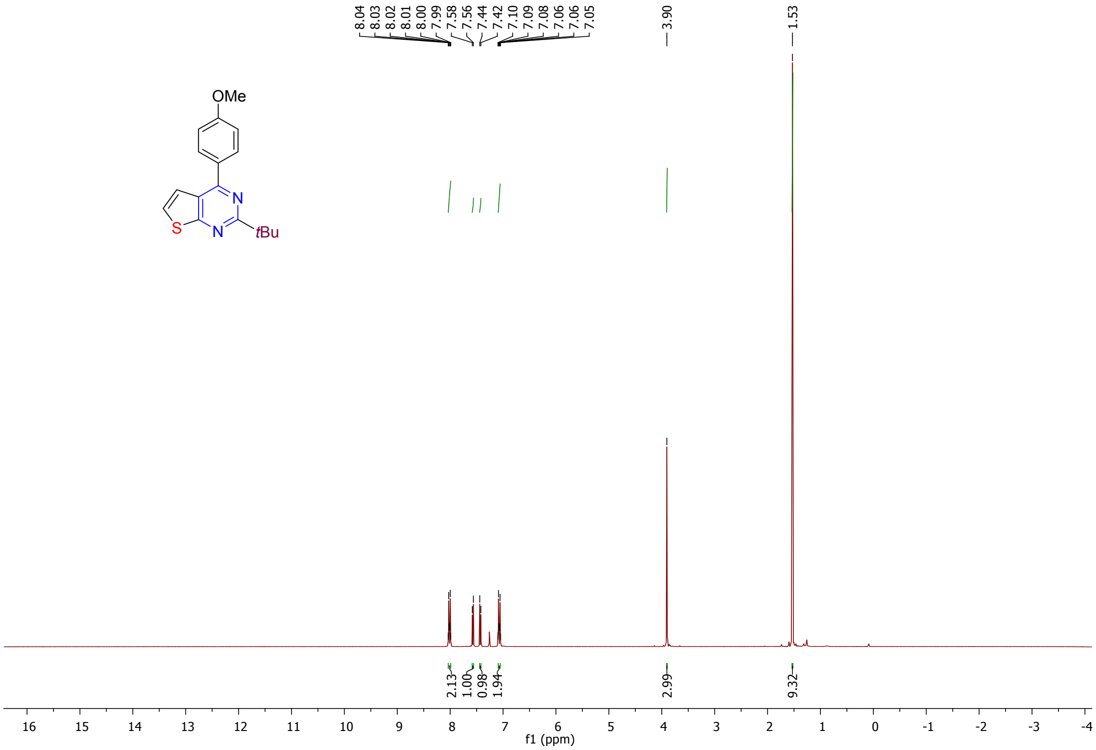

Fig. S-77: ^{1}HNMR Spectrum of **5f**

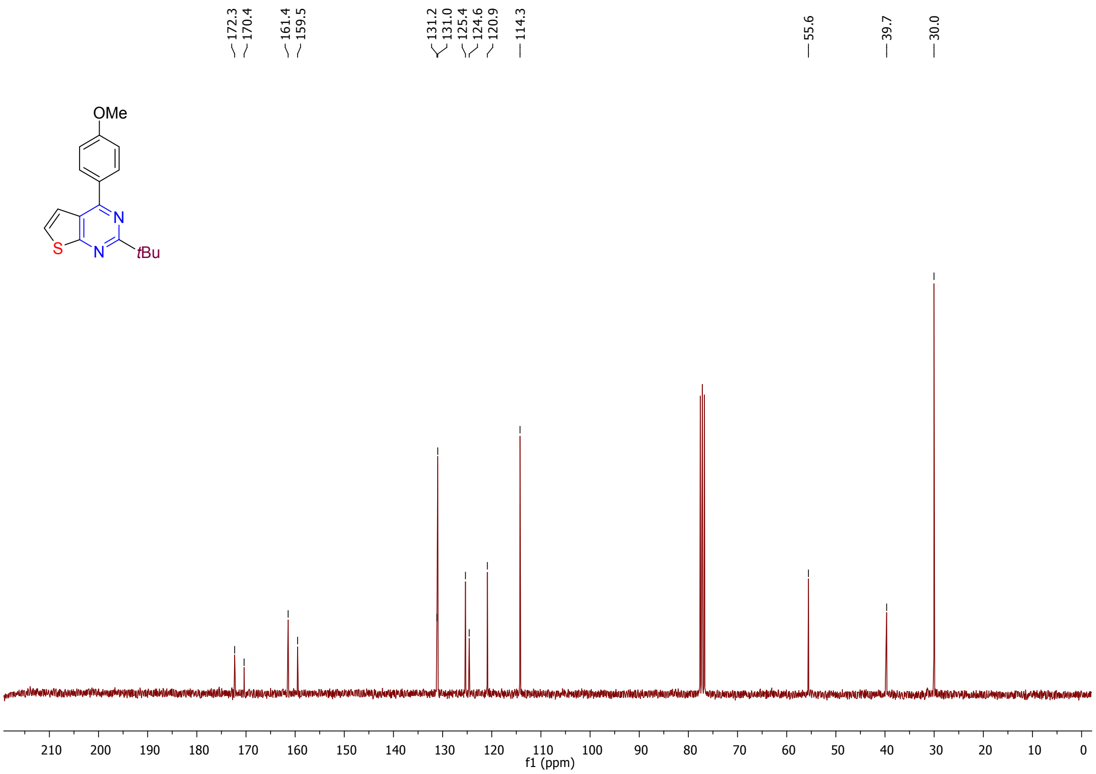

Fig. S-78: ^{13}C NMR Spectrum of **5f**

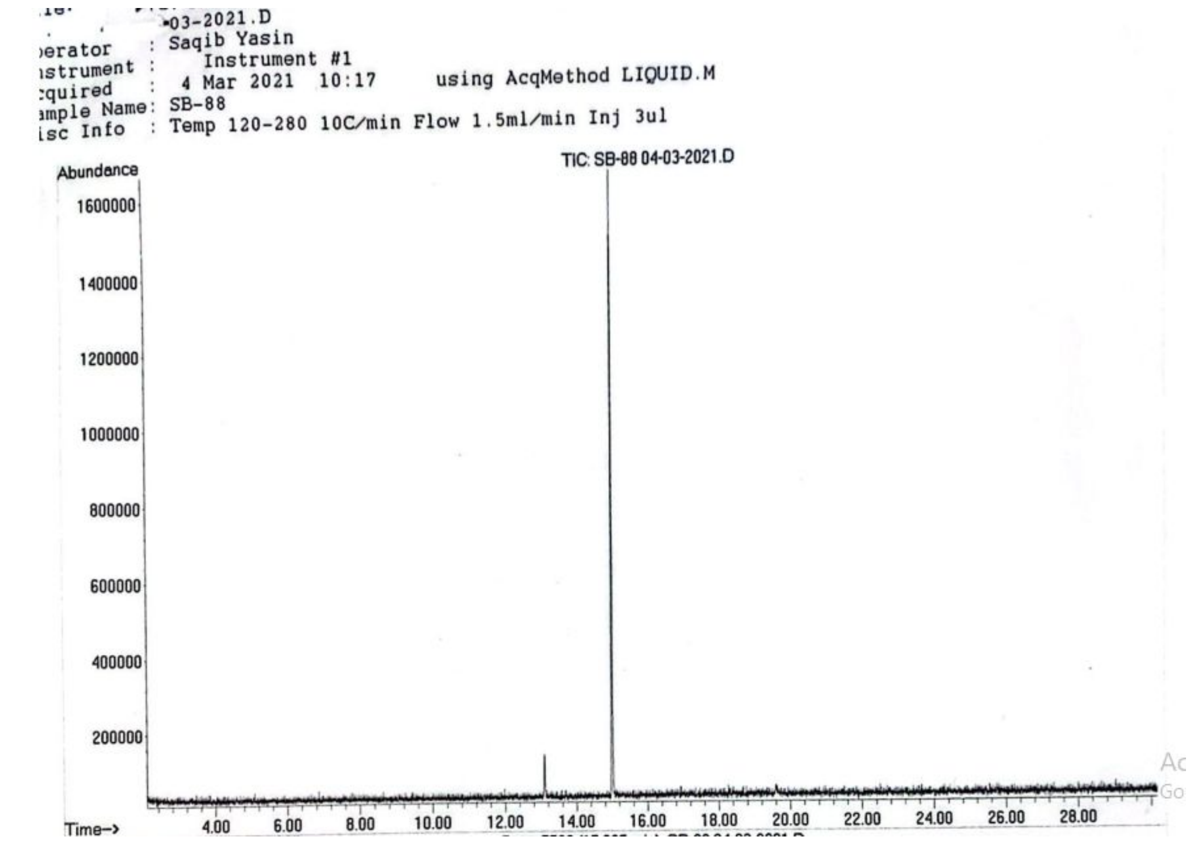

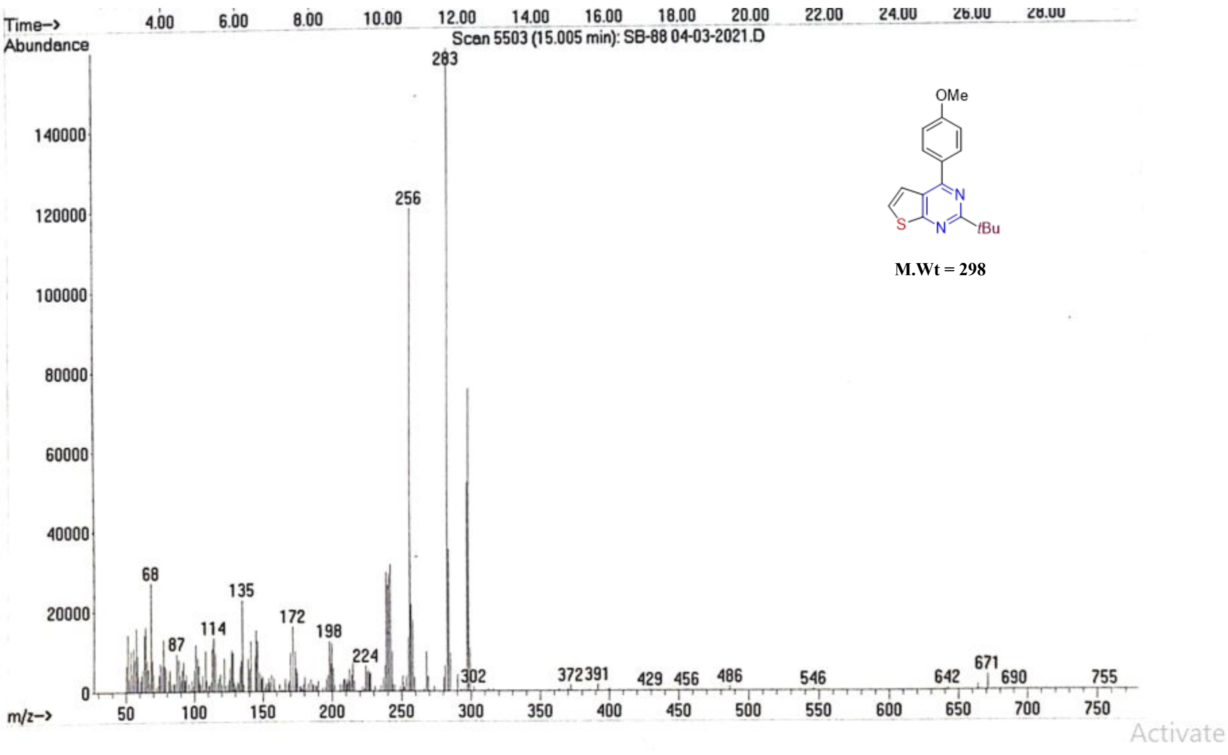

Fig. S-79: GCMS Spectrum of **5f**

2-(*tert*-Butyl)-4-(pyridin-3-yl)thieno[2,3-*d*]pyrimidine (5g)

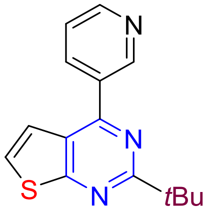

Yield: 48%

mp = 173-177 ˚C

IR (*v,* cm^{-1}) 3094-3047, 1586-1374, 1313-1078, 719-688

^{1}H NMR (300 MHz, $CDCl_3$): δ (ppm) 9.25 (*ap*. d, *J* = 3.0 Hz, 1H), 8.78-8.76 (*ap*. d, *J* = 6.0 Hz, 1H), 8.40-8.37 (dt, *J* = 9.0 Hz, 3.0 Hz, 1H), 7.57-7.52 (m, 3H), 1.51 (s, 9H)

^{13}C NMR (75 MHz, $CDCl_3$): δ (ppm) 172.7, 170.8, 156.6, 150.3, 149.6, 137.3, 134.7, 127.0, 125.0, 124.0, 119.8, 39.8, 30.0

GC-MS Analysis (m/z): M^+ = 269, 254, 227, 78, 51

HRMS-ESI (m/z): $[M+H]^+$ calc'd for $C_{15}H_{16}N_3S^+$, 270.0988; found, 270.0992

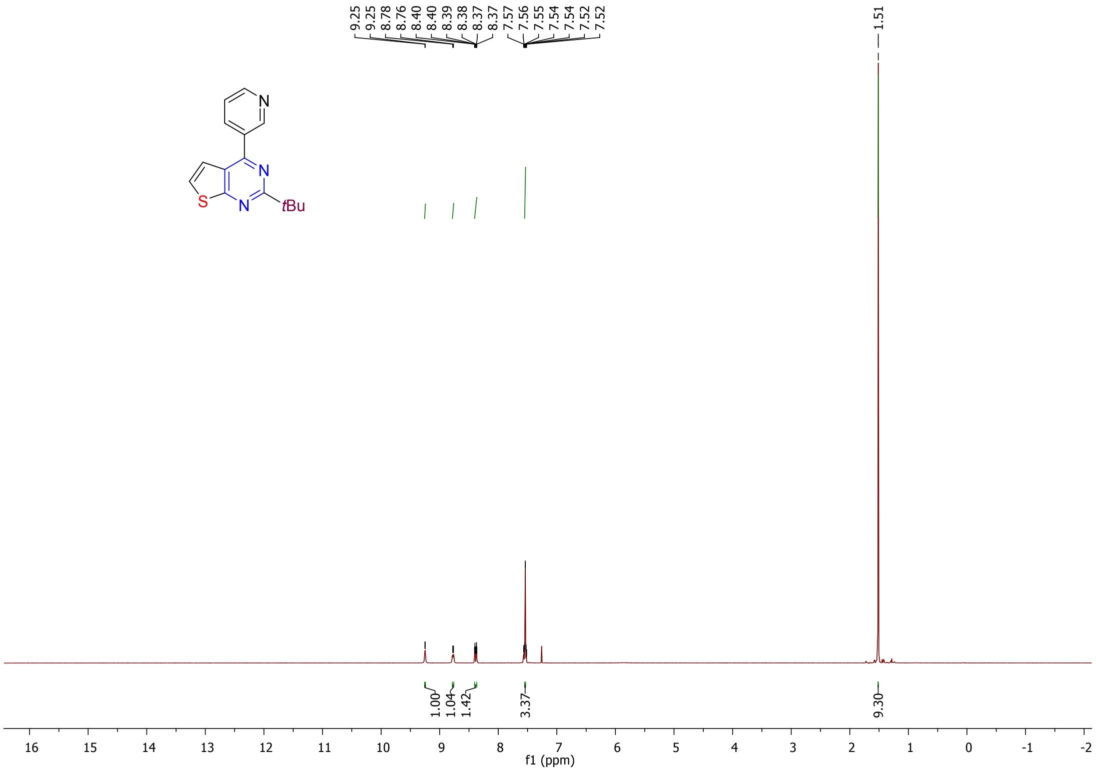

Fig. S-80: ^{1}HNMR Spectrum of **5g**

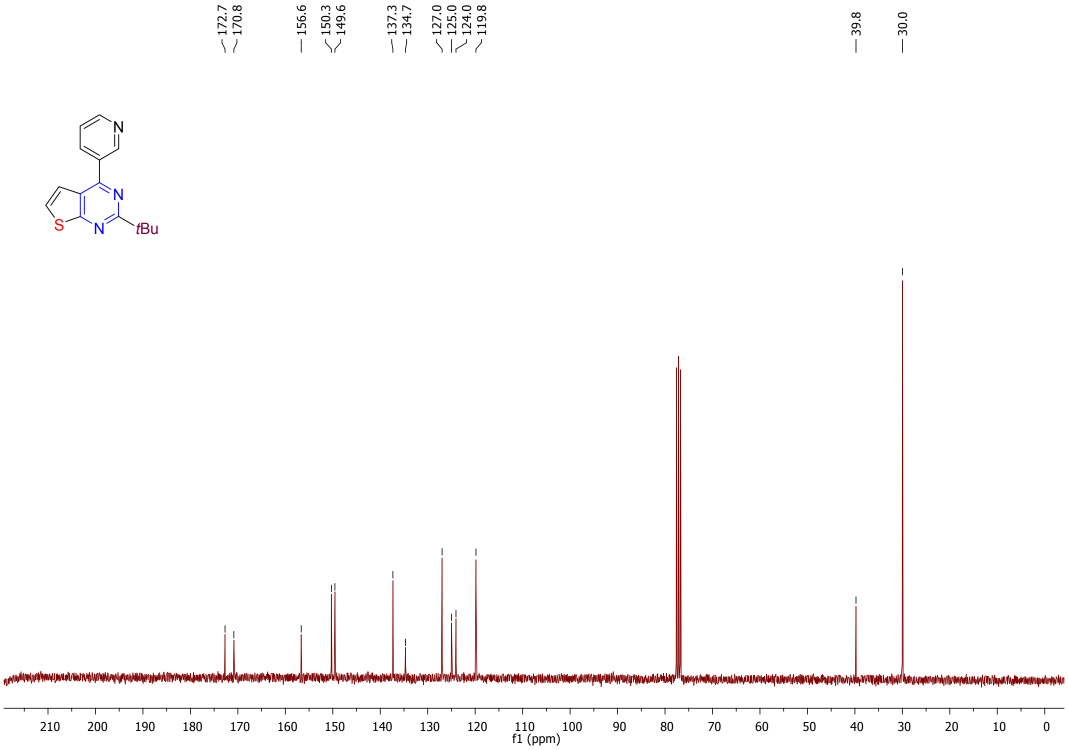

Fig. S-81: ^{13}C NMR Spectrum of **5g**

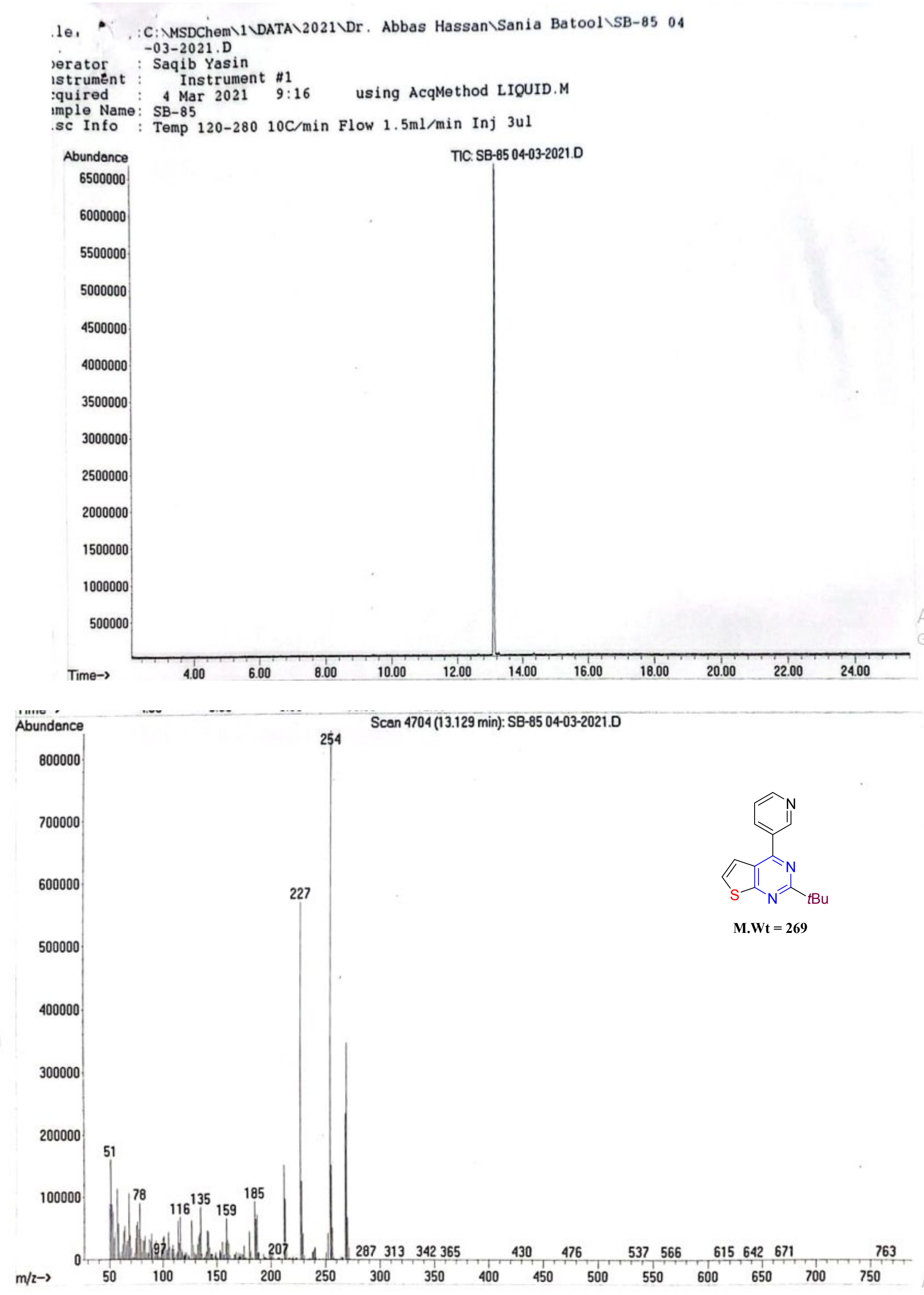

Fig. S-82: GCMS Spectrum of **5g**

2-Phenyl-4-(phenyl ethynyl)thieno[2,3-*d*]pyrimidine (6a)

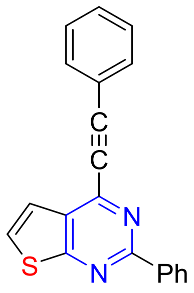

Yield: 51%

mp = 177-180 °C

IR (*v,* cm^{-1}) 3071, 2205, 1524-1379, 1310-1028, 756-722, 682

^{1}H NMR (300 MHz, $CDCl_3$): δ (ppm) 8.59-8.56 (m, 2H), 7.74-7.71 (m, 2H), 7.57-7.41 (m, 8H)

^{13}C NMR (75 MHz, $CDCl_3$): δ (ppm) 169.7, 160.2, 145.4, 137.4, 132.6, 130.7, 130.1, 129.7, 128.7, 128.7, 127.5, 121.5, 120.7, 96.5, 86.0

GC-MS Analysis (m/z): M^+ = 312, 278, 240, 209, 103, 76, 50

HRMS-ESI (m/z): $[M+H]^+$ calc'd for $C_{20}H_{13}N_2S^+$, 313.0821; found, 313.0826

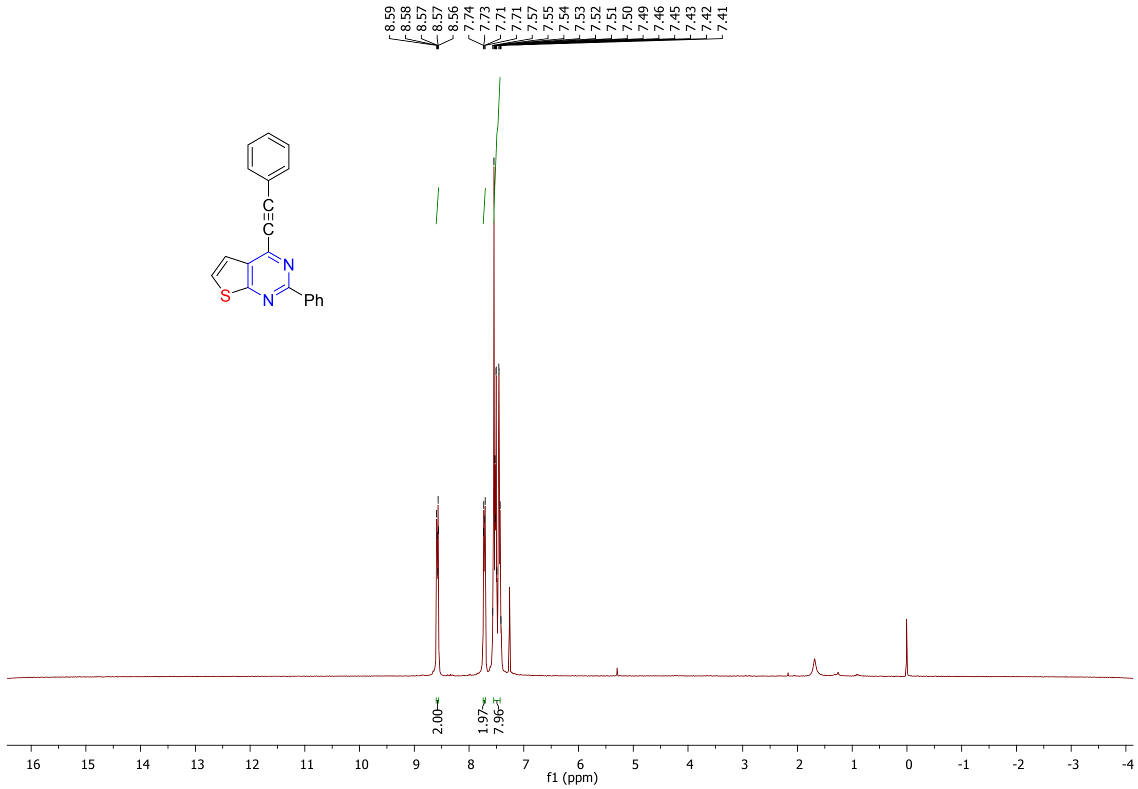

Fig. S-83: ^{1}HNMR Spectrum of **6a**

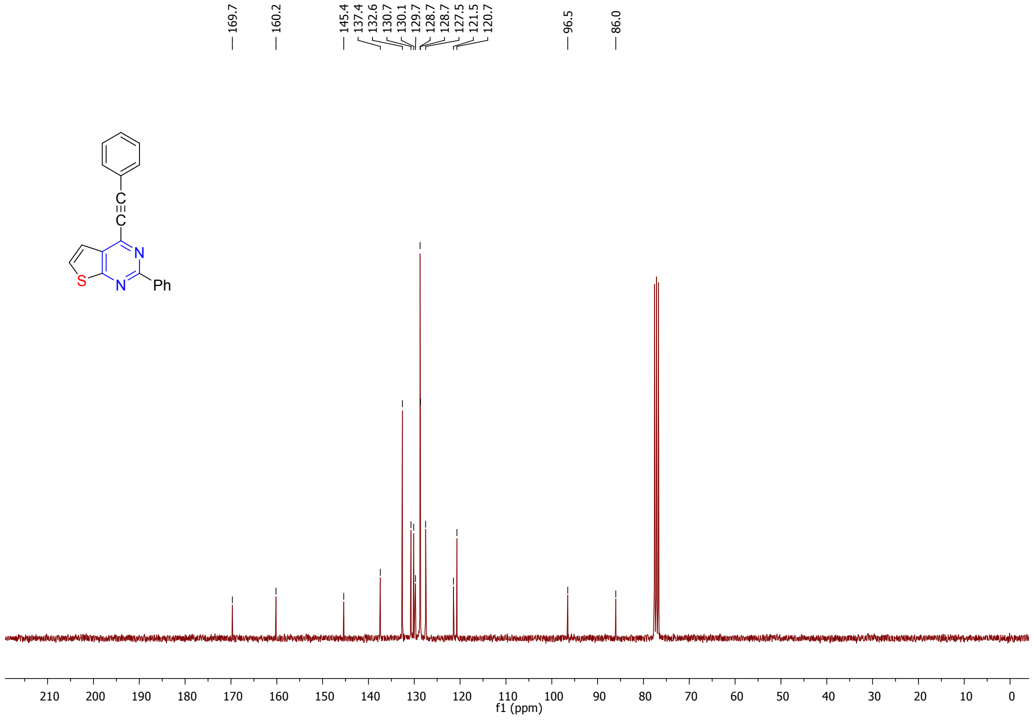

Fig. S-84: ^{13}C NMR Spectrum of **6a**

ile :C:\MSDChem\1\DATA\2021\Dr. Abbas Hassan\Sania Batool\SB-72 03
... -03-2021.D
perator : Saqib Yasin
nstrument : Instrument #1
cquired : 3 Mar 2021 12:38 using AcqMethod LIQUID.M
ample Name: SB-72
isc Info : Temp 120-280 10C/min Flow 1.5ml/min Inj 3ul

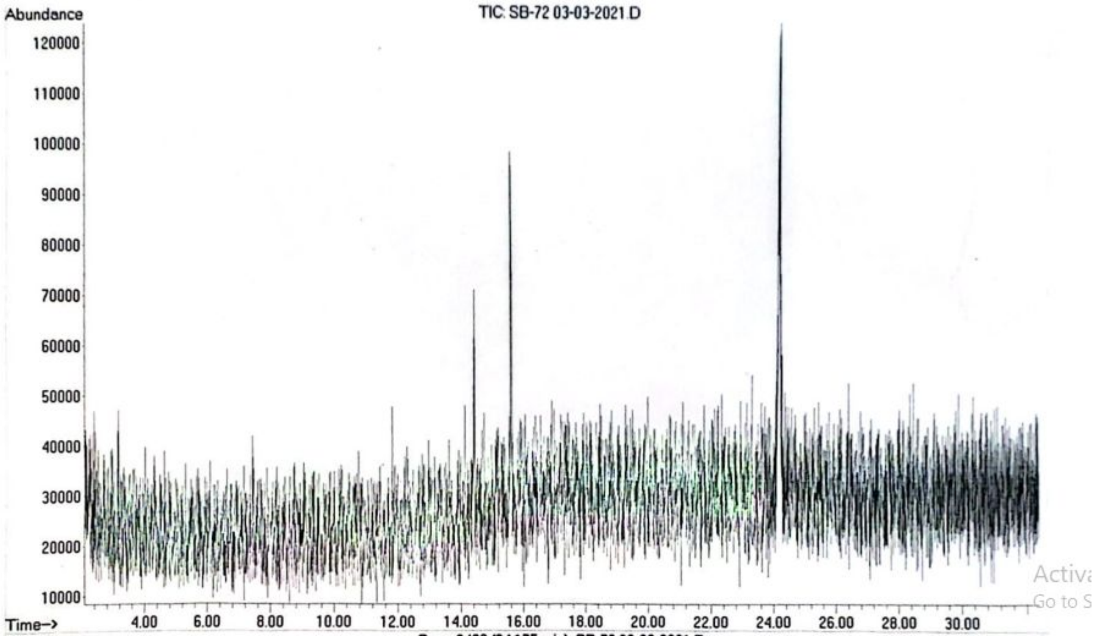

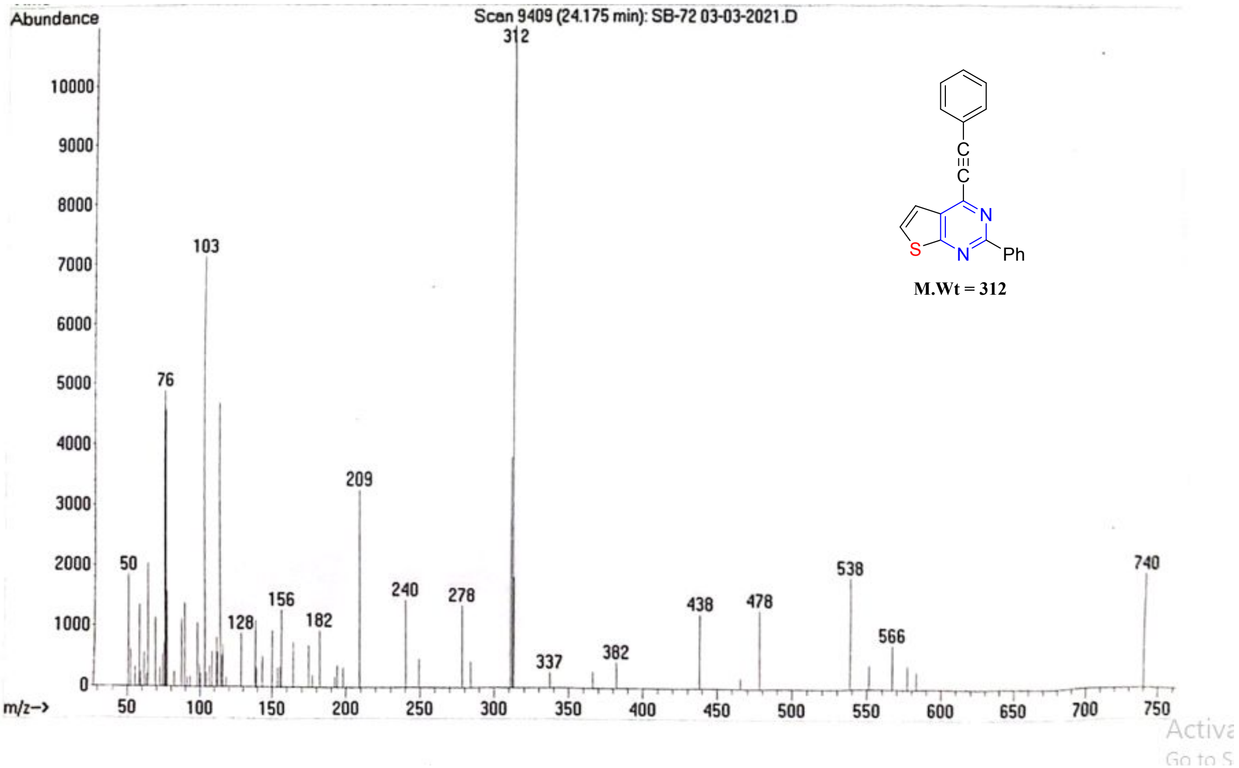

Fig. S-85: GCMS Spectrum of **6a**

2-(*tert*-Butyl)-4-(phenylethynyl)thieno[2,3-*d*]pyrimidine (6b)

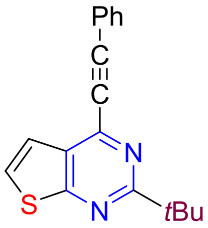

Yield: 54%

mp = 171-175 °C

IR (*v,* cm^{-1}) 3067, 2213, 1561-1355, 1321-1018, 752-719, 677

^{1}H NMR (300 MHz, $CDCl_3$): δ (ppm) 7.71-7.68 (m, 2H), 7.52-7.41 (m, 5H), 1.50 (s, 9H)

^{13}C NMR (75 MHz, $CDCl_3$): δ (ppm) 172.8, 169.1, 144.9, 132.5, 130.0, 129.0, 128.7, 126.7, 121.6, 120.5, 96.0, 86.2, 39.8, 30.0

HRMS-ESI (m/z): $[M+H]^+$ calc'd for $C_{18}H_{17}N_2S^+$, 293.1056; found, 293.1059

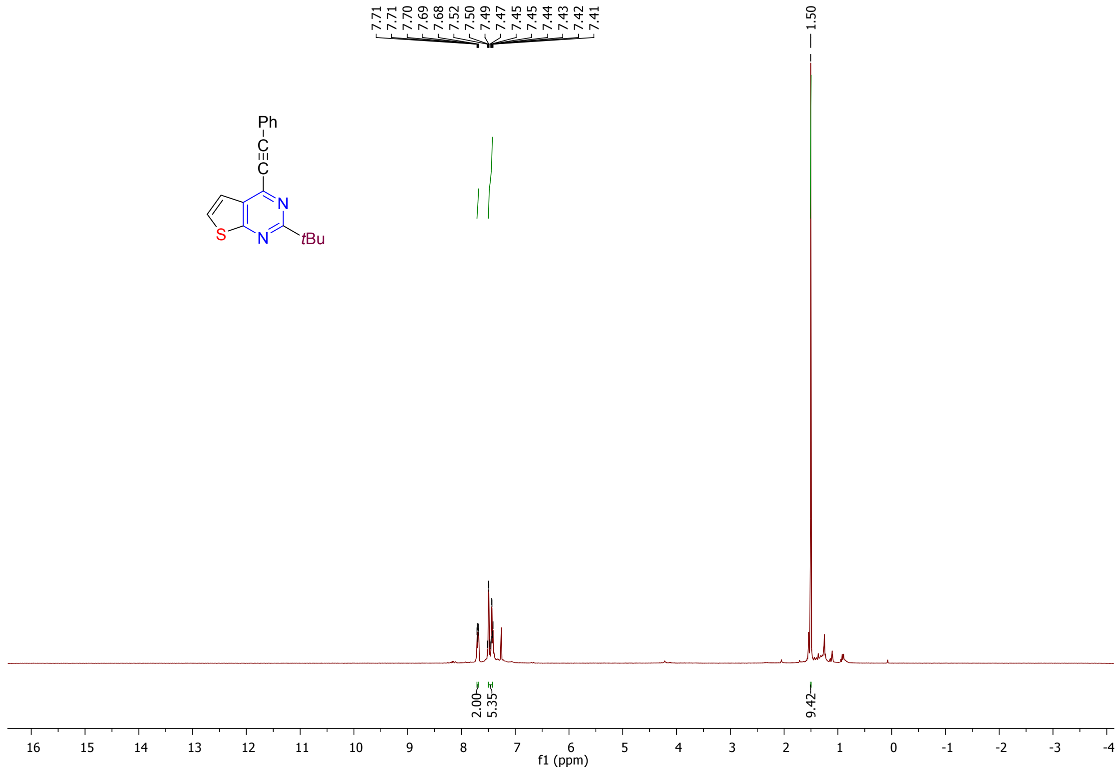

Fig. S-86: ^{1}HNMR Spectrum of **6b**

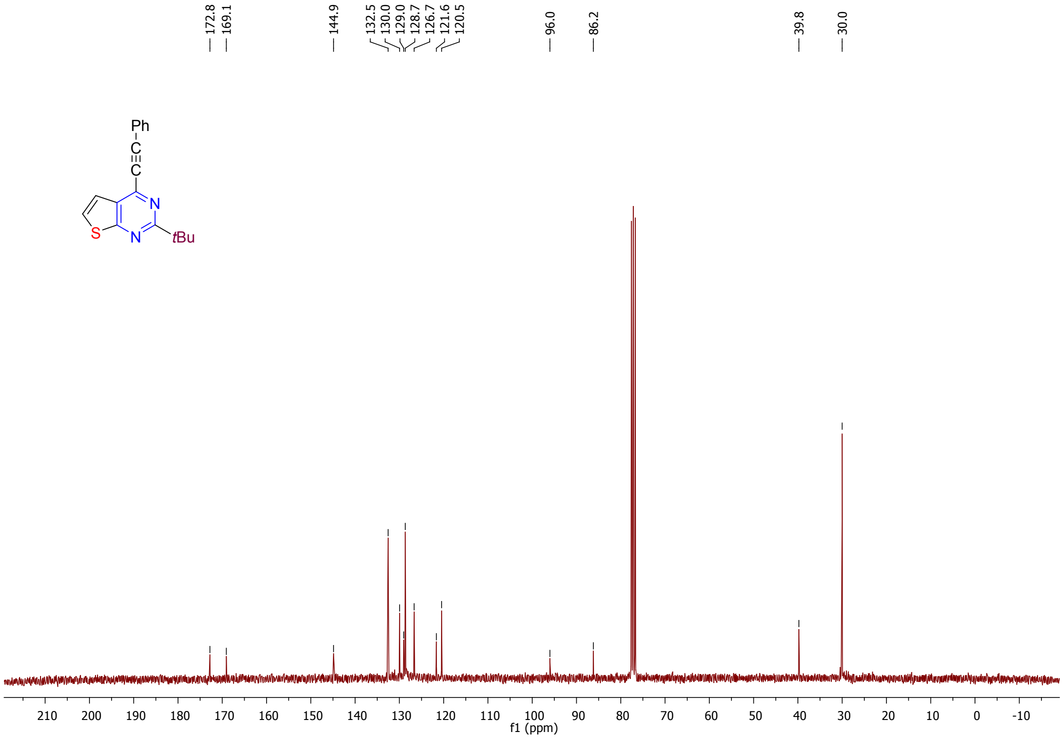

Fig. S-87: ^{13}C NMR Spectrum of **6b**

4-(Hex-1-yn-1-yl)-2-phenylthieno[2,3-*d*]pyrimidine (6c)

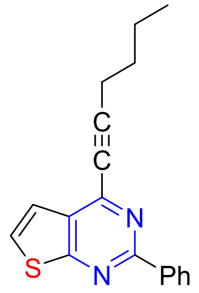

Yield: 43%

mp = 156-159 °C

IR (*v,* cm^{-1}) 3081, 2889-2813, 2219, 1526-1383, 1326-1038, 758-729, 685

^{1}H NMR (300 MHz, $CDCl_3$): δ (ppm) 8.55-8.52 (m, 2H), 7.52-7.43 (m, 5H), 2.63-2.59 (t, *J* = 6.9 Hz, 2H), 1.77-1.68 (m, 2H), 1.62-1.50 (m, 2H), 1.02-0.97 (t, *J* = 7.2 Hz, 3H)

^{13}C NMR (75 MHz, $CDCl_3$): δ (ppm) 169.5, 160.1, 146.0, 137.5, 130.6, 129.9, 128.6, 127.1, 120.8, 94.4, 78.1, 30.4, 22.3, 19.6, 13.8

HRMS-ESI (m/z): $[M+H]^+$ calc'd for $C_{18}H_{16}N_2S^+$, 293.1022; found, 293.1027

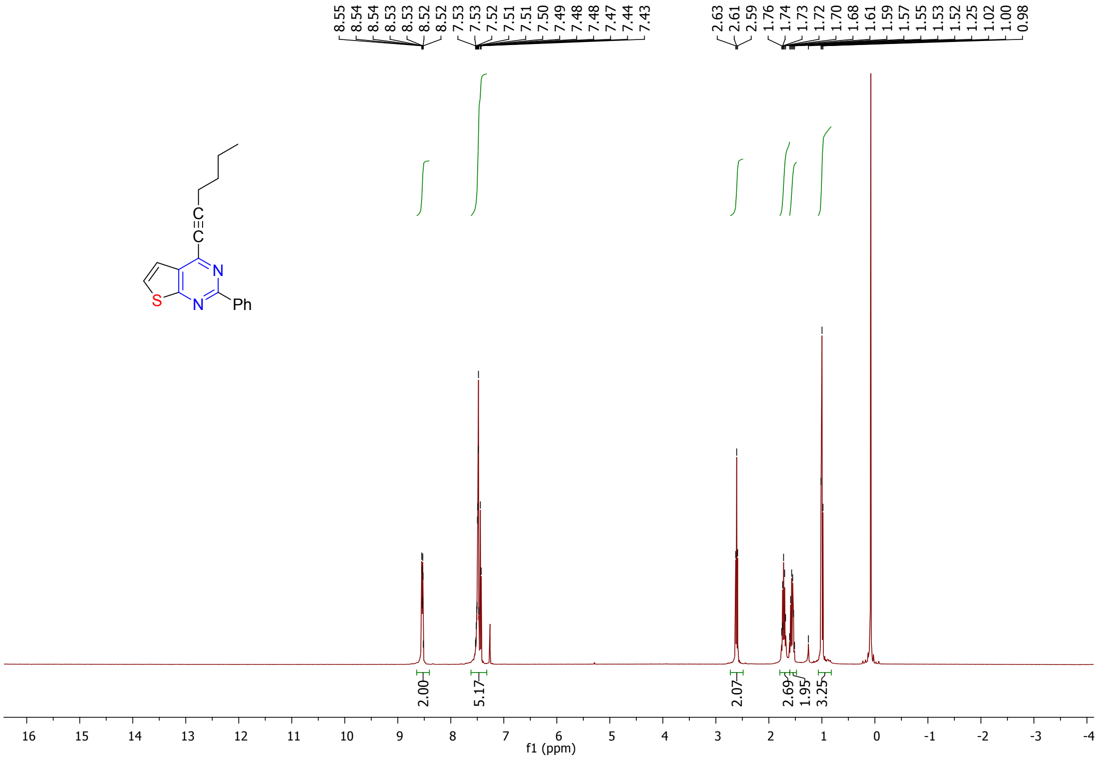

Fig. S-88: ^{1}HNMR Spectrum of **6c**

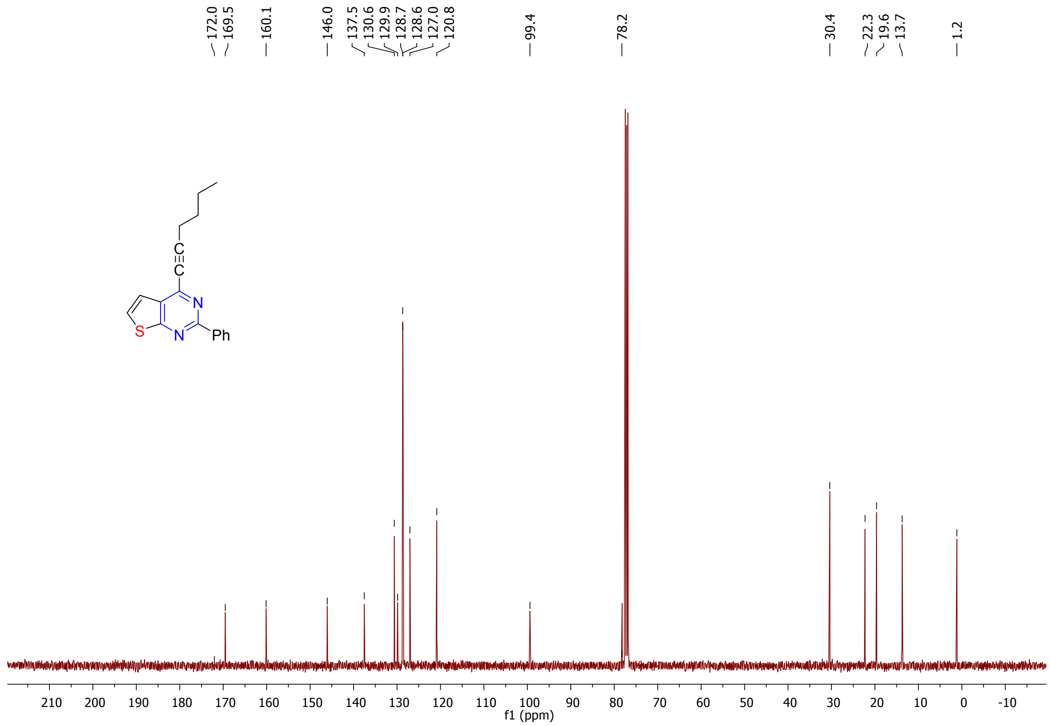

Fig. S-89: ^{13}C NMR Spectrum of **6c**

4-Methoxy-2-phenylthieno[2,3-*d*]pyrimidine (7a)

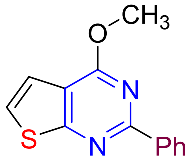

Yield: 68%

mp = 122-126 °C

IR (*v,* cm^{-1}) 3081-3020, 2983-2857, 1589-1368, 1317-1036, 922-659

^{1}H NMR [19] (300 MHz, $CDCl_3$): δ (ppm) 8.55-8.52 (m, 2H), 7.53-7.47 (m, 3H) 7.38-7.33 (m, 2H), 4.24 (s, 3H)

^{13}C NMR[19] (75 MHz, $CDCl_3$): δ (ppm) 169.7, 164.1, 159.9, 137.7, 130.5, 128.6, 128.5, 124.3, 118.7, 117.4, 53.9

GC-MS Analysis (m/z): M^+ = 242, 213, 103, 77, 51

HRMS-ESI (m/z): $[M+H]^+$ calc'd for $C_{13}H_{11}N_2OS^+$, 243.0614; found, 243.0619

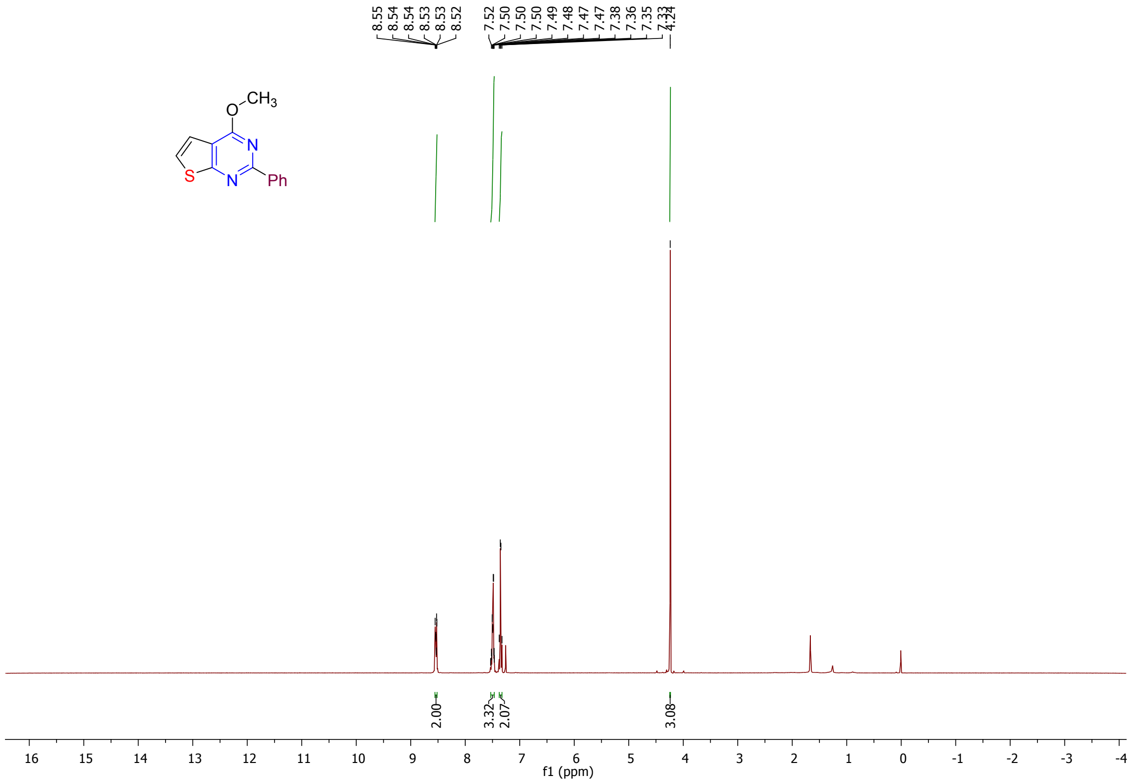

Fig. S-90: ^{1}HNMR Spectrum of **7a**

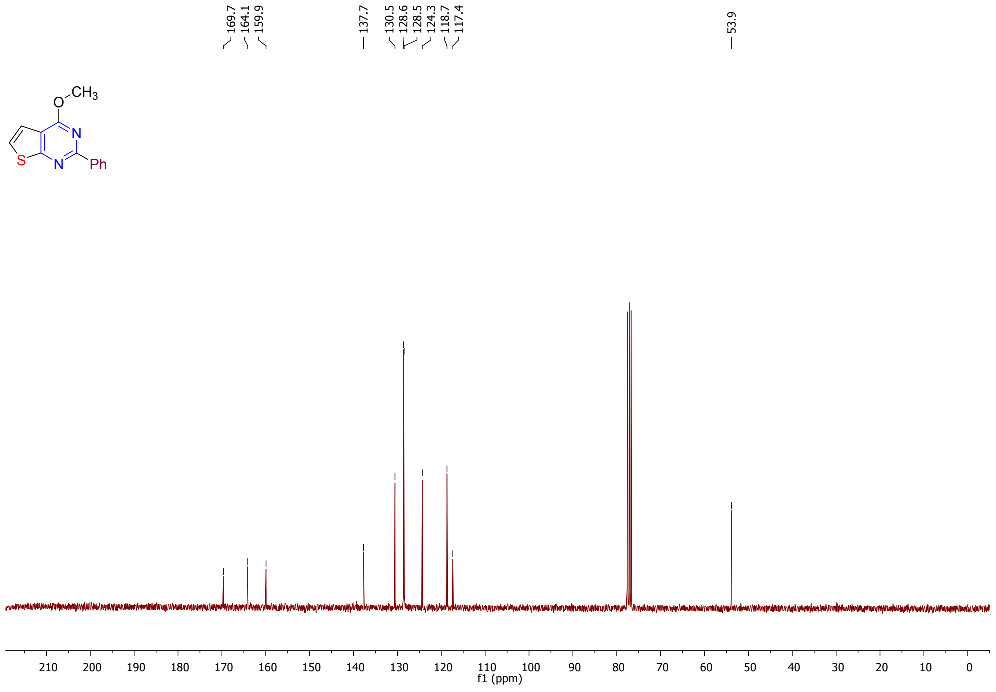

Fig. S-91: ^{13}C NMR Spectrum of **7a**

File :C:\MSDChem\1\DATA\2019\Dr.Abbas Hassan\Sania Batool\SB-39 16-09-19.D
Operator : Saqib Yasin
Instrument : Instrument #1
Acquired : 16 Oct 2019 12:16 using AcqMethod LIQUID 50 TO 500.M
Sample Name: SB-39
Misc Info : Temp 120-280 10 C/min flow 1.5ml/min Inj 5ul

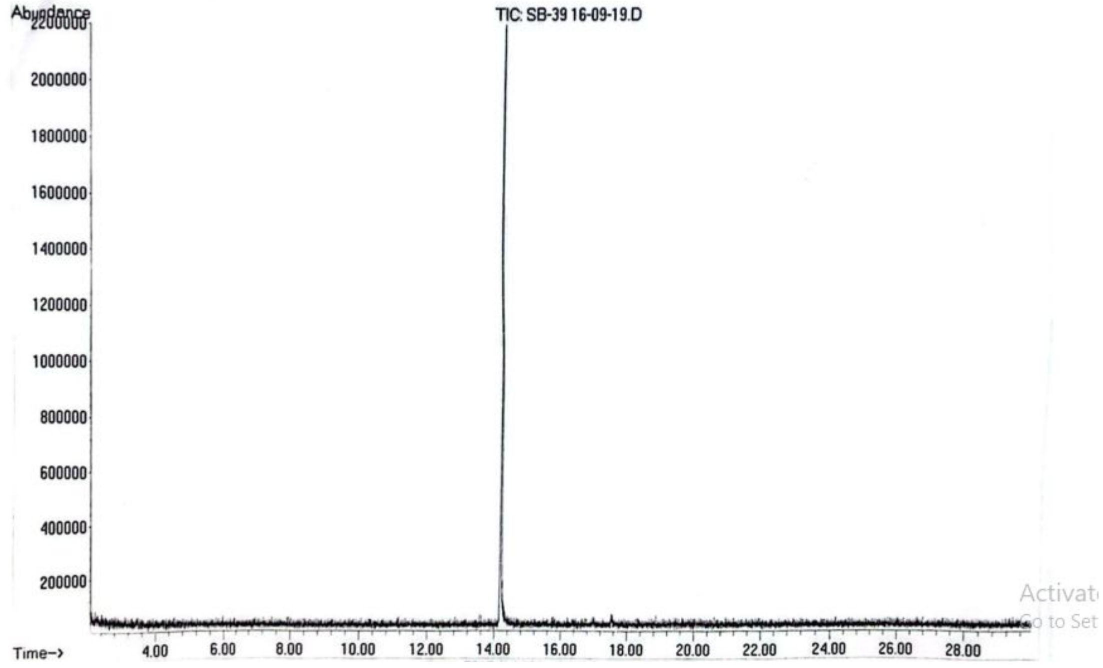

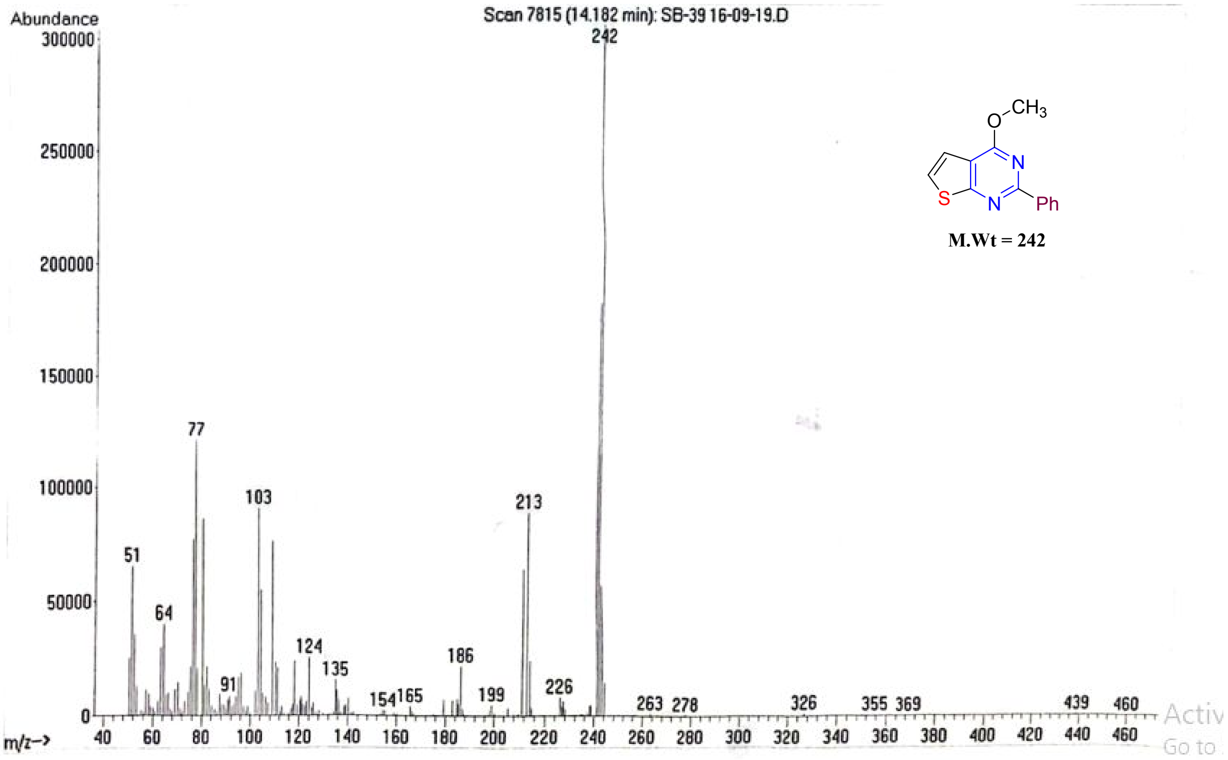

Fig. S-92: GCMS Spectrum of **7a**

3-Methyl-2-phenylthieno[2,3-*d*]pyrimidin-4(3*H*)-one (minor product)

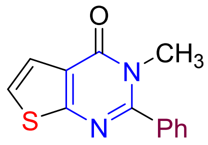

Yield: 21%

^{1}H NMR (300 MHz, $CDCl_3$) δ 7.58 – 7.51 (m, 1H), 7.51 – 7.49 (m, 1H), 7.24 (d, J = 5.8 Hz, 1H), 3.52 (s, 1H)

^{13}C NMR (75 MHz, $CDCl_3$) δ 163.3, 159.2, 156.4, 135.0, 130.4, 129.0, 128.2, 123.7, 122.6, 122.2, 34.4

GC-MS Analysis (m/z): M^+ = 242, 241, 118, 77, 51

HRMS-ESI (m/z): $[M+H]^+$ calc'd for $C_{13}H_{11}N_2OS^+$, 243.0515; found, 243.0518

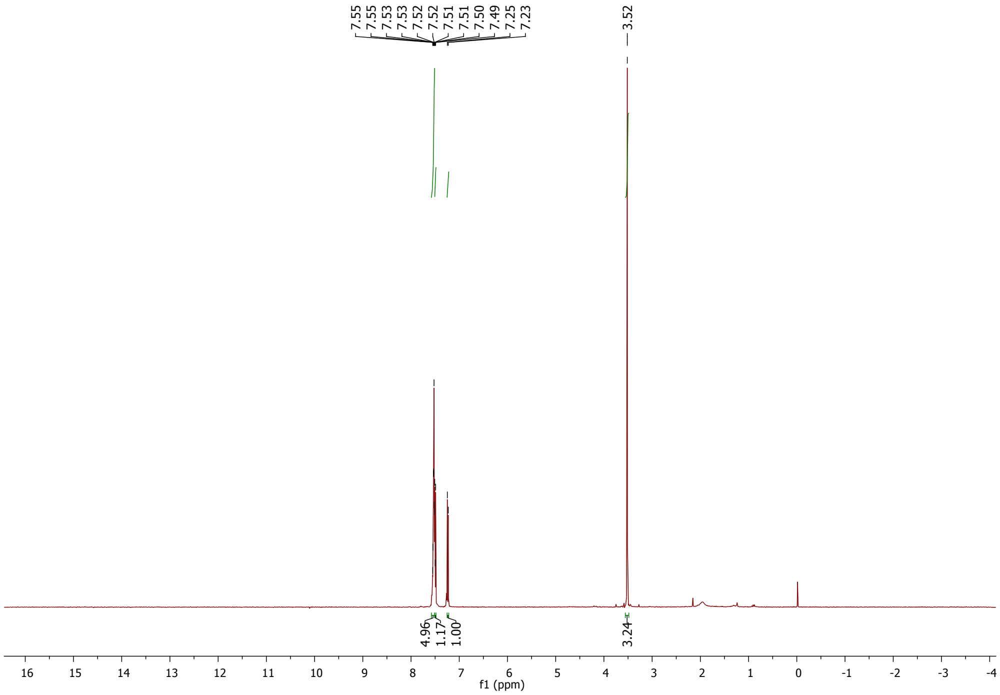

Fig. S-93: 1HNMR Spectrum of 3-Methyl-2-phenylthieno[2,3-d]pyrimidin-4(3H)-one

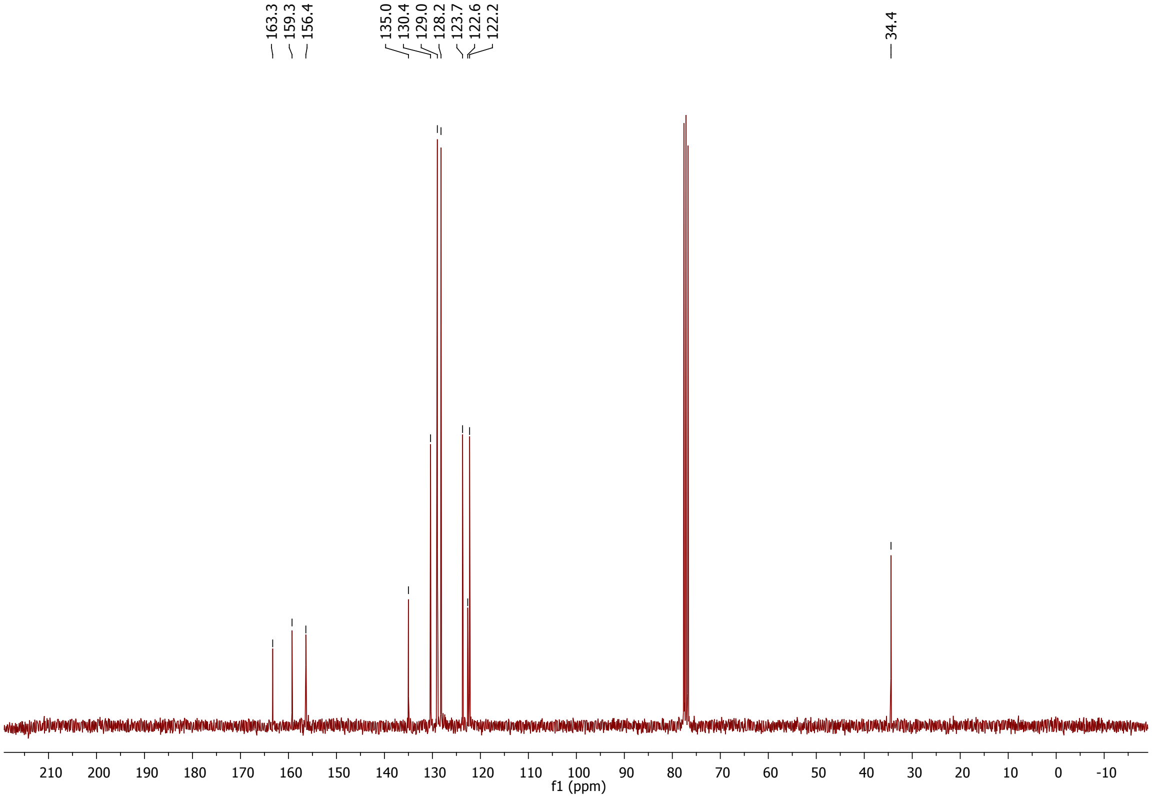

Fig. S-94: ^{13}C NMR Spectrum of 3-Methyl-2-phenylthieno[2,3-d]pyrimidin-4(3H)-one

File :C:\MSDChem\1\DATA\2019\Dr.Abbas Hassan\Sania Batool\SB-39-B 1
9-11-19.D
Operator : Saqib Yasin
Instrument : Instrument #1
Acquired : 19 Nov 2019 13:54 using AcqMethod LIQUID 50 TO 500.M
Sample Name: SB-39-B
Misc Info : temp 120-280C 10 C/min Flow 1.5ml/min inj 5ul

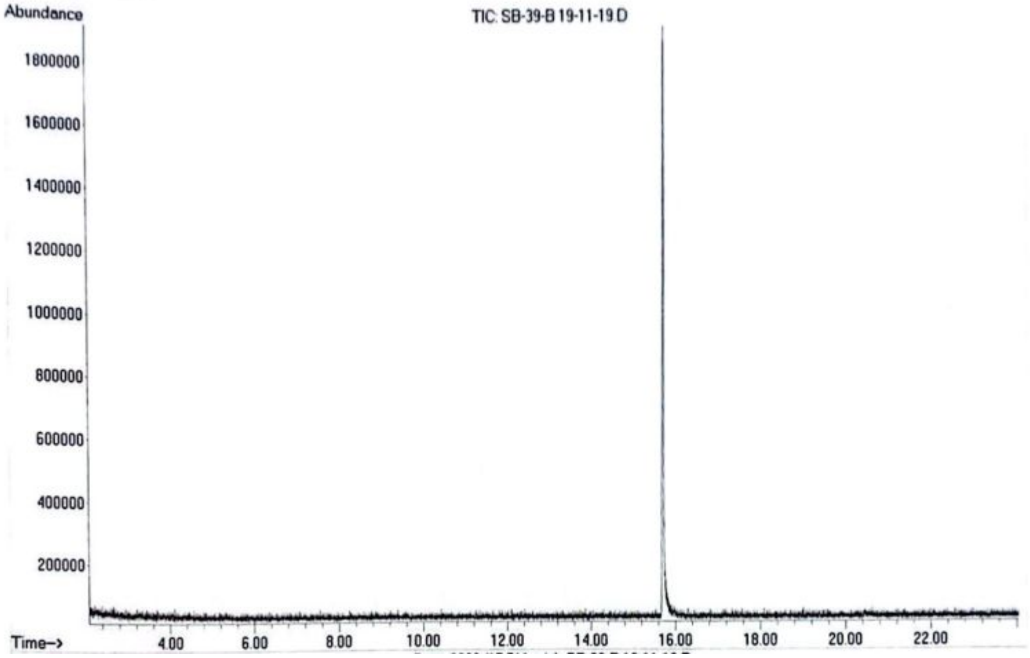

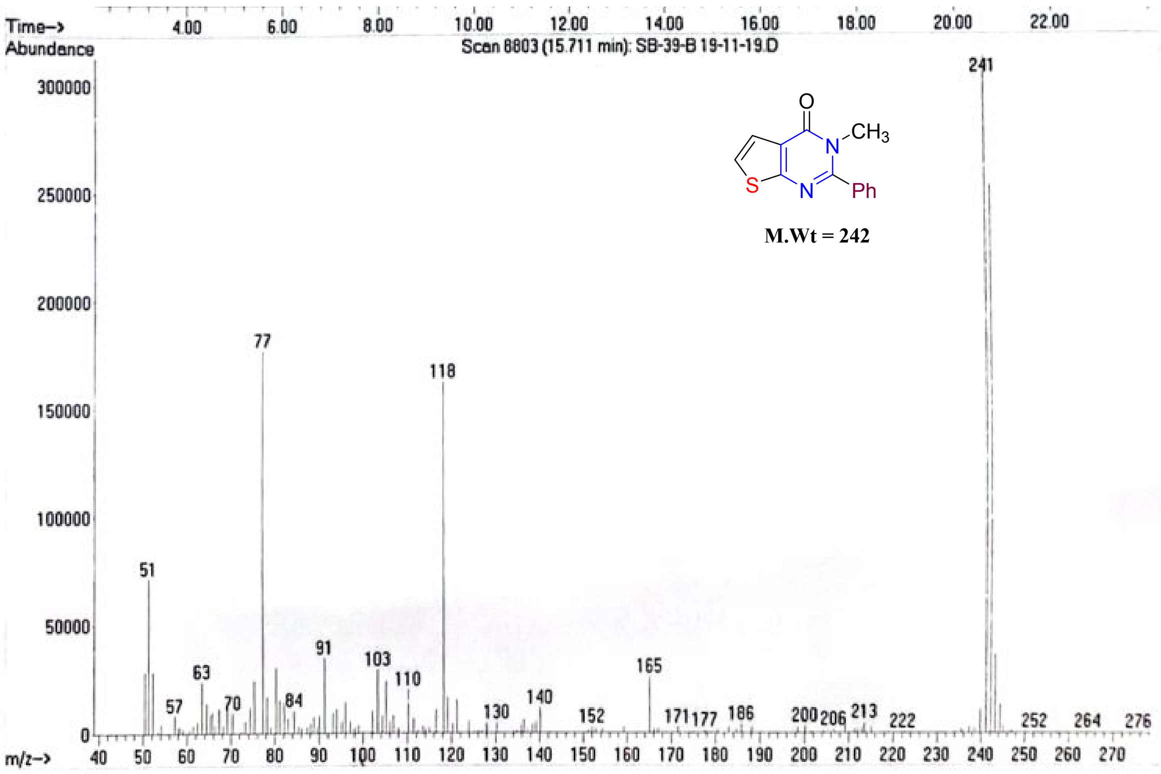

Fig. S-95: GCMS Spectrum of 3-Methyl-2-phenylthieno[2,3-d]pyrimidin-4(3H)-one

4-Ethoxy-2-phenylthieno[2,3-*d*]pyrimidine (7b)

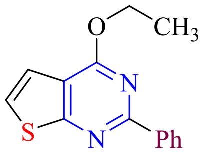

Yield: 66 %

mp = 127-129 °C

IR (*v*, cm^{-1}) 3096-3032, 2975-2924, 1585-1347, 1316-1032, 989-664

^{1}H NMR[20] (300 MHz, $CDCl_3$): δ (ppm) 8.53-8.50 (m, 2H), 7.53-7.46 (m, 3H) 7.39-7.32 (m, 2H), 4.78-4.71 (q, J = 6.0 Hz, 2H), 1.56-1.51 (t, J = 6.0 Hz, 3H)

^{13}C NMR[20] (75 MHz, $CDCl_3$): δ (ppm) 169.7, 163.8, 159.9, 137.9, 130.5, 128.6, 128.4, 124.1, 118.8, 117.5, 62.7, 14.4

GC-MS Analysis (m/z): M^+ = 256, 241, 228, 211, 125, 104, 77, 51

HRMS-ESI (m/z): $[M+H]^+$ calc'd for $C_{14}H_{13}N_2OS^+$, 257.0871; found, 257.0875

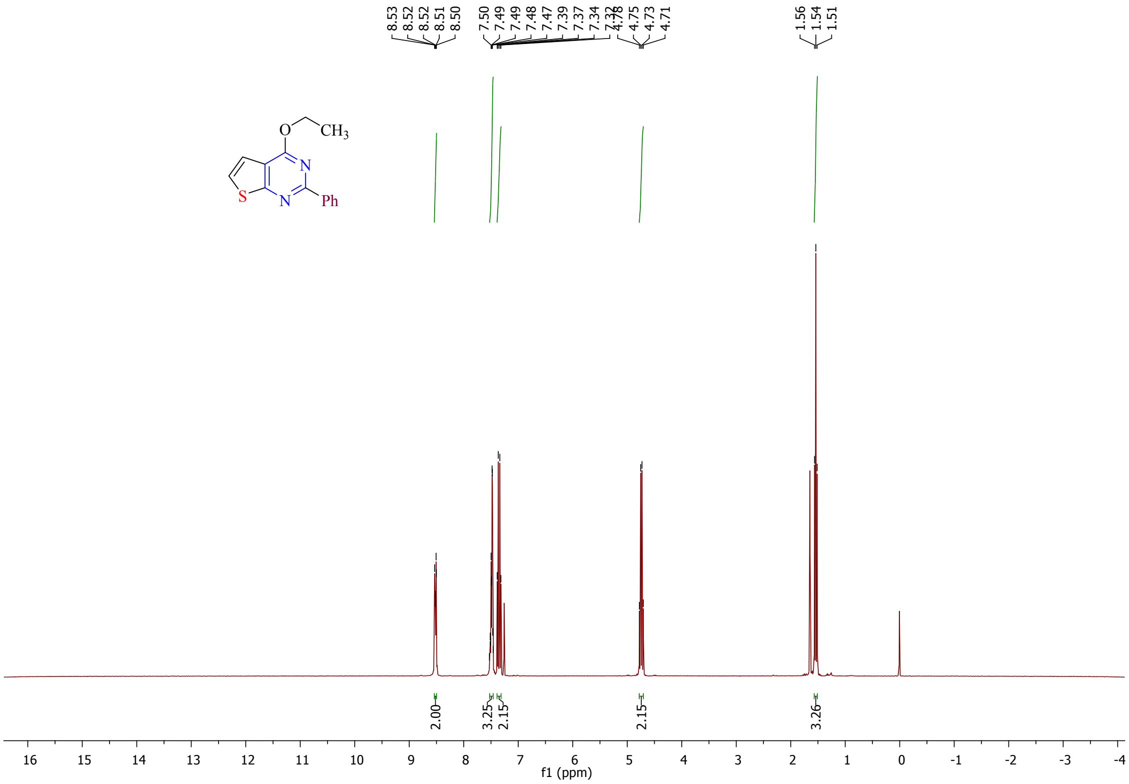

Fig. S-96: ^{1}HNMR Spectrum of **7b**

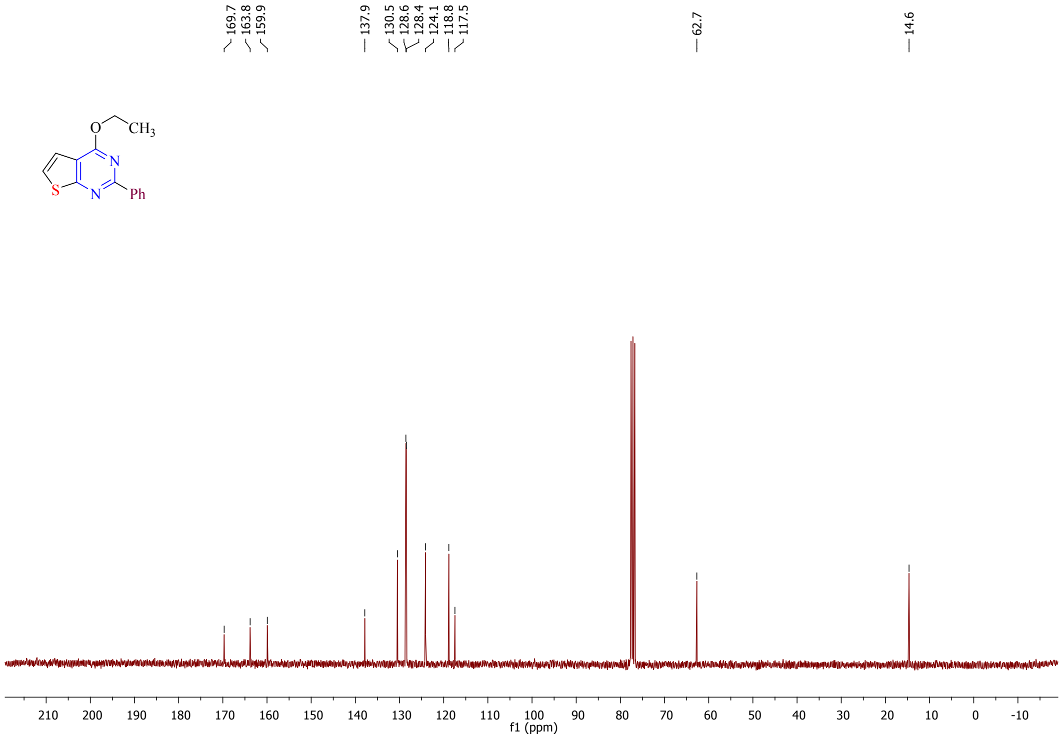

Fig. S-97: ^{13}C NMR Spectrum of **7b**

```
File         :C:\MSDChem\1\DATA\2019\Dr.Abbas Hassan\Sania Batool\SB-40 16-
...           09-19.D
Operator     : Saqib Yasin
Instrument   :     Instrument #1
Acquired     : 16 Oct 2019  12:53     using AcqMethod LIQUID 50 TO 500.M
Sample Name: SB-40
Misc Info    : Temp 120-280 10 C/min flow 1.5ml/min  Inj 5ul
```

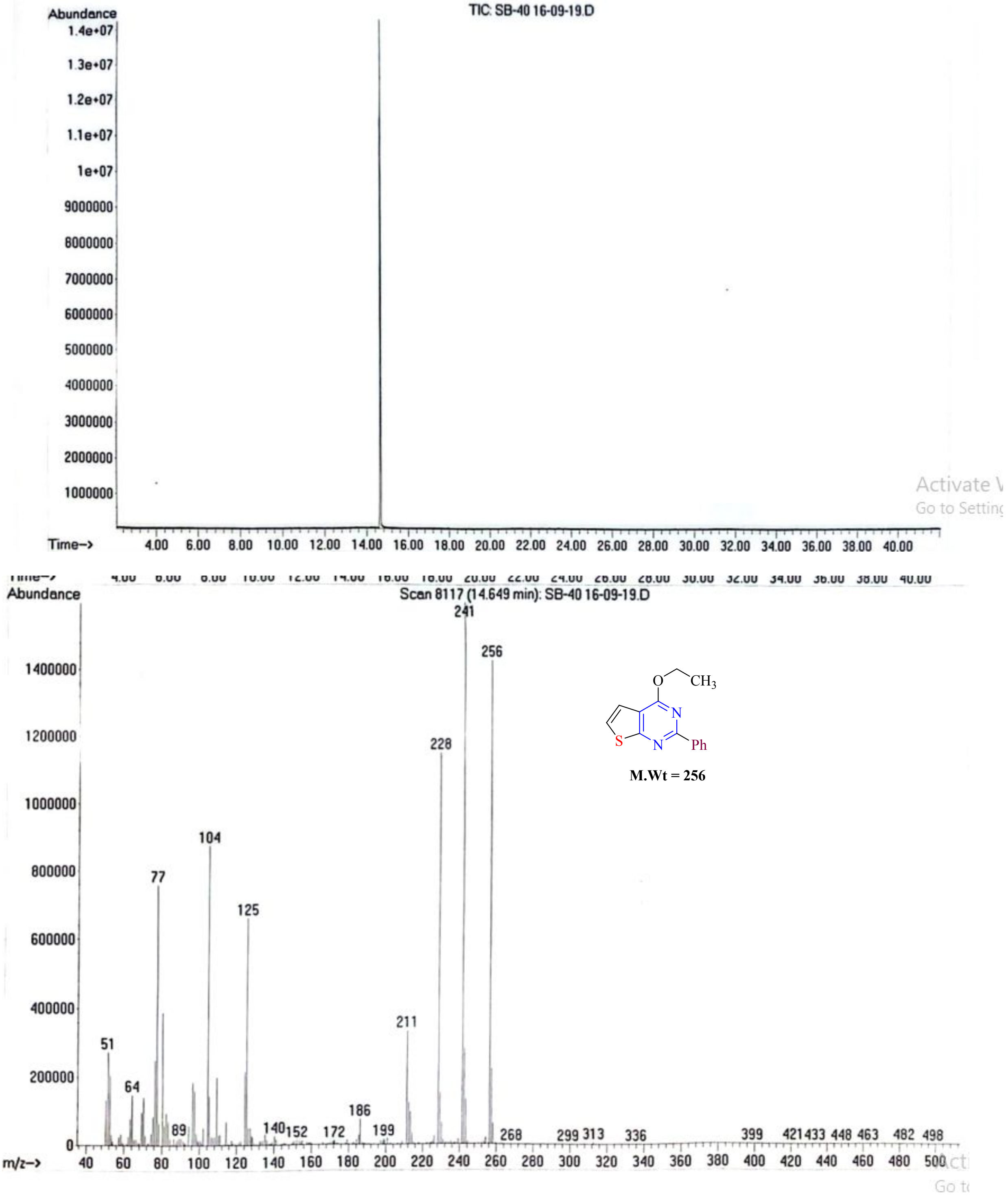

Fig. S-98: GCMS Spectrum of **7b**

4-Isobutoxy-2-phenylthieno[2,3-*d*]pyrimidine (7c)

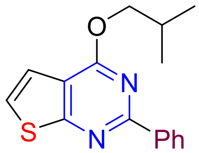

Yield: 62%

mp = 123-127 ˚C

IR (*v,* cm^{-1}) 3095-3058, 2921-2852, 1524-1398, 1356-1083, 712-670

^{1}H NMR (300 MHz, $CDCl_3$): δ (ppm) 8.53-8.50 (m, 2H), 7.53-7.46 (m, 3H) 7.40-7.38 (d, *J* = 6.0 Hz, 1H), 4.47-4.44 (d, *J* = 9.0 Hz, 2H), 2.32-2.19 (sep.,1H), 1.12-1.10 (d, *J* = 6.0 Hz, 6H)

^{13}C NMR (75 MHz, $CDCl_3$): δ (ppm) 169.7, 164.1, 160.0, 137.8, 130.5, 128.6, 128.4, 124.1, 118.8, 117.5, 72.8, 28.1, 19.5

GC-MS Analysis (m/z): M^+ = 284, 228, 125, 104, 77, 51

HRMS-ESI (m/z): $[M+H]^+$ calc'd for $C_{16}H_{17}N_2OS^+$, 285.0982; found, 285.0986

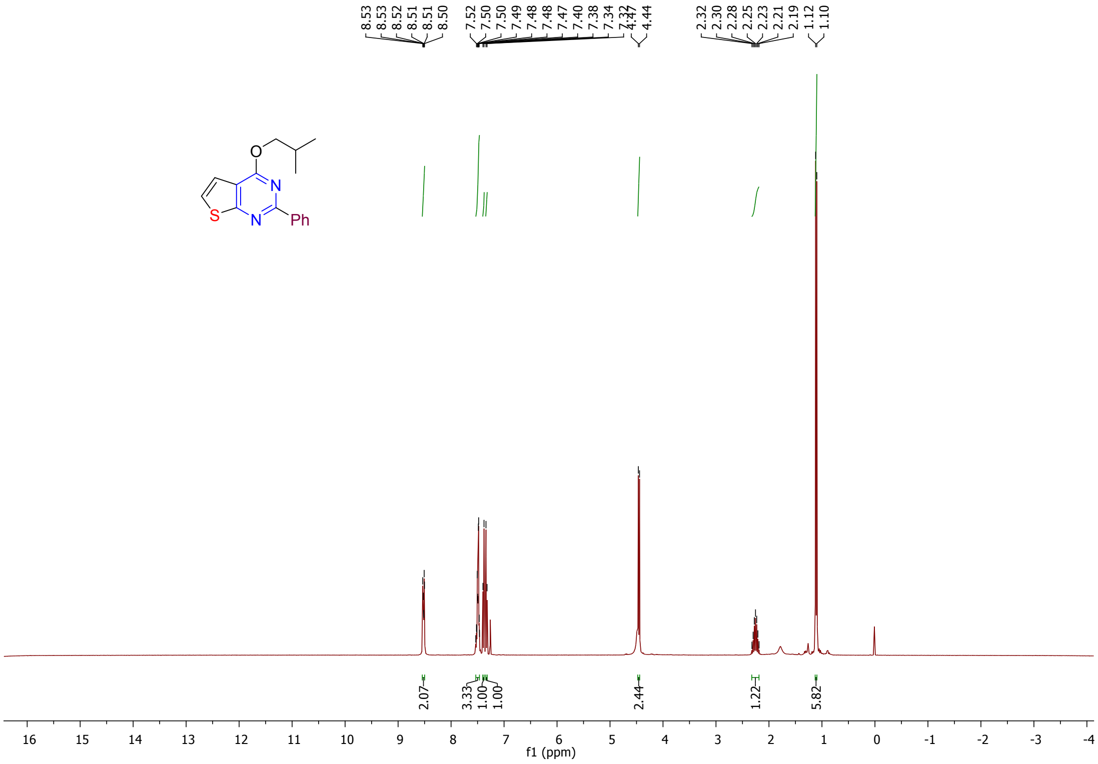

Fig. S-99: ^{1}HNMR Spectrum of **7c**

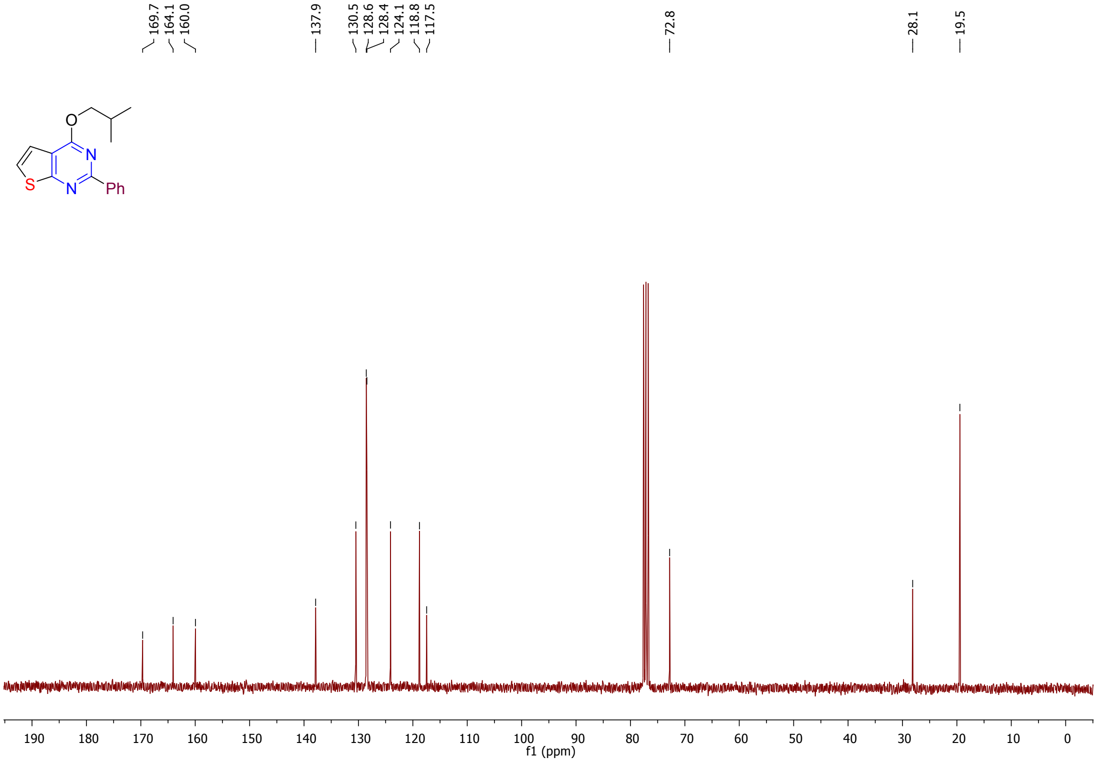

Fig. S-100: ^{13}C NMR Spectrum of **7c**

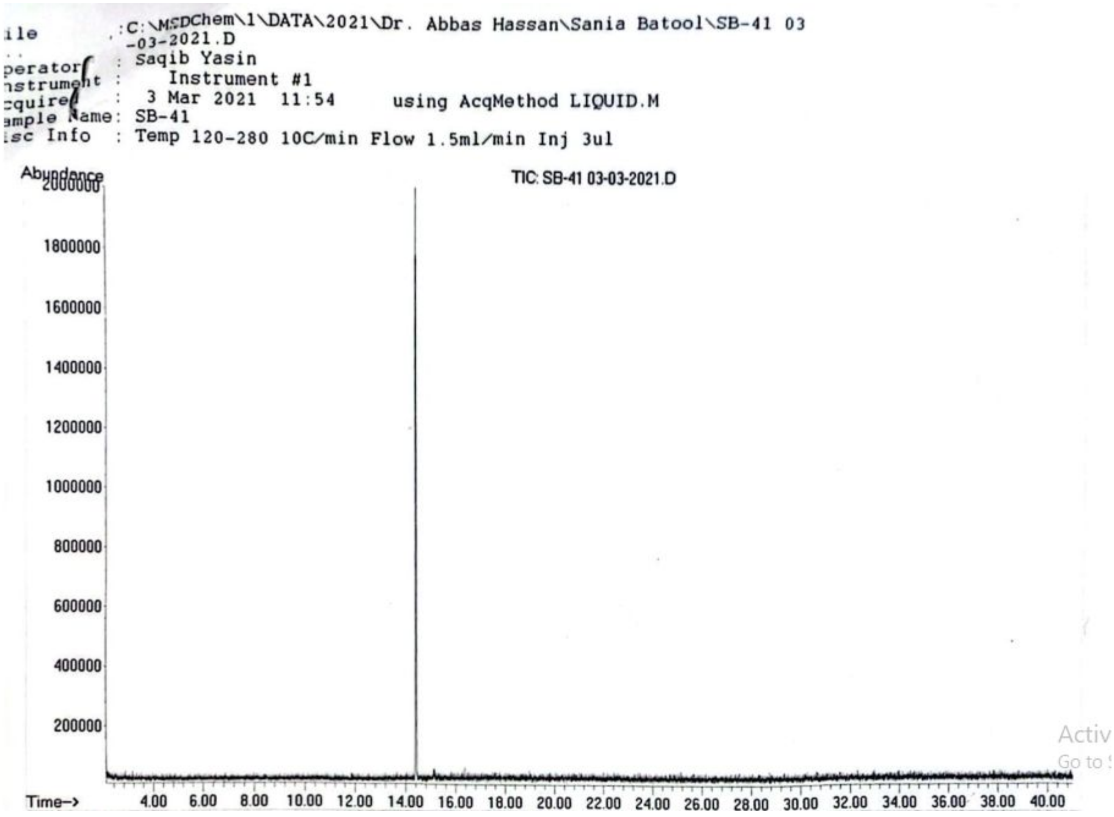

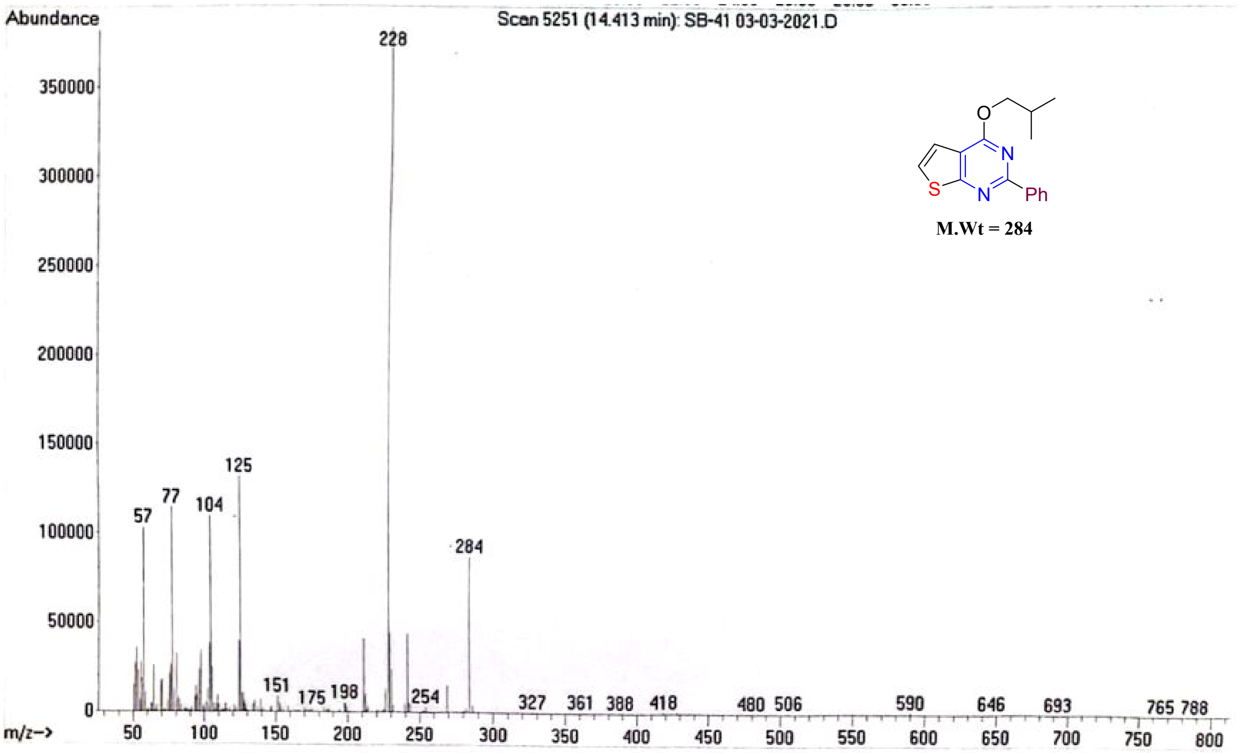

Fig. S-101: GCMS Spectrum of **7c**

4-(Allyloxy)-2-phenylthieno[2,3-*d*]pyrimidine (7d)

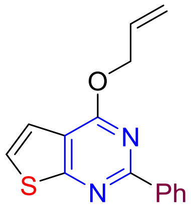

Yield: 70%

mp = 132-135 °C

IR (*v,* cm^{-1}) 3074-3028, 2920-2849, 1660-1421, 1365-1010, 918-652

^{1}H NMR (300 MHz, $CDCl_3$): δ (ppm) 8.53-8.50 (m, 2H), 7.53-7.48 (m, 3H), 7.41-7.39 (d, *J* = 6.0 Hz, 1H), 7.35-7.33 (d, *J* = 6.0 Hz, 1H), 6.28-6.15 (m, 1H), 5.56-5.48 (m, 1H), 5.37-5.33 (dd, *J* = 9.0 Hz 1H), 5.21-5.18 (dt, *J* = 6.0 Hz, 3.0 Hz, 2H)

^{13}C NMR (75 MHz, $CDCl_3$): δ (ppm) 169.9, 163.4, 159.8, 137.7, 132.8, 130.6, 128.6, 128.5, 124.4, 118.8, 118.7, 117.4, 67.3

GC-MS Analysis (m/z): M^+ = 268, 267, 253, 211, 191, 104, 77, 51

HRMS-ESI (m/z): $[M+H]^+$ calc'd for $C_{15}H_{13}N_2OS^+$, 269.0716; found, 269.0718

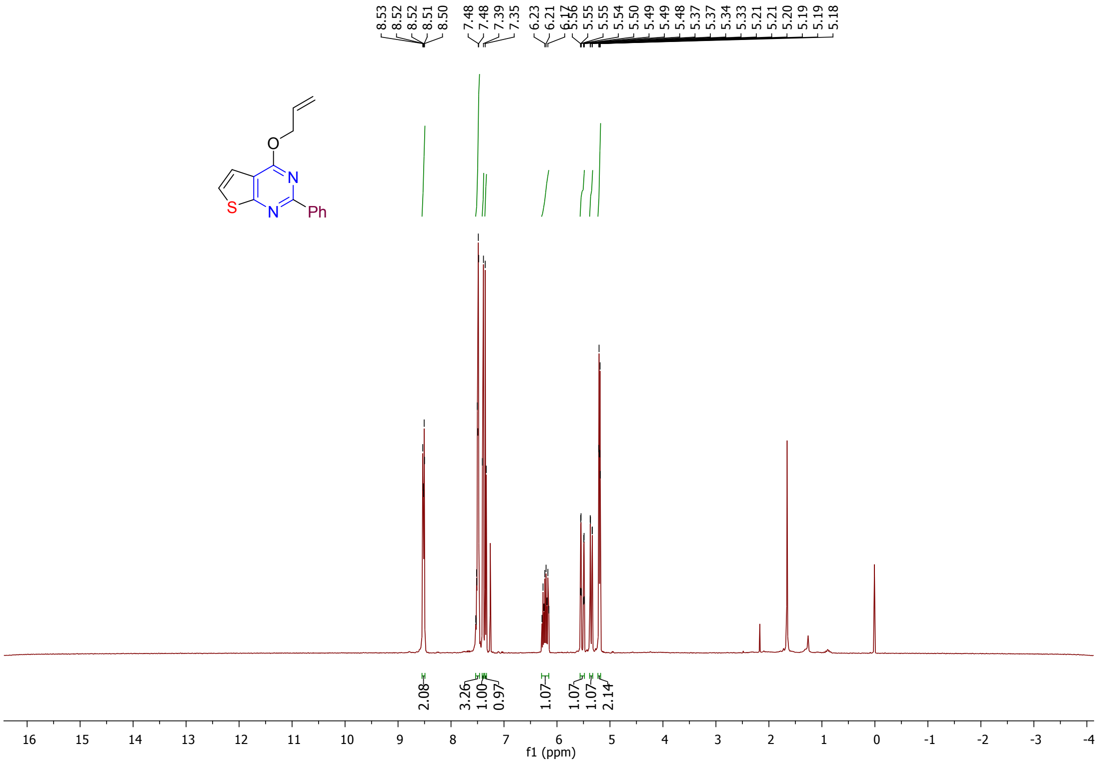

Fig. S-102: ^{1}HNMR Spectrum of **7d**

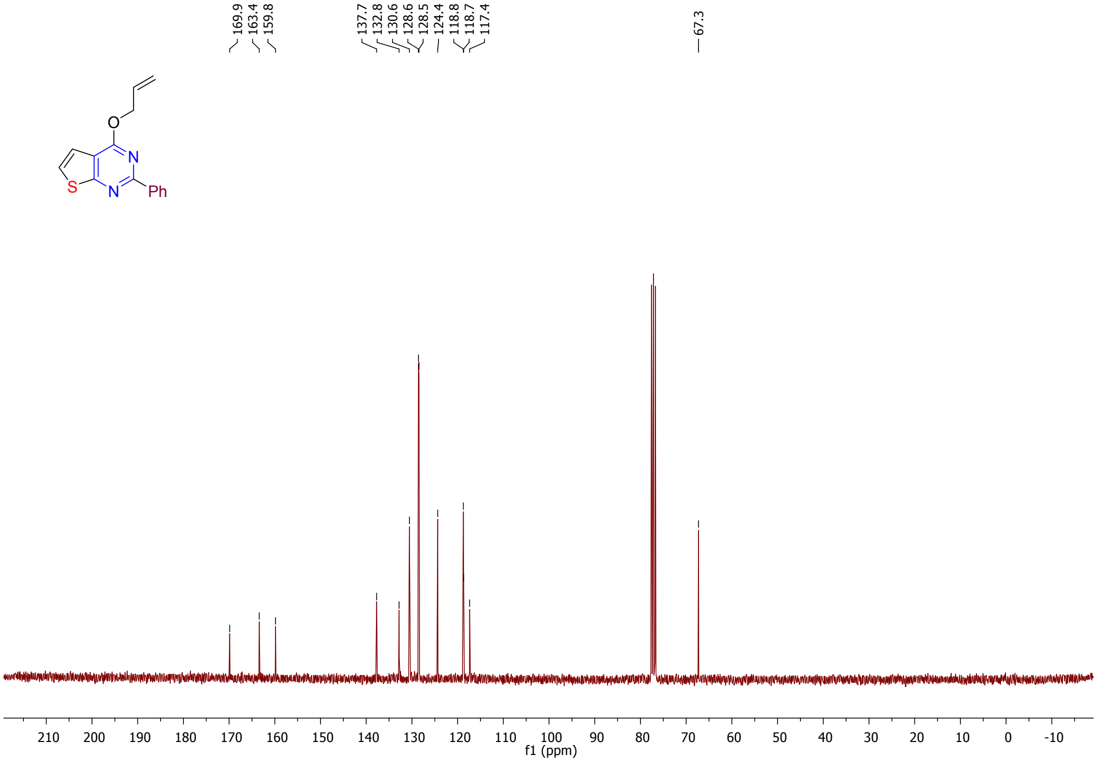

Fig. S-103: ^{13}C NMR Spectrum of **7d**

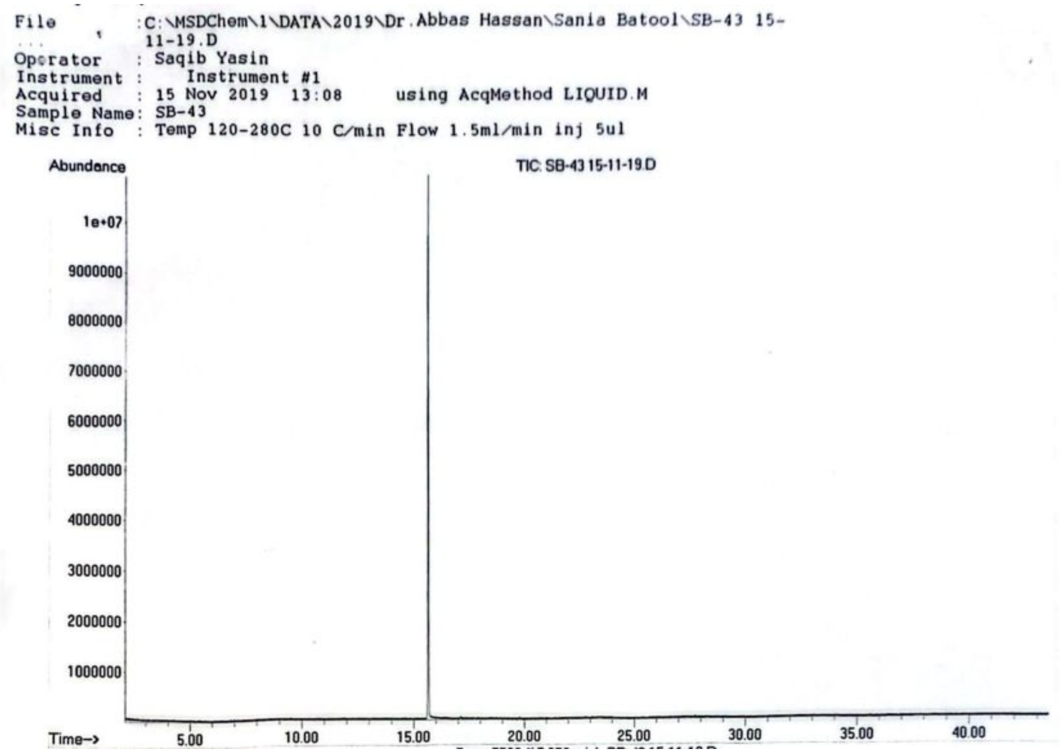

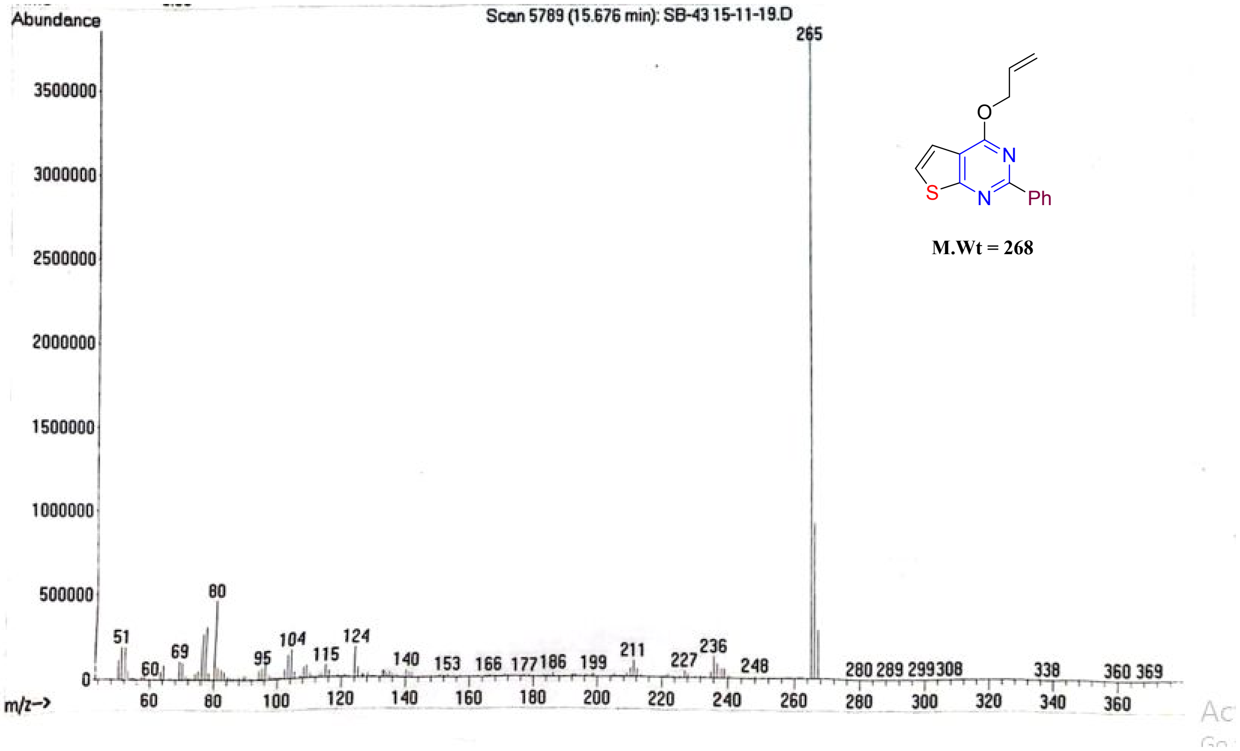

Fig. S-104: GCMS Spectrum of **7d**

2-Phenyl-4-(prop-2-yn-1-yloxy)thieno[2,3-*d*]pyrimidine (7e)

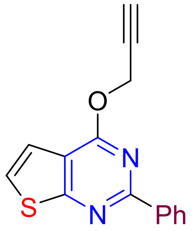

Yield: 65 %

mp = 144-147 °C

IR (*v,* cm^{-1}) 3207-3105, 2923-2852, 2120, 1589-1395, 1340-1024, 945-665

^{1}H NMR (300 MHz, $CDCl_3$): δ (ppm) 8.55-8.51 (m, 2H), 7.54-7.47 (m, 3H) 7.42-7.40 (d, *J* = 6.0 Hz, 1H), 7.38-7.36 (d, *J* = 6.0 Hz, 1H), 5.31-5.30 (*ap.* d, *J* = 3.0 Hz, 2H), 2.57-2.55 (t, *J* = 3.0 Hz, 1H)

^{13}C NMR (75 MHz, $CDCl_3$): δ (ppm) 170.1, 162.5, 159.6, 137.4, 130.7, 128.6, 128.5, 124.8, 118.6, 117.2, 78.4, 75.2, 54.0

GC-MS Analysis (m/z): M^+ = 266, 265, 211, 77, 51

HRMS-ESI (m/z): $[M+H]^+$ calc'd for $C_{15}H_{11}N_2OS^+$, 267.0526; found, 267.0529

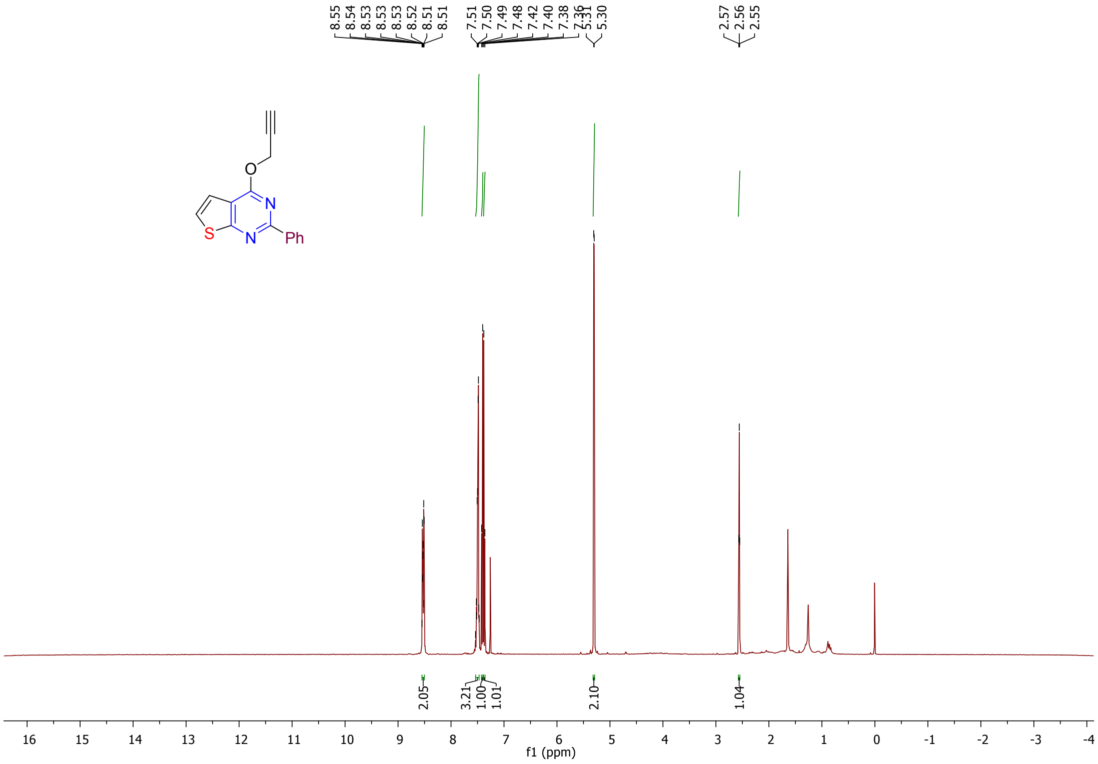

Fig. S-105: ^{1}HNMR Spectrum of **7e**

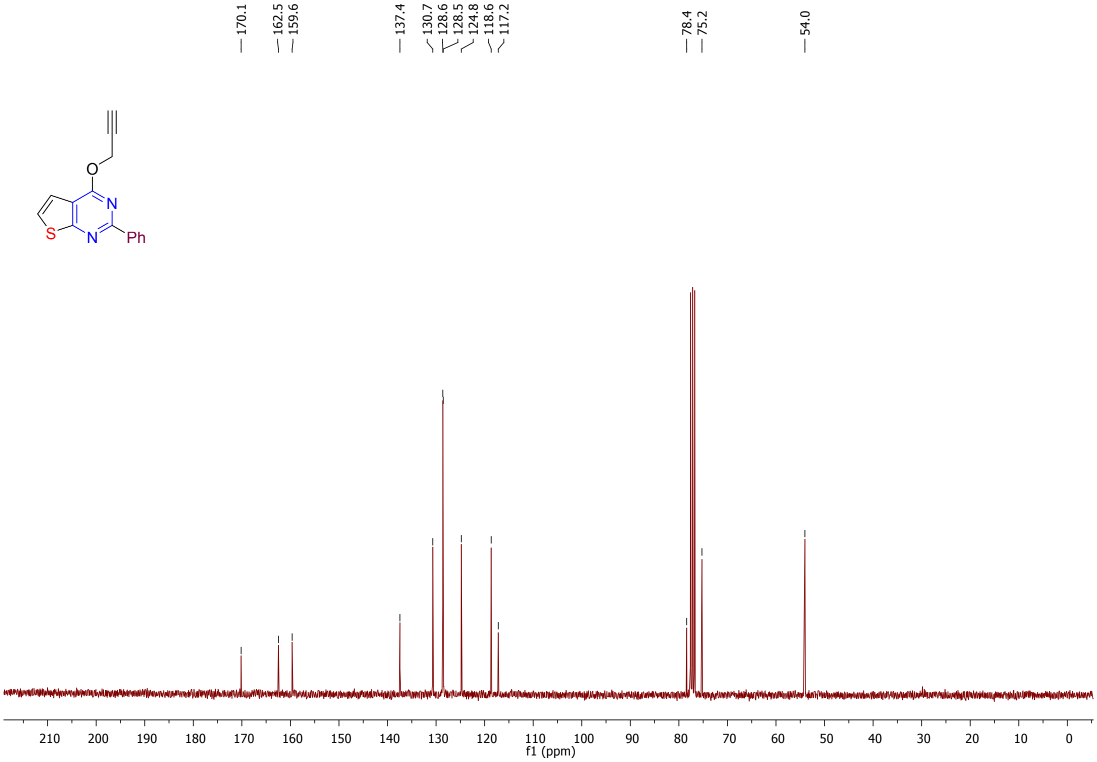

Fig. S-106: ^{13}C NMR Spectrum of **7e**

File :C:\MSDChem\1\DATA\2019\Dr.Abbas Hassan\Sania Batool\SB-44 19-11-19.D
Operator : Saqib Yasin
Instrument : Instrument #1
Acquired : 19 Nov 2019 10:22 using AcqMethod LIQUID 50 TO 500.M
Sample Name: SB-44
Misc Info : temp 120-280C 10 C/min Flow 1.5ml/min inj 5ul

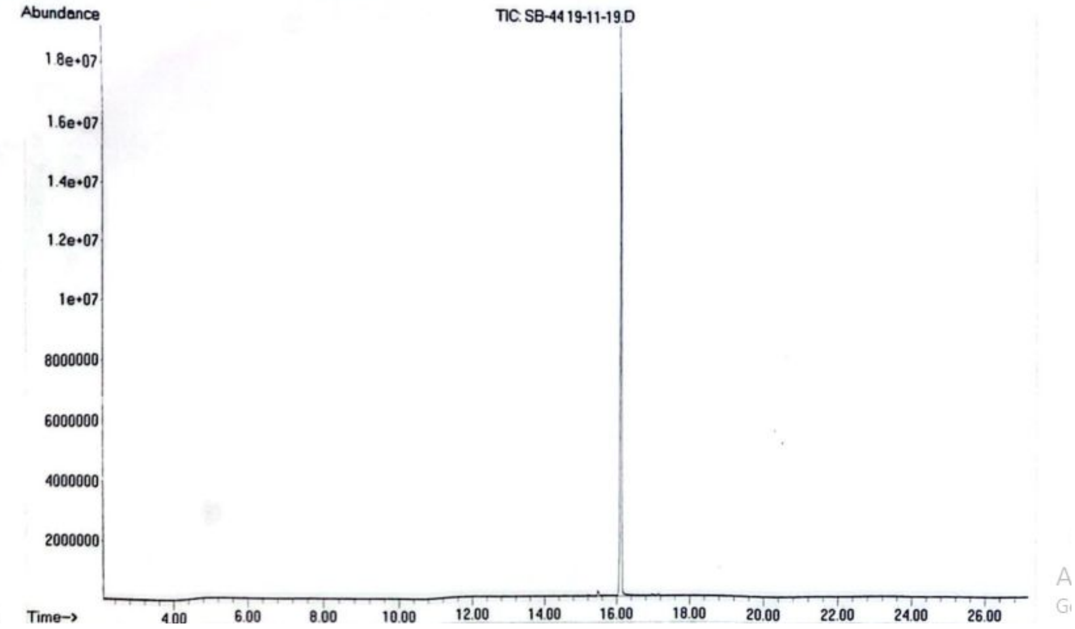

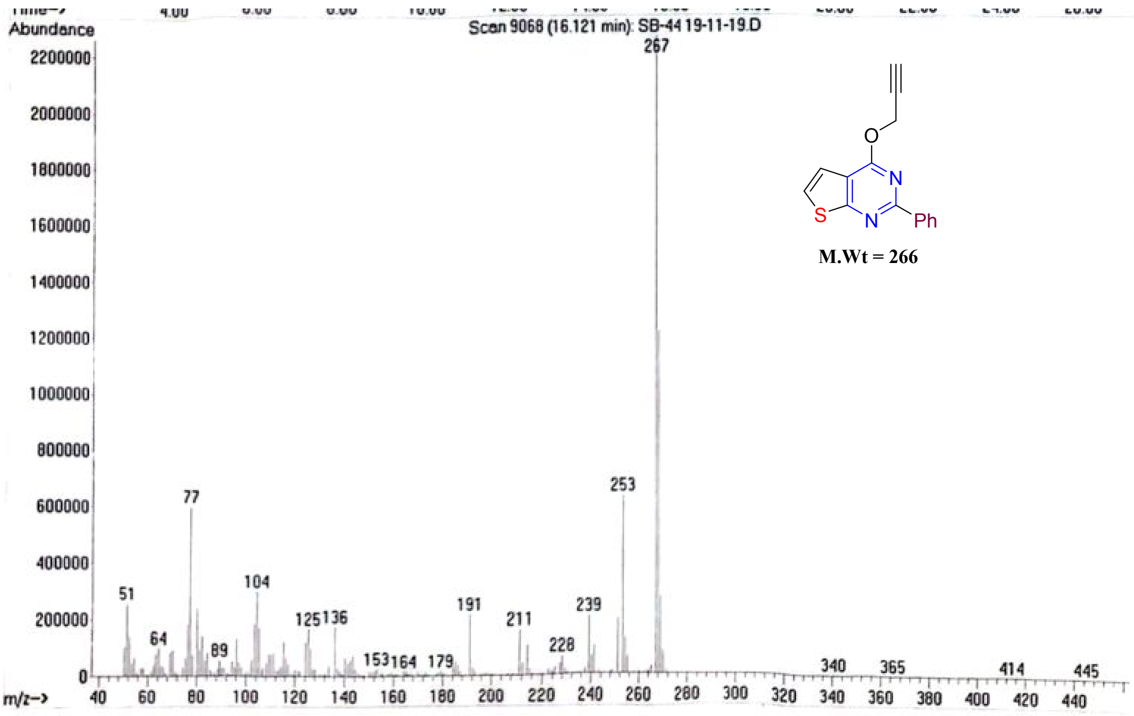

Fig. S-107: GCMS Spectrum of **7e**

4-(Benzyloxy)-2-phenylthieno[2,3-*d*]pyrimidine (7f)

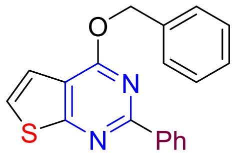

Yield: 72%

mp = 147-149 ˚C

IR (*v,* cm^{-1}) 2923, 1315-1005, 1563-1397, 777-663

^{1}H NMR (300 MHz, $CDCl_3$): δ (ppm) 8.56-8.53 (m, 2H), 7.58-7.49 (m, 2H) 7.57-7.52 (m, 5H), 7.45-7.33 (m, 5H), 5.75 (2H, s)

^{13}C NMR (75 MHz, $CDCl_3$): δ (ppm) 169.9, 163.6, 159.8, 137.7, 136.6, 130.6, 128.7, 128.6, 128.5, 128.4, 124.4, 118.7, 117.4, 68.2

GC-MS Analysis (m/z): M^+ = 318, 91, 65

HRMS-ESI (m/z): $[M+H]^+$ calc'd for $C_{19}H_{15}N_2OS^+$, 319.0837; found, 319.0841

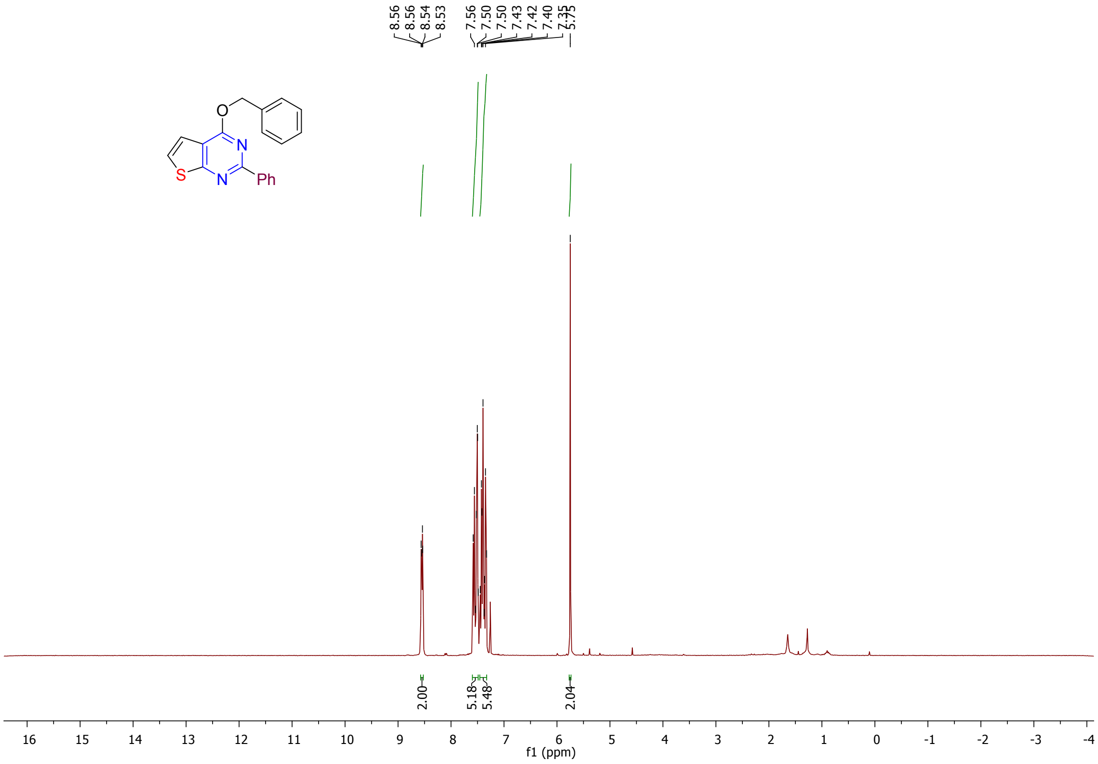

Fig. S-108: ^{1}HNMR Spectrum of **7f**

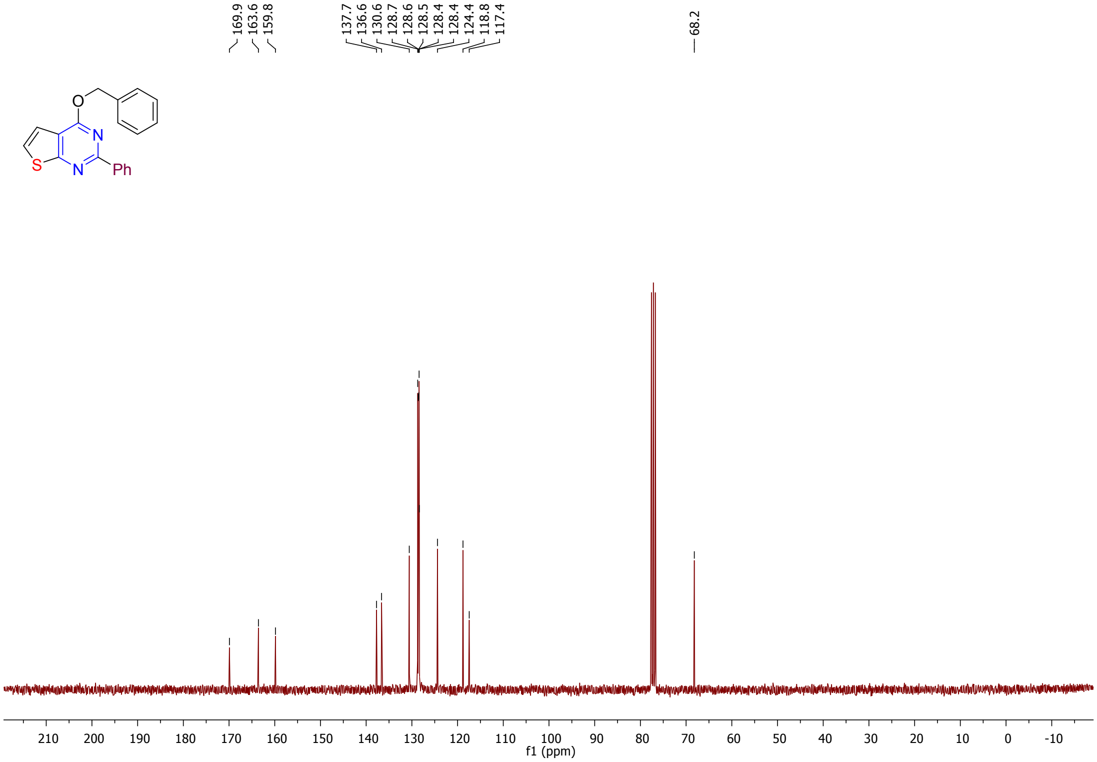

Fig. S-109: ^{13}C NMR Spectrum of **7f**

File :C:\MSDChem\1\DATA\2019\Dr.Abbas Hassan\Sania Batool\SB-32 03-10-19.D
Operator : Saqib Yasin
Instrument : Instrument #1
Acquired : 3 Oct 2019 10:02 using AcqMethod LIQUID.M
Sample Name: SB-32
Misc Info : temp 120-280 10 C/min flow 1.5ml/min inj 5ul

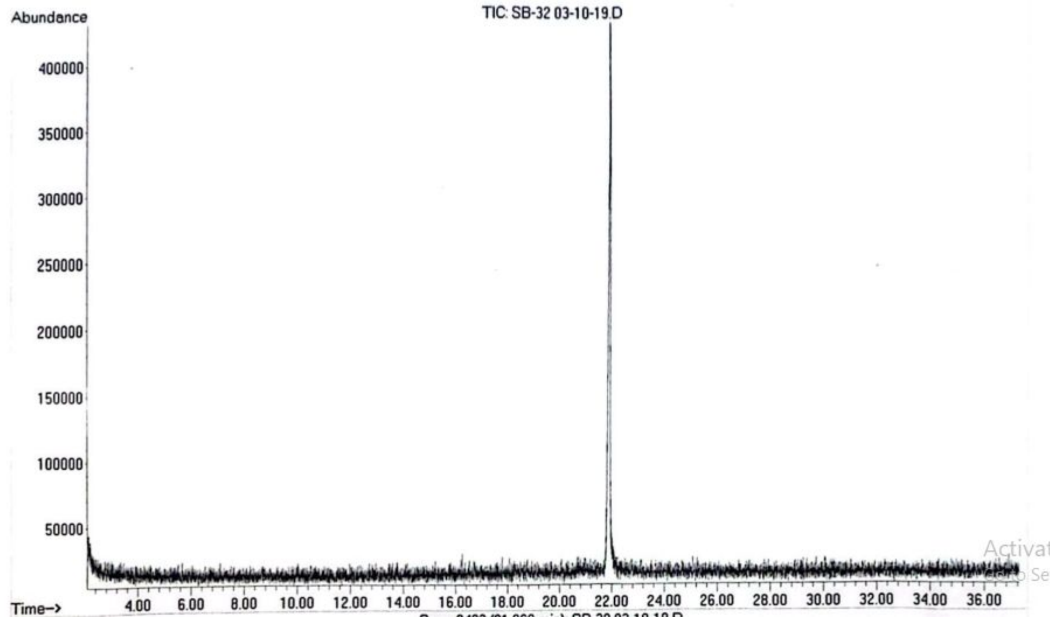

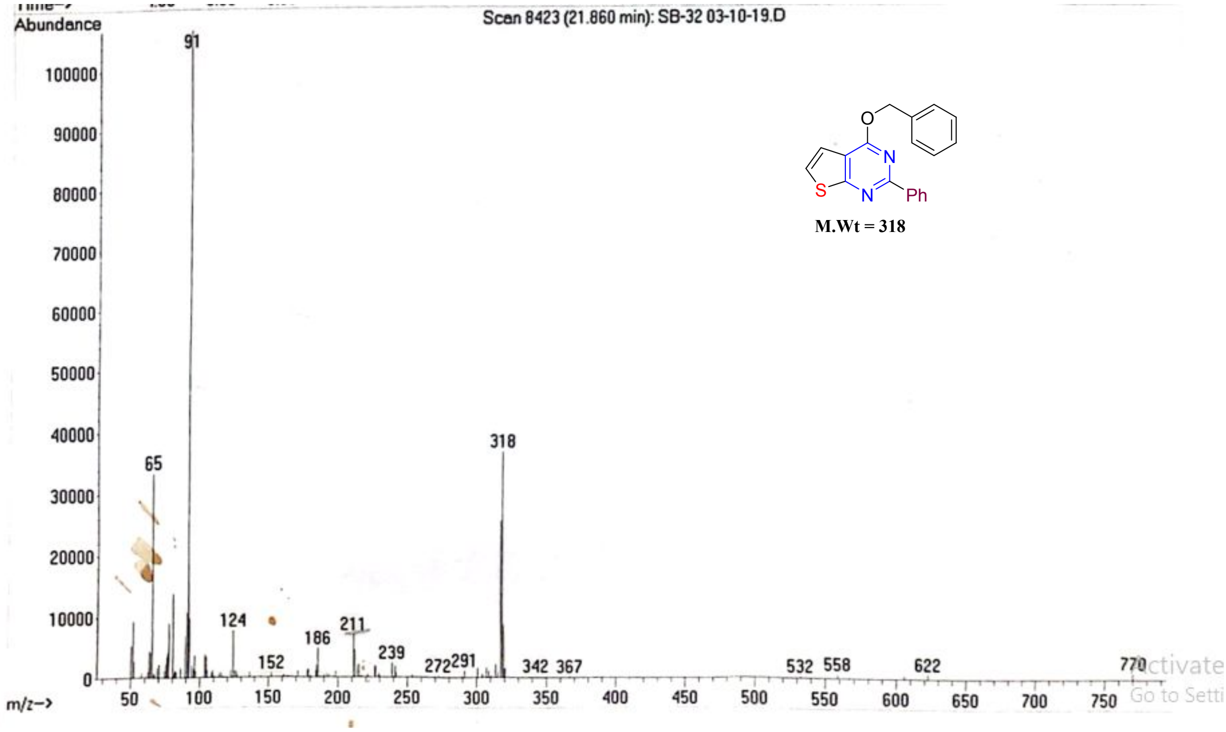

Fig. S-110: GCMS Spectrum of **7f**

4-((2-Phenylthieno[2,3-*d*]pyrimidin-4-yl)oxy)butanenitrile (7g)

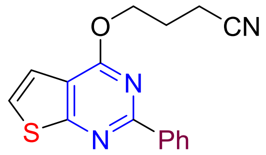

Yield: 80%

mp = 127-130 °C

IR (*v,* cm^{-1}) 3104-3063, 2969-2890, 2254, 1585-1398, 1348-1035, 995-662

^{1}H NMR (300 MHz, $CDCl_3$): δ (ppm) 8.51-8.48 (m, 2H), 7.53-7.46 (m, 3H) 7.39-7.35 (m, 2H), 4.81-4.77 (t, J = 6.0 Hz, 2H), 2.65-2.60 (t, J = 6.0 Hz, 2H), 2.34-2.25 (quint. J = 6.0 Hz, 2H)

^{13}C NMR (75 MHz, $CDCl_3$): δ (ppm) 170.0, 163.2, 159.8, 137.5, 130.7, 128.6, 128.4, 124.8, 119.3, 118.5, 117.2, 64.4, 25.2, 14.6

GC-MS Analysis (m/z): M^+ = 295, 255, 241, 228, 211, 125, 104, 77, 51

HRMS-ESI (m/z): $[M+H]^+$ calc'd for $C_{16}H_{14}N_3OS^+$, 296.0785; found, 296.0788

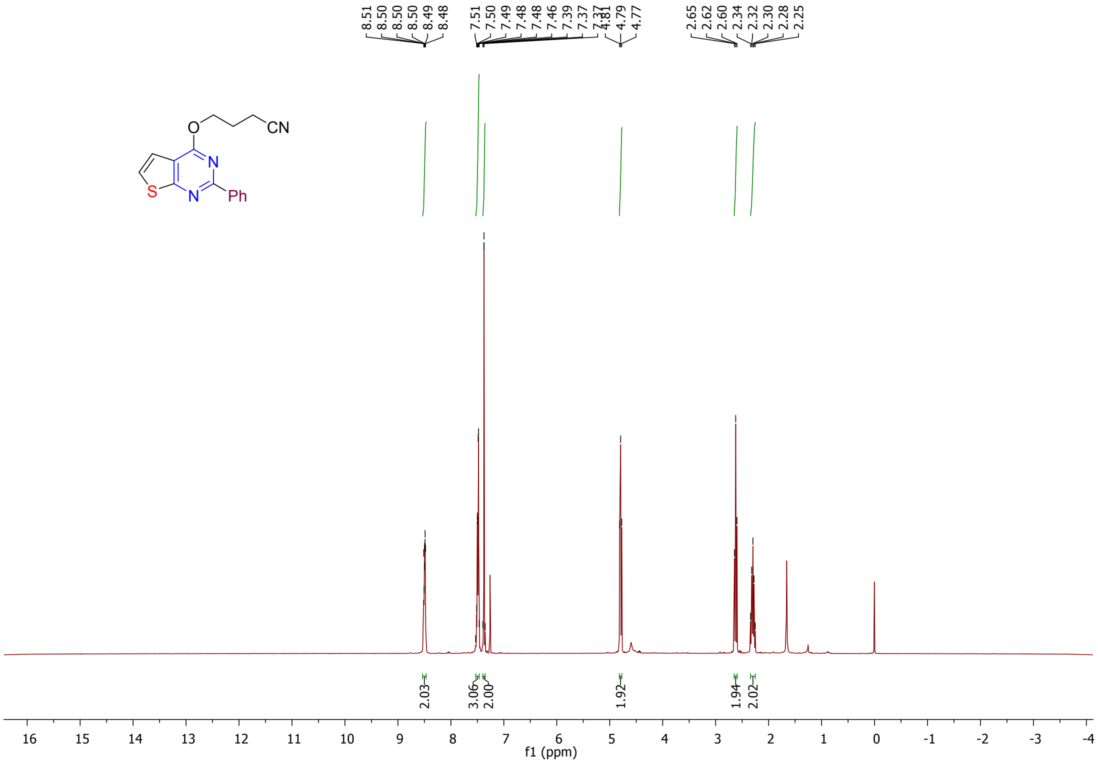

Fig. S-111: ^{1}HNMR Spectrum of **7g**

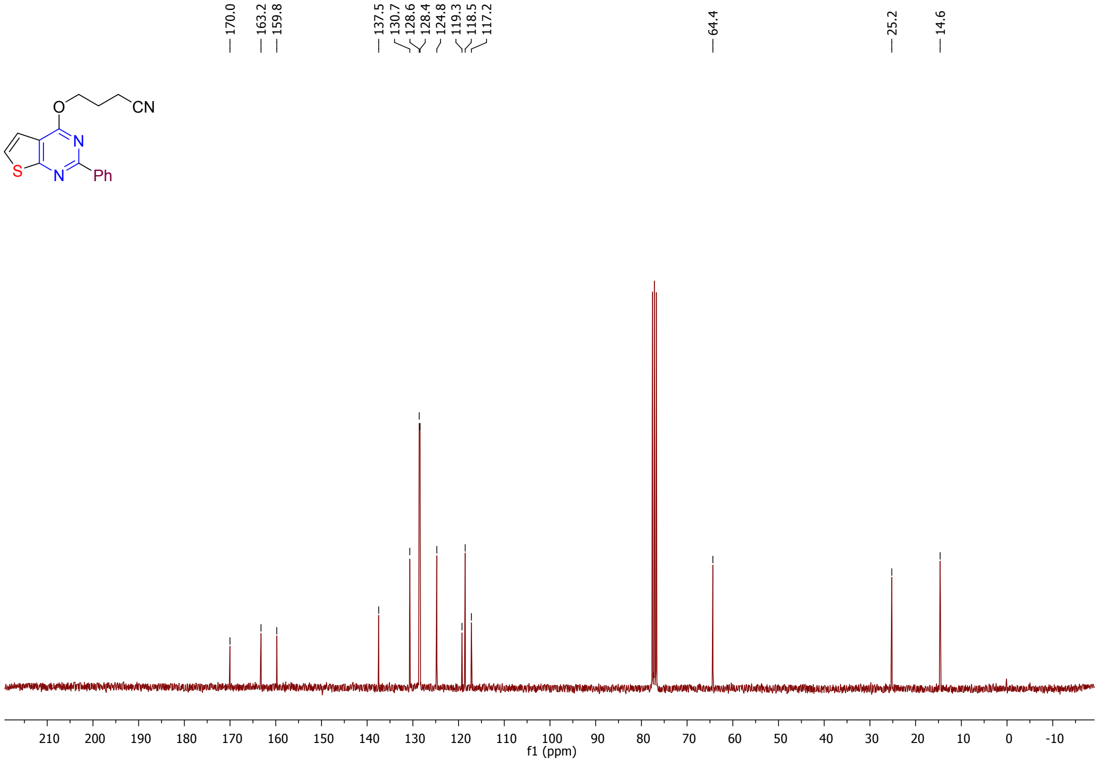

Fig. S-112: ^{13}C NMR Spectrum of **7g**

File :C:\MSDChem\1\DATA\2019\Dr.Abbas Hassan\Sania Batool [illegible]
11-19.D
Operator : Saqib Yasin
Instrument : Instrument #1
Acquired : 19 Nov 2019 8:56 using AcqMethod LIQUID 50 TO 500.M
Sample Name: SB-46
Misc Info : temp 120-280C 10 C/min Flow 1.5ml/min inj 5ul

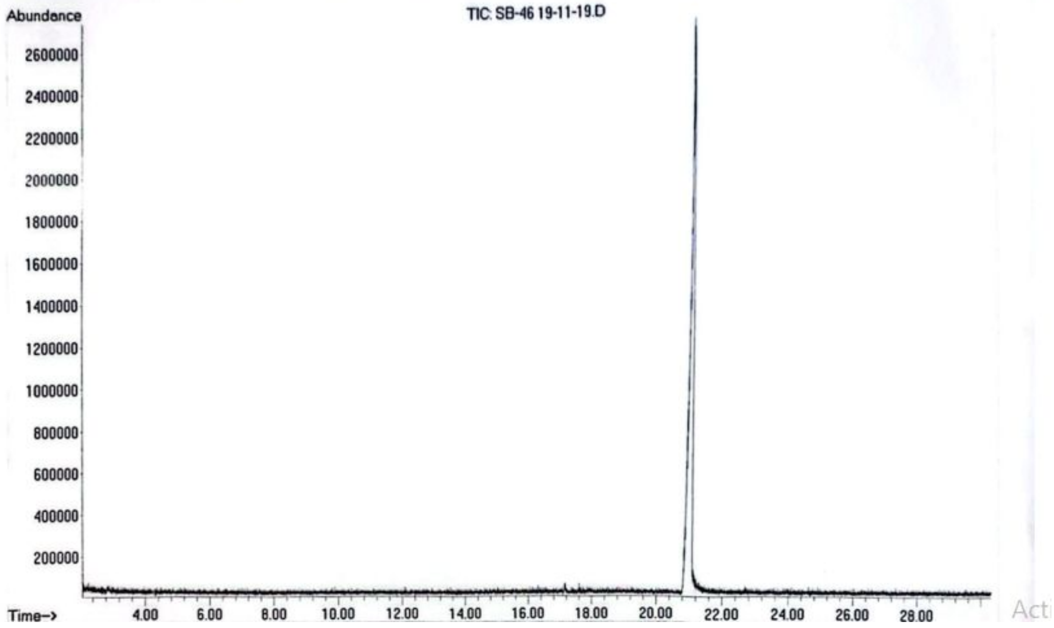

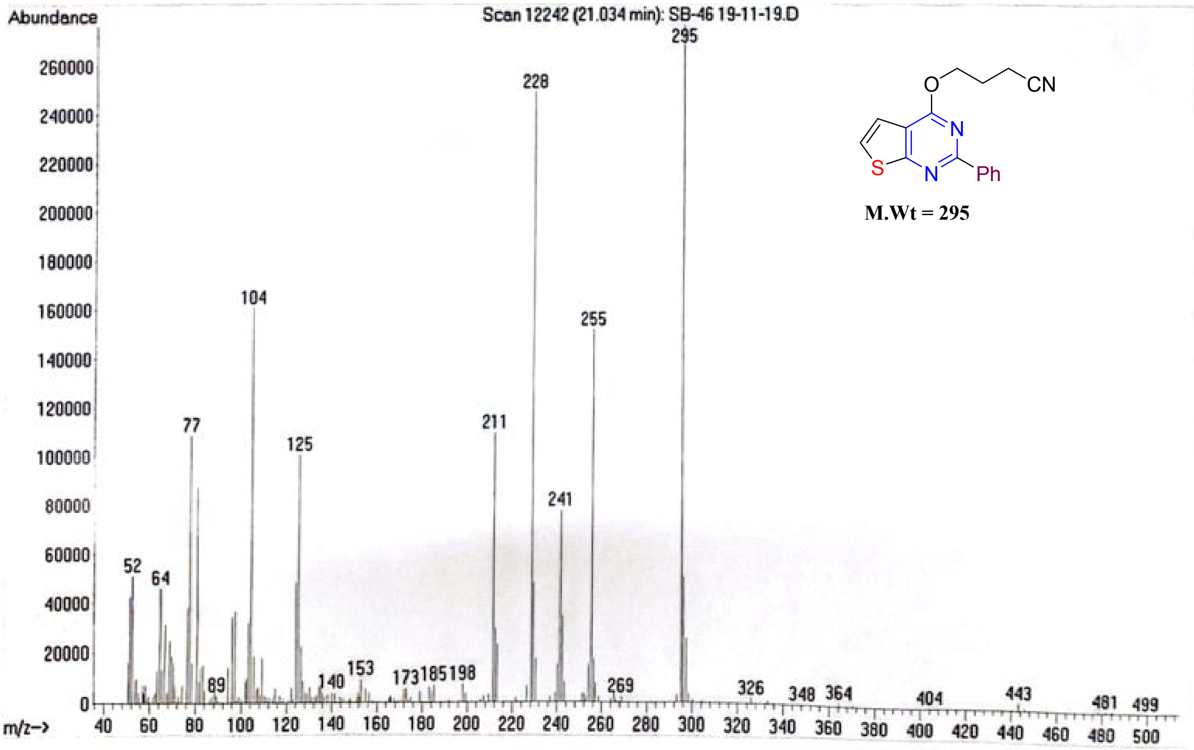

Fig. S-113: GCMS Spectrum of **7g**

Ethyl 5-((2-phenylthieno[2,3-*d*]pyrimidin-4-yl)oxy)pentanoate (7h)

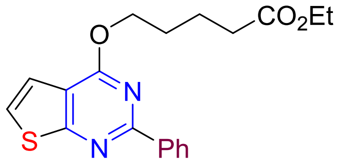

Yield: 77 %

mp = 136-139 °C

IR (*v,* cm^{-1}) 3133-3087, 2876-2814, 1728, 1587-1377, 1289-1024, 717-649

^{1}H NMR (300 MHz, $CDCl_3$): δ (ppm) 8.52-8.49 (m, 2H), 7.53-7.46 (m, 3H) 7.37-7.31 (m, 2H), 4.70-4.66 (t, J = 6.0 Hz, 2H), 4.17-4.10 (q, J = 6.0 Hz, 2H), 2.46-2.41 (t, J = 6.0 Hz, 2H), 2.01-1.82 (m, 4H), 1.28-1.23 (t, J = 6.0 Hz, 3H)

^{13}C NMR (75 MHz, $CDCl_3$): δ (ppm) 173.5, 169.6, 163.7, 159.8, 137.7, 130.5, 128.5, 128.4, 124.2, 118.7, 117.3, 66.2, 60.5, 34.0, 28.4, 21.8, 14.3

GC-MS Analysis (m/z): M^+ = 356, 228, 129, 77, 51

HRMS-ESI (m/z): $[M+H]^+$ calc'd for $C_{19}H_{21}N_2O_3S^+$, 357.1294; found, 357.1298

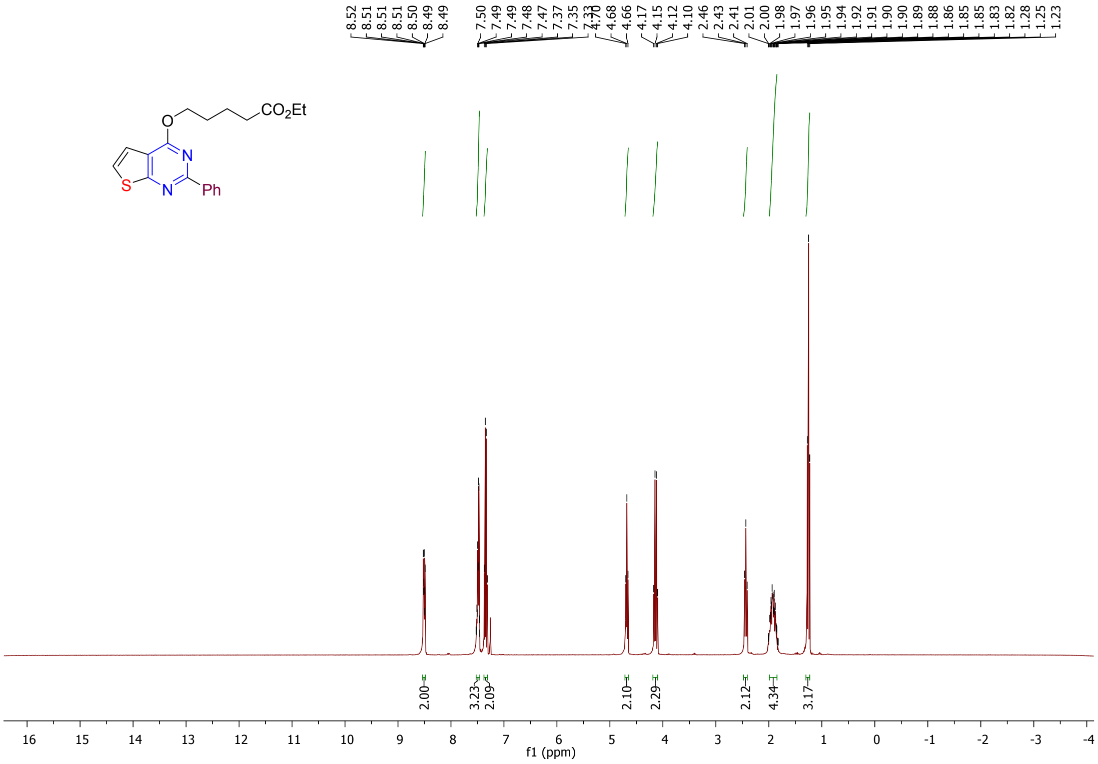

Fig. S-114: ^{1}HNMR Spectrum of **7h**

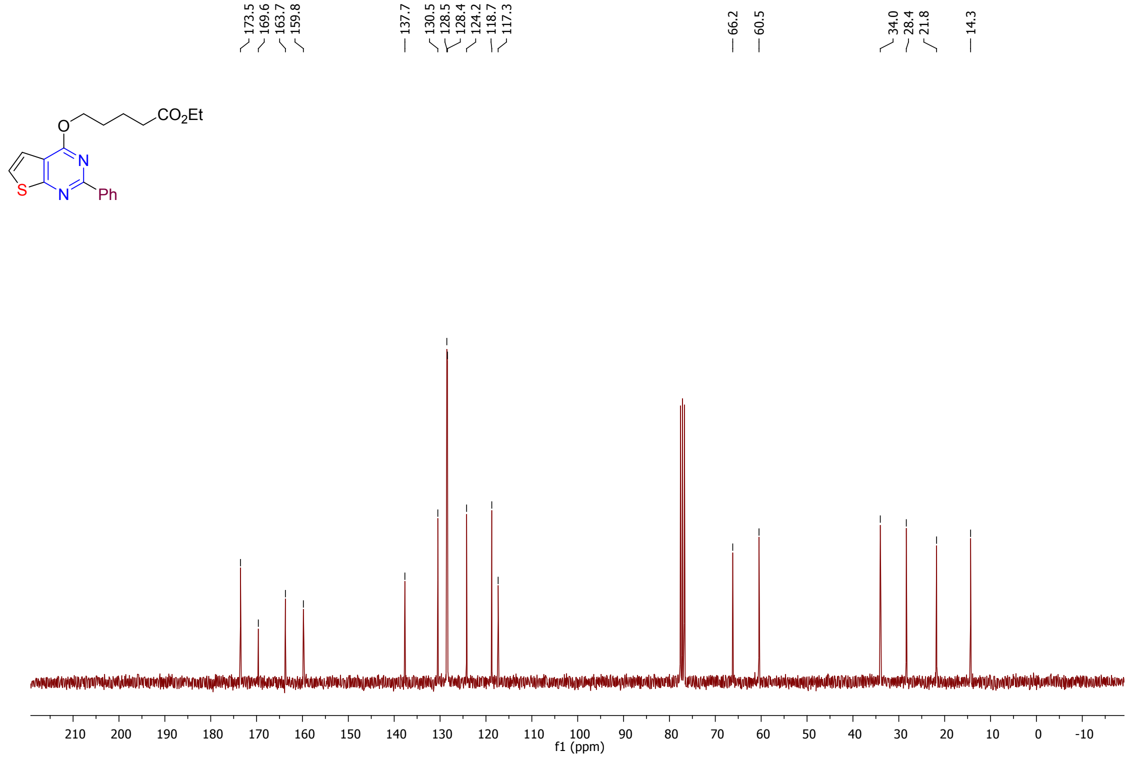

Fig. S-115: ^{13}C NMR Spectrum of **7h**

Ethyl 6-((2-phenylthieno[2,3-*d*]pyrimidin-4-yl)oxy)hexanoate (7i)

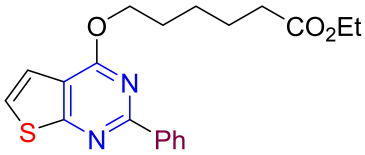

Yield: 83%

IR (*v*, cm^{-1}) 3066, 2986-2870, 1727, 1586-1372, 1322-1039, 927-664

^{1}H NMR (300 MHz, $CDCl_3$): δ (ppm) 8.52-8.49 (m, 2H), 7.52-7.45 (m, 3H) 7.38-7.36 (d, *J* = 6.0 Hz, 1H), 7.34-7.32 (d, *J* = 6.0 Hz, 1H), 4.69-4.65 (t, *J* = 6.0 Hz, 2H), 4.17-4.09 (q, *J* = 9.0, 2H), 2.38-2.33 (t, *J* = 6.0 Hz, 2H), 1.99-1.89 (quint., *J* = 6.0 Hz, 2H), 1.81-1.71 (quint., *J* = 6.0 Hz, 2H), 1.62-1.52 (m, 2H), 1.27-1.22 (t, 3H, *J* = 9.0 Hz)

^{13}C NMR (75 MHz, $CDCl_3$): δ (ppm) 173.7, 169.7, 163.9, 159.9, 137.8, 130.5, 128.6, 128.4, 124.2, 118.8, 117.4, 66.5, 60.4, 34.4, 28.7, 25.8, 24.9, 14.4

GC-MS Analysis (m/z): M^+ = 370, 228, 143, 125, 97, 77, 51

HRMS-ESI (m/z): $[M+H]^+$ calc'd for $C_{20}H_{23}N_2O_3S^+$, 371.1451; found, 371.1454

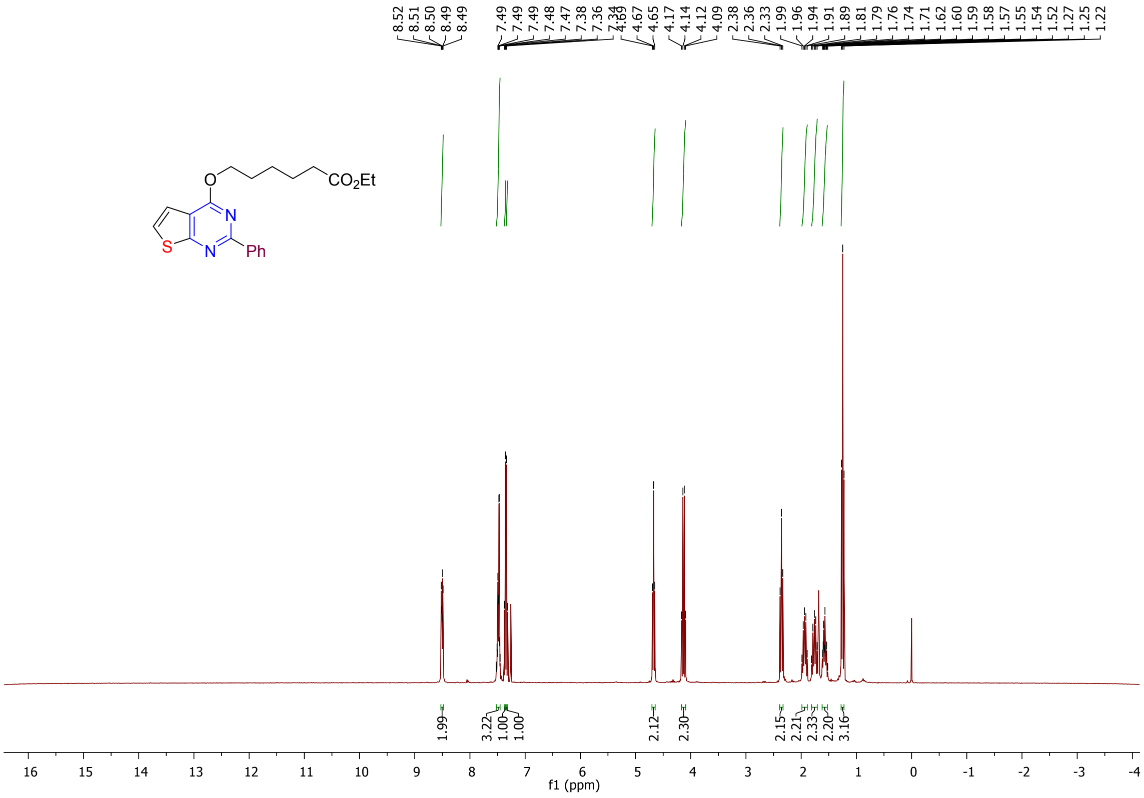

Fig. S-116: ^{1}HNMR Spectrum of **7i**

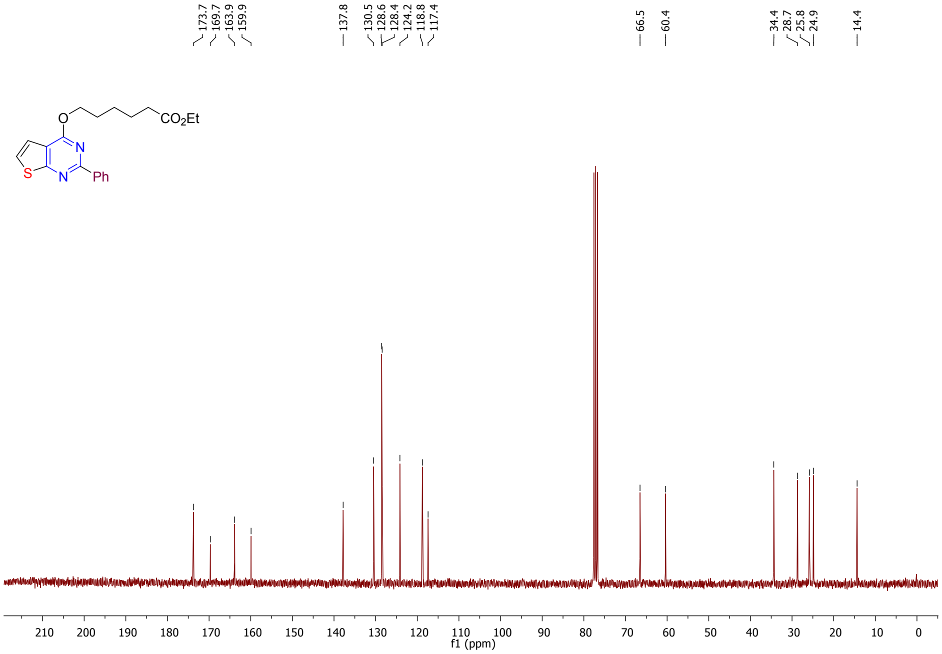

Fig. S-117: ^{13}C NMR Spectrum of **7i**

File :C:\MSDChem\1\DATA\2019\Dr.Abbas Hassan\Sania Batool\SB-47 19-11-19.D
Operator : Saqib Yasin
Instrument : Instrument #1
Acquired : 19 Nov 2019 9:29 using AcqMethod LIQUID 50 TO 500.M
Sample Name: SB-47
Misc Info : temp 120-280C 10 C/min Flow 1.5ml/min inj 5ul

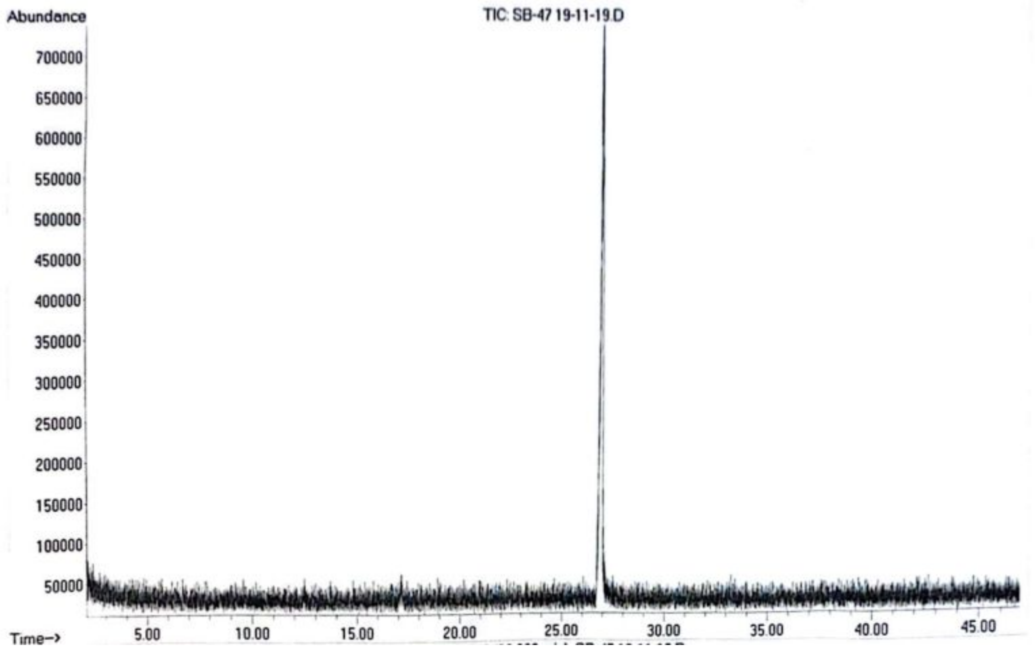

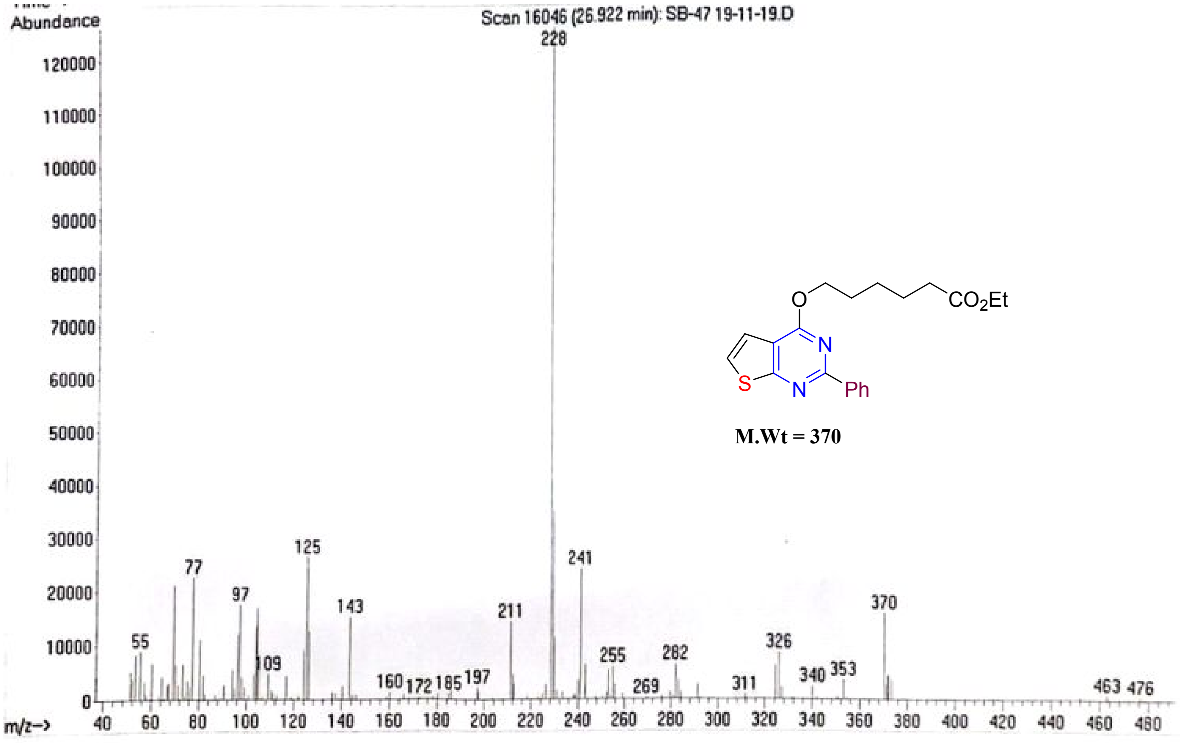

Fig. S-118: GCMS Spectrum of **7i**

Ethyl 2-((2-phenylthieno[2,3-*d*]pyrimidin-4-yl)oxy)acetate (7j)

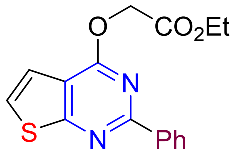

Yield: 83%

mp = 125-128 °C

IR (*v,* cm^{-1}) 3122-3091, 1725, 1571-1392, 1298-1018, 714-659

^{1}H NMR (300 MHz, $CDCl_3$): δ (ppm) 8.47-8.44 (m, 2H), 7.48-7.38 (m, 5H), 5.13 (s, 2H), 4.31-4.24 (q, *J* = 6.0 Hz, 2H), 1.30-1.25 (t, *J* = 6.0 Hz, 3H)

^{13}C NMR (75 MHz, $CDCl_3$): δ (ppm) 170.4, 168.6, 162.6, 159.4, 137.3, 130.7, 128.6, 128.4, 125.0, 118.7, 117.1, 63.2, 61.5, 14.4

GC-MS Analysis (m/z): M^{+} = 314, 241, 211, 77

HRMS-ESI (m/z): $[M+H]^+$ calc'd for $C_{16}H_{15}N_2O_3S^+$, 315.3592; found, 315.3596

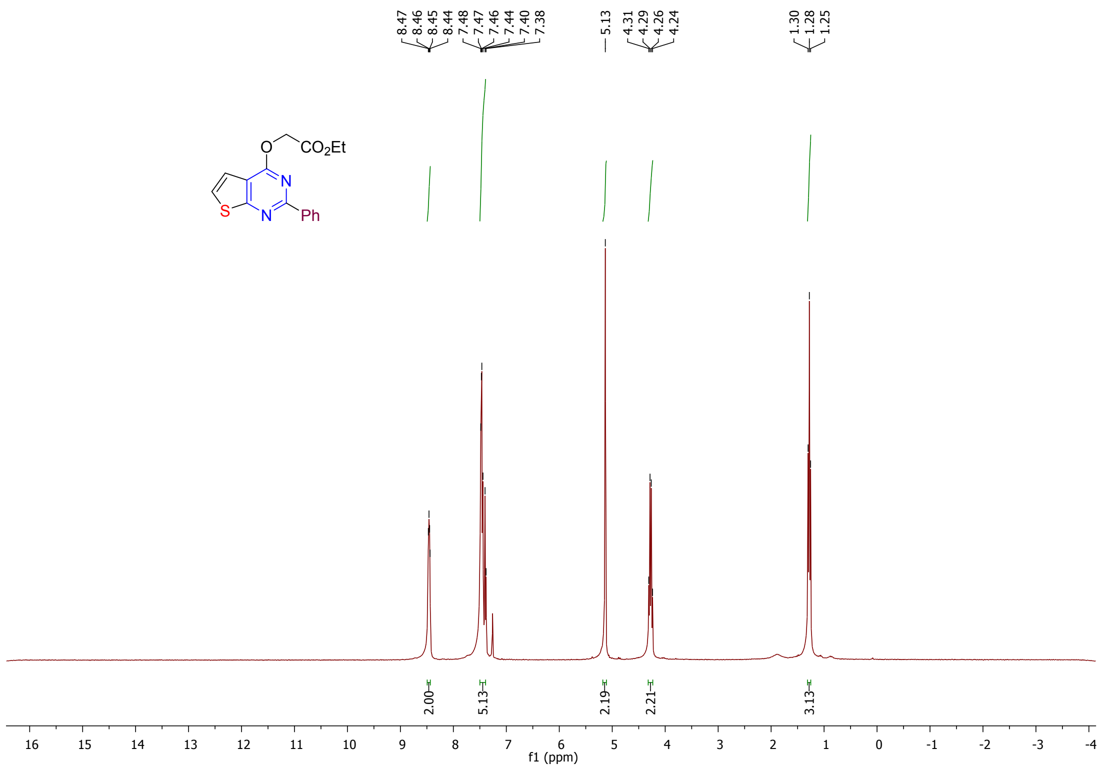

Fig. S-119: ^{1}HNMR Spectrum of **7j**

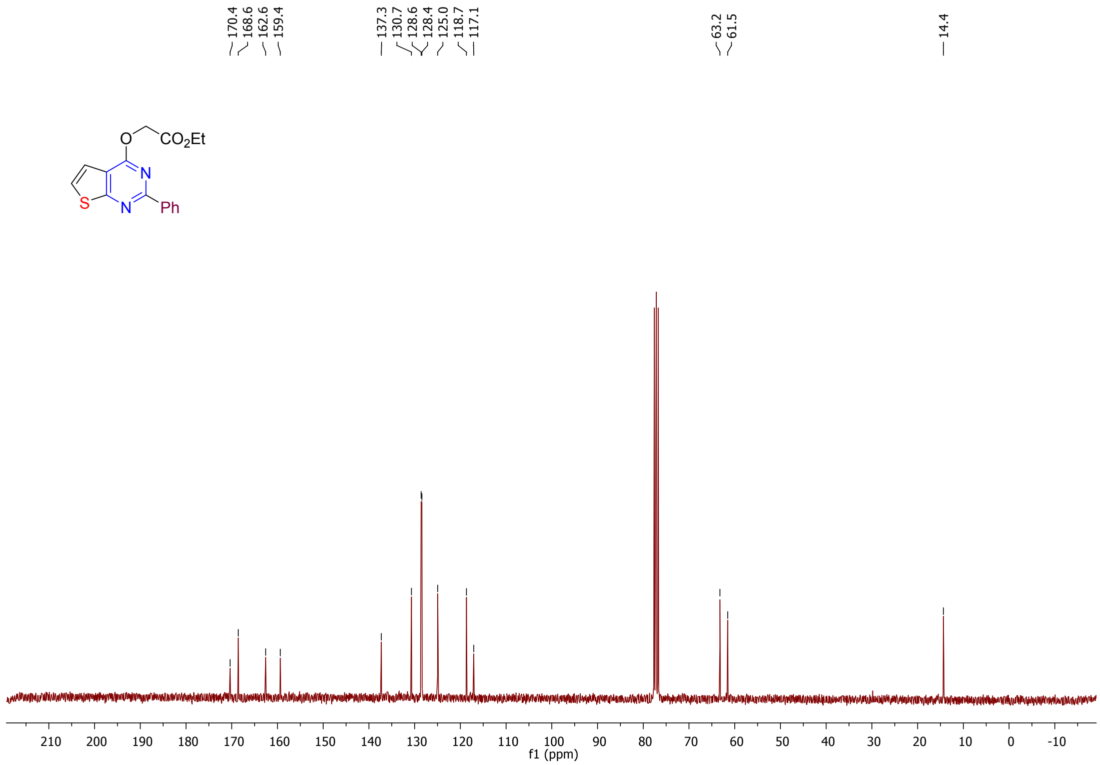

Fig. S-120: ^{13}C NMR Spectrum of **7j**

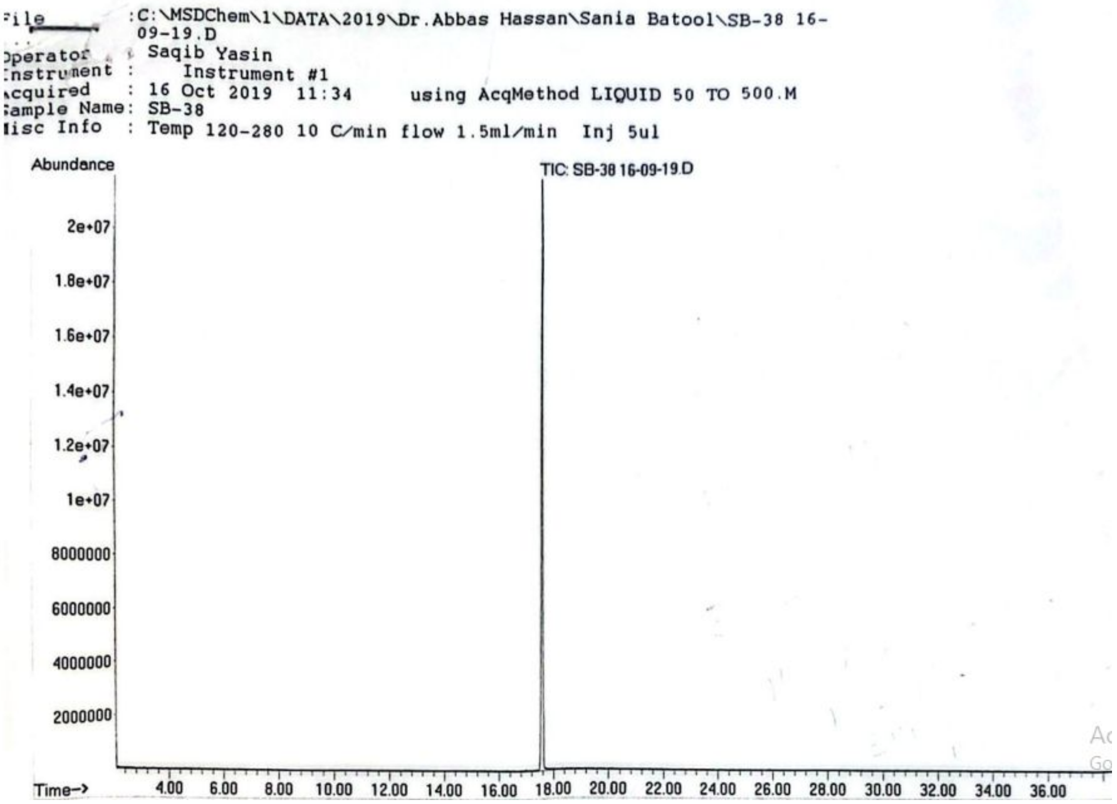

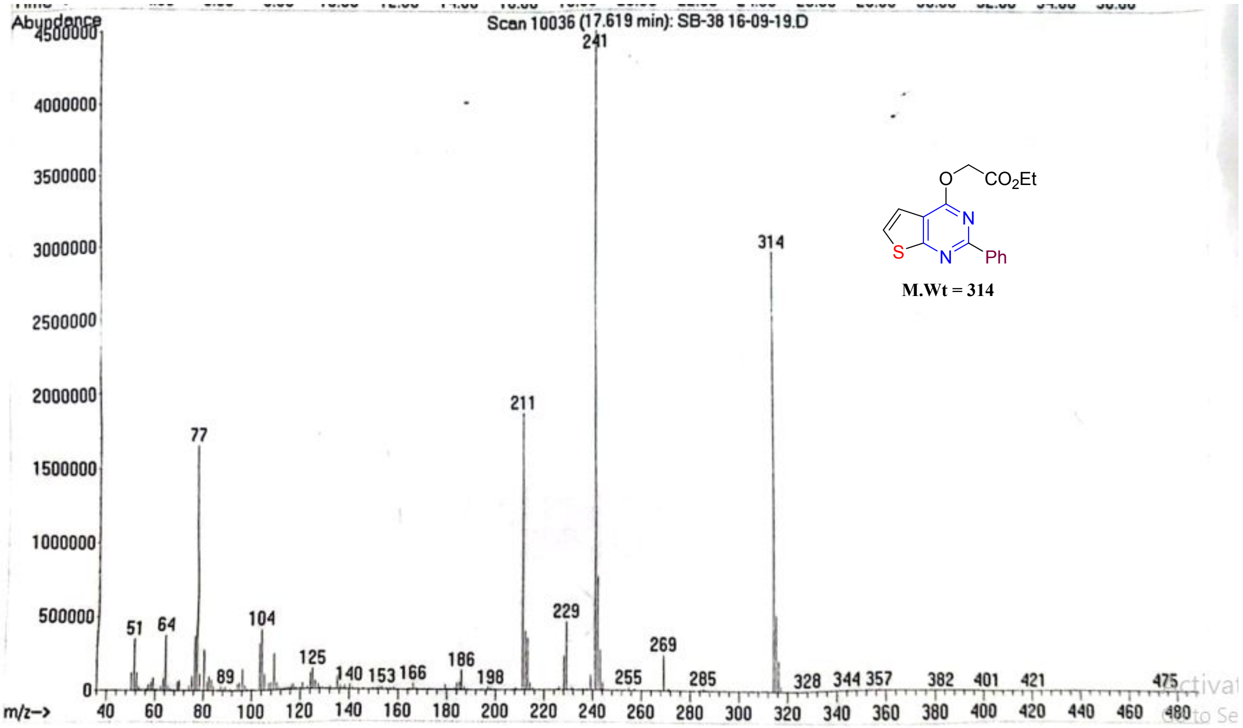

Fig. S-121: GCMS Spectrum of **7j**

Ethyl 2-((2-methylthieno[2,3-*d*]pyrimidin-4-yl)oxy)acetate (7k)

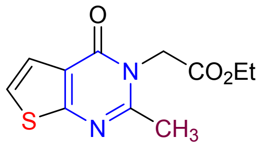

Yield: 68%

mp = 149-151 °C

IR (*v,* cm^{-1}) 2973, 1744, 1657, 1567-1473, 694

^{1}H NMR (300 MHz, $CDCl_3$): δ (ppm) 7.43-7.41 (d, *J* = 6.0 Hz, 1H), 7.18-7.16 (d, *J* = 6.0 Hz, 1H), 4.87 (s, 2H), 4.29-4.22 (q, *J* = 6.0 Hz, 2H), 2.54 (s, 3H), 1.32-1.27 (t, *J* = 6.0 Hz, 3H)

^{13}C NMR (75 MHz, $CDCl_3$): δ (ppm) 167.6, 163.6, 158.4, 154.4, 123.0, 122.4, 122.0, 62.3, 45.2, 23.2, 14.2

GC-MS Analysis (m/z): M^+ = 252, 206, 178, 150

HRMS-ESI (m/z): $[M+H]^+$ calc'd for $C_{11}H_{13}N_2O_3S^+$, 253.0574; found, 253.0578

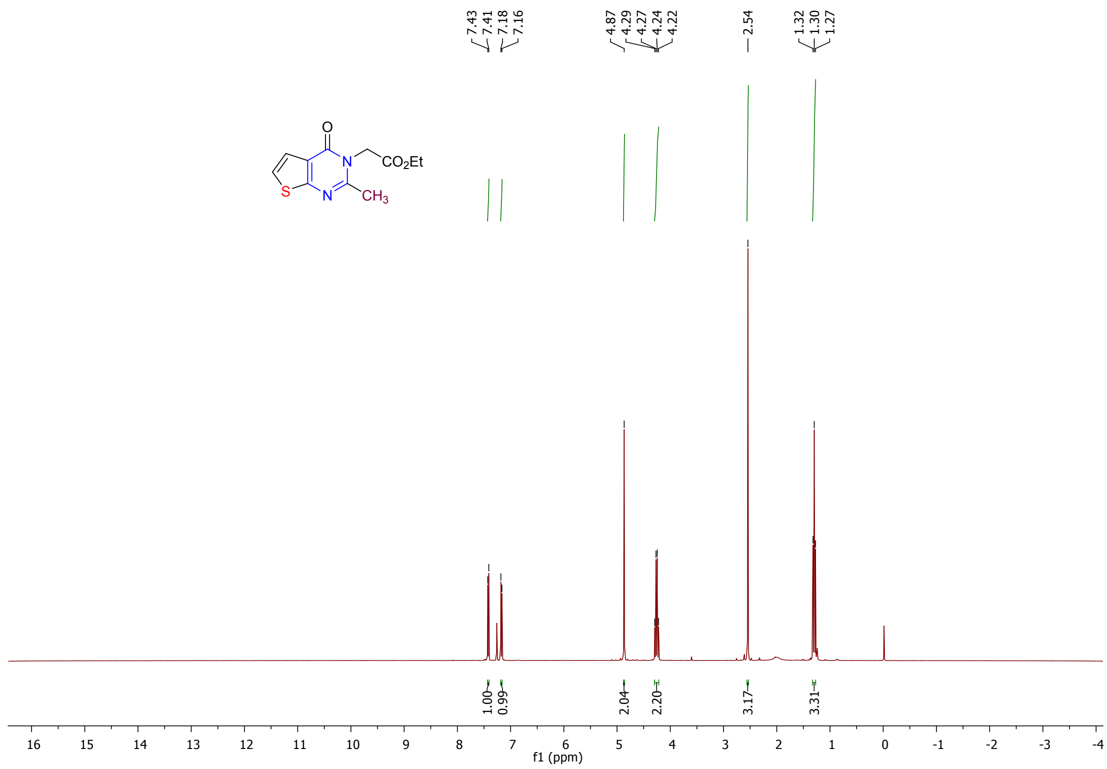

Fig. S-122: ^{1}HNMR Spectrum of **7k**

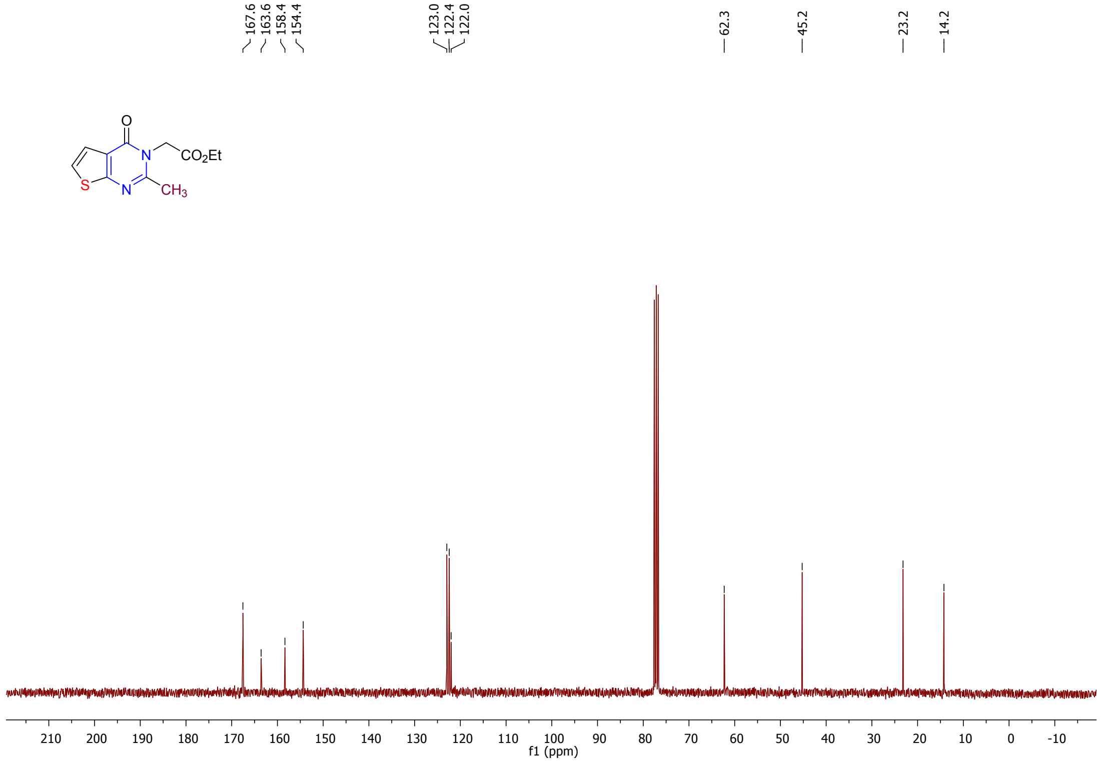

Fig. S-123: ^{13}C NMR Spectrum of **7k**

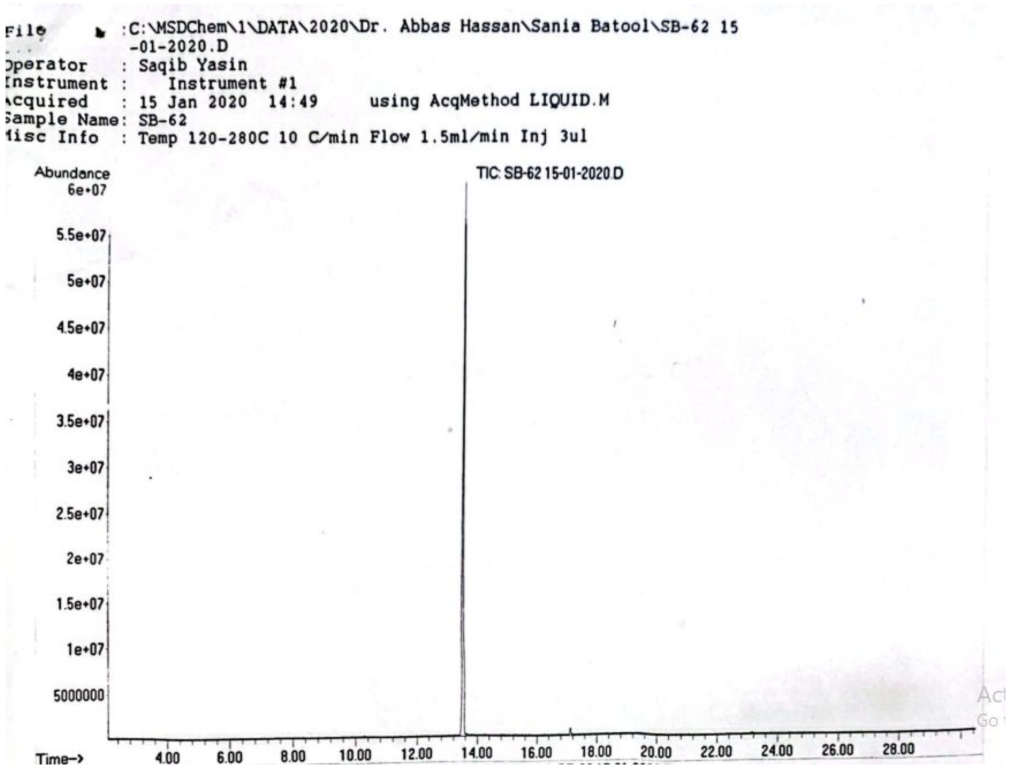

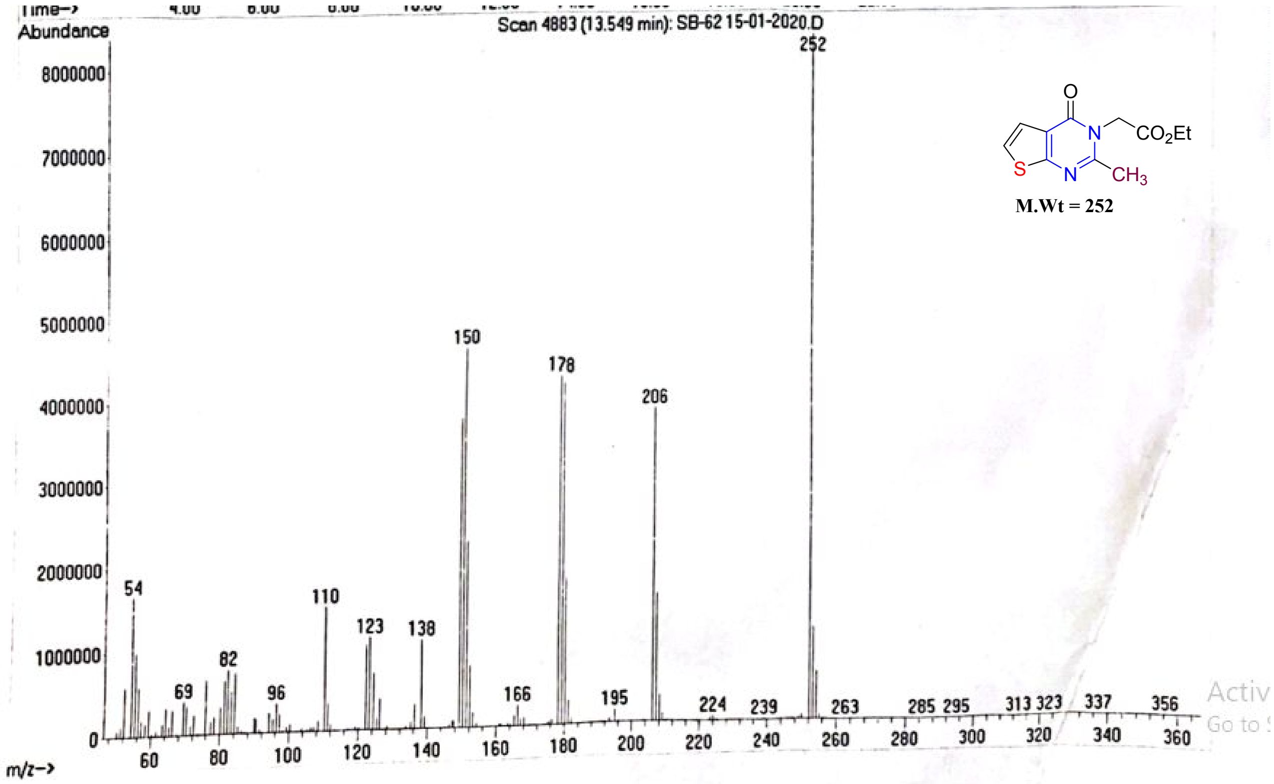

Fig. S-124: GCMS Spectrum of **7k**

5-((2-Phenylthieno[2,3-*d*]pyrimidin-4-yl)oxy)pentanoic acid (8h)

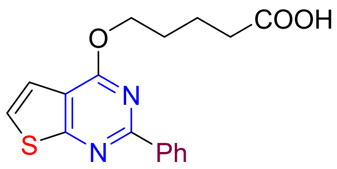

Yield: 79%

mp = 152-155 °C

IR (*v*, cm^{-1}) 3407-2467, 3167-3087, 2865-2817, 1715, 1587-1311, 1274-1018, 719-665

^{1}H NMR (300 MHz, Acetone d_6): δ (ppm) 8.51-8.48 (m, 2H), 7.53-7.46 (m, 3H) 7.38-7.36 (d, J = 6.0 Hz, 1H), 7.34-7.32 (d, J = 6.0 Hz, 1H), 4.71-4.67 (t, J = 6.0 Hz, 2H), 2.52-2.47 (t, J = 9.0 Hz, 2H), 2.01-1.86 (m, 4H)

^{13}C NMR (75 MHz, Acetone d_6): δ (ppm) 179.4, 169.7, 163.8, 159.9, 137.7, 130.5, 128.6, 128.4, 124.3, 118.7, 117.4, 66.2, 33.7, 28.3, 21.5

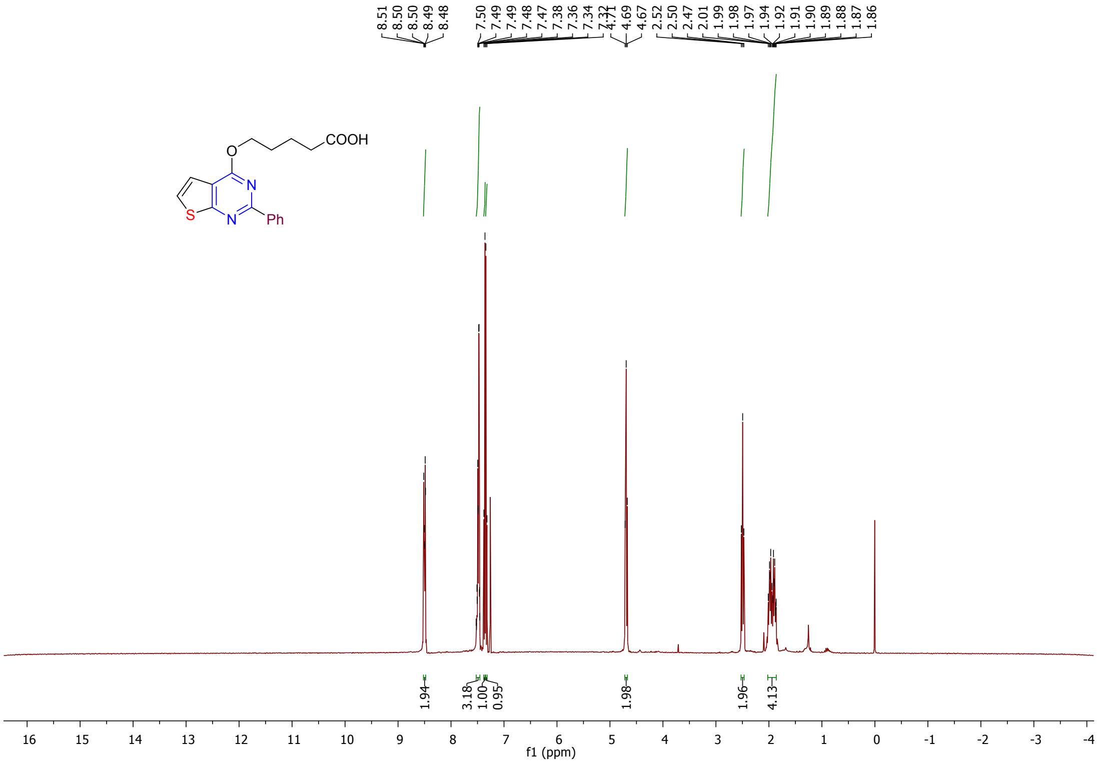

Fig. S-125: ^{1}HNMR Spectrum of **8h**

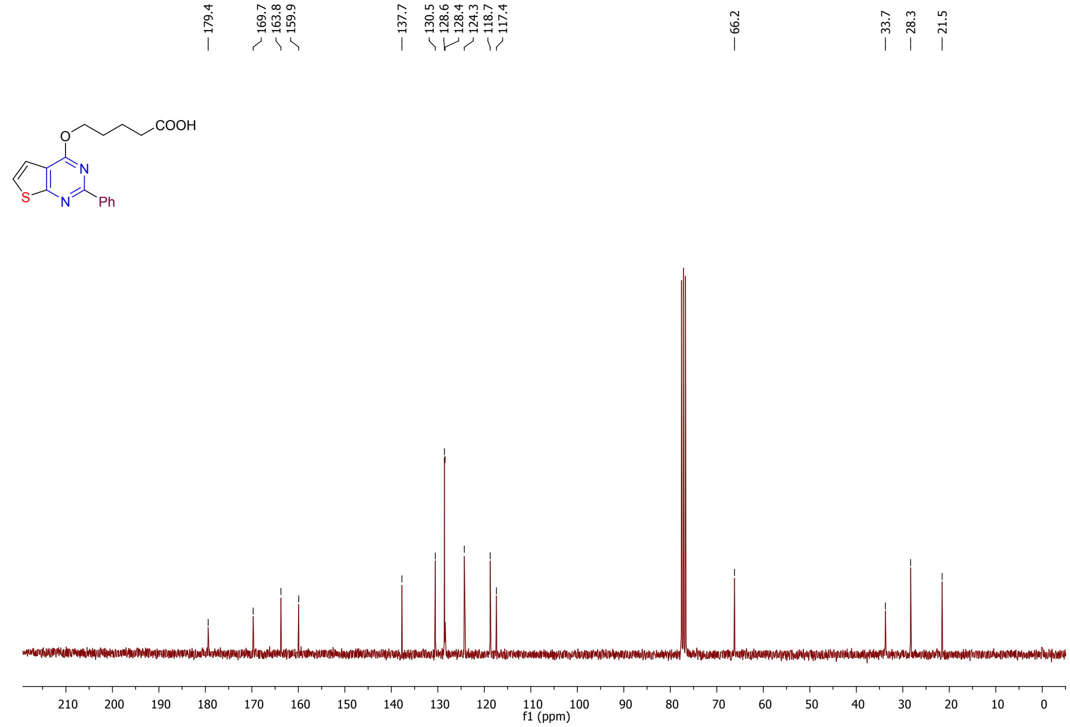

Fig. S-126: ^{13}C NMR Spectrum of **8h**

6-((2-Phenylthieno[2,3-*d*]pyrimidin-4-yl)oxy)hexanoic acid (8i)

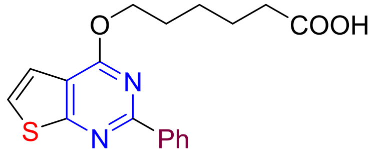

Yield: 72%

mp = 144-147 °C

IR (*v*, cm^{-1}) 3456-2489, 2976-2865, 1716, 1589-1365, 1361-1098, 928-663

^{1}H NMR (300 MHz, Acetone d_6): δ (ppm) 8.55-8.51 (m, 2H), 7.67-7.65 (d, J = 6.0 Hz, 1H), 7.53-7.48 (m, 3H) 7.45-7.43 (d, J = 6.0 Hz, 1H), 4.74-4.70 (t, J = 6.0 Hz, 2H), 2.38-2.33 (t, J = 9.0, 2H), 2.01-1.92 (m, 2H), 1.79-1.69 (m, 2H), 1.66-1.58 (m, 2H)

^{13}C NMR (75 MHz, Acetone d_6): δ (ppm) 174.7, 170.4, 164.6, 160.3, 138.4, 131.3, 129.3, 129.0, 125.8, 119.4, 118.1, 67.4, 34.1, 29.2, 26.3, 25.4

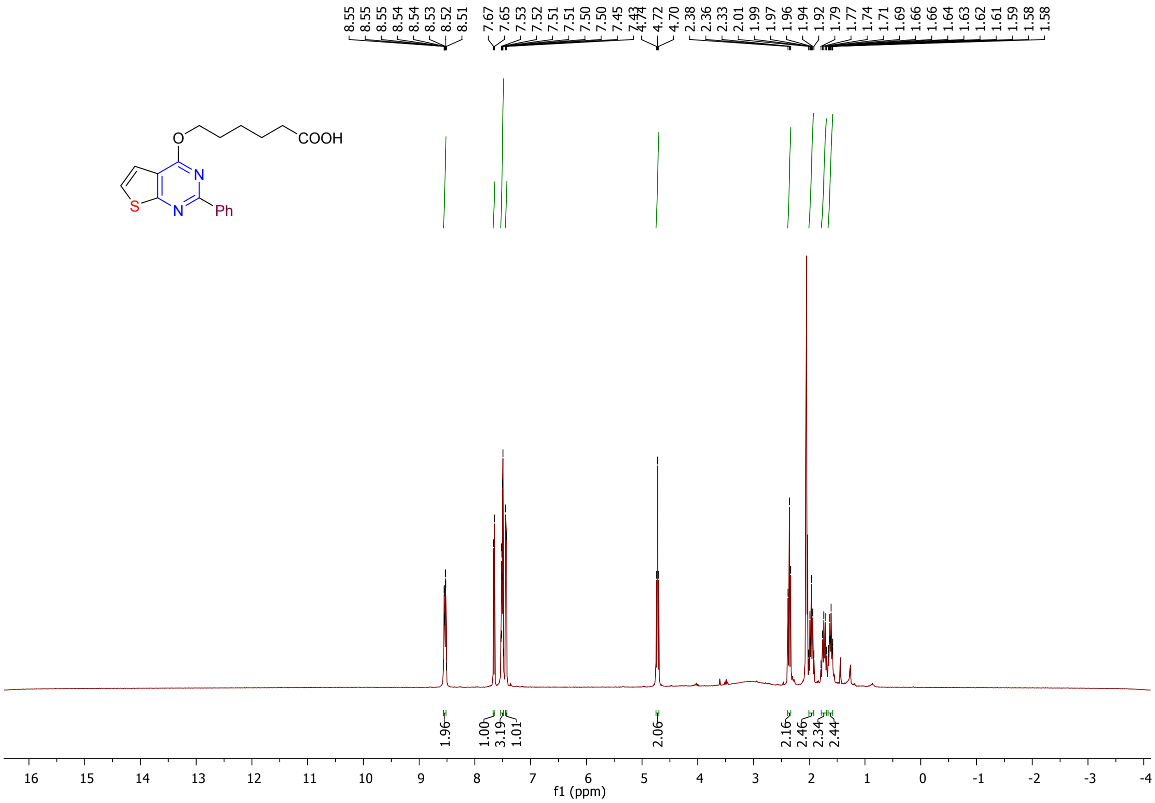

Fig. S-127: ^{1}HNMR Spectrum of **8i**

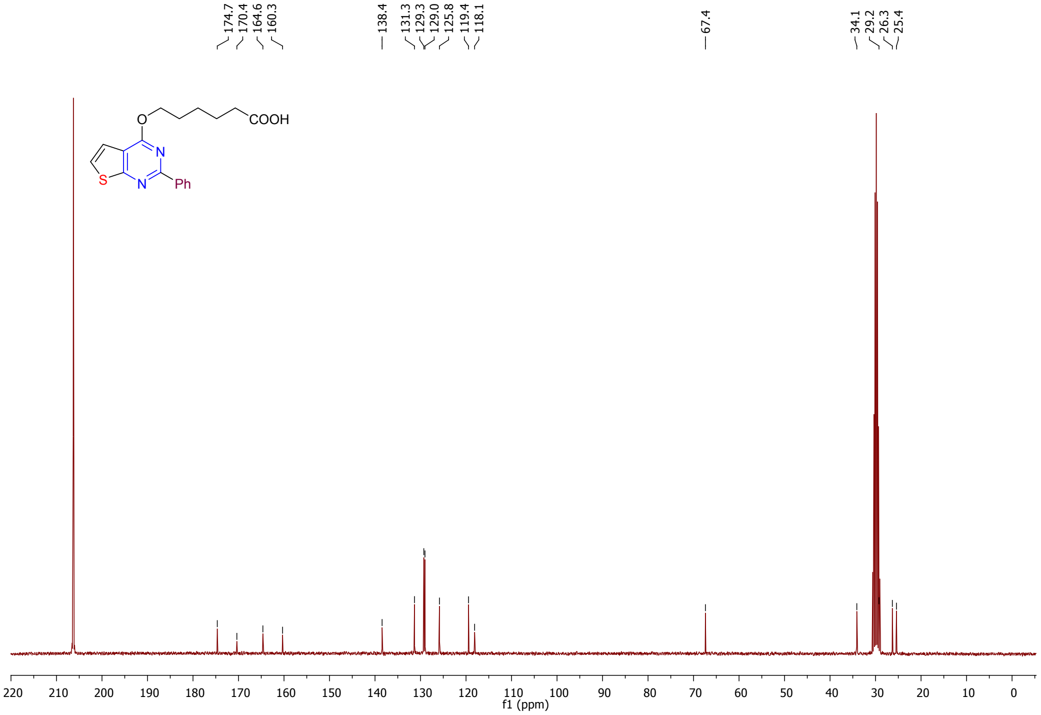

Fig. S-128: ^{13}C NMR Spectrum of **8i**

2-((2-Phenylthieno[2,3-*d*]pyrimidin-4-yl)oxy)acetic acid (8j)

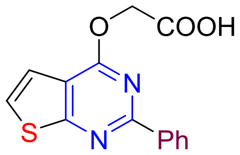

Yield: 81%

mp = 143-147 °C

IR (*v,* cm^{-1}) 3413-2497, 3187-3027, 1718, 1585-1376, 1287-1012, 711-655

^{1}H NMR (300 MHz, Acetone d_6): δ (ppm) 8.52-8.49 (m, 2H), 7.77-7.75 (d, J = 6.0 Hz, 1H), 7.53-7.50 (m, 4H), 3.85-3.88 (m, 4H), 5.27 (s, 2H)

^{13}C NMR (75 MHz, Acetone d_6): δ (ppm) 170.8, 169.6, 163.6, 159.9, 138.0, 131.5, 129.3, 129.0, 126.6, 119.2, 117.8, 63.5

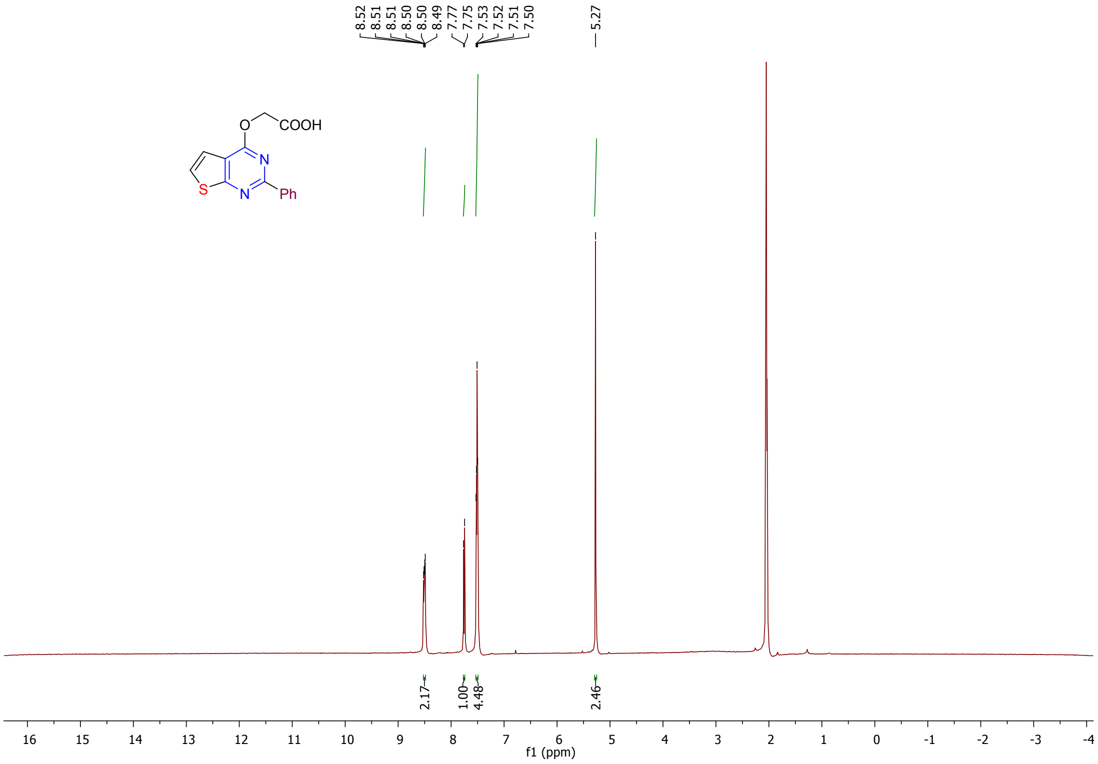

Fig. S-129: ^{1}HNMR Spectrum of **8j**

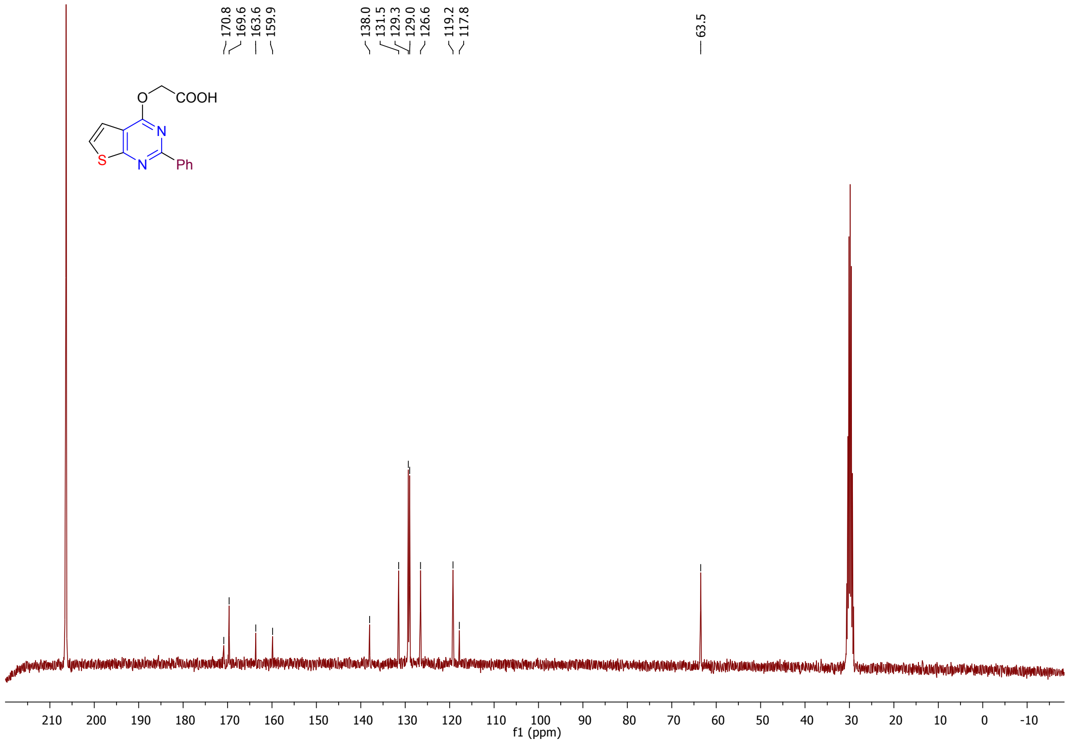

Fig. S-130: ^{13}C NMR Spectrum of **8j**

References:

[1] Hesse, S.; Perspicace, E.; Kirsch, G.; Microwave-assisted synthesis of 2-aminothiophene-3-carboxylic acid derivatives, 3H-thieno[2,3-d]pyrimidin-4-one and 4-chlorothieno[2,3-d]pyrimidine. *Tetrahedron Letters* **2007,** 48 (30), 5261–5264.

[2] Rashad, A, E.; Shamroukh,A, H.; Abdel-Megeid, R, E.; El-Sayed, W, A.; Synthesis, reactions, and antimicrobial evaluation of some polycondensed thienopyrimidine derivatives. *Synthetic Communications* **2010**, 40 (8), 1149–1160.

[3] Theoclitou, M, E.; Discovery of (+)-N-(3-aminopropyl)-N-[1-(5-benzyl-3-methyl-4-oxo-[1,2] thiazolo[5,4- d]pyrimidin-6-yl)-2-methylpropyl]-4-methylbenzamide (AZD4877), a kinesin spindle protein inhibitor and potential anticancer agent. *Journal of Medicinal Chemistry* **2011**, 54 (19) 6734–6750.

[4] Ahmad, L.; Design, synthesis and antimicrobial activity of tricyclic tetrahydro thieno [2,3-d] pyrimidine derivatives. *World Journal of Pharmaceutical Sciences* **2015,** 3 (3), 463-467.

[5] Kanawade, S.; Toche, B.; Rajani, P.; Synthetic tactics of new class of 4-aminothieno[2,3-d]pyrimidine-6-carbonitrile derivatives acting as antimicrobial agents. *European Journal of Medicinal Chemistry* **2013**, 64, 314–320.

[6] Nirogi, S.; Convenient and efficient synthesis of some novel fused thieno pyrimidines using gewald's reaction. *Synthetic Communications* **2011**, 41 (19), 2835–2851.

[7] Parveen, S.; Modification of Bischler-Möhlau indole derivatives through palladium catalyzed Suzuki reaction as effective cholinesterase inhibitors, their kinetic and molecular docking studies. *Bioorganic Chemistry* **2018**, 76, 166–176.

[8] Loubidi, M.; Moutardier, A.; Campos, F.; Berteina-Raboin, S.; Pd-catalyzed Suzuki/Sonogashira cross-coupling reaction and the direct sp3 arylation of 7-chloro-5-methyl-[1,2,4]triazolo[1,5-a]pyrimidine. *Tetrahedron Letters* **2018,** 59 (11), 1050–1054.

[9] Sirisha, B.; Synthesis and theoretical studies on energetics of novel N- and O- perfluoroalkyl triazole tagged thienopyrimidines - Their potential as adenosine receptor ligands. *European Journal of Medicinal Chemistry* **2010**, 45 (5), 1739–1745.

[10] Shao, X.; Chemical Space Exploration around Thieno[3,2- d]pyrimidin-4(3 H)-one Scaffold Led to a Novel Class of Highly Active Clostridium difficile Inhibitors. *Journal of Medicinal Chemistry* **2020**, 62 (21), 9772–9791.

[11] Kawasaki, N.; Hayato, F.; Ishihara, J.; Concise Synthesis of TPCA-1 and Related Thiophene-carboxamides by Cross Coupling. *Heterocycles* **2020,** 2, 707-716.

[12] SeongShick, R.; Sim, K.; Song, C.; Shin, I.; Kim, Lee, S.; Y.; Park, J.; Sim, T.; Anti-glioma effects of 2-aminothiophene-3-carboxamide derivatives, ANO1 channel blockers. *European Journal of Medicinal Chemistry* **2020**, 208, 112688.

[13] Kuwabara, J.; Sawada, Y.; Yoshimatsu, M.; Nitrile hydration reaction using copper iodide/cesium carbonate/DBU in nitromethane–water. *Synlett* **2018**, 29 (15), 2061-2065.

[14] Riabova, O.; Egorova, A.; Lepioshkin, A.; Voigt, K.; Kloss, F.; Makarov,V.; Thieno [2, 3-d] pyrimidine-Core Compounds Show Activity against Clinically Relevant Gram-Positive Bacteria. *ChemMedChem* **2022**, 17 (17), e202200207.

[15] Bulletin de la societe chimique de France, **1975**, p. 815-819

[16] Katia, S.; Lassagne, F.; Bentabed-Ababsa, G.; Nassar, E.; Sidaty, C.; Stéphanie, H.; Perspicace, E.; Derdour, A; Mongin, F.; Direct metallation of thienopyrimidines using a mixed lithium–cadmium base and antitumor activity of functionalized derivatives. *Organic & Biomolecular Chemistry* **2009**, 22, 4782-4788.

[17] Desroches, J.; Kieffer, C.; Primas, N.; Hutter, S.; Gellis, A.; El-Kashef, H.; Rathelot, P.; Verhaeghe, P.; Azas, N.; Vanelle, P.; Discovery of new hit-molecules targeting Plasmodium falciparum through a global SAR study of the 4-substituted-2-trichloromethylquinazoline antiplasmodial scaffold. *European journal of medicinal chemistry* **2017***,* 125, 68-86.

[18] Antczak, M. I.; Zhang, Y.; Wang, C.; Doran, J.; Naidoo, J.; Voruganti, S.; Ready, J. M.; Inhibitors of 15-prostaglandin dehydrogenase to potentiate tissue repair. *Journal of medicinal chemistry* **2017**, 60 (9), 3979-4001.

[19] Wang, J.; Zha, S.; Chen, K.; Zhang, F.; Song, C.; Zhu, J.; Quinazoline synthesis via Rh (III)-catalyzed intermolecular C–H functionalization of benzimidates with dioxazolones *Organic Letters* **2016**, 18 (9), 2062-2065.

[20] Wang, X.; Lerchen, A.; and Glorius, F.; A comparative investigation: group 9 Cp* M (III)-catalyzed formal [4+ 2] cycloaddition as an atom-economic approach to quinazolines. *Organic Letters* **2016**, 18 (9), 2090-2093.
